# Biological and genomic resources for the cosmopolitan phytoplankton *Bathycoccus*: insights into genetic diversity and function of outlier chromosomes

**DOI:** 10.1111/tpj.70982

**Published:** 2026-06-07

**Authors:** Louis Dennu, Martine Devic, Janaina Rigonato, Angela Falciatore, Jean‐Claude Lozano, Valérie Vergé, Cédric Mariac, Nathalie Joli, Olivier Jaillon, François Sabot, François‐Yves Bouget

**Affiliations:** ^1^ Laboratoire d'Océanographie Microbienne (LOMIC) CNRS/Sorbonne Université, UMR 7621, Observatoire Océanologique 66650 Banyuls/mer France; ^2^ Génomique Métabolique Genoscope, Institut de Biologie François Jacob, Commissariat à l'Energie Atomique (CEA), CNRS, Université Evry, Université Paris‐Saclay Evry France; ^3^ Laboratory of Chloroplast Biology and Light Sensing in Microalgae Institut de Biologie Physico Chimique, CNRS, Sorbonne Université 75005 Paris France; ^4^ Diversité, Adaptation et Développement des Plantes (DIADE) IRD/UM/CIRAD, UMR 232, Centre IRD de Montpellier 911 Avenue Agropolis, BP 604501 34394 Montpellier Cedex 5 France; ^5^ Institut de Biologie de l'École Normale Supérieure (IBENS), École Normale Supérieure, CNRS, INSERM, PSL Université Paris 75005 Paris France

**Keywords:** phytoplankton, *Mamiellophyceae*, natural diversity, whole‐genome sequencing, *Bathycoccus*, structural variations

## Abstract

Population‐scale genome sequencing has become essential for exploring genetic diversity and adaptation, particularly in land plants. In contrast, eukaryotic phytoplankton resources remain limited to model reference genomes or community‐level metagenomics, leaving a gap in understanding intraspecific variation and evolutionary processes. To address this, we developed a comprehensive biological and genomic resource for the cosmopolitan and ecologically important genus *Bathycoccus*. Extensive metagenomic data from across the world Ocean are available for this genus, and previous studies have identified four *Bathycoccus* species and reconstructed 34 metagenome‐assembled genomes (MAGs). Here we report 28 high‐quality strain genome sequences using a combination of Oxford Nanopore Technologies long reads and Illumina short reads and associated biological resources. These include 24 *Bathycoccus prasinos* strains spanning a latitudinal gradient from 40° to 78° N, a reference genome for *Bathycoccus calidus*, and three genomes of the recently identified B3 clade, which we propose as the *Bathycoccus catiminus* species. Comparative analyses of sequenced genomes with MAGs highlight the complementarity between resources: While MAGs capture environmental diversity and uncover uncultured taxa, the cultured strain genomes provide complete, non‐chimeric high‐quality assemblies that resolve structural variations and haplotype‐level diversity not detected in MAGs. These include the big outlier chromosome, a putative sexual chromosome revealing a second mating type, and extensive variability in the small outlier chromosome, associated with viral resistance and genome plasticity. Together, these biological and genomic resources establish *B. prasinos* as a powerful model for studying diversity, adaptation, and evolution of eukaryotic phytoplankton in the ocean, complementing existing global metagenomic datasets.

## Significance Statement

Eukaryotic phytoplankton are key to ocean ecosystems, yet their intraspecific genomic diversity is poorly understood. We present 28 high‐quality genomes and their annotations of the cosmopolitan microalga *Bathycoccus*, revealing whole‐genome structural variations, chromosomal haplotype diversity linked to mating and viral resistance, and offering a genomic framework that complements metagenomic data to establish this picoalga as a model for functional and ecological studies.

## INTRODUCTION

Accessing the natural diversity of a species constitutes an invaluable resource, providing insights into its evolutionary history and facilitating correlations between phenotypes and genotypes through functional studies. In plants, the availability of extensive collections of natural accessions and crop cultivars from diverse environments has greatly improved our knowledge of adaptation (Alonso‐Blanco et al., 2009). Furthermore, large‐scale sequencing projects on widely distributed models, such as for *Arabidopsis thaliana*, and in several model crops, have provided a comprehensive framework for the study of genomic diversity, its underlying mechanisms and their impact on environmental adaptation (3,000 Rice Genomes Project, 2014; Alonso‐Blanco et al., 2016; The 100 Tomato Genome Sequencing Consortium et al., 2014). Other reference organisms have also emerged as models in population genomics, such as *Saccharomyces cerevisiae* for which extensive sequencing of available strains highlighted complex genomic structures with great diversity (Peter et al., 2018). While these comprehensive sequencing projects have benefited their research communities, the study of eukaryotic phytoplankton lags behind in terms of comparable large‐scale sequencing initiatives on single species. Pioneer studies have been conducted for the marine diatom *Phaeodactylum tricornutum* or the freshwater and estuarine haptophyte *Prymnesium parvum* (Rastogi et al., 2020; Wisecaver et al., 2023). However, in most currently available pangenomic studies of eukaryotic phytoplankton, descriptions of intraspecific diversity are limited by the presence of cryptic species within taxa that were initially separated only as morphospecies, as exemplified for *Emiliana huxleyi* (*Gephyrocapsa huxleyi*) (Bendif et al., 2023; Read et al., 2013), *Microcystis* (Cai et al., 2023) or *P. parvum* (Wisecaver et al., 2023). This highlights current challenges in the study of phytoplankton intraspecific diversity, as the taxonomic resolution and large morphological diversity of many phytoplankton taxa must be taken into account when characterizing intraspecific subpopulations and distinguishing cryptic species that have undergone speciation events. Although access to intraspecific diversity in eukaryotic phytoplankton is constrained by the limited availability of biological resources for most species, the rise of large metagenomic datasets over the past 15 years has enabled the reconstruction of metagenome‐assembled genomes (MAGs), providing a complementary resource for exploring diversity (Delmont et al., 2022; Duncan et al., 2022; Xu et al., 2024).

The global distribution and high abundance of phytoplankton species make them major actors of the planet's primary production, contributing as much as land plants (Li, 1994; Worden et al., 2004). In particular, eukaryotic phytoplankton species are characterized by their great taxonomic diversity and relatively short generation times allowing for their ecological success in a wide range of environmental conditions in the world Ocean (De Vargas et al., 2015; Lynch et al., 1991). Microalgae also serve as valuable models for both fundamental studies and biotechnological research. Among eukaryotic phytoplankton, the *Mamiellophyceae* class stands out as unicellulars widely distributed in the oceans from poles to equator and marked by seasonal population dynamics in polar and temperate regions (Lambert et al., 2019; Leconte et al., 2020). *Mamiellophyceae* diverged at the base of the green lineage, making them attractive models for studying essential cellular functions from an evolutionary perspective (Leliaert et al., 2012; Yung et al., 2022). The early whole‐genome sequencing of *Ostreococcus tauri* and the implementation of genetic transformation tools, including gene targeting by homologous recombination, have established *O. tauri* as a model for the study of several cellular pathways such as the circadian clock, cell division, iron metabolism and vitamin B1 metabolism (Derelle et al., 2006; Corellou et al., 2009; Moulager et al., 2010; O'Neill et al., 2011; Lozano et al., 2014; Botebol et al., 2015; Paerl et al., 2017; De Barros Dantas et al., 2023). The genomic content of *O. tauri* unveiled several features shared by *Mamiellophyceae*: a compact haploid genome, a majority of mono‐exonic genes, limited gene redundancy and two outlier chromosomes, characterized by lower GC content and unique gene structures, named big outlier chromosome (BOC) and small outlier chromosome (SOC), respectively (Derelle et al., 2006; Grimsley et al., 2015). The BOC is putatively involved in mating mechanisms, as evidenced by the identification of two haplotypes in several *Mamiellophyceae*, and potential recombination suppression, but experimental evidence for sexual reproduction in *Mamiellophyceae* is still lacking (Blanc‐Mathieu et al., 2017). The SOC is hypervariable between strains and has been shown to be associated with viral resistance mechanisms (Blanc‐Mathieu et al., 2017; Yau et al., 2016). While *O. tauri* offers valuable insight into the origin and evolution of biological processes in the green lineage, its low abundance in marine environments, as inferred from metagenomic dataset, hinders its broader use in investigating questions relevant to ecology and adaptation (Demir‐Hilton et al., 2011).

In addition to *Ostreococcus*, the *Bathycoccaceae* family also includes the *Bathycoccus* genus, a picoalga characterized by its scales‐covered cell (Eikrem & Throndsen, 1990). Mostly studied through metabarcoding and metagenomic approaches, there are four *Bathycoccus* species, initially defined as a single species *Bathycoccus prasinos*, but later split into distinct evolutionary clades (B1, B2, B3, and B4) (Bachy et al., 2021; Moreau et al., 2012; Xu et al., 2024). *Bathycoccus prasinos* (B1) *per se* shows high abundance at high latitudes between temperate and polar regions, as opposed to *Bathycoccus calidus* (B2), mostly found in warm oligotrophic waters at lower latitudes (De Vargas et al., 2015; Leconte et al., 2020; Vannier et al., 2016). For the recently characterized B3 and B4 clades, B3 is particularly abundant in the China Sea, while B4 occurs primarily in the Baltic Sea. Notably, a representative strain of the B3 clade was isolated from the Bay of Hong Kong and sequenced, providing additional genomic resources for this lineage (Xu et al., 2024).

Among *Bathycoccus* species, *B. prasinos* displays the widest geographic distribution along latitudinal gradients and exhibits strong seasonal patterns in both temperate and arctic waters, with population growth often occurring through annual bloom events (Devic et al., 2024; Joli et al., 2017; Lambert et al., 2019). The cosmopolitan distribution of *B. prasinos* from polar environments, marked by dramatic changes in photoperiod and temperature, to temperate Mediterranean climate makes this phytoplankton a prime model for the study of both latitudinal and seasonal adaptation mechanisms. The reference genome of *B. prasinos* is 15 Mb in size and is composed of 19 nuclear chromosomes supporting 7847 annotated genes (Moreau et al., 2012). It features, as for other *Mamiellophyceae*, a BOC and a SOC, characterized by atypical genomic features on a large sub‐region of chromosome 14 and on the whole length of chromosome 19, respectively. Additional genomic resources comprise single‐cell assembled genomes of both *Bathycoccus* species and several environmental MAGs, but the variable completion rate of genomes produced by these methods currently provides a fragmented and incomplete view of the genetic diversity within the *Bathycoccus* genus (Benites et al., 2019; Delmont et al., 2022; Joli et al., 2017; Vannier et al., 2016; Vaulot et al., 2012).

In this study, we aimed to draw a comprehensive landscape of *Bathycoccus* genetic diversity at the intraspecific (i.e., within *B. prasinos*) levels and to provide a biological and genomic resource for future studies of molecular mechanisms underlying adaptation to environmental niches in *B. prasinos*. For this purpose we selected and sequenced a panel of *Bathycoccus* strains including (i) collection strains previously isolated in different geographic locations in north western Europe, (ii) strains that have been isolated during the winter bloom of 2018–2019 in the Banyuls Bay (Mediterranean sea, France) (Devic et al., 2024) and (iii) Arctic strains from the Baffin Bay (Arctic Ocean) we isolated and genotyped. Both ONT long reads and illumina short reads were used to generate 28 *de novo* genome assemblies of high quality and completeness, including 24 genomes of *B. prasinos*, one genome of *B. calidus* and 3 genomes of the recently described *Bathycoccus* B3 clade. This resource was used to describe the genetic diversity of *Bathycoccus* at both species and intraspecific levels with a focus on outlier chromosomes and their putative functions.

## RESULTS

### Biological resource

The biological resource used in this study includes 256 strains of *Bathycoccus* sp. from different geographic locations including the Mediterranean Sea, English Channel, North Sea, Arctic Ocean, and Indian Ocean. These comprise seven strains of *B. prasinos* selected from the Roscoff Culture Collection (RCC) (RCC5417, RCC1613, RCC685, RCC1615, RCC1868, RCC4222, RCC4752) and initially sequenced using ONT sequencing to identify polymorphic indel markers (Devic et al., 2024). These markers were used to characterize the genetic diversity of 66 *Bathycoccus* sp. strains isolated in the Bay of Banyuls‐sur‐mer (Mediterranean Sea, France) during the 2018/2019 winter bloom, allowing the selection of seven representative *B. prasinos* isolates for sequencing (G11, C2, G2, E2, A8, B8, A1) (Devic et al., 2024). Similarly, seawater and ice samplings were conducted in three stations of the Baffin Bay as part of the DarkEdge cruise in October 2021. After incubation of microbial communities (sizes <1.2 μm) at 4°C or 15°C, picophytoplankton clones were obtained in semi‐solid low‐melting agarose. Genotyping using *B. prasinos*‐specific primers of the TOC1 gene revealed that all 183 isolates corresponded to *B. prasinos*. Further genotyping using two polymorphic indel markers led to the identification of 10 multi‐loci genotypes representative of the *B. prasinos* diversity in the Baffin Bay. One isolate of each genotype was retained for sequencing (A818, B218, B518, C218, E318, H718, D119, H44, A727, A827) (Table S1).

In total, we selected 24 strains of *B. prasinos* for sequencing, with origins covering a latitudinal gradient from 40° N to 78° N (Table 1). The only strain of *B. calidus* (RCC716) isolated from the Indian Ocean (Bachy et al., 2021) was also included. In addition, 11 isolates from the Banyuls Bay showed weak amplifications of the LOV histidine‐kinase marker. Sequencing of the 18S rDNA confirmed their identity as *Bathycoccus* sp., but variations in the ITS2 region revealed that they were phylogenetically related to the recently described *Bathycoccus* B3 clade, that is distinct from *B. prasinos* (B1), *B. calidus* (B2), and from *Bathycoccus* clade B4 (Xu et al., 2024) corresponding to ITS2 environmental sequences recovered from the Kara Sea (Belevich et al., 2021) (Figure S1). Three of these B3 isolates were thus selected for sequencing (C3, G5, and G8).

**Table 1:** Metadata of sequenced strains

	Species	Latitude (North)	Longitude (East)	Depth (m)	Sampling site	Sampling date	Sample source
Arctic Ocean							
A818	*B. prasinos*	78.12	−74.36	0	Baffin Bay DE310^a^	16‐10‐2021	DarkEdge
B218	*B. prasinos*	78.12	−74.36	0	Baffin Bay DE310^a^	16‐10‐2021	DarkEdge
B518	*B. prasinos*	78.12	−74.36	0	Baffin Bay DE310^a^	16‐10‐2021	DarkEdge
C218	*B. prasinos*	78.12	−74.36	0	Baffin Bay DE310^a^	16‐10‐2021	DarkEdge
E318	*B. prasinos*	78.12	−74.36	0	Baffin Bay DE310^a^	16‐10‐2021	DarkEdge
H718	*B. prasinos*	78.12	−74.36	0	Baffin Bay DE310^a^	16‐10‐2021	DarkEdge
D119	*B. prasinos*	78.12	−74.36	0	Baffin Bay DE310^a^	16‐10‐2021	DarkEdge
H44	*B. prasinos*	76.03	−77.23	0	Baffin Bay DE110	12‐10‐2021	DarkEdge
A727	*B. prasinos*	75.95	−85.58	20	Baffin Bay DE410	21‐10‐2021	DarkEdge
A827	*B. prasinos*	75.95	−85.58	20	Baffin Bay DE410	21‐10‐2021	DarkEdge
RCC5417	*B. prasinos*	67.48	−63.78	0	Baffin Island	01‐06‐2016	RCC^b^
Atlantic Ocean							
RCC1613	*B. prasinos*	57.57	8.67	35	North Sea	26‐07‐2007	RCC^b^
RCC685	*B. prasinos*	54.18	7.90	0	North Sea	16‐05‐2001	RCC^b^
RCC1615	*B. prasinos*	50.2	0.32	10	English Channel	08‐07‐2007	RCC^b^
RCC1868	*B. prasinos*	48.75	−3.95	0	English Channel	05‐02‐2009	RCC^b^
Mediterranean Sea							
RCC4222	*B. prasinos*	42.48	3.53	3	Banyuls Bay	01‐01‐2006	RCC^b^
G11	*B. prasinos*	42.48	3.53	3	Banyuls Bay	03‐12‐2018	OOB^c^
C2	*B. prasinos*	42.48	3.53	3	Banyuls Bay	28‐01‐2019	OOB^c^
G2	*B. prasinos*	42.48	3.53	3	Banyuls Bay	11‐02‐2019	OOB^c^
E2	*B. prasinos*	42.48	3.53	3	Banyuls Bay	25‐02‐2019	OOB^c^
A8	*B. prasinos*	42.48	3.53	3	Banyuls Bay	26‐02‐2019	OOB^c^
B8	*B. prasinos*	42.48	3.53	3	Banyuls Bay	26‐02‐2019	OOB^c^
A1	*B. prasinos*	42.48	3.53	3	Banyuls Bay	19‐03‐2019	OOB^c^
G8	*Bathycoccus* sp.	42.48	3.53	3	Banyuls Bay	03‐12‐2018	OOB^c^
C3	*Bathycoccus* sp.	42.48	3.53	3	Banyuls Bay	12‐12‐2018	OOB^c^
G5	*Bathycoccus* sp.	42.48	3.53	3	Banyuls Bay	12‐12‐2018	OOB^c^
RCC4752	*B. prasinos*	40.48	14.14	100	Gulf of Naples	17‐04‐1986	RCC^b^
Indian Ocean							
RCC716	*B. calidus*	−14.48	113.45	70	Indian Ocean	11‐06‐2003	RCC^b^

^a^ Ice station.

^b^ Roscoff Culture Collection.

^c^ Oceanological Observatory of Banyuls‐sur‐Mer.

### Genomic resources

**Figure 1:**
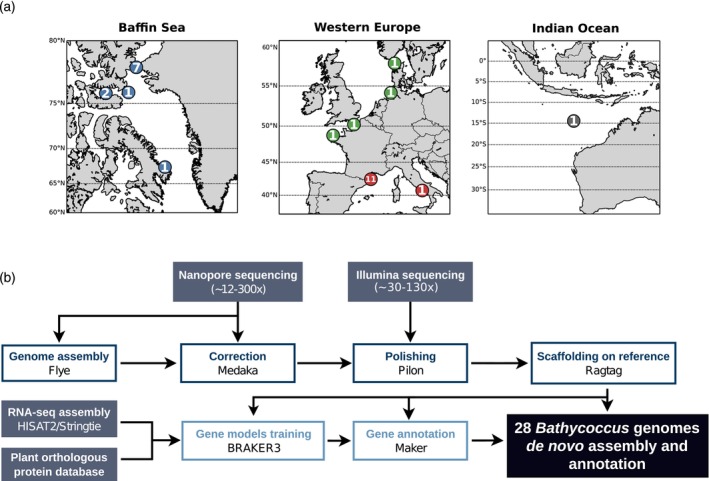
Sampling, sequencing, genome assembly, and annotation strategy of *Bathycoccus*. (a) Geographical distribution of the sampling sites for the selected *Bathycoccus* strains. The number of strains sampled at each site is indicated. Colors correspond to the oceanic basin of origin: blue, Arctic Ocean; green, Atlantic Ocean; red, Mediterranean Sea; gray, Indian Ocean. (b) Summary of the sequencing, assembly, and annotation strategy.

The 28 *Bathycoccus* sp. strains, corresponding to 24 *B. prasinos*, 1 *B. calidus*, and 3 *Bathycoccus* B3 clade, display a wide geographical distribution ranging from Arctic to Equatorial regions and seasonal patterns in the Banyuls Bay (Devic et al., 2024) (Figure 1a; Table 1). These strains were sequenced using both ONT and Illumina to produce long and short high‐quality reads respectively, ensuring contiguity and low error rates in the final assemblies. Genome assembly was conducted using a pipeline outlined in Figure 1b. ONT reads of approximative depth ranging from 12 to 300×, assuming a genome size of 15 Mb (Moreau et al., 2012), were used for a genome assembly using FLYE and a post‐assembly correction using Medaka. Illumina reads (30–130× depth) were used for final assembly polishing using Pilon. Scaffolding and orientation of the created contigs upon the reference genome from the strain RCC1105 (Moreau et al., 2012) were performed through RagTag.

**Figure 2:**
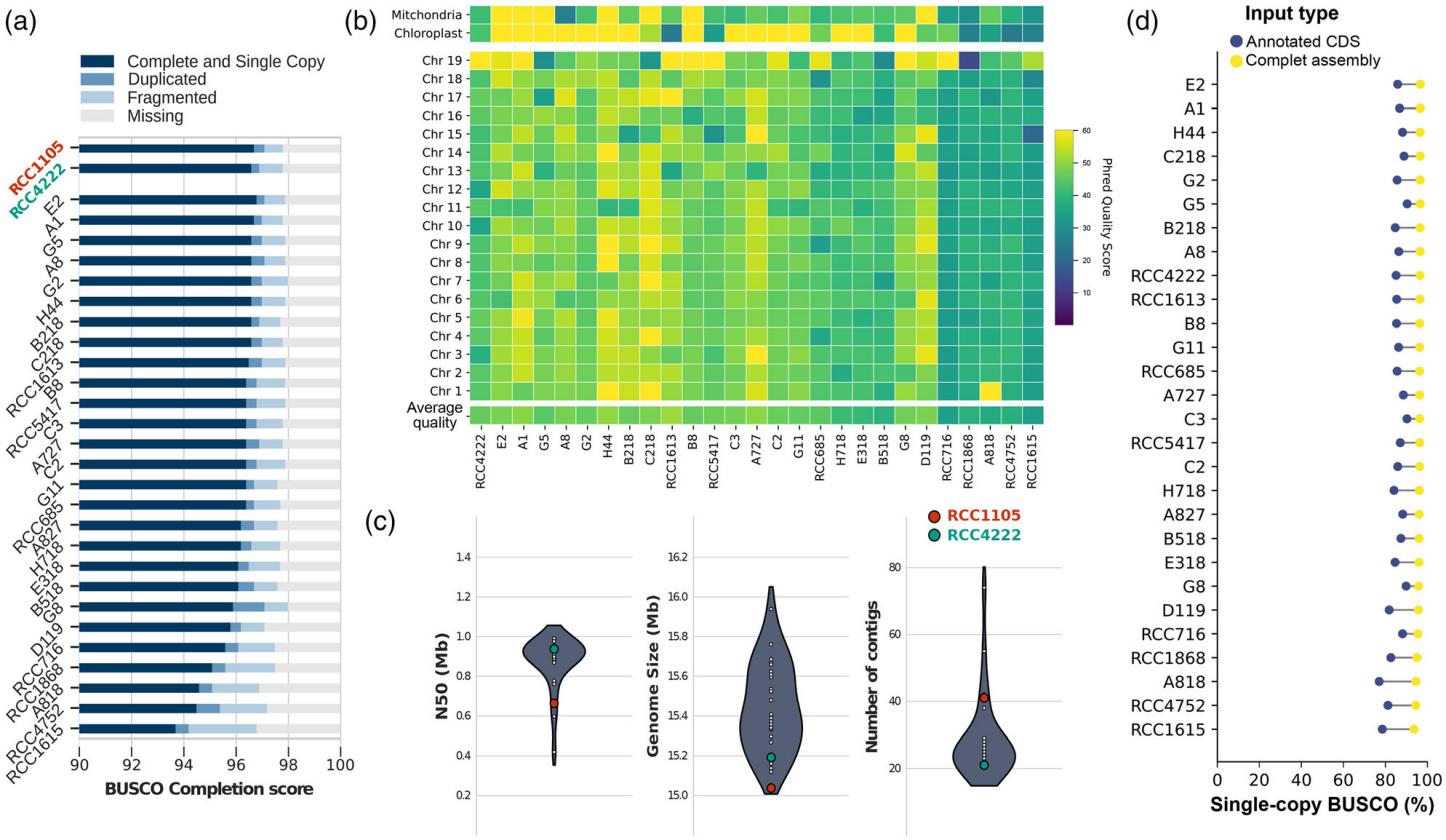
Genome assemblies statistics and quality assessment. (a) BUSCO completion score of genome assemblies for the 28 *de novo* assembled *Bathycoccus* genomes using the *chlorophyta_odb10* library. RCC1105 corresponds to the reference genome produced by Moreau et al. (2012), while RCC4222 corresponds to the *de novo* assembly obtained from a clonal strain of RCC1105. (b) PHRED quality score of genome assemblies per chromosome. (c) Distribution of principal assembly statistics. The N50 corresponds to the sequence length of the shortest contig at 50% of the total assembly length. (d) Comparison of BUSCO single‐copy completion score using complete genome sequence or annotated CDS sequences as input.

*De novo* assembly of the 28 *Bathycoccus* sp. strains resulted in assembly statistics that were either superior to or on par with the reference genome (Data S1). These assemblies have a BUSCO completion score between 94.10 and 97.10%, with a median value of 96.80% and showed an average QPHRED score of 44.28 ± 4.96 (Figure 2a,b). These high‐quality assemblies also displayed an average of 24 contigs per assembly, with a mean N50 of 920 kb, including six assemblies without any identified gap (21 contigs) highlighting their high continuity (Figure 2c).

RCC4222, a clonal strain of the *B. prasinos* reference strain RCC1105 isolated in 2006 (Moreau et al., 2012), was sequenced in 2018 to check clonal conservation of the genome structures and to test the assembly pipeline. No major structural variations were identified compared to the original reference (Figure S2). In particular, chromosome 19, that is, the SOC described in *Bathycoccaceae* as a hypervariable structure with a high intraspecific diversity, was fully conserved between RCC4222 and RCC1105 (Blanc‐Mathieu et al., 2017; Moreau et al., 2012). This indicates that the *B. prasinos* genome is stable in culture and validates our pipeline. Overall, reassembly of the reference using long reads improved continuity, as evidenced by the higher N50 (from 663 to 937 kb), higher genome size (from 15.04 to 15.19 Mb), and a reduction of contig number from 41 to 21, corresponding to the 19 nuclear chromosomes in addition to the chloroplastic and mitochondrial genomes (Figure 2c). The BUSCO completion scores remained comparable between assemblies, confirming furthermore the higher completion of the resequenced reference genome (Figure 2a).

A non‐redundant repeat library, generated from all *Bathycoccus* sp. genomes with RepeatModeler and CD‐hit, was used for repeat annotation via RepeatMasker, resulting in the annotation of ~10.36 ± 0.82% of each genome as repeated sequences (Figure S3; Table S2). However, only a few of them could be associated with known structures, including LINEs, LTR elements, and transposons, while the majority of classified sequences corresponded to simple repeats (~3.22 ± 0.18% of sequences) (Table S2).

A structural annotation of coding sequences was performed for each genome using available RNAseq data from *B. prasinos* strain RCC4752 and plant orthologous protein data to train three gene prediction models through the BRAKER3 and Maker pipelines (GENEMARK‐ETP, Augustus, and SNAP). The output of each model was then integrated using the Maker annotation pipeline into a complete structural annotation (Figure 1b; Figure S2). The structural annotation predicted 7431 (±236) genes, with 7243 annotated in the strain RCC4222 while 7847 coding genes were initially annotated in the reference (Moreau et al., 2012). This discrepancy may be due to a lower ratio of mono‐exonic genes (73% of predicted transcripts) in our annotation, resulting from the integration of genome assembled transcripts from RNAseq data (Table S3). For quality control of the produced annotation, single‐copy BUSCO completion scores were performed either for complete assembly genome sequences or for protein sequences extracted from annotated CDS (coding DNA sequences). Complete assembly sequences produced an average BUSCO completion score of 96.10 ± 0.75%, and proteins from annotated CDS produced an average score of 85.72 ± 3.24% (Figure 2d), confirming the quality of the structural annotation.

### Phylogenomic of the *Bathycoccus* genus

**Figure 3:**
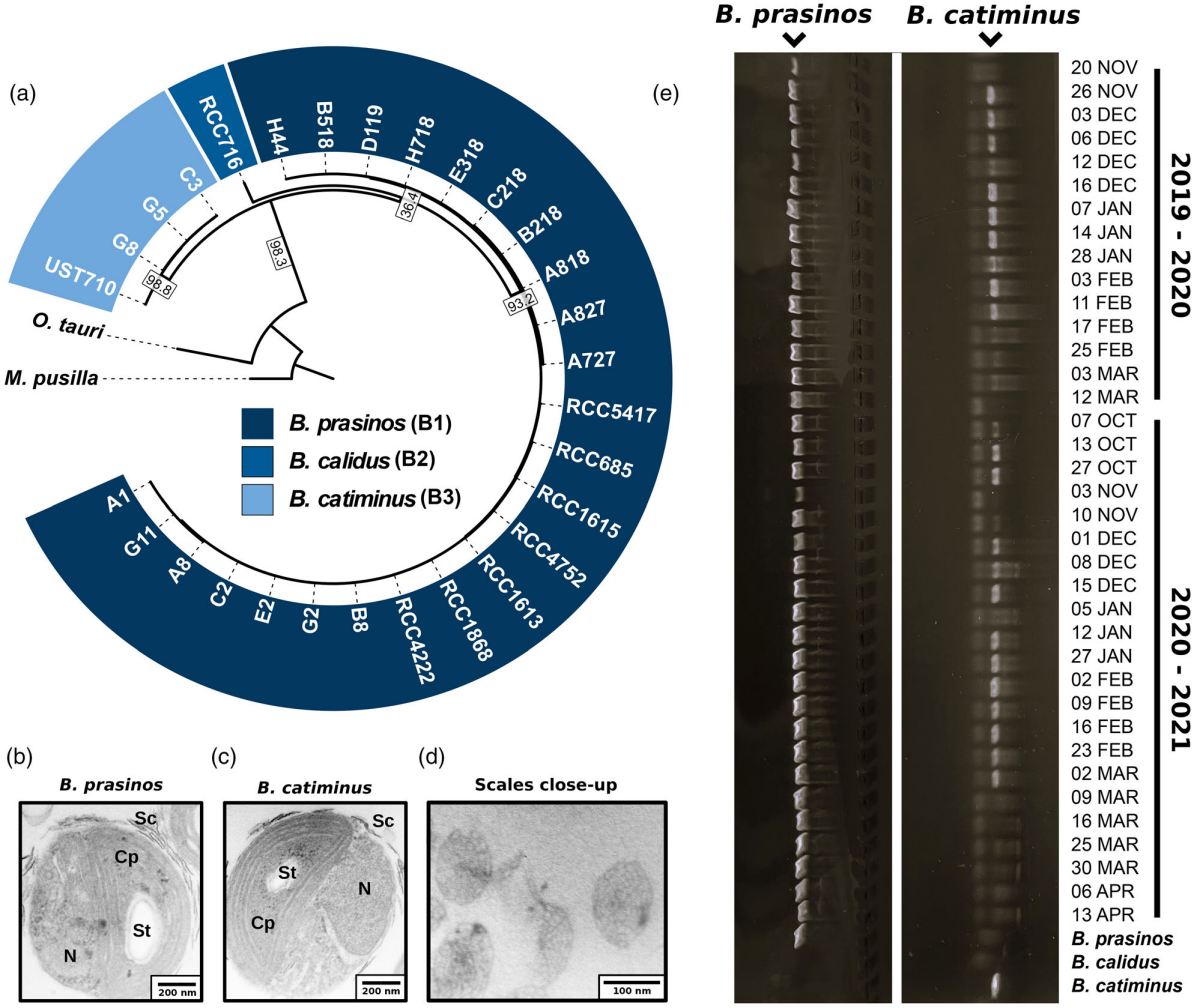
Phylogenomic of the *Bathycoccus* genus and *Bathycoccus catiminus* characterization. (a) Maximum‐likelihood phylogenetic tree of *Bathycoccus* strains based on 1200 shared genes. Species separation is derived from the branch length and marked by colors. The tree includes strain UST710 isolated and sequenced by Xu et al. (2024) for reference. *Ostreococcus tauri* and *Micromonas pusilla* are used as outgroups. Only branches with bootstrap values higher than 90% are displayed. Gene concordance factors are displayed for all visible branches. Transmission electron microscopy images of (b) *Bathycoccus prasinos*, (c) *B. catiminus* strain isolated from the Banyuls Bay, and (d) a close up of detached spider web scales from *B. catiminus*. Cp, chloroplast; N, nucleus; Sc, scales; St, starch granule. Bar = 100 nm. (e) Presence of *B. catiminus* in Banyuls Bay seawater samples from 2019–2020 and 2020–2021 phytoplanktons blooms, identified using specific primers designed from non‐conserved sequences of TOC1 ORF specific to *B. catiminus*.

Using this high‐quality dataset, we investigated the phylogenomics of the *Bathycoccus* genus. Maximum‐likelihood phylogenetic distances were inferred from nucleic acid alignments of genes shared between all strains. To avoid potential annotation biases between species caused by the predominance of *B. prasinos* transcriptomic data in the annotation model training, a set of 1200 shared BUSCO genes coming from the *chlorophyta_odb10* database was used. The inferred phylogenetic tree showed a clear separation between strains of *B. prasinos*, *B. calidus*, and the three putative *Bathycoccus* B3 genome sequences from Banyuls Bay. As expected, these putative *Bathycoccus* B3 grouped with the B3 strain UST710 isolated in Hong Kong by Xu et al. (2024), showing an early divergence in the phylogenetic tree (Figure 3a). Cell ultrastructure determined by electronic microscopy revealed characteristic features of the *Bathycoccus* genus with a single mitochondrion, a single chloroplast containing a single starch granule, and scales at the cell surface that have been described earlier for *B. prasinos* (Moreau et al., 2012), *B. calidus* (Bachy et al., 2021), and *Bathycoccus* B3 strains from Hong Kong (Xu et al., 2024) (Figure 3b–d).

The mapping of metagenomic data from TARA Ocean and TARA Polar Circle cruises was performed on a representative genome for each species. This analysis confirms the clear latitudinal and depth separations between *B. prasinos* and *B. calidus* populations, initially described by Vannier et al. (2016), with *B. prasinos* found in temperate regions at the surface, and *B. calidus* in tropical water at the deep chlorophyll maximum. *Bathycoccus prasinos* was also abundant (up to 4% of sequences) in Arctic waters. *Bathycoccus* B3 was also detected in metagenomic data and shows geographical distribution patterns similar to *B. prasinos*; however, at much lower levels of abundance than *B. prasinos* and *B. calidus* (up to 0.5% of sequences). However, the comparison of mapped metagenomic reads indicates a high level of cross‐mapping between *B. prasinos* and *Bathycoccus* B3, with only ~9% of reads being specific to *Bathycoccus* B3 in all stations. This results in approximately 0.5% of horizontal coverage distributed along all chromosomes of *Bathycoccus* B3 and suggests that its abundance is ~10 times lower than initially computed (Figure S4). Based on the current evidence of the phylogenetic and spatial–temporal distinctions of the Bathycoccus B3 clade strains, we propose to name this cryptic species as *Bathycoccus catiminus* (from the French expression ‘en catimini’ meaning ‘on the sly’) in reference to its unexpected discovery and its very low abundance in metagenomic datasets compared to *B. prasinos* and *B. calidus* species (Appendix S1).

The presence of *B. catiminus* was also experimentally investigated through PCR in water samples from the Banyuls Bay between January and March 2019, using species‐specific primers designed from sequences of the *B. catiminu*s TOC1 ORF. *Bathycoccus catiminus* was detected in Banyuls Bay water between November 2019 and February 2020, and between October 2020 and March 2021 (Figure 3e), in addition to its presence in December 2018 when the *B. catiminus* strains were isolated.

### Comparative quality assessment of *B. prasinos* MAGs and *de novo* genome assemblies from isolated strains

**Figure 4:**
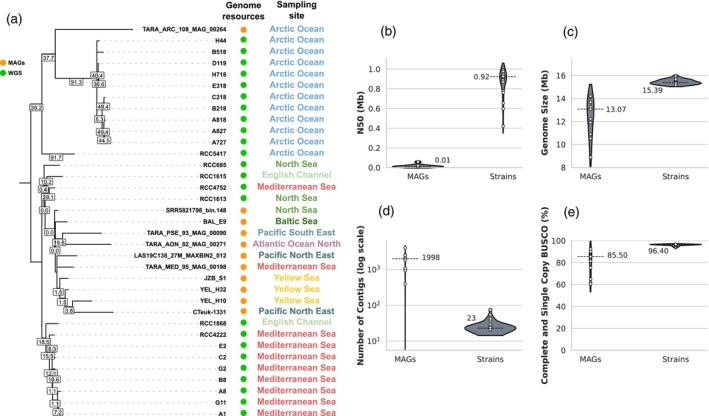
Qualitative comparison between *de novo* genome assembly from whole‐genome sequencing data (WGS) and metagenome‐assembled genomes (MAGs) of *Bathycoccus prasinos*. (a) Maximum‐likelihood phylogenetic tree of *B. prasinos* available genomes based on 265 shared genes. Only branches with bootstrap values higher than 90% are displayed. Gene concordance factors are displayed for all visible branches. Green dots represent WGS, while orange dots represent MAGs. (b–e) Distribution of principal assembly statistics between available MAGs (*n* = 11) and whole‐genome assemblies produced from isolated strains of *B. prasinos* (*n* = 24). Median values are displayed for reference.

The *B. prasinos* genomic resource was expanded by adding 11 MAGs available in the literature, as reported by Xu et al. (2024), to our 24 *de novo* assembled genomes to investigate the species' intraspecific phylogenetic diversity. Based on 265 BUSCO genes conserved between the 35 samples, this analysis revealed an apparent disconnection between these datasets, as illustrated by extreme phylogenetic distances among MAGs, and between MAGs and *de novo* assembled genomes (Figure 4a). MAGs appeared to cluster together rather than with strains isolated from the same geographical basins, with the exception of TARA_ARC_108_MAG_00264 which presented a weak clustering with other Arctic strains. Moreover, comparison of assembly metrics highlighted drastically lower values for MAGs with a wide distribution compared to *de novo* assembled genomes. For instance, the median N50 values were 0.01 Mb for MAGs compared to 0.92 Mb for assembled genomes (Figure 4b). Similarly, the median genome size was approximately 15% smaller in MAGs (13.07 Mb) compared to *de novo* assembled genomes (15.39 Mb) (Figure 4c), while the median number of contigs reached up to 1998 in MAGs for only 23 in *de novo* assembled genomes (Figure 4d). Finally, the median BUSCO completion score in MAGs was 85.5% compared to 96.4% in *de novo* assembled genomes (Figure 4e). Collectively, these differences highlight the large variations in continuity, contiguity, and completeness among MAGs and compared to *de novo* assembled genomes, limiting their use in high‐resolution intraspecific diversity characterization including in phylogenetic analysis.

### Intraspecific diversity and chromosomal haplotypes in *Bathycoccus* strain genomes

**Figure 5:**
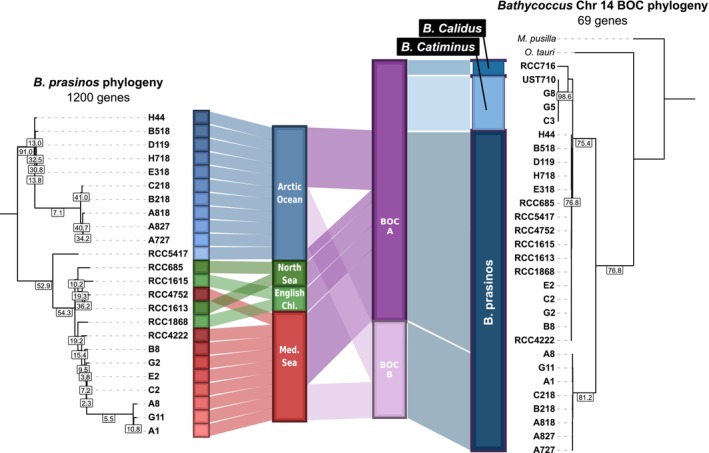
Phylogenomic of *Bathycoccus prasinos* linked to strain origin and Chromosome 14 big outlier chromosome (BOC) haplotypes. (Left) Maximum‐likelihood phylogenetic tree of *B. prasinos* strains based on 1200 shared genes. (Right) Maximum‐likelihood phylogenetic tree of *Bathycoccus* strains based on 69 shared genes located on chromosome 14. Only branches with bootstrap values higher than 90% are displayed. Gene concordance factors are displayed for all visible branches.

For an accurate assessment of *B. prasinos* intraspecific diversity, the phylogenetic diversity of the *B. prasinos* species was inferred focusing on the 24 corresponding *de novo* sequenced genomes and using 1200 conserved genes. The resulting phylogenetic tree revealed two main branches, separating the strains isolated from the North of Baffin Bay during the DarkEdge cruise in October 2021 from all other strains, including the arctic strain RCC5417 isolated from the south of Baffin Bay in 2016. However, this last strain diverges early from the cluster of temperate strains. Similarly, Mediterranean strains from the Banyuls Bay isolated in 2018 and 2019 were more closely related to each other than to the RCC4222 strain isolated from the Bay of Banyuls in 2006. Comparably, the strain RCC4752 isolated in 1986 in the Gulf of Naples did not cluster together with other Mediterranean strains. Geographical origin of strains from the English Channel and the North Sea were not resolved in our phylogenetic tree (Figure 5). Within geographic basins, subgroups were observed including three strains in the Banyuls Bay (A1, G11, and A8) and five strains in the Baffin Bay (C218, B218, A818, A827, A727). A second phylogenetic analysis was performed based on 69 shared genes located on the entire chromosome 14, the BOC of *B. prasinos*. This analysis revealed that strains within the aforementioned subgroups clustered together, independently of their geographic origin. Furthermore, using the same approach, the sister species *B. calidus* and *B. catiminus* clustered together with *B. prasinos* regardless of their taxonomic classification, revealing that the two BOC haplotypes are shared within the *Bathycoccus* genus. These were designated as BOC A and BOC B (Figure 5).

### Genomic diversity and geographic distribution of the BOC

**Figure 6:**
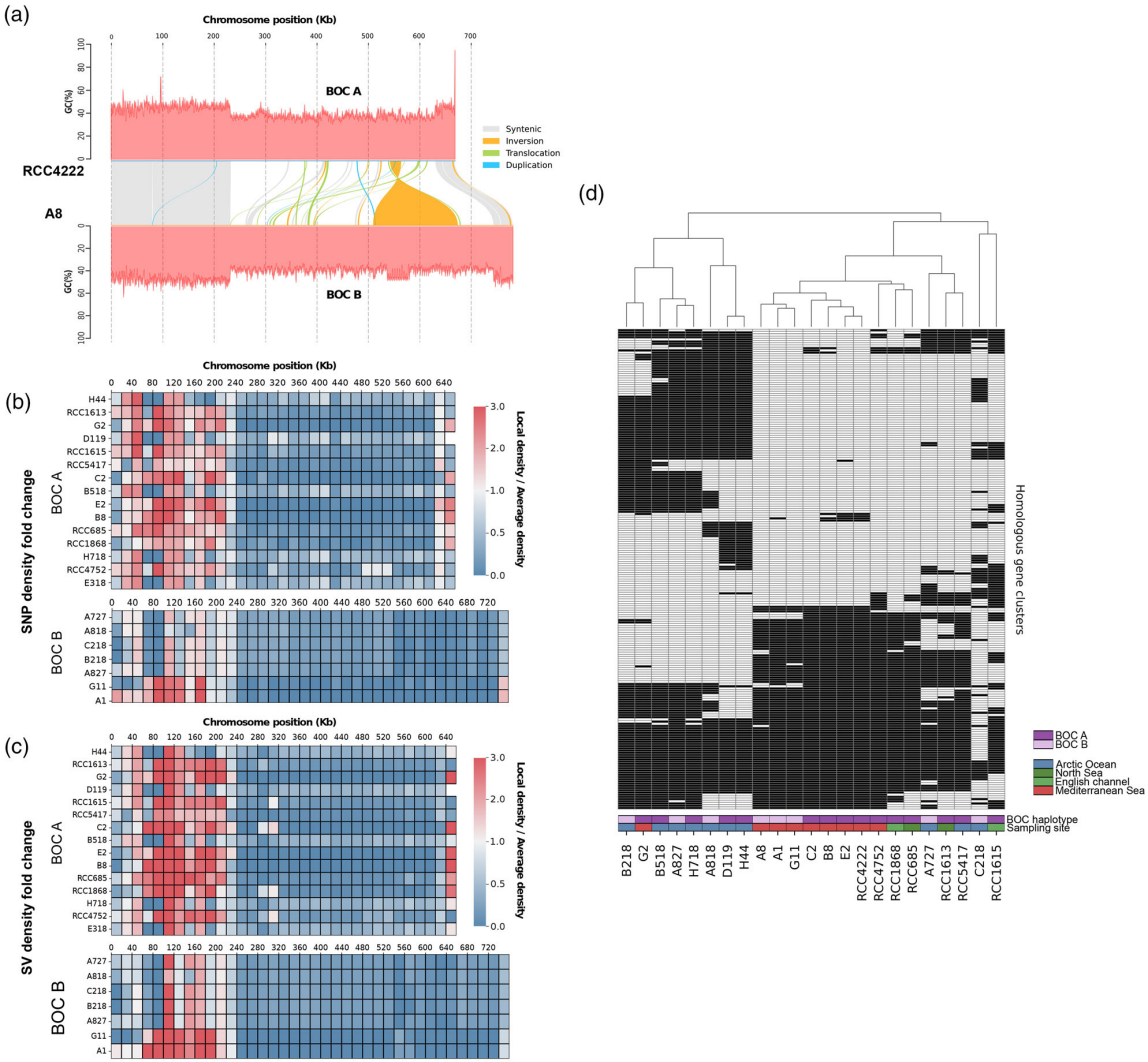
Genomic diversity and gene content distribution of *Bathycoccus prasinos* big outlier chromosome (BOC). (a) Alignment of Chromosome 14 from *B. prasinos* strains RCC4222 and A8. Colors indicate syntenic regions and structural rearrangements. *Y* axis displays the GC content of the corresponding sequences. (b) Divergences between local single nucleotide polymorphism (SNP) density and genome‐wide SNP density along a 20 kb sliding window. (c) Divergences between local structural variation (SV) density and genome‐wide SV density along a 20 kb sliding window. SNP and SV calling was computed for each BOC haplotype against strain RCC4222 for BOC A and strain A8 for BOC B. (d) Distribution of homologous gene clusters identified from the BOC outlier region between all strains (*N* = 256). Strain order follows hierarchical clustering of gene clusters presence/absence. Gene clusters are colored in black if present and white if absent.

The genomic diversity between BOC haplotypes of chromosome 14 was investigated by alignment of chromosome sequences between strains. The identification of syntenic regions confirms that the haplotype‐specific sequences are restricted to the outlier region, featured by a lower GC content. This chromosome segment corresponds to a ~400 (BOC A) to 500 kb (BOC B) non‐syntenic region between the two haplotypes (i.e., ~2/3 of the chromosome length), with major inversion and duplication events, as well as other smaller structural rearrangements (Figure 6). Since several strains were sequenced for each BOC haplotype (16 for BOC A, 8 for BOC B), single nucleotide polymorphism as well as structural variation density along chromosome 14 could be determined. Polymorphism density was computed on a 20 kb sliding window along chromosome 14, and compared to the genome‐wide polymorphism to detect local divergences in sequence diversity. Lower than average polymorphism (local density/average density <1) was detected in the outlier region of both identified haplotypes and, overall, lower polymorphism density of this region could be seen compared to the common regions located at both extremities of chromosome 14 (Figure 6b,c; Figure S5). This clear pattern of localized reduced polymorphism could not be seen on any other non‐outlier chromosomes (Figure S6). Additionally, gene homology was computed within the respective BOC outlier region of all strains (Figure 6d; Figure S7). This led to the identification of 256 homologous gene clusters unevenly distributed among strains of distinct BOC haplotypes and/or geographical origin. Hierarchical clustering of homologous gene distribution highlighted a segregation of strains independently of BOC haplotype but partially based on geographical origin for strains isolated in the Arctic Ocean and the Mediterranean Sea (Figure 6d), while being non‐syntenic between the two BOC haplotypes. Moreover, few gene clusters were identified as core or associated to a specific BOC haplotype, rather demonstrating large variation of presence/absence and an even distribution between BOC haplotypes (Figure S7). This gene diversity is associated with reduced coding density in the outlier region (BOC A: 46.0 ± 2.1%; BOC B: 48.1 ± 3.4%) relative to the whole‐genome average (78.7 ± 2.5%). Finally, Gene Ontology analysis of these gene clusters showed limited enrichment with biological processes such as translation, DNA replication, and nucleotide‐excision repair (Table S4).

**Figure 7:**
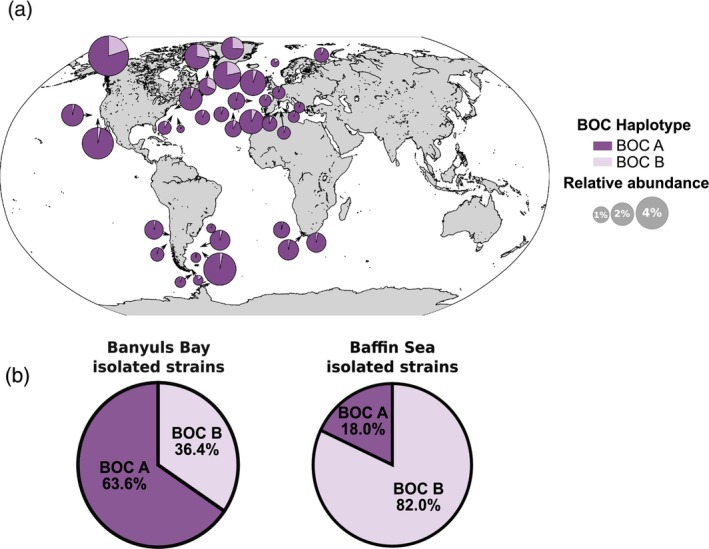
Characterization of big outlier chromosome (BOC) haplotype geographical distribution. (a) BOC haplotypes ratio based on read mapping from TARA ocean metagenomic datasets in stations with *Bathycoccus prasinos* relative abundance >0.1%. (b) BOC haplotype ratio among strains isolated in the Banyuls Bay (Left) (*n* = 55) and the Baffin Sea (Right) (*n* = 183).

The geographical distribution of both BOC haplotypes in the world Ocean was assessed by mapping of TARA Ocean and TARA polar circle metagenomic dataset on their respective outlier sequences. In all stations defined by relatively abundant *B. prasinos* sequences, the BOC A haplotype, corresponding to the haplotype of the reference strain RCC4222, was predominant, with a mapping ratio ranging from 68 to 97% of all mapped reads. This imbalance seemed less strict in polar circle stations, with the BOC B haplotype representing up to 32% of mapped reads (Figure 7a). This difference in BOC ratios between temperate and arctic regions was further amplified in the haplotype ratio of isolated strains, with BOC A corresponding to the majority in strains isolated from the Banyuls Bay (63.6% of strains), while BOC B was predominant in the Baffin Bay (82% of strains) (Figure 7b).

### Genomic diversity of the SOC

**Figure 8:**
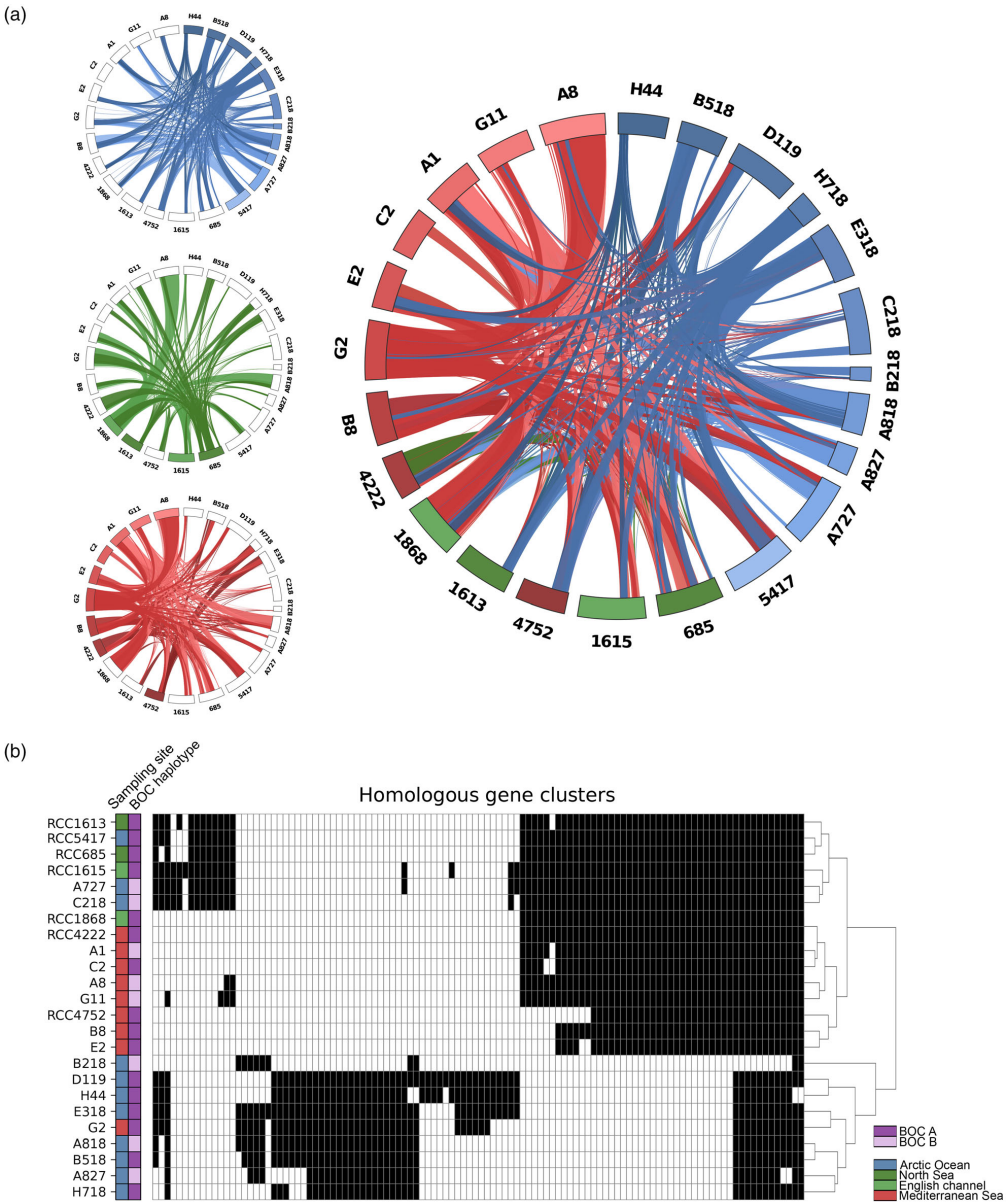
Chromosome 19 small outlier chromosome (SOC) shared sequences between *Bathycoccus prasinos* strains. (a) Chord plot representation of chromosome 19 shared sequences between strains of geographically close water basins (Left) and between all 24 *B. prasinos* sequenced strains (Right). Chromosome color corresponds to basin and geographical distribution of strains as shown in Figure 5: blue, Arctic Ocean; green, North Sea and English Channel; red, Mediterranean Sea. Only shared sequences longer than 500 bp are displayed. (b) Distribution of homologous gene clusters identified from chromosome 19 between all strains (*N* = 110). Strain order follows hierarchical clustering of gene clusters presence/absence. Gene clusters are colored in black if present and white if absent.

Another characteristic feature of *Mamiellophyceae* genomes is the SOC, corresponding to chromosome 19 in *Bathycoccus* species. The SOC chromosome size varied considerably among the 24 assemblies of *B. prasinos*, particularly in arctic strains, with sizes ranging from 48 to 230 kb. With the exception of RCC1868 from the English Channel, for which major conserved and syntenic regions were identified with strains A8, G2, and A818 (up to 190 kb conserved with A8), little or no synteny was detected between SOCs (Figure 8a; Figure S8). This held true even between strains originating from the same geographic basin or isolated at the same time in both Banyuls and Baffin bays. Surprisingly, despite this low synteny, conserved sequences with a wide range of sizes (Average size: 15 787 ± 22 706 bp) were identified, indicating extensive reorganization within each genome (Figure 8a).

Higher chromosome 19 sequence conservation was observed between strains isolated from the same geographic region, at the same time, such as for the Baffin Bay in 2021, or the Banyuls Bay in 2018–2019, up to the point that several assemblies had their chromosome almost fully composed of shared sequences (Figure 8a). However, chromosome 19 of Mediterranean strains isolated earlier (such as RCC4752 isolated in 1986 in the Gulf of Naples, or RCC4222 isolated in 2006 in the Banyuls Bay) had fewer conserved sequences with chromosome 19 of other Mediterranean strains than with strains from other geographical regions. Apart from the strain C2, which showed no chromosome 19 conservation with arctic assemblies, all genomes had at least one conserved region with another strain (Figure 8a). Remarkably, all sequences of the shortest chromosomes 19 (H718, B218, and A827 strains) were detected in other strains. These conserved sequences were usually concentrated in a distal region of chromosome 19, as seen in strains from the Banyuls Bay and the Baffin Bay (Figure S8).

Through homology clustering, 110 gene clusters were identified within chromosome 19 (Figure 8b). Hierarchical clustering of their distribution highlighted a separation of strains in three groups respectively composed of Arctic strains, Mediterranean strains, and a mix of strains originating from the North Sea, the English Channel and the Arctic Ocean. No association with the strains BOC haplotype was observable. Nineteen genes were identified as core (presence >90%) while only seven were present in less than 10% of strains, in accordance with important sequence conservation and reorganization. Gene Ontology analysis highlighted enrichment in several molecular functions such as protein dimerization, hydrolase activity, or DNA helicase activity but was limited by a low count of functionally annotated genes (Table S5).

## DISCUSSION

### Worldwide biological and genomic resources for the study of latitudinal and seasonal adaptation

In this study, we isolated 183 Arctic strains to add to the 66 Mediterranean strains previously isolated from the Banyuls Bay (Devic et al., 2024), and to the available strains of the RCC, further extending the current *Bathycoccus* sp. biological resource. All Arctic and Mediterranean strains were genotyped, and the main haplotypes were fully sequenced using ONT and Illumina sequencing, including 10 strains from the Baffin Bay and 10 strains from the Banyuls Bay. This biological resource, covering a latitudinal gradient from pole to equator, provides an unprecedented tool to study the *Bathycoccus* sp. diversity and the physiological responses underlying adaptation to latitudinal gradients and seasons. Phylogenomic analysis revealed that three strains isolated from the Banyuls Bay corresponded to the recently described *Bathycoccus* B3 clade that we named *B. catiminus* (Figure 3). Unlike *B. prasinos* and *B. calidus*, *B. catiminus* is barely detectable in large metagenomic datasets such as TARA Ocean and TARA Polar Circle, being restricted to a few geographic locations such as the Yellow Sea and the Caspian Sea (Xu et al., 2024). Concurrently, sequencing of ITS2 rDNA regions in the Kara Sea and MAG reconstruction indicates that a fourth species is yet to be isolated (Belevich et al., 2021; Xu et al., 2024) (Figure S1), highlighting the underestimated taxonomic diversity of the *Bathycoccus* genus. As reviewed by Yung et al. (2022), the description of new species following genome sequencing is frequent in *Mamiellophyceae*, due to the lack of polymorphism in cellular ultrastructure and in the sequence of rRNA 18S V4 and V9 regions, commonly used as distinguishing characters (Piganeau et al., 2011).

The exploitation of natural diversity has greatly contributed to the characterization of genomic features and to the exploration of adaptive mechanisms, not only in Arabidopsis but also in other wild land plants and crop cultivars (Alonso‐Blanco et al., 2016; Gabur et al., 2019 for review). However, the intraspecific diversity of eukaryotic phytoplankton remains widely unexplored, with only a few studies conducted such as in *O. tauri* (Blanc‐Mathieu et al., 2017), *P. tricornutum* (Rastogi et al., 2020), and *E. huxleyi* (Bendif et al., 2023; Read et al., 2013). In all these studies, reference‐assisted assembly approaches based on short reads mapping to a single reference genome were used. This allows for high resolution of single nucleotide polymorphisms and small structural variations, but ignores larger structural variations and population‐specific sequences. However, larger modifications also play an important role in environmental adaptation, since they may contain additional genes or regulatory sequences (Gordon et al., 2017). The application of *de novo* assembly methods based on long‐read sequencing in *Bathycoccus* sp. enables the identification of such large structural variations and the resolution of complex genomic structures specific to this taxonomic class (Figures 6, 7, 8). While the current state of algal genomic data is characterized by assembled genomes of heterogeneous quality and completeness, restricting intraspecific comparisons to a few generally incomplete genomes (Hanschen & Starkenburg, 2020), we aimed to produce a resource for the study of intraspecific genetic diversity within the *Bathycoccus* genus. Thus, we report here 24 *de novo* assembled and annotated genomes of the cosmopolitan species *B. prasinos*. To our knowledge, this is the largest genomic resource associated with a biological resource available for a single species of green algae (Hanschen & Starkenburg, 2020; Shi et al., 2021). Thanks to the abundance of *B. prasinos* in large metagenomic datasets, numerous MAGs have been reconstructed, opening the way to the intraspecific comparison of populations adapted to specific niches (Xu et al., 2024). However, phylogenomic comparisons revealed inconsistencies between available MAGs and our *de novo* assembled genomes (Figure 4). MAG reconstruction is not free from biases, since MAGs are assembled from binned reads often originating from geographic basins, rather than from single samples, to compensate for low sequencing depth and horizontal coverage. Therefore, the quality of the assembly is strongly influenced by the sequencing depth and the genetic/genomic heterogeneity of the sequenced population, leading to chimeric, fragmented, and most of the time incomplete assemblies with frequent contamination sequences embedded within (Meziti et al., 2021). For instance, the BOC B haplotype of *B. prasinos* was not identified in MAGs, probably due to BOC B representing only 3–30% of sequences in metagenomic samples (Figure 7). For the same reasons, MAGs seem not well suited to identifying the fine genetic variations associated with environmental parameters, involved in adaptation to specific environmental niches. The biological and genomic resources presented here, and that will be expanded in the future, should make it possible to identify the genetic signatures underlying the adaptation and ecological success of *B. prasinos* through population genetics and genome‐wide association studies similar to those conducted in land plants (Alonso‐Blanco et al., 2016; Beaulieu et al., 2025).

### Low polymorphism within putative sexual chromosome haplotypes indicates strong selective pressure

We used the *B. prasinos* resource consisting of 24 genomes together with the existing large metagenomics dataset to conduct an analysis of outlier chromosomes. The BOC, discovered in *O. tauri*, is a putative sexual chromosome that has been identified in all other *Mamiellophyceae* genomes (Derelle et al., 2006; Grimsley et al., 2015). While two haplotypes of this BOC were identified in *O. tauri*, only one haplotype was previously described for the *Bathycoccus* genus (Blanc‐Mathieu et al., 2017; Moreau et al., 2012). In this study, the two BOC haplotypes were identified in *B. prasinos* strains from both the Banyuls and the Baffin bays, suggesting that these haplotypes are not the result of population segregation due to geographic isolation, but rather a defining feature of the *B. prasinos* genome, as it was previously reported for other *Mamiellophyceae* (Grimsley et al., 2015).

The BOC of *B. prasinos*, corresponding to chromosome 14, has been characterized by several distinct features located in a large distal sub‐region, including lower GC content, a high number of introns, and increased expression levels (Derelle et al., 2006; Moreau et al., 2012). In *O. tauri*, previous reports indicated a higher linkage disequilibrium and reduced recombination events in the outlier region, leading to an accumulation of transposable elements and suggesting a putative mating function of this region (Blanc‐Mathieu et al., 2017), thus following the model of the mating chromosome of *Chlamydomonas reinhardtii* (Ferris & Goodenough, 1994). However, no accumulation of transposable elements was detected in the *B. prasinos* reference genome, and no loss of synteny was identified in strains of the same BOC haplotype (Figure S5) (Moreau et al., 2012). Instead, a lower density of single nucleotide polymorphisms and of structural variations was observed in the outlier region of both *B. prasinos* BOC haplotypes, even between geographically distant strains (Figure 6b,c). Lower than average polymorphism has also been reported in the *O. tauri* outlier region and *C. reinhardtii* mating chromosome (Blanc‐Mathieu et al., 2017; Hasan et al., 2019), in apparent contradiction with recombination suppression, that would intensify mutation accumulation in sexual chromosomes. Gene conversion between mating types was proposed as a sequence homogenizing mechanism by De Hoff et al. (2013), but this hypothesis proved difficult to test with *O. tauri* genomic data, due to the representation of one of the putative mating types by just a single strain. In *B. prasinos*, our analysis of shared genes within chromosome 14 revealed a divergent evolution of core genes between haplotypes (Figure 5). However, this analysis also highlighted important presence/absence variation in accessory genes within the outlier region, along with limited synteny between the two haplotypes and an overall lower coding density (Figure 6). The distribution of the accessory genes was primarily associated with the geographical origin of strains rather than with BOC haplotypes (Figure 6d), suggesting the accumulation of population‐specific genes within this region. These observations point to the coexistence of two evolutionary forces possibly shaping this complex genomic structure. On the one hand, a strong haplotype‐specific selective pressure appears to drive the divergence of core genes, consistent with previous observations in mating chromosomes (Hasan & Ness, 2020; Hasan et al., 2019). On the other hand, the patterns observed in accessory genes are consistent with gene conversion events within the outlier region and between haplotypes, contributing to local sequence homogenization for adaptive purposes. Together, these opposing dynamics may contribute to the maintenance of the atypical structure of the BOC, the increased expression level and the fragmented gene structure within the outlier region. Although the fitness impact of this 400–500 kb haplotype‐specific outlier region still needs to be assessed, it is possible that the regional imbalances observed in metagenomic datasets in *B. prasinos* (Figure 7) and *Ostreococcus lucimarinus* (Leconte et al., 2020) could be attributed to either differential fitness, or seasonal dynamics among BOC haplotypes, or both. The cosmopolitan distribution of *B. prasinos*, along with its abundance in metagenomic datasets and the important biological resource available, offers a unique opportunity to monitor the spatio‐temporal population dynamics of BOC haplotypes and to further study the role of BOCs in an environmental context (Lambert et al., 2019; Vannier et al., 2016).

### The viral immunity chromosome presents hypervariable size and a bipartite chromosome structure in the environment

The SOC is a hypervariable chromosomal structure common to *Mamiellophyceae* genomes, characterized in *O. tauri* by a low synteny, a high sequence diversity between strains due to extensive structural rearrangements, and an important variation in chromosome size (Blanc‐Mathieu et al., 2017; Derelle et al., 2006). In this study, we report SOC conserved and rearranged sequences between 24 *B. prasinos* strains. This genomic resource encompasses broad geographical and temporal scales, revealing significant sequence conservation between strains from separate oceanic basins, despite the overall lack of synteny. While several strains come from the same water samples in the Baffin and Mediterranean Sea, others were isolated at different times over a period spanning from 1986 to 2020, offering the opportunity to study both the diversity and dynamics of SOC structure at different spatial and temporal scales.

Important size variations for the SOC were displayed in our dataset, especially between Arctic strains isolated during the DarkEdge cruise for which we identified the smallest SOCs (Figure 8). Structural and transcriptional modifications of the SOC were shown by Yau et al. (2016, 2018) to be induced by prasinovirus infection and linked to increased spectrum of resistance, suggesting a role of the SOC in viral resistance mechanisms in *Mamiellophyceae*. Moreover, switches between resistant and susceptible phenotypes within isogenic cultures were associated with SOC rearrangements in *O. tauri* and *O. mediterraneus* and proposed as a coexistence mechanism in microalgae‐virus interactions (Yau et al., 2020). As shown by Blanc‐Mathieu et al. (2017), smaller SOC sizes are associated with decreased resistance spectrum. Thus, the observed size variations, particularly in Arctic strains, might reflect the immune history of the corresponding strains, selecting a more compact chromosome 19 for better local fitness and specific resistance at the expense of broader resistance range. However, these could also be the sign of continuous rearrangement at the time of sampling due to ongoing viral infection.

The smallest SOCs may also provide valuable insight into the structure of *B. prasinos* chromosome 19, as the sequences found in these reduced chromosomes tend to be conserved in nearly all strains and located at a distal position. This separates the SOC into a conserved portion and a more variable one. The smallest chromosomes may represent a minimal chromosomal content, consistent with the presence of a substantial set of core genes within an otherwise variable chromosome composed largely of population‐specific genes (Figure 8b). This observation aligns with the bipartite pattern of SOC transcription reported for *O. tauri* by Yau et al. (2016), in which one half of the chromosome is expressed in susceptible strains while the other half is expressed by resistant strains. Therefore, it suggests a common regulation of both transcription and structural rearrangement in this region to minimize loss of fitness in favor of genomic diversity for rapid genomic adaptation. Yau et al. (2016) proposed several theories to explain these observations, including the activation of retrotransposons and epigenetic modifications in response to biotic stress. To comprehensively test these theories, an in‐depth characterization of SOC structural variations, including gene content and structure, is necessary. As such, the extensive dataset described here represents a valuable resource for understanding the intricate interactions between *Mamiellophyceae* and associated viruses.

## CONCLUSION

The biological and associated genomic resources of *Bathycoccus* presented in this study cover an unprecedented range of latitudes for a eukaryotic phytoplankton species. Genotyping and *de novo* whole‐genome sequencing of newly isolated strains provide high‐quality reference genomes for three cryptic species of *Bathycoccus* including *B. prasinos*, *B. calidus*, and *B. catiminus*. This includes 24 high‐quality genomes of the cosmopolitan species *B. prasinos*, allowing for a deep exploration of the intraspecific diversity of this ecologically significant species.

This resource complements the MAGs currently available and described by Xu et al. (2024). However, comparison of these MAGs with genomic data from isolated strains revealed that while MAGs are crucial for characterizing uncultured species, such as *Bathycoccus* B4 (Xu et al., 2024), their quality is inadequate for resolving genomic structural variations such as for BOC and SOC outlier chromosomes. Moreover, their large variations in completeness and quality limit high‐resolution diversity analysis. This emphasizes the importance of isolating and extensively sequencing additional strains of ecologically relevant species, such as *B. prasinos*.

Comparative analysis focused on *B. prasinos* strains also provided further biological insights into the putative function of outlier chromosomes. In addition to identifying a second BOC haplotype that was not detected in MAGs, we observed a low polymorphism in the outlier region of this putative sexual chromosome, suggesting a strong selective pressure consistent with its proposed biological function (Blanc‐Mathieu et al., 2017; Grimsley et al., 2015). Meanwhile, a more detailed characterization of SOC hypervariability revealed a significantly higher level of sequence conservation than previously reported and uncovered a bipartite SOC structure that varies across oceanic regions. This provides a valuable resource for investigating the molecular mechanisms underlying viral resistance in natural environments.

Altogether, the *Bathycoccus* genomic resources established here pave the way for multi‐scale comparative analyses at the genus level, and for the construction of a first species‐specific pangenome for *B. prasinos*. This genomic resource, combined with metagenomic studies, physiological analysis of *Bathycoccus* natural variants, and recently developed genetic engineering tools for *B. prasinos* (Faktorová et al., 2020), offers new opportunities to study the molecular basis of adaptation underlying the ecological success of the *Bathycoccus* genus and its cosmopolitan representative species *B. prasinos*.

## EXPERIMENTAL PROCEDURES

### Algal strains and culture conditions

Selection of *Bathycoccus* sp. strains from the RCC and isolated strains from the Banyuls Bay are detailed in Devic et al. (2024). Culture conditions of available and isolated Mediterranean strains are described in Devic et al. (2024). Arctic isolates were grown at 4°C or 15°C under constant light (10 μE).

### Sampling and cell isolation

Sea water sampling at the SOLA buoy in Banyuls Bay and the isolation of *Bathycoccus* strains were reported earlier by Devic et al. (2024).

Sampling in the Baffin Bay was performed during the DarkEdge campaign in October 2021 (Figure 1a). Samples were filtered using a 1.2 μm pore‐size Acrodisc filter (FP 30/1.2 CA‐S, Cat. No. 10462260; Whatman, GE Healthcare Life Sciences, Little Chalfont, Buckinghamshire, UK). Sea water was supplemented with vitamins, NaH_2_PO_4_, NaNO_3_ and metal traces at the same concentration as in L1 culture medium. For samples isolated from the DE310 ice station, the salinity of the culture medium was halved by adding 10 ml of mQ water. Antibiotics (Streptomycin sulfate 100 μg ml^−1^) were added to half of the samples to limit bacterial growth. Finally, cultures were incubated on board either at 4°C or 15°C for 10–20 days under constant light before shipping through express carriers to Banyuls‐sur‐Mer (France) at 4°C or 15°C depending on the culture conditions. In the laboratory, the presence of picophytoplankton was checked using a BD accuri C6 flow cytometer. Cultures containing at least 90% of picophytoplankton were used for plating in low melting agarose 0.21% W/v as described by Devic et al. (2024). Colonies appearing after 10–15 days at 15°C, and up to a month at 4°C, were handpicked and further cultured. Individual *Bathycoccus* cells were obtained from three sampling zones at 4°C and 15°C with or without antibiotics (Table S1).

### Genotyping

Genotyping, identification, or detection of strains was performed by PCR in 25 or 50 μl final volume containing DNA, REDTaq polymerase 2× master mix (VWR), 200 μM final of each primer and water. Amplification began with a pre‐denaturing step at 95°C for 3 min, followed by 40 cycles (95°C for 20 sec, annealing at 58°C for 30 sec, extension at 72°C for 1 min) and a final extension step for 5 min. The PCR products were loaded onto an agarose gel to verify their size and, if necessary for Sanger sequencing, to be purified using the NucleoSpin Gel and PCR Clean‐up kit (Macherey Nagel GmbH & Co. KG, Düren, Germany).

The identification of *Bathycoccus* sp. strains from the Banyuls Bay was performed through specific amplifications of a 614 bp fragment of the LOV‐HK gene (Bathy10g02360) and 18S rDNA followed by Sanger sequencing (GENEWIZ), as reported by Devic et al. (2024). 55 isolates were unambiguously identified as *B. prasinos* by Devic et al. (2024), while 11 strains showed a low amplification signal with the LOV‐HK primers but were determined to be *Bathycoccus* sp. by 18S rDNA sequencing results. We amplified the ITS2 region with 5′‐GTACACACCGCCCGTCGC‐3′ and 5′‐ATATGCTTAARTTCAGCGGGT‐3′ primers for these 11 strains. Sanger sequencing of the PCR products led to the identification of *B. catiminus*. Primers in the variable region of the Flavodoxin‐like gene (Bathy03g02080; 5′‐GCAAGAGAAGATTGAGGCGGAA‐3′ and 5′‐CTCTGCTGCCGCTTTTGCCTCA‐3′) were designed for *B. catiminus* to select the most variable isolates for whole‐genome sequencing. Detection of *B. catiminus* in seawater was done by PCR amplification of the same environmental DNA samples reported by Devic et al. (2024) with specific primers in the TOC1 ORF, TOCNI5B3 (5′‐GGGACCCACCACAGGTTGCTGT‐3′) and TOCNI3B3 (5′‐TACCGCGAGCAGCAACAGTAGT‐3′).

Since LOV‐HK primers failed to amplify *Bathycoccus* strains from the Baffin Bay, the identification of *Bathycoccus* sp. strains was performed through specific amplification of a portion of TOC1 ORF (Bathy17g01510) with primer TOCNI5 (5′‐AGGGGTTTTTGCAGAAACCGCT‐3′) and TOCNI3 (5′‐TCTCGCATTTGATTTCGAGTCCA‐3′). Intra‐species diversity of Baffin Bay strains was assessed using two markers, a fragment of the flavodoxin‐like gene (Bathy03g02080) and the C‐terminal region of the TIM gene (Bathy14g30100) (Devic et al., 2024), resulting in the identification of 10 multi‐loci genotypes (Table S1).

### Ultrastructure of *Bathycoccus* species

Cells were prepared according to Chrétiennot‐Dinet et al. (1995). Thin sections were stained with uranyl acetate and lead citrate and observed with a 7500 Hitachi transmission electron microscope.

### Genome sequencing and assembly

Isolates from the Baffin Bay were cultured at 4°C before DNA extraction, other strains were grown at 15°C. DNA extraction and Oxford Nanopore Technology (ONT) genome sequencing were performed on 28 *Bathycoccus* sp. strains as described in Devic et al. (2024). Raw ONT data were basecalled using Guppy 6.1.2 (https://nanoporetech.com) and the SUP model; reads with a PHRED score higher than 7 were retained for assembly. Quality control was performed with NanoPlot 1.19.0 (De Coster et al., 2018). Illumina pair‐end sequencing (Genewiz Europe, Leipzig, Germany and Novogene Europe, Cambridge, UK) was also performed on the same extracted DNA, yielding between 450 Mb and 2 Gb (~30–130× coverage) of sequences per strain. Paired‐end sequencing of 250 bp long was used for strains from the RCC and the Banyuls Bay, while 150 bp paired‐end sequencing was used for strains isolated during the DarkEdge cruise.

Genome assemblies were produced using FLYE 2.9 (Kolmogorov et al., 2019) (options: ‐‐nano‐hq ‐‐genome size 15 m). Assembly polishing with ONT reads was performed using MEDAKA 1.5 (https://github.com/nanoporetech/medaka) (options: ‐m r941_min_high_g360). Assembly correction with Illumina reads was performed using BWA 0.7.17 (Li, 2013) for read mapping, Samtools 1.9 (Danecek et al., 2021), and PILON 1.24 (Walker et al., 2014), with standard options. Scaffolding and direction of contigs on the reference genome (Strain RCC1105) was performed using RagTag 2.1.0 (Alonge et al., 2021). Unmapped contigs, derived from RagTag scaffolding, were taxonomically assigned using Blobtools 1.1.1 (Laetsch & Blaxter, 2017) (Data S2). Most unmapped contigs were labeled as contaminating DNA, mainly including extracted DNA from bacteria associated in culture with *Bathycoccus*. Contigs assigned to the *Chlorophyta* phylum were either small (<10 kb) or presented inconsistent coverage compared to mapped contigs. Therefore, all unmapped contigs were considered contaminating DNA and excluded. Gap filling was performed using TGS‐GapCloser 1.0.1 (Xu et al., 2020) with Samtools 1.9 and PILON 1.24. One round of Pilon correction was applied to corrected gaps for consistency with the global assembly correction (options: ‐‐p_round 1 ‐‐r_round 0 ‐‐min_nread 3). Abnormal chromosomal fusions due to assembly error were manually checked using D‐GENIES 1.5.0 (Cabanettes & Klopp, 2018) and corrected using samtools (samtools faidx) (Danecek et al., 2021).

Assembly statistics were calculated with Assembly_stats 0.1.4 (https://github.com/MikeTrizna/assembly_stats) and QUAST 5.2.0 (Gurevich et al., 2013). Completion was assessed through BUSCO 5.4.4 (Manni et al., 2021) using the *chlorophyta_odb10* library, and quality was estimated with MERQURY 1.1 (Rhie et al., 2020) with the corresponding Illumina reads as input.

Basecalling and assembly scripts used in this paper are available at https://github.com/LouisDennu/Bathycoccus_GenomeAssemblyAndAnnotation.

### Genome annotation

Genome assemblies were individually submitted to RepeatModeler 2.0.1 (https://github.com/Dfam‐consortium/RepeatModeler) to build strain specific repeat libraries, which were subsequently concatenated in a non‐redundant repeat library using CD‐hit (Fu et al., 2012) (90% identity and coverage). This library was used with RepeatMasker 4.1.2 (https://github.com/rmhubley/RepeatMasker) on all samples to produce soft masked assemblies.

Available Illumina RNAseq whole transcriptome of *B. prasinos* strain RCC4752 (SRX554258) (Keeling et al., 2014) was mapped upon the RCC4752 genome assembly using HISAT2 2.1.0 (Kim et al., 2015), and transcripts inferred using STRINGTIE 1.3.4 (Shumate et al., 2022). Plant orthologous proteins database 10 (Kriventseva et al., 2019) was used as protein evidences for annotation.

Training of GENEMARK‐ETP 4.71 (Brůna et al., 2023) and AUGUSTUS 3.3.3 (Stanke et al., 2008) *ab initio* prediction models were performed through BRAKER3 2.1.6 (Buchfink et al., 2015; Gabriel et al., 2023; Gotoh, 2008; Lomsadze et al., 2014), using transcriptome mapping data and the plant protein database as evidence. Training of SNAP (Korf, 2004) *ab initio* prediction model was performed after a first round of MAKER 2.31.9 (Campbell et al., 2014; Cantarel et al., 2008), using genome assembled transcripts, available *de novo* assembled transcripts (https://www.ncbi.nlm.nih.gov/Traces/wgs?val=HBMR01) and plant protein database as evidence. GENEMARK‐ETP, AUGUSTUS and SNAP prediction models were merged through a second round of Maker to produce *de novo* structural annotation.

Annotation scripts used in this paper are available at https://github.com/LouisDennu/Bathycoccus_GenomeAssemblyAndAnnotation.

### Phylogenetic analysis

BUSCO (Manni et al., 2021) analysis with *chlorophyta_odb10* library was computed for each assembled genome and on *O. tauri* and *Micromonas pusilla* reference genomes to be used as outgroups. This resulted in 1200 predicted genes shared across all genomes. Amino acid sequences were aligned for each gene using MAFFT 7.520 (options: ‐‐auto ‐‐maxiterate 1000) (Katoh & Standley, 2013) and converted to nucleic acid alignment using PAL2NAL 14 (Suyama et al., 2006) to ensure accurate coding sequence alignment.

Sequence alignments were concatenated in a partitioned supermatrix using catfasta2phyml.pl script (https://github.com/nylander/catfasta2phyml). Maximum‐likelihood phylogenetic inferences with gene concordance factors were computed through a partitioned analysis for multi‐gene alignments using IQ‐TREE 2.2.0.3 (Chernomor et al., 2016; Minh et al., 2020), with ModelFinder for automatic model finding (Kalyaanamoorthy et al., 2017) and 1000 ultrafast bootstrap (Hoang et al., 2018) (options: ‐B 1000 ‐m MFP+MERGE).

### rRNA structure prediction

2D structure prediction of the 18S rRNA ITS2 region was computed using RNAfold (Lorenz et al., 2011) and default parameters. Predictions are displayed using centroid structures.

### Comparative genomics

Complete genomes dotplot comparison was computed using D‐GENIES 1.5.0 (Cabanettes & Klopp, 2018) with Minimap2 2.24 (Li, 2018) as aligner and ‘Few repeats’ option.

Genome assembly alignments were performed with MUMmer 3.1 (Kurtz et al., 2004) nucmer (options: ‐‐maxmatch ‐c 500 ‐b 500 ‐l 200); alignments were then filtered for identity (>90%) and length (>100 bp) using delta‐filter (options: ‐m ‐i 90 ‐l 100). Synteny, single nucleotide polymorphism, and structural variations were identified from the MUMmer output by SYRI 1.6 (Goel et al., 2019) and visualized with Plotsr 0.5.3 (Goel & Schneeberger, 2022).

Homologous clustering of gene CDS from the BOC and SOC chromosomic regions was performed using get_homologues‐est 3.4.2 (Contreras‐Moreira et al., 2017) (options: ‐M ‐C 80 ‐S 80 ‐t 0) with 80% sequence identity and 80% sequence coverage clustering thresholds. Gene Ontology (GO) enrichment analysis was computed using the topGO 2.54.0 package (Alexa & Rahnenführer, 2025) against a background gene set consisting of all annotated genes defined from a genome‐wide automatic functional annotation via Interproscan 5.53.87.0 (Jones et al., 2014).

### Mapping of metagenomic datasets

Metagenomic reads from the TARA Ocean and TARA Polar Circle (De Vargas et al., 2015) campaigns were mapped to genome sequences using BWA mem 0.7.17 (Li, 2013). Samtools 1.13 (Danecek et al., 2021) was used to recover mapped reads (samtools view, options: ‐F 4) and to remove duplicates to avoid bias due to PCR artifacts. Using bamFilters (https://github.com/institut‐de‐genomique/bamFilters), mapped reads were filtered out for low‐complexity bases (>75%), high‐complexity bases (<30%), coverage (<80%), and identity (<95%). Remaining reads were retained for further analysis. Relative abundance was computed for each sample as the number of reads mapped normalized by the number of reads sequenced.

For *Bathycoccus* sp. species biogeography, complete assembled genomes of strains RCC4222, RCC716, and G8 were respectively used as reference for *B. prasinos*, *B. calidus*, and *B. catiminus*. For BOC haplotypes, Chr14:232230‐630064 genome segment of strain RCC4222 and Chr14:231394‐743201 genome segment of strain A8 were respectively used as reference for BOC A and BOC B outlier regions, according to loss of synteny.

## ACCESSION NUMBERS

Whole‐genome sequences, annotation files, and basecalled reads of the strains sequenced in this study are available at the European Nucleotide Archive under project accession PRJEB67444. All strains sequenced in this study have been deposited in the Roscoff Culture Collection (RCC11109–RCC11132).

## AUTHOR CONTRIBUTIONS

F‐YB, FS and MD designed the study. MD, AF and NJ designed and carried out the sampling. MD, J‐CL, VV and CM isolated, genotyped strains and produced the sequencing data. LD, JR and FS performed bioinformatic analyses. MD, LD, FS and F‐YB described the novel species *B. catiminus*. LD, FS and F‐YB wrote the manuscript. All authors reviewed the final version of the manuscript.

## CONFLICT OF INTEREST

The authors report no conflicts of interest.

## Supporting information


**Data S1.** Quast output for all assembled genomes against the reference genome.

**Data S2.** Taxonomic partitioning of assembled contigs.

**Appendix S1.** Taxonomic description of *Bathycoccus catiminus*.
**Figure S1.** (A) Maximum‐likelihood phylogeny and (B) 2D centroid structures of available *Bathycoccus* 18S rRNA ITS2 region.
**Figure S2.** Clonal conservation of the genome structure between reference strain RCC1105 and clonal strain RCC4222.
**Figure S3.** Structural annotation pipeline of repeat and coding sequences.
**Figure S4.** Biogeography of the *Bathycoccus* genus. Geographical distribution of *Bathycoccus* species based on read mapping from TARA ocean metagenomic datasets.
**Figure S5.** BOC haplotypes shared sequences.
**Figure S6.** Genomic diversity of Chromosome 1 in *Bathycoccus prasinos*.
**Figure S7.** BOC gene cluster presence frequency between haplotypes.
**Figure S8.** Chromosome 19 pairwise alignments between *B. prasinos* strains.

**Table S1.** Multi‐loci genotypes identified in isolated strains from water samples collected in the Baffin Bay during the DarkEdge cruise.

**Table S2.** Prevalence of annotated repeat types in assembled genomes.

**Table S3.** Statistics of annotated coding sequences in assembled genomes.

**Table S4.** *Bathycoccus prasinos* BOC GO enrichment analysis.

**Table S5.** *Bathycoccus prasinos* SOC GO enrichment analysis.

## Data Availability

The data that support the findings of this study are openly available in European Nucleotide Archive (ENA) at https://www.ebi.ac.uk/ena/browser/home, reference number PRJEB67444.

## References

[tpj70982-bib-0001] 3,000 Rice Genomes Project . (2014) The 3,000 rice genomes project. GigaScience, 3, 7. Available from: 10.1186/2047-217X-3-7 24872877 PMC4035669

[tpj70982-bib-0002] Alexa, A. & Rahnenführer, J. (2025) topGO: Enrichment analysis for gene ontology. Available from: 10.18129/B9.bioc.topGO

[tpj70982-bib-0003] Alonge, M. , Lebeigle, L. , Kirsche, M. , Aganezov, S. , Wang, X. , Lippman, Z.B. et al. (2021) Automated assembly scaffolding elevates a new tomato system for high‐throughput genome editing. Available from: 10.1101/2021.11.18.469135 PMC9753292 36522651

[tpj70982-bib-0004] Alonso‐Blanco, C. , Aarts, M.G.M. , Bentsink, L. , Keurentjes, J.J.B. , Reymond, M. , Vreugdenhil, D. et al. (2009) What has natural variation taught us about plant development, physiology, and adaptation? The Plant Cell, 21, 1877–1896. Available from: 10.2307/40536969 19574434 PMC2729614

[tpj70982-bib-0005] Alonso‐Blanco, C. , Andrade, J. , Becker, C. , Bemm, F. , Bergelson, J. , Borgwardt, K.M. et al. (2016) 1,135 genomes reveal the global pattern of polymorphism in *Arabidopsis thaliana* . Cell, 166, 481–491. Available from: 10.1016/j.cell.2016.05.063 27293186 PMC4949382

[tpj70982-bib-0006] Bachy, C. , Yung, C.C.M. , Needham, D.M. , Gazitúa, M.C. , Roux, S. , Limardo, A.J. et al. (2021) Viruses infecting a warm water picoeukaryote shed light on spatial co‐occurrence dynamics of marine viruses and their hosts. The ISME Journal, 15, 3129–3147. Available from: 10.1038/s41396-021-00989-9 33972727 PMC8528832

[tpj70982-bib-0007] Beaulieu, C. , Libourel, C. , Mbadinga Zamar, D.L. , El Mahboubi, K. , Hoey, D.J. , Greiff, G.R.L. et al. (2025) The *Marchantia polymorpha* pangenome reveals ancient mechanisms of plant adaptation to the environment. Nature Genetics, 57, 729–740. Available from: 10.1038/s41588-024-02071-4 39962240 PMC11906373

[tpj70982-bib-0008] Belevich, T.A. , Milyutina, I.A. , Abyzova, G.A. & Troitsky, A.V. (2021) The pico‐sized Mamiellophyceae and a novel *Bathycoccus* clade from the summer plankton of Russian Arctic seas and adjacent waters. FEMS Microbiology Ecology, 97, fiaa251. Available from: 10.1093/femsec/fiaa251 33307552

[tpj70982-bib-0009] Bendif, E.M. , Probert, I. , Archontikis, O.A. , Young, J.R. , Beaufort, L. , Rickaby, R.E. et al. (2023) Rapid diversification underlying the global dominance of a cosmopolitan phytoplankton. The ISME Journal, 17, 630–640. Available from: 10.1038/s41396-023-01365-5 36747097 PMC10030636

[tpj70982-bib-0010] Benites, L.F. , Poulton, N.J. , Labadie, K. , Sieracki, M.E. , Grimsley, N. & Piganeau, G. (2019) Single cell ecogenomics reveals mating types of individual cells and ssDNA viral infections in the smallest photosynthetic eukaryotes. Philosophical Transactions of the Royal Society B, 374, 20190089. Available from: 10.1098/rstb.2019.0089 PMC6792451 31587637

[tpj70982-bib-0011] Blanc‐Mathieu, R. , Krasovec, M. , Hebrard, M. , Yau, S. , Desgranges, E. , Martin, J. et al. (2017) Population genomics of picophytoplankton unveils novel chromosome hypervariability. Science Advances, 3, e1700239. Available from: 10.1126/sciadv.1700239 28695208 PMC5498103

[tpj70982-bib-0012] Botebol, H. , Lesuisse, E. , Šuták, R. , Six, C. , Lozano, J.‐C. , Schatt, P. et al. (2015) Central role for ferritin in the day/night regulation of iron homeostasis in marine phytoplankton. Proceedings. National Academy of Sciences. United States of America, 112, 14652–14657. Available from: 10.1073/pnas.1506074112 PMC4664360 26553998

[tpj70982-bib-0013] Brůna, T. , Lomsadze, A. & Borodovsky, M. (2023) GeneMark‐ETP: Automatic gene finding in eukaryotic genomes in consistence with extrinsic data. Available from: 10.1101/2023.01.13.524024

[tpj70982-bib-0014] Buchfink, B. , Xie, C. & Huson, D.H. (2015) Fast and sensitive protein alignment using DIAMOND. Nature Methods, 12, 59–60. Available from: 10.1038/nmeth.3176 25402007

[tpj70982-bib-0015] Cabanettes, F. & Klopp, C. (2018) D‐GENIES: dot plot large genomes in an interactive, efficient and simple way. PeerJ, 6, e4958. Available from: 10.7717/peerj.4958 29888139 PMC5991294

[tpj70982-bib-0016] Cai, H. , McLimans, C.J. , Beyer, J.E. , Krumholz, L.R. & Hambright, K.D. (2023) Microcystis pangenome reveals cryptic diversity within and across morphospecies. Science Advances, 9, eadd3783. Available from: 10.1126/sciadv.add3783 36638170 PMC9839332

[tpj70982-bib-0017] Campbell, M.S. , Holt, C. , Moore, B. & Yandell, M. (2014) Genome annotation and curation using MAKER and MAKER‐P. Current Protocols in Bioinformatics, 39, 4.11.1–4.11.39. Available from: 10.1002/0471250953.bi0411s48 PMC4286374 25501943

[tpj70982-bib-0018] Cantarel, B.L. , Korf, I. , Robb, S.M.C. , Parra, G. , Ross, E. , Moore, B. et al. (2008) MAKER: an easy‐to‐use annotation pipeline designed for emerging model organism genomes. Genome Research, 18, 188–196. Available from: 10.1101/gr.6743907 18025269 PMC2134774

[tpj70982-bib-0019] Chernomor, O. , von Haeseler, A. & Minh, B.Q. (2016) Terrace aware data structure for phylogenomic inference from supermatrices. Systematic Biology, 65, 997–1008. Available from: 10.1093/sysbio/syw037 27121966 PMC5066062

[tpj70982-bib-0020] Chrétiennot‐Dinet, M.‐J. , Courties, C. , Vaquer, A. , Neveux, J. , Claustre, H. , Lautier, J. et al. (1995) A new marine picoeucaryote: *Ostreococcus tauri* gen. et sp. nov. (Chlorophyta, Prasinophyceae). Phycologia, 34, 285–292. Available from: 10.2216/i0031-8884-34-4-285.1

[tpj70982-bib-0021] Contreras‐Moreira, B. , Cantalapiedra, C.P. , García‐Pereira, M.J. , Gordon, S.P. , Vogel, J.P. , Igartua, E. et al. (2017) Analysis of plant pan‐genomes and transcriptomes with GET_HOMOLOGUES‐EST, a clustering solution for sequences of the same species. Frontiers in Plant Science, 8, 184. Available from: 10.3389/fpls.2017.00184 28261241 PMC5306281

[tpj70982-bib-0022] Corellou, F. , Schwartz, C. , Motta, J.‐P. , Djouani‐Tahri, E.B. , Sanchez, F. & Bouget, F.‐Y. (2009) Clocks in the green lineage: comparative functional analysis of the circadian architecture of the picoeukaryote *Ostreococcus* . The Plant Cell, 21, 3436–3449. Available from: 10.1105/tpc.109.068825 19948792 PMC2798331

[tpj70982-bib-0023] Danecek, P. , Bonfield, J.K. , Liddle, J. , Marshall, J. , Ohan, V. , Pollard, M.O. et al. (2021) Twelve years of SAMtools and BCFtools. GigaScience, 10, giab008. Available from: 10.1093/gigascience/giab008 33590861 PMC7931819

[tpj70982-bib-0024] De Barros Dantas, L.L. , Eldridge, B.M. , Dorling, J. , Dekeya, R. , Lynch, D.A. & Dodd, A.N. (2023) Circadian regulation of metabolism across photosynthetic organisms. The Plant Journal, 16405, 650–668. Available from: 10.1111/tpj.16405 PMC10953457 37531328

[tpj70982-bib-0025] De Coster, W. , D'Hert, S. , Schultz, D.T. , Cruts, M. & Van Broeckhoven, C. (2018) NanoPack: visualizing and processing long‐read sequencing data. Bioinformatics, 34, 2666–2669. Available from: 10.1093/bioinformatics/bty149 29547981 PMC6061794

[tpj70982-bib-0026] De Hoff, P.L. , Ferris, P.J. , Olson, B.J.S.C. , Miyagi, A. , Geng, S. & Umen, J.G. (2013) Species and population level molecular profiling reveals cryptic recombination and emergent asymmetry in the dimorphic mating locus of *C. reinhardtii* . PLoS Genetics, 9, e1003724. Available from: 10.1371/journal.pgen.1003724 24009520 PMC3757049

[tpj70982-bib-0027] De Vargas, C. , Audic, S. , Henry, N. , Decelle, J. , Mahé, F. , Logares, R. et al. (2015) Eukaryotic plankton diversity in the sunlit ocean. Science, 348, 1261605. Available from: 10.1126/science.1261605 25999516

[tpj70982-bib-0028] Delmont, T.O. , Gaia, M. , Hinsinger, D.D. , Frémont, P. , Vanni, C. , Fernandez‐Guerra, A. et al. (2022) Functional repertoire convergence of distantly related eukaryotic plankton lineages abundant in the sunlit ocean. Cell Genomics, 2, 100123. Available from: 10.1016/j.xgen.2022.100123 36778897 PMC9903769

[tpj70982-bib-0029] Demir‐Hilton, E. , Sudek, S. , Cuvelier, M.L. , Gentemann, C.L. , Zehr, J.P. & Worden, A.Z. (2011) Global distribution patterns of distinct clades of the photosynthetic picoeukaryote *Ostreococcus* . The ISME Journal, 5, 1095–1107. Available from: 10.1038/ismej.2010.209 21289652 PMC3146286

[tpj70982-bib-0030] Derelle, E. , Ferraz, C. , Rombauts, S. , Rouzé, P. , Worden, A.Z. , Robbens, S. et al. (2006) Genome analysis of the smallest free‐living eukaryote *Ostreococcus tauri* unveils many unique features. Proceedings. National Academy of Sciences. United States of America, 103, 11647–11652. Available from: 10.1073/pnas.0604795103 PMC1544224 16868079

[tpj70982-bib-0031] Devic, M. , Dennu, L. , Lozano, J.‐C. , Mariac, C. , Vergé, V. , Schatt, P. et al. (2024) An INDEL genomic approach to explore population diversity of phytoplankton. BMC Genomics, 25, 1045. Available from: 10.1186/s12864-024-10896-w 39506649 PMC11539686

[tpj70982-bib-0032] Duncan, A. , Barry, K. , Daum, C. , Eloe‐Fadrosh, E. , Roux, S. , Schmidt, K. et al. (2022) Metagenome‐assembled genomes of phytoplankton microbiomes from the Arctic and Atlantic oceans. Microbiome, 10, 67. Available from: 10.1186/s40168-022-01254-7 35484634 PMC9047304

[tpj70982-bib-0033] Eikrem, W. & Throndsen, J. (1990) The ultrastructure of *Bathycoccus* gen. nov. and *B. prasinos* sp. nov., a non‐motile picoplanktonic alga (Chlorophyta, Prasinophyceae) from the Mediterranean and Atlantic. Phycologia, 29, 344–350. Available from: 10.2216/i0031-8884-29-3-344.1

[tpj70982-bib-0034] Faktorová, D. , Nisbet, R.E.R. , Fernández Robledo, J.A. , Casacuberta, E. , Sudek, L. , Allen, A.E. et al. (2020) Genetic tool development in marine protists: emerging model organisms for experimental cell biology. Nature Methods, 17, 481–494. Available from: 10.1038/s41592-020-0796-x 32251396 PMC7200600

[tpj70982-bib-0035] Ferris, J. & Goodenough, W. (1994) The mating‐type locus of *Chlamydomonas reinhardtii* contains highly rearranged DNA sequences. Cell, 76, 1135–1145. Available from: 10.1016/0092-8674(94)90389-1 8137428

[tpj70982-bib-0036] Fu, L. , Niu, B. , Zhu, Z. , Wu, S. & Li, W. (2012) CD‐HIT: accelerated for clustering the next‐generation sequencing data. Bioinformatics, 28, 3150–3152. Available from: 10.1093/bioinformatics/bts565 23060610 PMC3516142

[tpj70982-bib-0037] Gabriel, L. , Brůna, T. , Hoff, K.J. , Ebel, M. , Lomsadze, A. , Borodovsky, M. et al. (2023) BRAKER3: fully automated genome annotation using RNA‐Seq and protein evidence with GeneMark‐ETP, AUGUSTUS and TSEBRA. Available from: 10.1101/2023.06.10.544449 PMC11216308 38866550

[tpj70982-bib-0038] Gabur, I. , Chawla, H.S. , Snowdon, R.J. & Parkin, I.A.P. (2019) Connecting genome structural variation with complex traits in crop plants. Theoretical and Applied Genetics, 132, 733–750. Available from: 10.1007/s00122-018-3233-0 30448864

[tpj70982-bib-0039] Goel, M. & Schneeberger, K. (2022) Plotsr: visualizing structural similarities and rearrangements between multiple genomes. Bioinformatics, 38, 2922–2926. Available from: 10.1093/bioinformatics/btac196 35561173 PMC9113368

[tpj70982-bib-0040] Goel, M. , Sun, H. , Jiao, W.‐B. & Schneeberger, K. (2019) SyRI: finding genomic rearrangements and local sequence differences from whole‐genome assemblies. Genome Biology, 20, 277. Available from: 10.1186/s13059-019-1911-0 31842948 PMC6913012

[tpj70982-bib-0041] Gordon, S.P. , Contreras‐Moreira, B. , Woods, D.P. , Des Marais, D.L. , Burgess, D. , Shu, S. et al. (2017) Extensive gene content variation in the *Brachypodium distachyon* pan‐genome correlates with population structure. Nature Communications, 8, 2184. Available from: 10.1038/s41467-017-02292-8 PMC5736591 29259172

[tpj70982-bib-0042] Gotoh, O. (2008) A space‐efficient and accurate method for mapping and aligning cDNA sequences onto genomic sequence. Nucleic Acids Research, 36, 2630–2638. Available from: 10.1093/nar/gkn105 18344523 PMC2377433

[tpj70982-bib-0043] Grimsley, N. , Yau, S. , Piganeau, G. & Moreau, H. (2015) Typical features of genomes in the Mamiellophyceae. In: Marine protists. Tokyo: Springer Japan, pp. 107–127. Available from: 10.1007/978-4-431-55130-0_6

[tpj70982-bib-0044] Gurevich, A. , Saveliev, V. , Vyahhi, N. & Tesler, G. (2013) QUAST: quality assessment tool for genome assemblies. Bioinformatics, 29, 1072–1075. Available from: 10.1093/bioinformatics/btt086 23422339 PMC3624806

[tpj70982-bib-0045] Hanschen, E.R. & Starkenburg, S.R. (2020) The state of algal genome quality and diversity. Algal Research, 50, 101968. Available from: 10.1016/j.algal.2020.101968

[tpj70982-bib-0046] Hasan, A.R. , Duggal, J.K. & Ness, R.W. (2019) Consequences of recombination for the evolution of the mating type locus in *Chlamydomonas reinhardtii* . The New Phytologist, 224, 1339–1348. Available from: 10.1111/nph.16003 31222749

[tpj70982-bib-0047] Hasan, A.R. & Ness, R.W. (2020) Recombination rate variation and infrequent sex influence genetic diversity in *Chlamydomonas reinhardtii* . Genome Biology and Evolution, 12, 370–380. Available from: 10.1093/gbe/evaa057 32181819 PMC7186780

[tpj70982-bib-0048] Hoang, D.T. , Chernomor, O. , von Haeseler, A. , Minh, B.Q. & Vinh, L.S. (2018) UFBoot2: improving the ultrafast bootstrap approximation. Molecular Biology and Evolution, 35, 518–522. Available from: 10.1093/molbev/msx281 29077904 PMC5850222

[tpj70982-bib-0049] Joli, N. , Monier, A. , Logares, R. & Lovejoy, C. (2017) Seasonal patterns in Arctic prasinophytes and inferred ecology of *Bathycoccus* unveiled in an Arctic winter metagenome. The ISME Journal, 11, 1372–1385. Available from: 10.1038/ismej.2017.7 28267153 PMC5437359

[tpj70982-bib-0050] Jones, P. , Binns, D. , Chang, H.‐Y. , Fraser, M. , Li, W. , McAnulla, C. et al. (2014) InterProScan 5: genome‐scale protein function classification. Bioinformatics, 30, 1236–1240. Available from: 10.1093/bioinformatics/btu031 24451626 PMC3998142

[tpj70982-bib-0051] Kalyaanamoorthy, S. , Minh, B.Q. , Wong, T.K.F. , von Haeseler, A. & Jermiin, L.S. (2017) ModelFinder: fast model selection for accurate phylogenetic estimates. Nature Methods, 14, 587–589. Available from: 10.1038/nmeth.4285 28481363 PMC5453245

[tpj70982-bib-0052] Katoh, K. & Standley, D.M. (2013) MAFFT multiple sequence alignment software version 7: improvements in performance and usability. Molecular Biology and Evolution, 30, 772–780. Available from: 10.1093/molbev/mst010 23329690 PMC3603318

[tpj70982-bib-0053] Keeling, P.J. , Burki, F. , Wilcox, H.M. , Allam, B. , Allen, E.E. , Amaral‐Zettler, L.A. et al. (2014) The marine microbial eukaryote transcriptome sequencing project (MMETSP): illuminating the functional diversity of eukaryotic life in the oceans through transcriptome sequencing. PLoS Biology, 12, 1–6. Available from: 10.1371/journal.pbio.1001889 PMC4068987 24959919

[tpj70982-bib-0054] Kim, D. , Langmead, B. & Salzberg, S.L. (2015) HISAT: a fast spliced aligner with low memory requirements. Nature Methods, 12, 357–360. Available from: 10.1038/nmeth.3317 25751142 PMC4655817

[tpj70982-bib-0055] Kolmogorov, M. , Yuan, J. , Lin, Y. & Pevzner, P.A. (2019) Assembly of long, error‐prone reads using repeat graphs. Nature Biotechnology, 37, 540–546. Available from: 10.1038/s41587-019-0072-8 30936562

[tpj70982-bib-0056] Korf, I. (2004) Gene finding in novel genomes. BMC Bioinformatics, 5, 59. Available from: 10.1186/1471-2105-5-59 15144565 PMC421630

[tpj70982-bib-0057] Kriventseva, E.V. , Kuznetsov, D. , Tegenfeldt, F. , Manni, M. , Dias, R. , Simão, F.A. et al. (2019) OrthoDB v10: sampling the diversity of animal, plant, fungal, protist, bacterial and viral genomes for evolutionary and functional annotations of orthologs. Nucleic Acids Research, 47, D807–D811. Available from: 10.1093/nar/gky1053 30395283 PMC6323947

[tpj70982-bib-0058] Kurtz, S. , Phillippy, A. , Delcher, A.L. , Smoot, M. , Shumway, M. , Antonescu, C. et al. (2004) Versatile and open software for comparing large genomes. Genome Biology, 5, R12. Available from: 10.1186/gb-2004-5-2-r12 14759262 PMC395750

[tpj70982-bib-0059] Laetsch, D.R. & Blaxter, M.L. (2017) BlobTools: interrogation of genome assemblies. F1000Research, 6, 1287. Available from: 10.12688/f1000research.12232.1

[tpj70982-bib-0060] Lambert, S. , Tragin, M. , Lozano, J.‐C. , Ghiglione, J.‐F. , Vaulot, D. , Bouget, F.‐Y. et al. (2019) Rhythmicity of coastal marine picoeukaryotes, bacteria and archaea despite irregular environmental perturbations. The ISME Journal, 13, 388–401. Available from: 10.1038/s41396-018-0281-z 30254323 PMC6331585

[tpj70982-bib-0061] Leconte, J. , Benites, L.F. , Vannier, T. , Wincker, P. , Piganeau, G. & Jaillon, O. (2020) Genome resolved biogeography of Mamiellales. Genes, 11, 66. Available from: 10.3390/genes11010066 31936086 PMC7016971

[tpj70982-bib-0062] Leliaert, F. , Smith, D.R. , Moreau, H. , Herron, M.D. , Verbruggen, H. , Delwiche, C.F. et al. (2012) Phylogeny and molecular evolution of the green algae. Critical Reviews in Plant Sciences, 31, 1–46. Available from: 10.1080/07352689.2011.615705

[tpj70982-bib-0063] Li, H. (2013) Aligning sequence reads, clone sequences and assembly contigs with BWA‐MEM. Available from: 10.48550/arXiv.1303.3997

[tpj70982-bib-0064] Li, H. (2018) Minimap2: pairwise alignment for nucleotide sequences. Bioinformatics, 34, 3094–3100. Available from: 10.1093/bioinformatics/bty191 29750242 PMC6137996

[tpj70982-bib-0065] Li, W.K.W. (1994) Primary production of prochlorophytes, cyanobacteria, and eucaryotic ultraphytoplankton: measurements from flow cytometric sorting. Limnology and Oceanography, 39, 169–175. Available from: 10.4319/lo.1994.39.1.0169

[tpj70982-bib-0066] Lomsadze, A. , Burns, P.D. & Borodovsky, M. (2014) Integration of mapped RNA‐Seq reads into automatic training of eukaryotic gene finding algorithm. Nucleic Acids Research, 42, e119. Available from: 10.1093/nar/gku557 24990371 PMC4150757

[tpj70982-bib-0067] Lorenz, R. , Bernhart, S.H. , Höner Zu Siederdissen, C. , Tafer, H. , Flamm, C. , Stadler, P.F. et al. (2011) ViennaRNA Package 2.0. Algorithms for Molecular Biology, 6(1), 26. Available from: 10.1186/1748-7188-6-26 22115189 PMC3319429

[tpj70982-bib-0068] Lozano, J.‐C. , Schatt, P. , Botebol, H. , Vergé, V. , Lesuisse, E. , Blain, S. et al. (2014) Efficient gene targeting and removal of foreign DNA by homologous recombination in the picoeukaryote *Ostreococcus* . The Plant Journal, 78, 1073–1083. Available from: 10.1111/tpj.12530 24698018

[tpj70982-bib-0069] Lynch, M. , Gabriel, W. & Wood, A.M. (1991) Adaptive and demographic responses of plankton populations to environmental change. Limnology and Oceanography, 36, 1301–1312. Available from: 10.4319/lo.1991.36.7.1301

[tpj70982-bib-0070] Manni, M. , Berkeley, M.R. , Seppey, M. & Zdobnov, E.M. (2021) BUSCO: assessing genomic data quality and beyond. Current Protocols, 1, e323. Available from: 10.1002/cpz1.323 34936221

[tpj70982-bib-0072] Meziti, A. , Rodriguez‐R, L.M. , Hatt, J.K. , Peña‐Gonzalez, A. , Levy, K. & Konstantinidis, K.T. (2021) The reliability of metagenome‐assembled genomes (MAGs) in representing natural populations: insights from comparing MAGs against isolate genomes derived from the same fecal sample. Applied and Environmental Microbiology, 87, e02593‐20. Available from: 10.1128/AEM.02593-20 33452027 PMC8105024

[tpj70982-bib-0073] Minh, B.Q. , Schmidt, H.A. , Chernomor, O. , Schrempf, D. , Woodhams, M.D. , von Haeseler, A. et al. (2020) IQ‐TREE 2: new models and efficient methods for phylogenetic inference in the genomic era. Molecular Biology and Evolution, 37, 1530–1534. Available from: 10.1093/molbev/msaa015 32011700 PMC7182206

[tpj70982-bib-0074] Moreau, H. , Verhelst, B. , Couloux, A. , Derelle, E. , Rombauts, S. , Grimsley, N. et al. (2012) Gene functionalities and genome structure in *Bathycoccus prasinos* reflect cellular specializations at the base of the green lineage. Genome Biology, 13, R74. Available from: 10.1186/gb-2012-13-8-r74 22925495 PMC3491373

[tpj70982-bib-0075] Moulager, M. , Corellou, F. , Vergé, V. , Escande, M.‐L. & Bouget, F.‐Y. (2010) Integration of light signals by the retinoblastoma pathway in the control of S phase entry in the picophytoplanktonic cell *Ostreococcus* . PLoS Genetics, 6, e1000957. Available from: 10.1371/journal.pgen.1000957 20502677 PMC2873908

[tpj70982-bib-0076] O'Neill, J.S. , Van Ooijen, G. , Dixon, L.E. , Troein, C. , Corellou, F. , Bouget, F.‐Y. et al. (2011) Circadian rhythms persist without transcription in a eukaryote. Nature, 469, 554–558. Available from: 10.1038/nature09654 21270895 PMC3040569

[tpj70982-bib-0098] Paerl, R.W. , Bouget, F.‐Y. , Lozano, J.‐C. , Vergé, V. , Schatt, P. , Allen, E.E. et al. (2017) Use of plankton‐derived vitamin B1 precursors, especially thiazole‐related precursor, by key marine picoeukaryotic phytoplankton. The ISME Journal, 11, 753–765. Available from: 10.1038/ismej.2016.145 27935586 PMC5322297

[tpj70982-bib-0077] Peter, J. , De Chiara, M. , Friedrich, A. , Yue, J.‐X. , Pflieger, D. , Bergström, A. et al. (2018) Genome evolution across 1,011 *Saccharomyces cerevisiae* isolates. Nature, 556, 339–344. Available from: 10.1038/s41586-018-0030-5 29643504 PMC6784862

[tpj70982-bib-0078] Piganeau, G. , Eyre‐Walker, A. , Grimsley, N. & Moreau, H. (2011) How and why DNA barcodes underestimate the diversity of microbial eukaryotes. PLoS One, 6, e16342. Available from: 10.1371/journal.pone.0016342 21347361 PMC3037371

[tpj70982-bib-0079] Rastogi, A. , Vieira, F.R.J. , Deton‐Cabanillas, A.‐F. , Veluchamy, A. , Cantrel, C. , Wang, G. et al. (2020) A genomics approach reveals the global genetic polymorphism, structure, and functional diversity of ten accessions of the marine model diatom *Phaeodactylum tricornutum* . The ISME Journal, 14, 347–363. Available from: 10.1038/s41396-019-0528-3 31624346 PMC6976637

[tpj70982-bib-0080] Read, B.A. , Kegel, J. , Klute, M.J. , Kuo, A. , Lefebvre, S.C. , Maumus, F. et al. (2013) Pan genome of the phytoplankton *Emiliania* underpins its global distribution. Nature, 499, 209–213. Available from: 10.1038/nature12221 23760476

[tpj70982-bib-0081] Rhie, A. , Walenz, B.P. , Koren, S. & Phillippy, A.M. (2020) Merqury: reference‐free quality, completeness, and phasing assessment for genome assemblies. Genome Biology, 21, 245. Available from: 10.1186/s13059-020-02134-9 32928274 PMC7488777

[tpj70982-bib-0082] Shi, C. , Liu, X. , Han, K. , Peng, L. , Li, L. , Ge, Q. et al. (2021) A database and comprehensive analysis of the algae genomes. Available from: 10.1101/2021.10.30.466624

[tpj70982-bib-0083] Shumate, A. , Wong, B. , Pertea, G. & Pertea, M. (2022) Improved transcriptome assembly using a hybrid of long and short reads with StringTie. PLoS Computational Biology, 18, e1009730. Available from: 10.1371/journal.pcbi.1009730 35648784 PMC9191730

[tpj70982-bib-0084] Stanke, M. , Diekhans, M. , Baertsch, R. & Haussler, D. (2008) Using native and syntenically mapped cDNA alignments to improve *de novo* gene finding. Bioinformatics, 24, 637–644. Available from: 10.1093/bioinformatics/btn013 18218656

[tpj70982-bib-0085] Suyama, M. , Torrents, D. & Bork, P. (2006) PAL2NAL: robust conversion of protein sequence alignments into the corresponding codon alignments. Nucleic Acids Research, 34, W609–W612. Available from: 10.1093/nar/gkl315 16845082 PMC1538804

[tpj70982-bib-0086] The 100 Tomato Genome Sequencing Consortium , Aflitos, S. , Schijlen, E. , De Jong, H. , De Ridder, D. , Smit, S. et al. (2014) Exploring genetic variation in the tomato (*Solanum* section *Lycopersicon*) clade by whole‐genome sequencing. The Plant Journal, 80, 136–148. Available from: 10.1111/tpj.12616 25039268

[tpj70982-bib-0087] Vannier, T. , Leconte, J. , Seeleuthner, Y. , Mondy, S. , Pelletier, E. , Aury, J.‐M. et al. (2016) Survey of the green picoalga *Bathycoccus* genomes in the global ocean. Scientific Reports, 6, 37900. Available from: 10.1038/srep37900 27901108 PMC5128809

[tpj70982-bib-0088] Vaulot, D. , Lepère, C. , Toulza, E. , De la Iglesia, R. , Poulain, J. , Gaboyer, F. et al. (2012) Metagenomes of the picoalga *Bathycoccus* from the Chile coastal upwelling. PLoS One, 7, e39648. Available from: 10.1371/journal.pone.0039648 22745802 PMC3382182

[tpj70982-bib-0089] Walker, B.J. , Abeel, T. , Shea, T. , Priest, M. , Abouelliel, A. , Sakthikumar, S. et al. (2014) Pilon: an integrated tool for comprehensive microbial variant detection and genome assembly improvement. PLoS One, 9, e112963. Available from: 10.1371/journal.pone.0112963 25409509 PMC4237348

[tpj70982-bib-0090] Wisecaver, J.H. , Auber, R.P. , Pendleton, A.L. , Watervoort, N.F. , Fallon, T.R. , Riedling, O.L. et al. (2023) Extreme genome diversity and cryptic speciation in a harmful algal‐bloom‐forming eukaryote. Current Biology, 33, 2246–2259.e8. Available from: 10.1016/j.cub.2023.05.003 37224809 PMC10247466

[tpj70982-bib-0091] Worden, A.Z. , Nolan, J.K. & Palenik, B. (2004) Assessing the dynamics and ecology of marine picophytoplankton: the importance of the eukaryotic component. Limnology and Oceanography, 49, 168–179. Available from: 10.4319/lo.2004.49.1.0168

[tpj70982-bib-0092] Xu, M. , Guo, L. , Gu, S. , Wang, O. , Zhang, R. , Peters, B.A. et al. (2020) TGS‐GapCloser: a fast and accurate gap closer for large genomes with low coverage of error‐prone long reads. GigaScience, 9, giaa094. Available from: 10.1093/gigascience/giaa094 32893860 PMC7476103

[tpj70982-bib-0093] Xu, Y. , Leung, S.K.K. , Li, T.M.W. & Yung, C.C.M. (2024) Hidden genomic diversity drives niche partitioning in a cosmopolitan eukaryotic picophytoplankton. The ISME Journal, 18(1), wrae163. Available from: 10.1093/ismejo/wrae163 39141834 PMC11409870

[tpj70982-bib-0094] Yau, S. , Caravello, G. , Fonvieille, N. , Desgranges, É. , Moreau, H. , Moreau, H. et al. (2018) Rapidity of genomic adaptations to Prasinovirus infection in a marine microalga. Viruses, 10, 441. Available from: 10.3390/v10080441 30126244 PMC6116238

[tpj70982-bib-0095] Yau, S. , Hemon, C. , Derelle, E. , Moreau, H. , Piganeau, G. & Grimsley, N. (2016) A viral immunity chromosome in the marine picoeukaryote, *Ostreococcus tauri* . PLoS Pathogens, 12, e1005965. Available from: 10.1371/journal.ppat.1005965 27788272 PMC5082852

[tpj70982-bib-0096] Yau, S. , Krasovec, M. , Benites, L.F. , Rombauts, S. , Groussin, M. , Vancaester, E. et al. (2020) Virus‐host coexistence in phytoplankton through the genomic lens. Science Advances, 6, eaay2587. Available from: 10.1126/sciadv.aay2587 32270031 PMC7112755

[tpj70982-bib-0097] Yung, C.C.M. , Redondo, E.R. , Sanchez, F. , Sheree, Y. & Piganeau, G. (2022) Diversity and evolution of Mamiellophyceae: early‐diverging phytoplanktonic green algae containing many cosmopolitan species. Journal of Marine Science and Engineering, 10, 240. Available from: 10.3390/jmse10020240

