# Supplementary material for: Biological and genomic resources for the cosmopolitan phytoplankton Bathycoccus: insights into genetic diversity and function of outlier chromosomes
Source: Plant J. 2026 Jun 7;126(5):e70982. doi: 10.1111/tpj.70982 (PMC13242883; doi:10.1111/tpj.70982)
Supplement: Supplementary file 1 — Data S1. Quast output for all assembled genomes against the reference genome. [file TPJ-126-0-s007.zip › tpj70982-sup-0001-Supinfo1.html]

|  |
| --- |
| QUAST **Quality Assessment Tool for Genome Assemblies** by CAB |

Loading...

Aligned to
""

Combined reference
Estimated reference size:
 bp
|
 bp
|
 references
|
 fragments
|
 % G+C
|
 chromosomes
  
 reads
|
% mapped
|
% properly paired
  
 genomic features
|
 operons

Unfortunately, JavaScript in your
browser is disabled or is not supported.
We need JavaScript to build report and plots.

Worst
Median
Best

Show heatmap

|  |  |
| --- | --- |
|  |  |
|  | |  |
| Contigs are ordered from largest (contig #1) to smallest.  FRCurve: Y is the total number of aligned bases divided by the reference length, in the contigs having the total number of  at most X.  Contigs are broken into nonoverlapping 100 bp windows. Plot shows number of windows for each GC percentage.  Plot shows number of contigs with GC percentage in a certain range. | | |

{"date":"08 September 2023, Friday, 16:54:26","assembliesNames":["A818","B218","B518","C218","E318","H718","D119","H44","A727","A827","RCC5417","RCC1613","RCC685","RCC1615","RCC1868","RCC4222","G11","C2","G2","E2","A8","B8","A1","C3","G5","G8","RCC4752","RCC716"],"referenceName":"Reference","order":[0,1,2,3,4,5,6,7,8,9,10,11,12,13,14,15,16,17,18,19,20,21,22,23,24,25,26,27],"report":[["Genome statistics",[{"metricName":"Genome fraction (%)","quality":"More is better","values":["79.455","80.229","83.086","80.162","82.248","81.903","81.656","83.156","80.655","80.765","94.405","96.316","96.330","95.934","97.280","99.738","94.958","97.426","97.202","97.271","95.547","97.226","95.334","0.653","0.633","0.592","96.355","0.373"],"isMain":true},{"metricName":"Duplication ratio","quality":"Less is better","values":["1.005","1.009","1.002","1.015","1.006","1.003","1.010","1.006","1.008","1.008","1.003","1.008","1.013","1.009","1.004","1.004","1.005","1.003","1.003","1.003","1.004","1.004","1.004","1.008","1.007","1.018","1.017","1.013"],"isMain":true},{"metricName":"Largest alignment","quality":"More is better","values":[493400,364389,496851,320809,480703,480720,479849,496778,496170,496168,463425,376128,375904,444906,823228,1122352,952289,952459,951914,788209,951813,953080,895946,8819,8819,8818,452382,8817],"isMain":true},{"metricName":"Total aligned length","quality":"More is better","values":[12003258,12175057,12524440,12237194,12446604,12355233,12399703,12576992,12231743,12239348,14240820,14604170,14681364,14551969,14685151,15060213,14353207,14699137,14667268,14676278,14426297,14674285,14393920,98918,95794,90720,14734622,56769],"isMain":true},{"metricName":"NG50","quality":"More is better","values":[1007122,984013,988276,898195,988727,989015,989737,998368,1001997,935865,986735,1001351,1007186,964497,984466,963931,972493,988013,991018,976262,989488,990352,986654,981089,968475,987998,972560,952936],"isMain":false},{"metricName":"NG90","quality":"More is better","values":[546013,721875,531826,686562,635674,612301,657160,617914,725812,597613,521692,612816,746082,647879,575062,523808,529393,527209,523127,528058,523849,523073,524588,538463,538424,561658,542641,566191],"isMain":false},{"metricName":"auNG","quality":"More is better","values":["953516.9","988328.2","932228.3","937902.8","969092.3","941496.3","964595.5","941904.5","960023.3","953316.6","922061.1","935580.0","978609.9","916006.3","924346.0","905468.9","915535.7","904691.7","896936.4","902006.4","920810.4","903621.8","918869.6","920433.0","924735.8","963169.0","922711.3","919768.6"],"isMain":false},{"metricName":"NA50","quality":"More is better","values":[81973,70032,90779,73528,78006,78950,76037,83777,80926,83086,127637,162082,90271,186244,242185,854688,195828,250326,255519,261484,255931,240614,250220,null,null,null,167150,null],"isMain":false},{"metricName":"NA90","quality":"More is better","values":[null,null,null,null,null,null,null,null,null,null,12248,23673,7610,19552,24400,294480,24403,56488,53890,53888,27232,55376,27400,null,null,null,26644,null],"isMain":false},{"metricName":"auNA","quality":"More is better","values":["102152.8","97937.4","117913.3","94954.1","109932.1","112546.5","106601.6","108834.6","103177.9","104145.4","148611.3","170611.2","103792.0","190102.9","281299.2","737013.3","329028.3","365431.3","367113.3","314300.1","316565.5","337706.2","326964.4","18.0","17.9","19.4","175237.0","14.8"],"isMain":false},{"metricName":"NGA50","quality":"More is better","values":[83472,75827,95417,81871,82632,84495,80619,87836,86328,86329,139208,165147,96915,189523,265303,854688,200168,250326,255519,273107,273222,250373,250220,null,null,null,167956,null],"isMain":true},{"metricName":"NGA90","quality":"More is better","values":[null,null,null,null,null,null,null,null,null,null,21623,30126,19737,28890,52653,296126,31985,59945,59948,55124,38116,59962,36661,null,null,null,33792,null],"isMain":false},{"metricName":"auNGA","quality":"More is better","values":["105730.2","102021.8","120380.1","99281.2","115062.2","115266.1","111298.0","112084.3","107236.8","107335.7","151612.9","175196.3","109744.0","194277.2","292709.5","742809.8","334691.7","367784.3","369226.9","315634.7","323573.5","340531.0","332557.9","18.2","18.1","20.0","178923.4","15.0"],"isMain":false},{"metricName":"LG50","quality":"Less is better","values":[7,7,7,8,7,7,7,7,7,7,7,7,7,7,7,7,7,7,7,7,7,7,7,7,7,7,7,7],"isMain":false},{"metricName":"LG90","quality":"Less is better","values":[15,14,15,15,15,15,15,15,15,15,15,15,14,15,15,15,15,15,15,15,15,15,15,15,15,15,15,15],"isMain":false},{"metricName":"LA50","quality":"Less is better","values":[54,56,48,56,53,51,55,53,54,53,37,31,56,29,20,8,18,16,16,18,18,18,17,null,null,null,31,null],"isMain":false},{"metricName":"LA90","quality":"Less is better","values":[null,null,null,null,null,null,null,null,null,null,143,121,238,106,79,21,85,63,60,66,77,65,81,null,null,null,113,null],"isMain":false},{"metricName":"LGA50","quality":"Less is better","values":[51,52,46,52,49,48,51,50,50,50,36,30,51,28,19,8,17,16,16,17,17,17,17,null,null,null,30,null],"isMain":true},{"metricName":"LGA90","quality":"Less is better","values":[null,null,null,null,null,null,null,null,null,null,127,107,177,94,63,20,77,61,58,65,67,63,73,null,null,null,104,null],"isMain":false}]],["Reads mapping",[]],["Misassemblies",[{"metricName":"# misassemblies","quality":"Less is better","values":[252,281,246,281,270,254,273,267,264,260,227,235,331,202,147,27,158,150,142,156,146,152,150,0,0,0,223,0],"isMain":true},{"metricName":" # relocations","quality":"Less is better","values":[168,168,169,169,175,169,179,176,164,164,145,134,233,132,87,23,91,80,78,82,75,83,73,0,0,0,140,0],"isMain":false},{"metricName":" # translocations","quality":"Less is better","values":[68,92,60,94,75,67,76,70,82,78,57,84,79,55,43,4,54,58,50,60,57,52,65,0,0,0,63,0],"isMain":false},{"metricName":" # inversions","quality":"Less is better","values":[16,21,17,18,20,18,18,21,18,18,25,17,19,15,17,0,13,12,14,14,14,17,12,0,0,0,20,0],"isMain":false},{"metricName":"# misassembled contigs","quality":"Less is better","values":[18,18,19,18,19,19,18,19,18,18,19,19,19,19,18,12,18,18,18,19,18,19,17,0,0,0,19,0],"isMain":false},{"metricName":"Misassembled contigs length","quality":"Less is better","values":[14679871,14899357,15194250,14770666,15551262,15305142,15160094,15307805,14665045,14605667,15111818,15255254,15680497,15138493,15028623,8193210,13479431,14078061,14207376,14943470,14366905,14985088,13456226,0,0,0,15228777,0],"isMain":true},{"metricName":"# local misassemblies","quality":"Less is better","values":[649,628,658,637,663,666,654,670,636,636,377,132,168,135,104,22,76,76,60,70,67,78,73,0,0,0,134,0],"isMain":false},{"metricName":"# scaffold gap ext. mis.","quality":"Less is better","values":[9,0,5,1,3,5,1,1,1,2,1,1,0,4,0,0,0,0,0,0,0,0,0,0,0,0,1,0],"isMain":false},{"metricName":"# scaffold gap loc. mis.","quality":"Less is better","values":[26,1,9,0,5,6,1,2,0,0,1,0,0,11,0,0,0,0,0,0,0,0,0,0,0,0,1,0],"isMain":false},{"metricName":"# unaligned mis. contigs","quality":"Less is better","values":[2,2,1,2,1,1,2,1,2,2,1,1,1,1,1,0,2,1,1,1,2,1,2,17,15,13,1,17],"isMain":false}]],["Unaligned",[{"metricName":"# fully unaligned contigs","quality":"Less is better","values":[0,0,0,0,0,0,0,0,0,0,0,0,0,0,1,0,0,0,0,0,0,0,0,2,2,2,0,1],"isMain":false},{"metricName":"Fully unaligned length","quality":"Less is better","values":[0,0,0,0,0,0,0,0,0,0,0,0,0,0,431628,0,0,0,0,0,0,0,0,347331,346411,349409,0,33697],"isMain":false},{"metricName":"# partially unaligned contigs","quality":"Less is better","values":[20,20,20,20,20,20,20,20,20,20,20,20,20,20,19,15,20,20,20,20,20,20,20,19,19,19,20,20],"isMain":false},{"metricName":"Partially unaligned length","quality":"Less is better","values":[3382511,3444491,2743097,3520031,3209796,2984335,3192500,2943984,3432799,3293454,1113998,871837,1249301,815643,565219,121689,978442,468303,489967,458341,977233,522553,933552,14834429,14678115,15168337,650244,15205794],"isMain":false}]],["Mismatches",[{"metricName":"# mismatches per 100 kbp","quality":"Less is better","values":["2140.77","2168.66","2104.91","2163.63","2112.30","2111.45","2118.85","2116.04","2154.25","2154.83","1551.40","758.76","787.49","759.79","531.98","10.29","435.52","392.51","381.55","402.33","404.80","372.43","415.89","3055.06","3038.81","2864.86","753.26","3059.77"],"isMain":true},{"metricName":"# mismatches","quality":"Less is better","values":[256962,264035,263628,264767,262909,260875,262731,266134,263502,263737,220932,110811,115614,110564,78122,1550,62511,57696,55963,59047,58398,54652,59863,3022,2911,2599,110990,1737],"isMain":false},{"metricName":"# indels per 100 kbp","quality":"Less is better","values":["646.93","637.80","636.82","631.83","629.64","632.19","621.66","628.65","639.15","639.34","495.22","234.93","271.64","264.62","189.48","9.06","136.82","124.10","123.13","127.94","126.69","117.59","129.10","237.57","238.01","234.79","244.32","331.17"],"isMain":true},{"metricName":"# indels","quality":"Less is better","values":[77653,77653,79758,77318,78369,78109,77084,79065,78179,78251,70524,34310,39880,38508,27826,1365,19638,18242,18060,18777,18277,17255,18582,235,228,213,36000,188],"isMain":false},{"metricName":" # indels (<= 5 bp)","quality":"Less is better","values":[65060,64934,66664,64620,65574,65311,64354,66094,65325,65400,59256,28981,33741,33275,23874,1301,16545,15393,15259,15812,15434,14542,15675,219,212,197,30627,172],"isMain":false},{"metricName":" # indels (> 5 bp)","quality":"Less is better","values":[12593,12719,13094,12698,12795,12798,12730,12971,12854,12851,11268,5329,6139,5233,3952,64,3093,2849,2801,2965,2843,2713,2907,16,16,16,5373,16],"isMain":false},{"metricName":"Indels length","quality":"Less is better","values":[294583,295282,304272,294818,300142,297980,295067,301887,298066,298222,280720,146055,167611,150536,111975,8791,83820,79074,77219,80216,79046,75120,80205,518,496,486,150237,439],"isMain":false},{"metricName":"# N's per 100 kbp","quality":"Less is better","values":["1363.74","502.24","788.41","2.54","752.10","635.32","907.16","4.79","0.64","3.86","138.72","0.67","0.00","219.53","1.26","0.00","0.00","0.66","0.00","0.66","0.00","0.00","1.30","62.31","480.70","3.20","3.25","162.23"],"isMain":true},{"metricName":"# N's","quality":"Less is better","values":[212774,78866,121334,400,118664,98084,142772,743,100,600,21334,104,0,33820,197,0,0,100,0,100,0,0,200,9528,73034,500,500,24855],"isMain":false}]],["Statistics without reference",[{"metricName":"# contigs","quality":"Equal","values":[21,21,21,21,21,21,21,21,21,21,21,21,21,21,21,21,21,21,21,21,21,21,21,21,21,21,21,21],"isMain":true},{"metricName":"# contigs (>= 0 bp)","quality":"Equal","values":[21,21,21,21,21,21,21,21,21,21,21,21,21,21,21,21,21,21,21,21,21,21,21,21,21,21,21,21],"isMain":false},{"metricName":"# contigs (>= 1000 bp)","quality":"Equal","values":[21,21,21,21,21,21,21,21,21,21,21,21,21,21,21,21,21,21,21,21,21,21,21,21,21,21,21,21],"isMain":false},{"metricName":"# contigs (>= 5000 bp)","quality":"Equal","values":[21,21,21,21,21,21,21,21,21,21,21,21,21,21,21,21,21,21,21,21,21,21,21,21,21,21,21,21],"isMain":false},{"metricName":"# contigs (>= 10000 bp)","quality":"Equal","values":[21,21,21,21,21,21,21,21,21,21,21,21,21,21,21,21,21,21,21,21,21,21,21,21,21,21,21,21],"isMain":false},{"metricName":"# contigs (>= 25000 bp)","quality":"Equal","values":[21,21,21,21,21,21,21,21,21,21,21,21,21,21,21,21,21,21,21,21,21,21,21,21,21,21,21,21],"isMain":false},{"metricName":"# contigs (>= 50000 bp)","quality":"Equal","values":[20,19,20,20,20,20,20,20,20,20,20,20,20,20,20,20,20,20,20,20,20,20,20,20,20,20,20,20],"isMain":false},{"metricName":"Largest contig","quality":"More is better","values":[1379102,1393337,1436564,1418329,1441520,1405785,1448332,1430540,1368327,1369564,1387673,1368800,1399097,1380033,1354659,1359012,1368101,1404025,1351263,1393237,1359092,1350358,1385962,1384637,1453669,1384562,1359563,1381209],"isMain":true},{"metricName":"Total length","quality":"More is better","values":[15602224,15702977,15389679,15761254,15777776,15438582,15738432,15524416,15667328,15536093,15378783,15479435,15938751,15405326,15685779,15192876,15333787,15171383,15161107,15138326,15408027,15200413,15332201,15290205,15193353,15608965,15391428,15321115],"isMain":true},{"metricName":"Total length (>= 0 bp)","quality":"More is better","values":[15602224,15702977,15389679,15761254,15777776,15438582,15738432,15524416,15667328,15536093,15378783,15479435,15938751,15405326,15685779,15192876,15333787,15171383,15161107,15138326,15408027,15200413,15332201,15290205,15193353,15608965,15391428,15321115],"isMain":false},{"metricName":"Total length (>= 1000 bp)","quality":"More is better","values":[15602224,15702977,15389679,15761254,15777776,15438582,15738432,15524416,15667328,15536093,15378783,15479435,15938751,15405326,15685779,15192876,15333787,15171383,15161107,15138326,15408027,15200413,15332201,15290205,15193353,15608965,15391428,15321115],"isMain":true},{"metricName":"Total length (>= 5000 bp)","quality":"More is better","values":[15602224,15702977,15389679,15761254,15777776,15438582,15738432,15524416,15667328,15536093,15378783,15479435,15938751,15405326,15685779,15192876,15333787,15171383,15161107,15138326,15408027,15200413,15332201,15290205,15193353,15608965,15391428,15321115],"isMain":false},{"metricName":"Total length (>= 10000 bp)","quality":"More is better","values":[15602224,15702977,15389679,15761254,15777776,15438582,15738432,15524416,15667328,15536093,15378783,15479435,15938751,15405326,15685779,15192876,15333787,15171383,15161107,15138326,15408027,15200413,15332201,15290205,15193353,15608965,15391428,15321115],"isMain":true},{"metricName":"Total length (>= 25000 bp)","quality":"More is better","values":[15602224,15702977,15389679,15761254,15777776,15438582,15738432,15524416,15667328,15536093,15378783,15479435,15938751,15405326,15685779,15192876,15333787,15171383,15161107,15138326,15408027,15200413,15332201,15290205,15193353,15608965,15391428,15321115],"isMain":false},{"metricName":"Total length (>= 50000 bp)","quality":"More is better","values":[15568329,15621232,15355822,15727165,15743909,15395041,15704573,15480873,15633435,15502199,15344884,15445624,15904851,15371461,15651959,15159425,15290172,15137482,15127207,15104429,15374156,15166517,15298304,15256630,15159777,15575398,15357634,15287418],"isMain":true},{"metricName":"N50","quality":"More is better","values":[933280,984013,988276,898195,958919,989015,920314,998368,935817,935865,985652,990084,945509,917895,943349,936923,923380,988013,925398,976262,945596,990352,924918,981089,968475,987998,932534,945702],"isMain":false},{"metricName":"N90","quality":"More is better","values":[534336,629083,527350,660540,586990,612301,506638,506344,725812,597613,518210,495993,522025,588400,513791,489133,501153,501400,495157,499512,512960,504031,501213,524041,538424,547196,491771,532550],"isMain":false},{"metricName":"auN","quality":"More is better","values":["921254.4","948761.2","913125.4","897025.5","925885.1","919282.4","923892.6","914596.1","923686.5","924981.5","903806.5","911094.7","925535.4","896324.6","888313.4","898403.2","900043.7","898903.7","891802.1","898192.7","900867.5","896125.9","903414.6","907437.2","917490.9","930178.1","903700.8","904952.8"],"isMain":false},{"metricName":"L50","quality":"Less is better","values":[8,7,7,8,8,7,8,7,8,7,8,8,8,8,8,8,8,7,8,7,8,7,8,7,7,7,8,8],"isMain":false},{"metricName":"L90","quality":"Less is better","values":[16,15,16,16,16,15,16,16,15,15,16,16,16,16,16,16,16,16,16,16,16,16,16,16,15,16,16,16],"isMain":false},{"metricName":"GC (%)","quality":"Equal","values":["47.65","47.62","47.74","47.56","47.55","47.69","47.62","47.67","47.61","47.65","47.81","47.91","48.06","47.87","47.72","48.04","47.94","48.00","47.99","48.01","47.92","48.01","47.92","48.35","48.44","48.48","47.90","47.66"],"isMain":false}]],["K-mer-based statistics",[]],["Predicted genes",[]],["Similarity statistics",[{"metricName":"# similar correct contigs","quality":"Equal","values":[1,1,1,1,1,0,1,1,1,1,1,1,1,1,1,1,0,1,1,1,1,1,1,0,0,0,1,0],"isMain":false},{"metricName":"# similar misassembled blocks","quality":"Equal","values":[9,11,8,13,11,11,11,13,12,12,9,9,11,11,7,6,8,8,9,9,8,8,9,0,0,0,12,0],"isMain":false}]],["Reference statistics",[{"metricName":"Reference length","quality":"Equal","values":[15074320,15074320,15074320,15074320,15074320,15074320,15074320,15074320,15074320,15074320,15074320,15074320,15074320,15074320,15074320,15074320,15074320,15074320,15074320,15074320,15074320,15074320,15074320,15074320,15074320,15074320,15074320,15074320],"isMain":false},{"metricName":"Reference fragments","quality":"Equal","values":[21,21,21,21,21,21,21,21,21,21,21,21,21,21,21,21,21,21,21,21,21,21,21,21,21,21,21,21],"isMain":false},{"metricName":"Reference GC (%)","quality":"Equal","values":["48.02","48.02","48.02","48.02","48.02","48.02","48.02","48.02","48.02","48.02","48.02","48.02","48.02","48.02","48.02","48.02","48.02","48.02","48.02","48.02","48.02","48.02","48.02","48.02","48.02","48.02","48.02","48.02"],"isMain":false}]]],"subreferences":[],"subreports":[],"minContig":500}

{{ qualities }}

{{ mainMetrics }}

{"filenames":["A818","B218","B518","C218","E318","H718","D119","H44","A727","A827","RCC5417","RCC1613","RCC685","RCC1615","RCC1868","RCC4222","G11","C2","G2","E2","A8","B8","A1","C3","G5","G8","RCC4752","RCC716"],"lists\_of\_lengths":[[1379102,1222833,1107062,1031096,1023375,1010111,1007122,933280,905029,807368,777067,774605,747657,733858,546013,534336,502463,317354,140801,67797,33895],[1393337,1351320,1096016,1094624,1088367,1033768,984013,946052,883539,791567,774784,768682,737726,721875,629083,514227,427001,317272,67979,47970,33775],[1436564,1136833,1073283,1049310,1043668,1038315,988276,917852,888554,783349,777642,738945,715053,688818,531826,527350,473020,317601,161572,67991,33857],[1418329,1088380,1087748,1027502,988574,944000,942503,898195,887750,813897,783643,743143,729845,717342,686562,660540,629372,317231,226654,135955,34089],[1441520,1160826,1100557,1076254,1046064,1005551,988727,958919,887982,779070,776861,772900,752066,741690,635674,586990,455038,316604,192647,67969,33867],[1405785,1193529,1083450,1045275,1034781,999314,989015,950931,888615,783923,781928,777094,725153,692187,612301,502873,453827,317192,89899,67969,43541],[1448332,1149162,1132280,1080573,1045474,1008355,989737,920314,888223,809000,776907,768865,750795,679518,657160,506638,475232,314478,230001,73529,33859],[1430540,1174667,1072677,1042730,1027144,1024272,998368,921926,887734,790440,785317,754237,733045,657272,617914,506344,473691,341526,173068,67961,43543],[1368327,1226042,1088739,1050855,1042420,1032508,1001997,935817,887771,819461,787705,745296,739700,737386,725812,509291,320350,317389,228690,67879,33893],[1369564,1292280,1088825,1050179,1044430,1001784,935865,930292,887823,818713,798075,787403,725809,711730,597613,502163,475900,317415,98457,67879,33894],[1387673,1146138,1096451,1016660,1013628,992004,986735,985652,911957,803017,748028,737724,734935,641580,521692,518210,482895,318881,233066,67958,33899],[1368800,1215633,1094603,1017758,1013967,1006628,1001351,990084,897590,788643,731444,725711,716370,693896,612816,495993,488639,327379,190370,67949,33811],[1399097,1188627,1132240,1092770,1019818,1017095,1007186,945509,925960,825711,822848,755331,749838,746082,567357,522025,485845,341203,224354,135955,33900],[1380033,1146277,1082997,1014591,1010103,971212,964497,917895,901748,785066,778859,729759,712812,666013,647879,588400,443152,329241,232968,67959,33865],[1354659,1124202,1081319,1016380,1004405,996712,984466,943349,896638,806733,770071,748153,726451,714867,575062,513791,451519,431628,319846,191708,33820],[1359012,1122352,1076186,1042483,1016583,995001,963931,936923,896176,796427,781469,775097,715892,669509,523808,489133,469966,306851,150971,71655,33451],[1368101,1124881,1078754,1026279,1025217,1000880,972493,923380,898711,814716,769802,751054,747011,721518,529393,501153,468591,310741,185843,71654,43615],[1404025,1125402,1079185,1023053,1005769,990880,988013,925144,898896,794956,746850,720742,717687,667499,527209,501400,474446,314146,160525,71655,33901],[1351263,1125799,1078536,1016268,1012529,993922,991018,925398,898043,793604,751820,747121,715890,647427,523127,495157,475408,309281,203941,71655,33900],[1393237,1125820,1086541,1021435,1013856,1001805,976262,927198,891361,795128,751318,720376,717683,646622,528058,499512,471221,308069,160959,67968,33897],[1359092,1131285,1079247,1016987,1008950,1006439,989488,945596,894004,791990,781878,770859,764371,720863,523849,512960,474879,308071,225373,67975,33871],[1350358,1125907,1079905,1040521,1022495,993645,990352,925420,897381,805861,751911,738719,719367,662588,523073,504031,477181,308408,181429,67965,33896],[1385962,1126251,1082459,1029491,1021119,993816,986654,924918,899716,813258,766306,760873,727240,719832,524588,501213,475356,309801,181489,67962,33897],[1384637,1137532,1105944,1088545,1018223,1003726,981089,961760,937956,818177,738401,738226,670243,566192,538463,524041,447370,313756,213903,68446,33575],[1453669,1135298,1106368,1088630,1018847,1003709,968475,963500,934855,826295,738219,677471,658877,596935,538424,514970,440498,312835,113456,68446,33576],[1384562,1313088,1101029,1091235,1049538,1016864,987998,953681,938278,840875,749994,729164,642554,633099,561658,547196,440184,315842,210109,68450,33567],[1359563,1183198,1121013,1015136,1003867,981534,972560,932534,891936,792914,785531,763076,736007,714114,542641,491771,488967,316515,135900,128857,33794],[1381209,1228988,1125243,1025998,989756,956288,952936,945702,885718,815487,737246,714434,686990,663052,566191,532550,458563,423380,129714,67973,33697]]}

{"filenames":["A818","B218","B518","C218","E318","H718","D119","H44","A727","A827","RCC5417","RCC1613","RCC685","RCC1615","RCC1868","RCC4222","G11","C2","G2","E2","A8","B8","A1","C3","G5","G8","RCC4752","RCC716"],"assemblies\_lengths":[15602224,15702977,15389679,15761254,15777776,15438582,15738432,15524416,15667328,15536093,15378783,15479435,15938751,15405326,15685779,15192876,15333787,15171383,15161107,15138326,15408027,15200413,15332201,15290205,15193353,15608965,15391428,15321115]}

{"reflen":[1352724,1122692,1091008,1037991,1019276,989707,955652,937610,895536,794368,741603,712459,708035,663424,519835,494108,465570,310170,146238,72700,43614]}

{"tickX":1}

{"coord\_x":[[0.0,1e-10,8.83913729222193,8.83913729232193,16.676693015047086,16.676693015147084,23.772232727847005,23.772232727947003,30.380880315524248,30.380880315624246,36.940041368461316,36.94004136856132,43.414188900249094,43.414188900349096,49.869178906802006,49.86917890690201,55.85089023205922,55.85089023215922,61.65153121760077,61.65153121770077,66.82622938883584,66.82622938893584,71.80671806788571,71.80671806798571,76.77142694528678,76.77142694538678,81.56341685646866,81.56341685656867,86.26696424817385,86.26696424827385,89.76654866639525,89.76654866649525,93.19129119028159,93.19129119038159,96.4117487353085,96.41174873540851,98.44577926839148,98.44577926849148,99.34822112539854,99.34822112549854,99.78275533026574,99.78275533036575,100.0,100.0000000001],[0.0,1e-10,8.873075468428693,8.873075468528693,17.478577469737107,17.478577469837106,24.458247630369705,24.458247630469703,31.429053229842978,31.429053229942976,38.36001288163384,38.360012881733844,44.943274132032414,44.943274132132416,51.20968463495807,51.209684635058075,57.23435116793459,57.23435116803459,62.86092121258281,62.86092121268281,67.9017933987931,67.9017933988931,72.83578776177282,72.83578776187282,77.73092325104979,77.73092325114979,82.42892414603932,82.42892414613932,87.0259823981147,87.02598239821471,91.03212085198876,91.03212085208877,94.3068311187108,94.3068311188108,97.02606709543038,97.02606709553038,99.0465247449576,99.0465247450576,99.47942991956239,99.4794299196624,99.78491339572108,99.78491339582108,100.0,100.0000000001],[0.0,1e-10,9.334593658516205,9.334593658616205,16.721576843805515,16.721576843905513,23.69562094180132,23.695620941901318,30.513891810219043,30.51389181031904,37.29550174503315,37.29550174513315,44.04232862816697,44.04232862826697,50.46400902838844,50.46400902848844,56.42808404255865,56.428084042658654,62.201784715587635,62.20178471568764,67.2918778877714,67.29187788787141,72.34488776536534,72.34488776546534,77.1464499032111,77.14644990331111,81.79276513824622,81.79276513834623,86.26860898138291,86.26860898148291,89.72434057916348,89.72434057926348,93.15098774964702,93.15098774974702,96.22460611426658,96.22460611436658,98.28833336939647,98.28833336949647,99.33820581962756,99.33820581972756,99.78000190907166,99.78000190917166,100.0,100.0000000001],[0.0,1e-10,8.998833468453716,8.998833468553716,15.904248481751516,15.904248481851516,22.80565366182158,22.80565366192158,29.32481768265393,29.324817682753928,35.59699627961075,35.59699627971075,41.5863674299012,41.586367430001204,47.56624060496709,47.566240605067094,53.26499401633905,53.264994016439054,58.89747732001527,58.89747732011527,64.0613875012737,64.0613875013737,69.0333459507727,69.0333459508727,73.74834515070945,73.74834515080946,78.3789728913702,78.3789728914702,82.93027318765373,82.93027318775373,87.28628445426995,87.28628445436995,91.4771946445378,91.4771946446378,95.47035407208081,95.47035407218081,97.48308097820136,97.48308097830136,98.92112645351696,98.92112645361696,99.78371644794252,99.78371644804253,100.0,100.0000000001],[0.0,1e-10,9.136395395650185,9.136395395750185,16.493744111971168,16.493744112071166,23.469106165533088,23.469106165633086,30.29043510314762,30.290435103247617,36.9204189487796,36.9204189488796,43.29363023026819,43.293630230368194,49.56021051382654,49.56021051392654,55.6378668324357,55.6378668325357,61.26592239616027,61.26592239626027,66.20369055816232,66.20369055826232,71.12745801436147,71.12745801446147,76.02612053815443,76.02612053825443,80.79273656819566,80.79273656829567,85.49358921054527,85.49358921064527,89.52250938281796,89.52250938291796,93.24286895694298,93.24286895704299,96.12691294387751,96.12691294397752,98.13355824040093,98.13355824050093,99.3545604906547,99.35456049075471,99.78534997581409,99.78534997591409,100.0,100.0000000001],[0.0,1e-10,9.105661387813985,9.105661387913985,16.83648148515194,16.836481485251937,23.8542892086851,23.854289208785097,30.624826813757895,30.624826813857894,37.32739185502917,37.32739185512917,43.800227248849666,43.80022724894967,50.206353148236026,50.20635314833603,56.365798361533464,56.365798361633466,62.12160546868877,62.12160546878877,67.19929330297303,67.19929330307303,72.26405896603717,72.26405896613717,77.29751346334787,77.29751346344787,81.9945316221399,81.9945316222399,86.47801980777768,86.47801980787769,90.4440640986329,90.4440640987329,93.70131272418672,93.70131272428672,96.64087673336839,96.64087673346839,98.69541775274439,98.69541775284439,99.27771864022228,99.27771864032228,99.71797280346084,99.71797280356084,100.0,100.0000000001],[0.0,1e-10,9.202517760346138,9.202517760446138,16.504147300061405,16.504147300161403,23.698510753803173,23.69851075390317,30.56433449024655,30.56433449034655,37.20714363413077,37.207143634230775,43.61410336175802,43.61410336185802,49.9027666796794,49.902766679779404,55.750325064148704,55.750325064248706,61.39398130639698,61.39398130649698,66.53426465863944,66.53426465873945,71.47063316091463,71.47063316101463,76.35590381557705,76.35590381567705,81.12635998300212,81.12635998310212,85.44393113621484,85.44393113631484,89.61944239426138,89.61944239436139,92.83855596288119,92.83855596298119,95.8581197923656,95.8581197924656,97.85627310268265,97.85627310278265,99.31767027363335,99.31767027373336,99.78486421010683,99.78486421020683,100.0,100.0000000001],[0.0,1e-10,9.214774971245296,9.214774971345296,16.781352676970265,16.781352677070263,23.69096525112442,23.69096525122442,30.40767523879803,30.40767523889803,37.02398853522091,37.02398853532091,43.62180194089105,43.62180194099105,50.05275560768276,50.052755607782764,55.99131071983642,55.99131071993642,61.70961922174721,61.70961922184721,66.80121171707844,66.80121171717845,71.85980458137685,71.85980458147685,76.71819667805862,76.71819667815862,81.44008122431143,81.44008122441143,85.67387655677354,85.67387655687354,89.65414866491597,89.65414866501597,92.91574639587087,92.91574639597087,95.96701093297165,95.96701093307165,98.16693909774126,98.16693909784127,99.28175075957769,99.28175075967769,99.71951923988638,99.71951923998638,100.0,100.0000000001],[0.0,1e-10,8.733633456834504,8.733633456934504,16.559103122115015,16.559103122215014,23.508207653532242,23.50820765363224,30.215509626146844,30.215509626246842,36.86897344588688,36.86897344598688,43.459171851128666,43.45917185122867,49.85462741317473,49.854627413274734,55.82767527430332,55.82767527440332,61.49405948480813,61.494059484908135,66.72444082360438,66.72444082370438,71.75213284613687,71.75213284623688,76.5091405503223,76.5091405504223,81.23043061331198,81.23043061341198,85.93695108700092,85.93695108710092,90.56959808334899,90.569598083449,93.82025448117254,93.82025448127254,95.86495540273364,95.86495540283364,97.89075712208233,97.89075712218234,99.35041890997623,99.35041891007623,99.78367083398011,99.78367083408011,100.0,100.0000000001],[0.0,1e-10,8.815369475453062,8.815369475553062,17.133290847319206,17.133290847419204,24.14164874013048,24.14164874023048,30.901256834649484,30.901256834749482,37.62386077374794,37.623860773847944,44.071968415740045,44.07196841584005,50.095780193900744,50.095780194000746,56.083720662588725,56.08372066268873,61.79830411674286,61.798304116842864,67.06805243763667,67.06805243773667,72.20496169789921,72.20496169799921,77.27317929932578,77.27317929942578,81.94493943876365,81.94493943886366,86.52607833900068,86.52607833910068,90.37268893794598,90.37268893804598,93.6049237089402,93.60492370904021,96.66811340534586,96.66811340544587,98.71119463561399,98.711194635714,99.34492539404856,99.34492539414856,99.78183704229886,99.78183704239886,100.0,100.0000000001],[0.0,1e-10,9.023295276355743,9.023295276455743,16.476017640667667,16.476017640767665,23.60565202070931,23.60565202080931,30.216448206597363,30.21644820669736,36.807528918250554,36.807528918350556,43.25800032421291,43.25800032431291,49.674210241473595,49.6742102415736,56.083377989012526,56.08337798911253,62.01334656975133,62.01334656985133,67.23493660063998,67.23493660073999,72.09896257720783,72.09896257730783,76.89598715320972,76.89598715330972,81.67487635400019,81.67487635410019,85.84672792378954,85.84672792388955,89.23901195562743,89.23901195572743,92.6086544039278,92.6086544040278,95.74866229661995,95.74866229671996,97.82217487560622,97.82217487570622,99.33767841057384,99.33767841067385,99.77957293499752,99.77957293509752,100.0,100.0000000001],[0.0,1e-10,8.842700008107531,8.842700008207531,16.695912996824497,16.695912996924495,23.76724990285498,23.76724990295498,30.342153961045735,30.342153961145733,36.89256746128008,36.89256746138008,43.39556967034004,43.39556967044004,49.86448148785792,49.864481487957924,56.26060641102211,56.26060641112211,62.059203065228154,62.059203065328155,67.1539820413342,67.1539820414342,71.87924494660173,71.87924494670173,76.5674716163736,76.5674716164736,81.19535370638528,81.19535370648528,85.67804961873608,85.67804961883608,89.63695380354645,89.63695380364645,92.841159900216,92.841159900316,95.99785780294953,95.99785780304953,98.11278641630008,98.11278641640008,99.34261166508985,99.34261166518985,99.7815747150978,99.7815747151978,100.0,100.0000000001],[0.0,1e-10,8.777958825004545,8.777958825104545,16.235425222465675,16.235425222565674,23.339118604713757,23.339118604813756,30.195176522928303,30.1951765230283,36.593532328850614,36.593532328950616,42.97480398558206,42.97480398568206,49.293906404585904,49.293906404685906,55.22604625669853,55.22604625679853,61.03553534401786,61.03553534411786,66.21606046797518,66.21606046807518,71.37862308031539,71.3786230804154,76.11758286455445,76.11758286465445,80.82207947159723,80.82207947169724,85.5030108695468,85.5030108696468,89.06261852010863,89.06261852020863,92.33781241704573,92.33781241714573,95.38601236696653,95.38601236706653,97.5267259021739,97.5267259022739,98.93432678633351,98.93432678643352,99.78731081249717,99.78731081259717,100.0,100.0000000001],[0.0,1e-10,8.95815512115745,8.95815512125745,16.398938912425482,16.39893891252548,23.428955674161003,23.428955674261,30.014931199768185,30.014931199868183,36.57177394363482,36.57177394373482,42.876165035391004,42.876165035491006,49.13696730598236,49.13696730608236,55.0952638068159,55.0952638069159,60.94874590774645,60.94874590784645,66.04481463099191,66.04481463109191,71.10059209392908,71.10059209402908,75.83764861580988,75.83764861590988,80.46469772856479,80.46469772866479,84.78796229304074,84.78796229314074,88.99351432095627,88.99351432105627,92.81297260440967,92.81297260450967,95.68958813335077,95.68958813345077,97.82677757030264,97.82677757040264,99.33903378610748,99.33903378620748,99.78017342833252,99.78017342843252,100.0,100.0000000001],[0.0,1e-10,8.636223932518748,8.636223932618748,15.803238079536884,15.803238079636884,22.696864465577388,22.696864465677386,29.176491648900573,29.17649164900057,35.57977579564267,35.57977579574267,41.934015518132696,41.9340155182327,48.2101845244664,48.2101845245664,54.22422437546774,54.22422437556774,59.94047219459104,59.94047219469104,65.08355753322803,65.08355753332803,69.99291523870124,69.99291523880125,74.76254128022586,74.76254128032586,79.39381270130097,79.39381270140098,83.95123378953637,83.95123378963638,87.61736984819179,87.61736984829179,90.89289094280878,90.89289094290878,93.77141549680127,93.77141549690127,96.52313092005186,96.52313092015186,98.56221358212429,98.56221358222429,99.78439068917139,99.78439068927139,100.0,100.0000000001],[0.0,1e-10,8.945060829825769,8.945060829925769,16.332417904286192,16.33241790438619,23.415908877292225,23.415908877392223,30.27756561693783,30.27756561703783,36.96874772097133,36.96874772107133,43.51787640470442,43.51787640480442,49.862501346025596,49.8625013461256,56.02935875998725,56.02935876008725,61.92801810532779,61.92801810542779,67.17012631446475,67.17012631456475,72.31378048501153,72.31378048511154,77.41549394597837,77.41549394607837,82.12751818681335,82.12751818691335,86.53424802519287,86.53424802529287,89.9819691808187,89.9819691809187,93.20145836772446,93.20145836782446,96.29478974224499,96.29478974234499,98.31449292418368,98.31449292428368,99.30818891696345,99.30818891706345,99.77982443876985,99.77982443886985,100.0,100.0000000001],[0.0,1e-10,8.922133847300735,8.922133847400735,16.258097233253597,16.258097233353595,23.29324125866624,23.293241258766237,29.986167148402412,29.98616714850241,36.672167156097835,36.67216715619784,43.19945229446581,43.19945229456581,49.541610301486514,49.541610301586516,55.56347561108029,55.563475611180294,61.4244608980156,61.424460898115605,66.73766891375236,66.73766891385236,71.75796820446247,71.75796820456247,76.65600154743248,76.65600154753248,81.52766827920591,81.52766827930591,86.23308123427044,86.23308123437045,89.68554212993828,89.68554213003829,92.953834561547,92.953834561647,96.0097724065164,96.0097724066164,98.03628418733089,98.0362841874309,99.24826789363907,99.24826789373907,99.71556276345824,99.71556276355824,100.0,100.0000000001],[0.0,1e-10,9.254429869709307,9.254429869809307,16.672356106229735,16.672356106329733,23.78564960096255,23.78564960106255,30.528957050257052,30.52895705035705,37.15833948691428,37.15833948701428,43.68958321070663,43.68958321080663,50.20192951427039,50.20192951437039,56.29988380096923,56.299883801069235,62.224828151790774,62.224828151890776,67.46466686656055,67.46466686666055,72.38742176636104,72.38742176646105,77.13808952025006,77.13808952035006,81.86862067881353,81.86862067891353,86.2683448173446,86.2683448174446,89.74336749655585,89.74336749665585,93.04827384556833,93.04827384566833,96.17551676073302,96.17551676083302,98.24616516503472,98.24616516513473,99.30424273119992,99.30424273129992,99.77654640977688,99.77654640987689,100.0,100.0000000001],[0.0,1e-10,8.91269351241964,8.91269351251964,16.338266064608607,16.338266064708606,23.452100166564353,23.45210016666435,30.155225472651832,30.15522547275183,36.83368899117986,36.833688991279864,43.389424004460885,43.38942400456089,49.92600474358502,49.92600474368502,56.029767483337466,56.02976748343747,61.95310144569259,61.95310144579259,67.18757409996513,67.18757410006513,72.14644682607938,72.14644682617939,77.07432577317738,77.07432577327738,81.79621052737112,81.79621052747112,86.06652535332677,86.06652535342677,89.51697920211235,89.51697920221235,92.78294784147359,92.78294784157359,95.91865554408395,95.91865554418395,97.9586187209153,97.9586187210153,99.30377775184886,99.30377775194886,99.77640155168089,99.77640155178089,100.0,100.0000000001],[0.0,1e-10,9.203375591198128,9.203375591298128,16.640261281201106,16.640261281301104,23.8176797091039,23.817679709203897,30.565024164494805,30.565024164594803,37.262303639120994,37.262303639220995,43.87997721808871,43.87997721818871,50.32892011970148,50.32892011980148,56.45375849350847,56.45375849360847,62.341866597403175,62.34186659750318,67.59428354231505,67.59428354241506,72.55730257097119,72.55730257107119,77.31592647694336,77.31592647704336,82.0567610976273,82.0567610977273,86.32818450335922,86.32818450345923,89.8164037423953,89.8164037424953,93.11605523622625,93.11605523632625,96.22882345115305,96.22882345125305,98.26385030947279,98.26385030957279,99.32710525589157,99.32710525599157,99.77608488547544,99.77608488557544,100.0,100.0000000001],[0.0,1e-10,8.82067509357298,8.82067509367298,16.162854595205474,16.162854595305472,23.16730104380009,23.16730104390009,29.76767239569349,29.76767239579349,36.31588262403746,36.31588262413746,42.84779615196676,42.84779615206676,49.26969559438077,49.26969559448077,55.40673053078113,55.406730530881134,61.208927009278995,61.208927009378996,66.34904001660952,66.34904001670952,71.42352489387513,71.42352489397513,76.4264950989507,76.4264950990507,81.3873573819672,81.38735738206721,86.06584736644088,86.06584736654088,89.4656921356641,89.4656921357641,92.79486594876813,92.79486594886814,95.87688936422555,95.87688936432555,97.87630823855643,97.87630823865643,99.33900686960115,99.33900686970115,99.78017302280169,99.78017302290169,100.0,100.0000000001],[0.0,1e-10,8.883692831240836,8.883692831340836,16.290774467772685,16.290774467872684,23.395219590415078,23.395219590515076,30.240566489871032,30.24056648997103,36.967324506248616,36.96732450634862,43.504285048044416,43.50428504814442,50.019581704786575,50.019581704886576,56.107705757731715,56.10770575783172,62.011367717442944,62.011367717542946,67.31294077338556,67.31294077348556,72.25958926247596,72.25958926257596,77.11945063597943,77.11945063607943,81.85199967921925,81.85199967931925,86.21101281919117,86.21101281929117,89.65218905565263,89.65218905575263,92.96809238012152,92.96809238022152,96.10735576724133,96.10735576734133,98.13630063867343,98.13630063877343,99.32988004996969,99.32988005006969,99.7770060589801,99.77700605908011,100.0,100.0000000001],[0.0,1e-10,9.03955015982376,9.03955015992376,16.38520783806578,16.38520783816578,23.445244423811037,23.445244423911035,30.159812019161503,30.1598120192615,36.81977558212288,36.81977558222288,43.30166295106619,43.30166295116619,49.73683817476695,49.73683817486695,55.76935757625405,55.769357576354054,61.637503969586625,61.63750396968663,66.94175219852649,66.94175219862649,71.93976911729764,71.93976911739765,76.90235081055877,76.90235081065877,81.64557065225013,81.64557065235013,86.34047388238649,86.34047388248649,89.76195263811113,89.76195263821113,93.03097448305041,93.03097448315042,96.13135126522278,96.13135126532278,98.15194178578797,98.15194178588797,99.3356531133397,99.3356531134397,99.77891628214371,99.77891628224371,100.0,100.0000000001],[0.0,1e-10,9.055712464286778,9.055712464386778,16.49532494822666,16.495324948326658,23.728347657863317,23.728347657963315,30.847578564185373,30.84757856428537,37.5068941194706,37.5068941195706,44.07139734228547,44.071397342385474,50.48785153632669,50.48785153642669,56.777891467118984,56.777891467218986,62.91225003196491,62.91225003206491,68.26323780485612,68.26323780495612,73.09247979343638,73.09247979353638,77.92057725844748,77.92057725854748,82.3040567474406,82.3040567475406,86.00702868274166,86.00702868284166,89.52864922347347,89.52864922357347,92.95594794183597,92.95594794193597,95.88180799407202,95.88180799417202,97.93381449104182,97.93381449114182,99.33276891971036,99.33276891981036,99.78041497808564,99.78041497818565,100.0,100.0000000001],[0.0,1e-10,9.567795864415181,9.567795864515181,17.040129324975204,17.040129325075203,24.322050570404045,24.322050570504043,31.487223393019303,31.4872233931193,38.19309667852777,38.193096678627775,44.79933428782969,44.79933428792969,51.17366785330401,51.17366785340401,57.51525683632836,57.515256836428364,63.66830942452268,63.668309424622684,69.10683902361777,69.10683902371777,73.96566774957444,73.96566774967444,78.42466373288372,78.42466373298372,82.76127725065034,82.76127725075034,86.69019932598157,86.69019932608157,90.23401220257306,90.23401220267306,93.623454941118,93.623454941218,96.52273596223296,96.52273596233296,98.5817613794664,98.5817613795664,99.32850898679179,99.32850898689179,99.77900862304719,99.77900862314719,100.0,100.0000000001],[0.0,1e-10,8.87029985652476,8.87029985662476,17.282696194142275,17.282696194242273,24.336520711014472,24.33652071111447,31.327599235439376,31.327599235539374,38.051542815298774,38.051542815398776,44.56615797395919,44.566157974059195,50.895840947814285,50.89584094791429,57.0056694982659,57.005669498365904,63.01681757887214,63.016817578972145,68.40394606561037,68.40394606571037,73.20883863856444,73.20883863866445,77.88028226086739,77.88028226096739,81.99685244985814,81.99685244995814,86.0528484752192,86.05284847531921,89.65115239863758,89.65115239873758,93.15680443898746,93.15680443908747,95.97687610933845,95.97687610943845,98.00034147043061,98.00034147053061,99.34642047054369,99.3464204706437,99.78495050760893,99.78495050770893,100.0,100.0000000001],[0.0,1e-10,8.833247961137848,8.833247961237849,16.520630834253975,16.520630834353973,23.803990117096347,23.803990117196346,30.39945351399493,30.39945351409493,36.92170083243738,36.92170083253738,43.298847904171076,43.29884790427108,49.617689794605155,49.61768979470516,55.67647784208197,55.676477842181974,61.47149569227754,61.471495692377545,66.62315543431058,66.62315543441058,71.72684691764793,71.72684691774793,76.68464550527736,76.68464550537736,81.46657347193516,81.46657347203517,86.10625992597957,86.10625992607957,89.63186521744441,89.63186521754442,92.82696186474705,92.82696186484705,96.00384057931467,96.00384057941467,98.0602774479405,98.06027744804051,98.94323645603254,98.94323645613254,99.78043622722986,99.78043622732986,100.0,100.0000000001],[0.0,1e-10,9.015068420281422,9.015068420381422,17.036599490311247,17.036599490411245,24.380993158787724,24.380993158887723,31.077620656198977,31.077620656298976,37.537698790199016,37.53769879029902,43.77933329264874,43.77933329274874,49.99908949185487,49.99908949195487,56.17162980631632,56.17162980641632,61.952658145311226,61.95265814541123,67.27529295354809,67.27529295364809,72.08725344075806,72.08725344085806,76.75032137021358,76.75032137031359,81.23426395533224,81.23426395543224,85.56196464813429,85.5619646482343,89.25745939508971,89.25745939518971,92.73338134985606,92.73338134995606,95.72639458681695,95.72639458691695,98.48977048994149,98.4897704900415,99.33640599917173,99.33640599927173,99.78006169916485,99.78006169926485,100.0,100.0000000001]],"coord\_y":[[1379102,1379102,1379102,1222833,1222833,1107062,1107062,1031096,1031096,1023375,1023375,1010111,1010111,1007122,1007122,933280,933280,905029,905029,807368,807368,777067,777067,774605,774605,747657,747657,733858,733858,546013,546013,534336,534336,502463,502463,317354,317354,140801,140801,67797,67797,33895,33895,0.0],[1393337,1393337,1393337,1351320,1351320,1096016,1096016,1094624,1094624,1088367,1088367,1033768,1033768,984013,984013,946052,946052,883539,883539,791567,791567,774784,774784,768682,768682,737726,737726,721875,721875,629083,629083,514227,514227,427001,427001,317272,317272,67979,67979,47970,47970,33775,33775,0.0],[1436564,1436564,1436564,1136833,1136833,1073283,1073283,1049310,1049310,1043668,1043668,1038315,1038315,988276,988276,917852,917852,888554,888554,783349,783349,777642,777642,738945,738945,715053,715053,688818,688818,531826,531826,527350,527350,473020,473020,317601,317601,161572,161572,67991,67991,33857,33857,0.0],[1418329,1418329,1418329,1088380,1088380,1087748,1087748,1027502,1027502,988574,988574,944000,944000,942503,942503,898195,898195,887750,887750,813897,813897,783643,783643,743143,743143,729845,729845,717342,717342,686562,686562,660540,660540,629372,629372,317231,317231,226654,226654,135955,135955,34089,34089,0.0],[1441520,1441520,1441520,1160826,1160826,1100557,1100557,1076254,1076254,1046064,1046064,1005551,1005551,988727,988727,958919,958919,887982,887982,779070,779070,776861,776861,772900,772900,752066,752066,741690,741690,635674,635674,586990,586990,455038,455038,316604,316604,192647,192647,67969,67969,33867,33867,0.0],[1405785,1405785,1405785,1193529,1193529,1083450,1083450,1045275,1045275,1034781,1034781,999314,999314,989015,989015,950931,950931,888615,888615,783923,783923,781928,781928,777094,777094,725153,725153,692187,692187,612301,612301,502873,502873,453827,453827,317192,317192,89899,89899,67969,67969,43541,43541,0.0],[1448332,1448332,1448332,1149162,1149162,1132280,1132280,1080573,1080573,1045474,1045474,1008355,1008355,989737,989737,920314,920314,888223,888223,809000,809000,776907,776907,768865,768865,750795,750795,679518,679518,657160,657160,506638,506638,475232,475232,314478,314478,230001,230001,73529,73529,33859,33859,0.0],[1430540,1430540,1430540,1174667,1174667,1072677,1072677,1042730,1042730,1027144,1027144,1024272,1024272,998368,998368,921926,921926,887734,887734,790440,790440,785317,785317,754237,754237,733045,733045,657272,657272,617914,617914,506344,506344,473691,473691,341526,341526,173068,173068,67961,67961,43543,43543,0.0],[1368327,1368327,1368327,1226042,1226042,1088739,1088739,1050855,1050855,1042420,1042420,1032508,1032508,1001997,1001997,935817,935817,887771,887771,819461,819461,787705,787705,745296,745296,739700,739700,737386,737386,725812,725812,509291,509291,320350,320350,317389,317389,228690,228690,67879,67879,33893,33893,0.0],[1369564,1369564,1369564,1292280,1292280,1088825,1088825,1050179,1050179,1044430,1044430,1001784,1001784,935865,935865,930292,930292,887823,887823,818713,818713,798075,798075,787403,787403,725809,725809,711730,711730,597613,597613,502163,502163,475900,475900,317415,317415,98457,98457,67879,67879,33894,33894,0.0],[1387673,1387673,1387673,1146138,1146138,1096451,1096451,1016660,1016660,1013628,1013628,992004,992004,986735,986735,985652,985652,911957,911957,803017,803017,748028,748028,737724,737724,734935,734935,641580,641580,521692,521692,518210,518210,482895,482895,318881,318881,233066,233066,67958,67958,33899,33899,0.0],[1368800,1368800,1368800,1215633,1215633,1094603,1094603,1017758,1017758,1013967,1013967,1006628,1006628,1001351,1001351,990084,990084,897590,897590,788643,788643,731444,731444,725711,725711,716370,716370,693896,693896,612816,612816,495993,495993,488639,488639,327379,327379,190370,190370,67949,67949,33811,33811,0.0],[1399097,1399097,1399097,1188627,1188627,1132240,1132240,1092770,1092770,1019818,1019818,1017095,1017095,1007186,1007186,945509,945509,925960,925960,825711,825711,822848,822848,755331,755331,749838,749838,746082,746082,567357,567357,522025,522025,485845,485845,341203,341203,224354,224354,135955,135955,33900,33900,0.0],[1380033,1380033,1380033,1146277,1146277,1082997,1082997,1014591,1014591,1010103,1010103,971212,971212,964497,964497,917895,917895,901748,901748,785066,785066,778859,778859,729759,729759,712812,712812,666013,666013,647879,647879,588400,588400,443152,443152,329241,329241,232968,232968,67959,67959,33865,33865,0.0],[1354659,1354659,1354659,1124202,1124202,1081319,1081319,1016380,1016380,1004405,1004405,996712,996712,984466,984466,943349,943349,896638,896638,806733,806733,770071,770071,748153,748153,726451,726451,714867,714867,575062,575062,513791,513791,451519,451519,431628,431628,319846,319846,191708,191708,33820,33820,0.0],[1359012,1359012,1359012,1122352,1122352,1076186,1076186,1042483,1042483,1016583,1016583,995001,995001,963931,963931,936923,936923,896176,896176,796427,796427,781469,781469,775097,775097,715892,715892,669509,669509,523808,523808,489133,489133,469966,469966,306851,306851,150971,150971,71655,71655,33451,33451,0.0],[1368101,1368101,1368101,1124881,1124881,1078754,1078754,1026279,1026279,1025217,1025217,1000880,1000880,972493,972493,923380,923380,898711,898711,814716,814716,769802,769802,751054,751054,747011,747011,721518,721518,529393,529393,501153,501153,468591,468591,310741,310741,185843,185843,71654,71654,43615,43615,0.0],[1404025,1404025,1404025,1125402,1125402,1079185,1079185,1023053,1023053,1005769,1005769,990880,990880,988013,988013,925144,925144,898896,898896,794956,794956,746850,746850,720742,720742,717687,717687,667499,667499,527209,527209,501400,501400,474446,474446,314146,314146,160525,160525,71655,71655,33901,33901,0.0],[1351263,1351263,1351263,1125799,1125799,1078536,1078536,1016268,1016268,1012529,1012529,993922,993922,991018,991018,925398,925398,898043,898043,793604,793604,751820,751820,747121,747121,715890,715890,647427,647427,523127,523127,495157,495157,475408,475408,309281,309281,203941,203941,71655,71655,33900,33900,0.0],[1393237,1393237,1393237,1125820,1125820,1086541,1086541,1021435,1021435,1013856,1013856,1001805,1001805,976262,976262,927198,927198,891361,891361,795128,795128,751318,751318,720376,720376,717683,717683,646622,646622,528058,528058,499512,499512,471221,471221,308069,308069,160959,160959,67968,67968,33897,33897,0.0],[1359092,1359092,1359092,1131285,1131285,1079247,1079247,1016987,1016987,1008950,1008950,1006439,1006439,989488,989488,945596,945596,894004,894004,791990,791990,781878,781878,770859,770859,764371,764371,720863,720863,523849,523849,512960,512960,474879,474879,308071,308071,225373,225373,67975,67975,33871,33871,0.0],[1350358,1350358,1350358,1125907,1125907,1079905,1079905,1040521,1040521,1022495,1022495,993645,993645,990352,990352,925420,925420,897381,897381,805861,805861,751911,751911,738719,738719,719367,719367,662588,662588,523073,523073,504031,504031,477181,477181,308408,308408,181429,181429,67965,67965,33896,33896,0.0],[1385962,1385962,1385962,1126251,1126251,1082459,1082459,1029491,1029491,1021119,1021119,993816,993816,986654,986654,924918,924918,899716,899716,813258,813258,766306,766306,760873,760873,727240,727240,719832,719832,524588,524588,501213,501213,475356,475356,309801,309801,181489,181489,67962,67962,33897,33897,0.0],[1384637,1384637,1384637,1137532,1137532,1105944,1105944,1088545,1088545,1018223,1018223,1003726,1003726,981089,981089,961760,961760,937956,937956,818177,818177,738401,738401,738226,738226,670243,670243,566192,566192,538463,538463,524041,524041,447370,447370,313756,313756,213903,213903,68446,68446,33575,33575,0.0],[1453669,1453669,1453669,1135298,1135298,1106368,1106368,1088630,1088630,1018847,1018847,1003709,1003709,968475,968475,963500,963500,934855,934855,826295,826295,738219,738219,677471,677471,658877,658877,596935,596935,538424,538424,514970,514970,440498,440498,312835,312835,113456,113456,68446,68446,33576,33576,0.0],[1384562,1384562,1384562,1313088,1313088,1101029,1101029,1091235,1091235,1049538,1049538,1016864,1016864,987998,987998,953681,953681,938278,938278,840875,840875,749994,749994,729164,729164,642554,642554,633099,633099,561658,561658,547196,547196,440184,440184,315842,315842,210109,210109,68450,68450,33567,33567,0.0],[1359563,1359563,1359563,1183198,1183198,1121013,1121013,1015136,1015136,1003867,1003867,981534,981534,972560,972560,932534,932534,891936,891936,792914,792914,785531,785531,763076,763076,736007,736007,714114,714114,542641,542641,491771,491771,488967,488967,316515,316515,135900,135900,128857,128857,33794,33794,0.0],[1381209,1381209,1381209,1228988,1228988,1125243,1125243,1025998,1025998,989756,989756,956288,956288,952936,952936,945702,945702,885718,885718,815487,815487,737246,737246,714434,714434,686990,686990,663052,663052,566191,566191,532550,532550,458563,458563,423380,423380,129714,129714,67973,67973,33697,33697,0.0]],"filenames":["A818","B218","B518","C218","E318","H718","D119","H44","A727","A827","RCC5417","RCC1613","RCC685","RCC1615","RCC1868","RCC4222","G11","C2","G2","E2","A8","B8","A1","C3","G5","G8","RCC4752","RCC716"]}

{"coord\_x":[[0.0,1e-10,9.148684650451894,9.148684650551894,17.260712257667343,17.26071225776734,24.604738389526027,24.604738389626025,31.444821391611693,31.44482139171169,38.23368483619825,38.23368483629825,44.93455757871665,44.93455757881665,51.61560189779705,51.61560189789705,57.80679327492053,57.80679327502053,63.81057321325274,63.81057321335274,69.16648976537581,69.16648976547582,74.32139559197364,74.32139559207364,79.45996900689384,79.45996900699384,84.41977482234688,84.41977482244688,89.2880408535841,89.28804085368411,92.91018102309093,92.91018102319093,96.45485832860122,96.45485832870122,99.78809657749072,99.78809657759072,101.89335903709089,101.89335903719089,102.82740448657054,102.82740448667055,103.27715611715819,103.27715611725819,103.50200871415758,103.50200871425758],[0.0,1e-10,9.243116770773076,9.243116770873076,18.207501233886504,18.207501233986502,25.478250428543376,25.478250428643374,32.739765375817946,32.73976537591795,39.959772646461,39.959772646561,46.81758115788971,46.817581157989714,53.34532502958674,53.34532502968674,59.621243279962215,59.62124328006222,65.4824628905317,65.48246289063171,70.73355879402853,70.73355879412853,75.87331965886355,75.87331965896355,80.97260108582012,80.97260108592012,85.86652664929495,85.86652664939496,90.65529987422319,90.65529987432319,94.82850967738511,94.82850967748512,98.23978793073253,98.23978793083253,101.07242648424605,101.07242648434605,103.17714497237687,103.17714497247687,103.62810395427455,103.62810395437455,103.94632726385005,103.94632726395005,104.17038380504063,104.17038380514063],[0.0,1e-10,9.529875974504986,9.529875974604986,17.071396918733317,17.071396918833315,24.191339974207793,24.19133997430779,31.152250980475404,31.152250980575403,38.0757340961317,38.0757340962317,44.96370648891625,44.96370648901625,51.51973024322159,51.51973024332159,57.608575378524534,57.608575378624536,63.503063488104274,63.503063488204276,68.69964283629378,68.69964283639378,73.85836309697552,73.85836309707553,78.76037526070827,78.76037526080827,83.50389271290513,83.50389271300513,88.07337246389886,88.07337246399887,91.60139893540803,91.60139893550803,95.09973252524824,95.09973252534824,98.23765184764554,98.23765184774554,100.34455285545219,100.3445528555522,101.41638893164003,101.41638893174003,101.8674275191186,101.86742751921861,102.09202803177855,102.09202803187856],[0.0,1e-10,9.408908660556497,9.408908660656497,16.62900217057884,16.629002170678838,23.844903120008066,23.844903120108064,30.661144250619596,30.661144250719595,37.219144876850166,37.21914487695017,43.48145057289483,43.48145057299483,49.733825472724476,49.73382547282448,55.69227003274443,55.692270032844434,61.581424568405076,61.58142456850508,66.98065319032634,66.98065319042634,72.17918287524743,72.17918287534744,77.10904372469206,77.10904372479206,81.95068832292269,81.95068832302269,86.70939053967277,86.70939053977277,91.26390444146071,91.26390444156071,95.6457936411062,95.6457936412062,99.82092061200771,99.82092061210771,101.92536711440384,101.92536711450384,103.4289440585048,103.4289440586048,104.33084212090496,104.33084212100496,104.55698167479528,104.55698167489528],[0.0,1e-10,9.562753079409221,9.562753079509221,17.26343874881255,17.26343874891255,24.564312022034827,24.564312022134825,31.703964092575983,31.70396409267598,38.643341789215036,38.64334178931504,45.31396441099831,45.31396441109831,51.87298000838512,51.87298000848512,58.23425534286124,58.23425534296124,64.12494891975227,64.12494891985227,69.29314224455896,69.29314224465897,74.44668150868497,74.44668150878498,79.57394429732153,79.57394429742153,84.56299852995028,84.56299853005028,89.48322047030977,89.48322047040978,93.7001536387711,93.7001536388711,97.59412696559447,97.59412696569447,100.6127573250402,100.61275732514021,102.71304443583524,102.71304443593525,103.99102579751525,103.99102579761525,104.44191844142887,104.44191844152887,104.66658529207288,104.66658529217288],[0.0,1e-10,9.32569429334126,9.32569429344126,17.24332507204305,17.243325072143048,24.430713955919735,24.430713956019734,31.364857585615802,31.3648575857158,38.22938613483063,38.22938613493063,44.858633755950514,44.858633756050516,51.419559887278496,51.4195598873785,57.72784444008088,57.72784444018088,63.62273721136343,63.62273721146343,68.82312435983846,68.82312435993846,74.01027708049186,74.01027708059186,79.16536201964665,79.16536201974665,83.97588083575246,83.97588083585246,88.5677098535788,88.5677098536788,92.62959125187737,92.62959125197737,95.96554935811366,95.96554935821366,98.97614618768873,98.97614618778873,101.080333971947,101.080333972047,101.67670581492233,101.67670581502233,102.12759845883595,102.12759845893595,102.41644067526761,102.41644067536761],[0.0,1e-10,9.607942514156527,9.607942514256527,17.231251558942624,17.231251559042622,24.742568819024672,24.74256881912467,31.910872264884915,31.910872264984913,38.846336020463944,38.846336020563946,45.535559812979955,45.535559813079956,52.10127554675766,52.101275546857664,58.206453093738226,58.20645309383823,64.0987454160453,64.0987454161453,69.46548832716833,69.46548832726833,74.61933274602104,74.61933274612105,79.71982815808607,79.71982815818608,84.70045083293972,84.70045083303972,89.20823625874998,89.20823625884998,93.5677032197804,93.5677032198804,96.9286375770184,96.92863757711841,100.08123086149159,100.08123086159159,102.16741451687373,102.16741451697374,103.69319478424234,103.69319478434234,104.18097134729793,104.18097134739793,104.40558512755467,104.40558512765467],[0.0,1e-10,9.48991397290226,9.48991397300226,17.28241804605448,17.28241804615448,24.39834101969442,24.39834101979442,31.315601632445112,31.31560163254511,38.129467863227,38.129467863327,44.924281824984476,44.92428182508448,51.54725387281151,51.54725387291151,57.66312510282388,57.66312510292388,63.552173497710015,63.55217349781002,68.79579311040233,68.79579311050233,74.00542777385647,74.00542777395647,79.00888398282642,79.00888398292642,83.8717567359589,83.8717567360589,88.23196668241089,88.23196668251089,92.33108359116696,92.33108359126696,95.69006761167336,95.69006761177336,98.83243821280163,98.83243821290164,101.09805284749163,101.09805284759163,102.24615107016436,102.24615107026436,102.69699064369073,102.69699064379073,102.9858461277192,102.9858461278192],[0.0,1e-10,9.077205472618333,9.077205472718333,17.21052093892129,17.21052093902129,24.432995982571686,24.432995982671684,31.404156207377845,31.404156207477843,38.31936034262242,38.31936034272242,45.16881026805853,45.16881026815853,51.81585637030393,51.815856370403935,58.02387769398553,58.023877694085535,63.91317153941272,63.913171539512724,69.34931061566957,69.34931061576957,74.5747867897192,74.5747867898192,79.51893020713372,79.51893020723372,84.42595088866364,84.42595088876364,89.31762096068015,89.31762096078015,94.13251144993605,94.13251145003605,97.51104527434737,97.51104527444737,99.63618259397438,99.63618259407438,101.7416772365188,101.7416772366188,103.25876059417605,103.25876059427605,103.70905619623306,103.70905619633307,103.93389552563565,103.93389552573565],[0.0,1e-10,9.08541148124758,9.08541148134758,17.65813648642194,17.65813648652194,24.88118203673532,24.88118203683532,31.847857813818468,31.847857813918466,38.776395883860765,38.77639588396077,45.42202898704552,45.422028987145524,51.630368733050645,51.63036873315065,57.801738320534525,57.80173832063453,63.69137712347887,63.691377123578874,69.1225541185274,69.1225541186274,74.41682278205585,74.41682278215585,79.64029554898661,79.64029554908662,84.4551661368473,84.4551661369473,89.17663947693826,89.17663947703826,93.14108364423736,93.14108364433736,96.4723317536048,96.47233175370481,99.62935641541377,99.62935641551377,101.73502353671675,101.73502353681675,102.38816742645771,102.38816742655771,102.83846302851472,102.83846302861473,103.06330899171572,103.06330899181572],[0.0,1e-10,9.205542936596808,9.205542936696808,16.808791375000663,16.80879137510066,24.082426271964508,24.082426272064506,30.82674376024922,30.826743760349217,37.55094757176443,37.550947571864434,44.13170212653042,44.13170212663042,50.67750319749083,50.67750319759083,57.21611986477665,57.21611986487665,63.26585875847136,63.26585875857136,68.59291165372633,68.59291165382633,73.5551786083883,73.5551786084883,78.44909090426633,78.44909090436633,83.32450153638771,83.32450153648772,87.58061391823976,87.58061391833976,91.04141347669415,91.04141347679415,94.47911414909595,94.47911414919595,97.68254223076066,97.68254223086066,99.79793450052804,99.79793450062805,101.34404736001359,101.34404736011359,101.79486703214474,101.79486703224474,102.01974616433776,102.01974616443776],[0.0,1e-10,9.080343259264763,9.080343259364763,17.14460751795106,17.144607518051057,24.405983155459086,24.405983155559085,31.157584554394493,31.15758455449449,37.88403722356962,37.88403722366962,44.56180444623705,44.56180444633705,51.20456511471164,51.20456511481164,57.772582776536524,57.772582776636526,63.72701388852035,63.72701388862035,68.9587125654756,68.9587125655756,73.81096460735874,73.81096460745874,78.62518508297555,78.62518508307555,83.37743924767419,83.37743924777419,87.9806054269778,87.9806054270778,92.04590323145588,92.04590323155588,95.33622080465321,95.33622080475321,98.57775342436673,98.57775342446674,100.74951971299535,100.74951971309535,102.012395915703,102.012395915803,102.46315588364848,102.46315588374848,102.68745124158171,102.68745124168171],[0.0,1e-10,9.2813274495964,9.2813274496964,17.166439348507925,17.166439348607923,24.6774912566537,24.677491256753697,31.926707141682012,31.92670714178201,38.69197416533549,38.69197416543549,45.439177355927164,45.439177356027166,52.1206462381056,52.1206462382056,58.392962335946166,58.39296233604617,64.53559430873167,64.53559430883168,70.01319462503118,70.01319462513118,75.47180237649194,75.47180237659194,80.48251596091896,80.48251596101896,85.4567900906973,85.4567900907973,90.40614767365958,90.40614767375958,94.16987963636171,94.16987963646172,97.63288824968556,97.63288824978557,100.8558860366504,100.8558860367504,103.119357954455,103.119357954555,104.60767716222026,104.60767716232026,105.50957522462042,105.50957522472042,105.73446099061185,105.73446099071185],[0.0,1e-10,9.15486071676865,9.15486071686865,16.759031253151054,16.759031253251052,23.94341502634945,23.943415026449447,30.674007185730435,30.674007185830433,37.374826857861585,37.37482685796159,43.81765147615282,43.81765147625282,50.21593013814222,50.21593013824222,56.305060526776664,56.305060526876666,62.287074972536075,62.28707497263608,67.4950445525901,67.4950445526901,72.66183814593295,72.66183814603295,77.5029122375006,77.5029122376006,82.23156334746774,82.23156334756774,86.6497593257938,86.6497593258938,90.94765800381046,90.94765800391046,94.85098498638745,94.85098498648745,97.79076601796963,97.79076601806963,99.97488443923176,99.97488443933176,101.52034718647342,101.52034718657342,101.97117349240297,101.97117349250297,102.19582707545017,102.19582707555017],[0.0,1e-10,8.986534715993823,8.986534716093823,16.44426415254552,16.44426415264552,23.61751641201726,23.617516412117258,30.35997643674806,30.359976436848058,37.02299672555711,37.02299672565711,43.63498320322243,43.634983203322435,50.16573218559776,50.16573218569776,56.42371927887958,56.42371927897958,62.3718350147801,62.371835014880105,67.72353910491485,67.72353910501485,72.8320348778585,72.8320348779585,77.79513105732133,77.79513105742133,82.61426054375919,82.61426054385919,87.35654410945237,87.35654410955237,91.1713894888791,91.1713894889791,94.57977540612114,94.57977540622115,97.57506142897324,97.57506142907324,100.43839456771516,100.43839456781517,102.56018845294514,102.56018845304514,103.83194067792112,103.83194067802113,104.05629574004001,104.05629574014002],[0.0,1e-10,9.01541164045874,9.01541164055874,16.46086854995781,16.460868550057807,23.600069522207303,23.6000695223073,30.515691586751508,30.515691586851506,37.25949827255889,37.259498272658895,43.86013432115014,43.86013432125014,50.254658253241274,50.254658253341276,56.470016557960825,56.47001655806083,62.41506747899739,62.415067479097395,67.69840364275139,67.69840364285139,72.88251144993605,72.88251145003605,78.02434869367242,78.02434869377242,82.7734319027326,82.7734319028326,87.21481964028891,87.21481964038891,90.68965631617213,90.68965631627213,93.93446603229864,93.93446603239865,97.05212573436148,97.05212573446148,99.08771340929475,99.08771340939475,100.08922458857182,100.08922458867183,100.56456941341301,100.56456941351301,100.78647660391978,100.78647660401978],[0.0,1e-10,9.07570623417839,9.07570623427839,16.537940019848325,16.537940019948323,23.694176586406552,23.69417658650655,30.50230458156653,30.50230458166653,37.303387482818465,37.30338748291847,43.943023632243445,43.94302363234345,50.39434614629383,50.39434614639383,56.51986291918972,56.51986291928972,62.4817305191876,62.4817305192876,67.88639222200405,67.88639222210405,72.99310350317626,72.99310350327626,77.97544433181729,77.97544433191729,82.93096471349952,82.93096471359952,87.7173696723965,87.7173696724965,91.22925611238185,91.22925611248185,94.55380408535841,94.55380408545841,97.66234231461188,97.66234231471188,99.72373546534769,99.72373546544769,100.95658046266763,100.95658046276763,101.43191865371041,101.43191865381041,101.72125177122417,101.72125177132418],[0.0,1e-10,9.314018808145242,9.314018808245242,16.779708802785134,16.779708802885132,23.938804536456704,23.938804536556702,30.72553189795626,30.725531898056257,37.39760068779222,37.39760068789222,43.97089885314893,43.97089885324893,50.52517791847327,50.52517791857327,56.6623967117588,56.662396711858804,62.625491564461946,62.62549156456195,67.8990694107595,67.8990694108595,72.85352175089821,72.85352175099821,77.6347788822315,77.6347788823315,82.39576975943193,82.39576975953193,86.82382356219054,86.82382356229054,90.32122178645538,90.32122178655538,93.64740830763841,93.64740830773842,96.79478742656386,96.79478742666386,98.87876866087493,98.87876866097493,99.94365915013081,99.94365915023081,100.419003974972,100.419003975072,100.64389637476185,100.64389637486185],[0.0,1e-10,8.964006336604239,8.964006336704239,16.43232994921164,16.432329949311637,23.58712034771718,23.587120347817176,30.328837387026415,30.328837387126413,37.04575065409252,37.045750654192524,43.639228834202804,43.639228834302806,50.21344246373966,50.21344246383966,56.35234624182053,56.35234624192053,62.309782464482645,62.309782464582646,67.57439141533416,67.57439141543416,72.56181373355481,72.56181373365482,77.51806383306179,77.51806383316179,82.26713377452515,82.26713377462515,86.56203397566192,86.56203397576192,90.0323530348301,90.0323530349301,93.31712475255932,93.31712475265932,96.47088558555211,96.47088558565211,98.52259339061398,98.52259339071398,99.87549687150067,99.87549687160067,100.35084169634186,100.35084169644186,100.57572746233329,100.57572746243329],[0.0,1e-10,9.242453390932393,9.242453391032393,16.71091631330634,16.710916313406337,23.918810268058525,23.918810268158524,30.694804143735837,30.694804143835835,37.42052046128781,37.42052046138781,44.0662928742391,44.066292874339105,50.54261817448482,50.542618174584824,56.693462789697975,56.69346278979798,62.60657197140568,62.60657197150568,67.88129083102919,67.88129083112919,72.86538298244962,72.86538298254962,77.64421214356601,77.64421214366601,82.40517648557281,82.40517648567281,86.69473647899208,86.69473647909209,90.19776679810433,90.19776679820433,93.51142870789528,93.51142870799528,96.63741382695869,96.6374138270587,98.68108146835148,98.68108146845148,99.74885102611594,99.74885102621595,100.19973703623116,100.19973703633116,100.42460290082737,100.42460290092737],[0.0,1e-10,9.015942344331286,9.015942344431286,16.52065897499854,16.520658975098538,23.680166004171333,23.68016600427133,30.426652744535076,30.426652744635074,37.11982364710315,37.119823647203155,43.796337081871684,43.796337081971686,50.36040099984609,50.360400999946094,56.63329423814805,56.633294238248055,62.563936549044996,62.563936549145,67.81783854926789,67.81783854936789,73.00465958000096,73.00465958010096,78.11838278608919,78.11838278618919,83.18906590811393,83.18906590821393,87.97112572905445,87.97112572915445,91.44623439067234,91.44623439077235,94.8491076214383,94.84910762153831,97.9993591750739,97.99935917517391,100.04304008406349,100.0430400841635,101.53811913240531,101.53811913250532,101.98905157910937,101.98905157920937,102.21374496494701,102.21374496504701],[0.0,1e-10,8.95800274904606,8.95800274914606,16.4270428118814,16.427042811981398,23.590914880405883,23.59091488050588,30.493521432475895,30.493521432575893,37.27654713446444,37.27654713456444,43.86818775241603,43.86818775251603,50.437983272213934,50.437983272313936,56.57703299385975,56.577032993959754,62.530077641976554,62.530077642076556,67.87599705990054,67.87599706000054,72.86402305377622,72.86402305387622,77.76453597906904,77.76453597916904,82.53667163759295,82.53667163769295,86.93214685637561,86.93214685647561,90.40210769042982,90.40210769052982,93.74574773522123,93.74574773532123,96.91127029278933,96.91127029288933,98.95718679184202,98.95718679194202,100.16075020299424,100.16075020309424,100.61161631171423,100.61161631181423,100.83647554251203,100.83647554261204],[0.0,1e-10,9.194192507522727,9.194192507622727,16.665514597010013,16.66551459711001,23.846329386665534,23.846329386765532,30.675765142308244,30.675765142408242,37.449662737688996,37.449662737789,44.04243773516815,44.04243773526815,50.58770146845762,50.58770146855762,56.72342102330321,56.72342102340321,62.69195559069995,62.69195559079995,68.08694521543924,68.08694521553925,73.1704647373812,73.1704647374812,78.21794283257886,78.21794283267886,83.0423063859597,83.0423063860597,87.81752676074278,87.81752676084278,91.29753779938332,91.29753779948332,94.62248380026429,94.6224838003643,97.77589967573994,97.77589967583994,99.83105705597333,99.83105705607333,101.03501849502996,101.03501849512996,101.48586470235473,101.48586470245473,101.71073056695094,101.71073056705094],[0.0,1e-10,9.185402724633681,9.185402724733681,16.731560693948385,16.731560694048383,24.068170239188234,24.068170239288232,31.289358325947706,31.289358326047704,38.04404444114229,38.04404444124229,44.7025603808331,44.7025603809331,51.21090702598857,51.21090702608857,57.59102898173848,57.591028981838484,63.81324000021228,63.813240000312284,69.24086127931476,69.24086127941476,74.1392646567142,74.1392646568142,79.03650711939245,79.03650711949246,83.48276406497938,83.48276406507938,87.23876765253756,87.23876765263756,90.81082264407283,90.81082264417283,94.28720499498485,94.28720499508485,97.25496738824704,97.25496738834704,99.33636144117943,99.33636144127944,100.75535082179495,100.75535082189495,101.20940778754863,101.20940778764863,101.43213756905784,101.43213756915785],[0.0,1e-10,9.64334709625376,9.64334709635376,17.174685159927613,17.17468516002761,24.514107435691958,24.514107435791956,31.73585939531601,31.735859395416007,38.49468500071645,38.49468500081645,45.15308816583435,45.15308816593435,51.5777560778861,51.5777560779861,57.96942084286389,57.96942084296389,64.17106045247812,64.17106045257812,69.65253490704721,69.65253490714721,74.54973093313662,74.54973093323662,79.0439369736081,79.0439369737081,83.41479416650303,83.41479416660303,87.37474061848229,87.3747406185823,90.9465368918797,90.9465368919797,94.3627440574434,94.3627440575434,97.28491898805386,97.28491898815386,99.36020331265357,99.36020331275357,100.11284754469854,100.11284754479854,100.56690451045222,100.56690451055222,100.78964092575984,100.78964092585984],[0.0,1e-10,9.18490518975317,9.18490518985317,17.89566627217679,17.895666272276788,25.199670698247086,25.199670698347084,32.43870370272092,32.43870370282092,39.40112721502529,39.40112721512529,46.146797998184994,46.146797998284995,52.70097755653323,52.700977556633234,59.02750505495439,59.02750505505439,65.25185215651518,65.25185215661519,70.83004739185581,70.83004739195582,75.80535639418561,75.80535639428561,80.64248337570119,80.64248337580119,84.90505707720149,84.90505707730149,89.10490821476525,89.10490821486525,92.83083416034687,92.83083416044687,96.46082211336896,96.46082211346896,99.38091403127969,99.38091403137969,101.47614618768873,101.47614618778873,102.86996693714875,102.86996693724875,103.32405043809605,103.32405043819605,103.54672714921801,103.54672714931802],[0.0,1e-10,9.019066863380901,9.019066863480901,16.868163870741764,16.868163870841762,24.304738124174094,24.304738124274092,31.0389457036868,31.038945703786798,37.69839700895297,37.698397009052975,44.209695694399485,44.20969569449949,50.661462672943124,50.661462673043125,56.84770523645511,56.847705236555115,62.76462885224674,62.76462885234674,68.02466048219753,68.02466048229753,73.23571477851074,73.23571477861074,78.29780713159865,78.29780713169865,83.18032919561215,83.18032919571215,87.91761751110498,87.91761751120498,91.51738851238397,91.51738851248398,94.77969818870768,94.77969818880769,98.02340669429864,98.02340669439864,100.12310339703549,100.1231033971355,101.02463660052328,101.02463660062328,101.87944796183177,101.87944796193177,102.10363054519209,102.10363054529209],[0.0,1e-10,9.162662063695079,9.162662063795079,17.31552070010455,17.315520700204548,24.780155920797753,24.78015592089775,31.586419818605417,31.586419818705416,38.152261594552854,38.152261594652856,44.496083405420606,44.49608340552061,50.81766872402868,50.81766872412868,57.09126514496176,57.09126514506176,62.96693980225974,62.96693980235974,68.37671616364784,68.37671616374784,73.2674575038874,73.2674575039874,78.00686863487043,78.00686863497043,82.5642218023765,82.5642218024765,86.96277510361993,86.96277510371993,90.71877205737971,90.71877205747971,94.2516013989354,94.25160139903541,97.2936158977652,97.29361589786521,100.10223346724761,100.10223346734762,100.96272999379076,100.96272999389076,101.41364917289802,101.41364917299802,101.63718827781287,101.63718827791287]],"coord\_y":[[1379102,1379102,1379102,1222833,1222833,1107062,1107062,1031096,1031096,1023375,1023375,1010111,1010111,1007122,1007122,933280,933280,905029,905029,807368,807368,777067,777067,774605,774605,747657,747657,733858,733858,546013,546013,534336,534336,502463,502463,317354,317354,140801,140801,67797,67797,33895,33895,0.0],[1393337,1393337,1393337,1351320,1351320,1096016,1096016,1094624,1094624,1088367,1088367,1033768,1033768,984013,984013,946052,946052,883539,883539,791567,791567,774784,774784,768682,768682,737726,737726,721875,721875,629083,629083,514227,514227,427001,427001,317272,317272,67979,67979,47970,47970,33775,33775,0.0],[1436564,1436564,1436564,1136833,1136833,1073283,1073283,1049310,1049310,1043668,1043668,1038315,1038315,988276,988276,917852,917852,888554,888554,783349,783349,777642,777642,738945,738945,715053,715053,688818,688818,531826,531826,527350,527350,473020,473020,317601,317601,161572,161572,67991,67991,33857,33857,0.0],[1418329,1418329,1418329,1088380,1088380,1087748,1087748,1027502,1027502,988574,988574,944000,944000,942503,942503,898195,898195,887750,887750,813897,813897,783643,783643,743143,743143,729845,729845,717342,717342,686562,686562,660540,660540,629372,629372,317231,317231,226654,226654,135955,135955,34089,34089,0.0],[1441520,1441520,1441520,1160826,1160826,1100557,1100557,1076254,1076254,1046064,1046064,1005551,1005551,988727,988727,958919,958919,887982,887982,779070,779070,776861,776861,772900,772900,752066,752066,741690,741690,635674,635674,586990,586990,455038,455038,316604,316604,192647,192647,67969,67969,33867,33867,0.0],[1405785,1405785,1405785,1193529,1193529,1083450,1083450,1045275,1045275,1034781,1034781,999314,999314,989015,989015,950931,950931,888615,888615,783923,783923,781928,781928,777094,777094,725153,725153,692187,692187,612301,612301,502873,502873,453827,453827,317192,317192,89899,89899,67969,67969,43541,43541,0.0],[1448332,1448332,1448332,1149162,1149162,1132280,1132280,1080573,1080573,1045474,1045474,1008355,1008355,989737,989737,920314,920314,888223,888223,809000,809000,776907,776907,768865,768865,750795,750795,679518,679518,657160,657160,506638,506638,475232,475232,314478,314478,230001,230001,73529,73529,33859,33859,0.0],[1430540,1430540,1430540,1174667,1174667,1072677,1072677,1042730,1042730,1027144,1027144,1024272,1024272,998368,998368,921926,921926,887734,887734,790440,790440,785317,785317,754237,754237,733045,733045,657272,657272,617914,617914,506344,506344,473691,473691,341526,341526,173068,173068,67961,67961,43543,43543,0.0],[1368327,1368327,1368327,1226042,1226042,1088739,1088739,1050855,1050855,1042420,1042420,1032508,1032508,1001997,1001997,935817,935817,887771,887771,819461,819461,787705,787705,745296,745296,739700,739700,737386,737386,725812,725812,509291,509291,320350,320350,317389,317389,228690,228690,67879,67879,33893,33893,0.0],[1369564,1369564,1369564,1292280,1292280,1088825,1088825,1050179,1050179,1044430,1044430,1001784,1001784,935865,935865,930292,930292,887823,887823,818713,818713,798075,798075,787403,787403,725809,725809,711730,711730,597613,597613,502163,502163,475900,475900,317415,317415,98457,98457,67879,67879,33894,33894,0.0],[1387673,1387673,1387673,1146138,1146138,1096451,1096451,1016660,1016660,1013628,1013628,992004,992004,986735,986735,985652,985652,911957,911957,803017,803017,748028,748028,737724,737724,734935,734935,641580,641580,521692,521692,518210,518210,482895,482895,318881,318881,233066,233066,67958,67958,33899,33899,0.0],[1368800,1368800,1368800,1215633,1215633,1094603,1094603,1017758,1017758,1013967,1013967,1006628,1006628,1001351,1001351,990084,990084,897590,897590,788643,788643,731444,731444,725711,725711,716370,716370,693896,693896,612816,612816,495993,495993,488639,488639,327379,327379,190370,190370,67949,67949,33811,33811,0.0],[1399097,1399097,1399097,1188627,1188627,1132240,1132240,1092770,1092770,1019818,1019818,1017095,1017095,1007186,1007186,945509,945509,925960,925960,825711,825711,822848,822848,755331,755331,749838,749838,746082,746082,567357,567357,522025,522025,485845,485845,341203,341203,224354,224354,135955,135955,33900,33900,0.0],[1380033,1380033,1380033,1146277,1146277,1082997,1082997,1014591,1014591,1010103,1010103,971212,971212,964497,964497,917895,917895,901748,901748,785066,785066,778859,778859,729759,729759,712812,712812,666013,666013,647879,647879,588400,588400,443152,443152,329241,329241,232968,232968,67959,67959,33865,33865,0.0],[1354659,1354659,1354659,1124202,1124202,1081319,1081319,1016380,1016380,1004405,1004405,996712,996712,984466,984466,943349,943349,896638,896638,806733,806733,770071,770071,748153,748153,726451,726451,714867,714867,575062,575062,513791,513791,451519,451519,431628,431628,319846,319846,191708,191708,33820,33820,0.0],[1359012,1359012,1359012,1122352,1122352,1076186,1076186,1042483,1042483,1016583,1016583,995001,995001,963931,963931,936923,936923,896176,896176,796427,796427,781469,781469,775097,775097,715892,715892,669509,669509,523808,523808,489133,489133,469966,469966,306851,306851,150971,150971,71655,71655,33451,33451,0.0],[1368101,1368101,1368101,1124881,1124881,1078754,1078754,1026279,1026279,1025217,1025217,1000880,1000880,972493,972493,923380,923380,898711,898711,814716,814716,769802,769802,751054,751054,747011,747011,721518,721518,529393,529393,501153,501153,468591,468591,310741,310741,185843,185843,71654,71654,43615,43615,0.0],[1404025,1404025,1404025,1125402,1125402,1079185,1079185,1023053,1023053,1005769,1005769,990880,990880,988013,988013,925144,925144,898896,898896,794956,794956,746850,746850,720742,720742,717687,717687,667499,667499,527209,527209,501400,501400,474446,474446,314146,314146,160525,160525,71655,71655,33901,33901,0.0],[1351263,1351263,1351263,1125799,1125799,1078536,1078536,1016268,1016268,1012529,1012529,993922,993922,991018,991018,925398,925398,898043,898043,793604,793604,751820,751820,747121,747121,715890,715890,647427,647427,523127,523127,495157,495157,475408,475408,309281,309281,203941,203941,71655,71655,33900,33900,0.0],[1393237,1393237,1393237,1125820,1125820,1086541,1086541,1021435,1021435,1013856,1013856,1001805,1001805,976262,976262,927198,927198,891361,891361,795128,795128,751318,751318,720376,720376,717683,717683,646622,646622,528058,528058,499512,499512,471221,471221,308069,308069,160959,160959,67968,67968,33897,33897,0.0],[1359092,1359092,1359092,1131285,1131285,1079247,1079247,1016987,1016987,1008950,1008950,1006439,1006439,989488,989488,945596,945596,894004,894004,791990,791990,781878,781878,770859,770859,764371,764371,720863,720863,523849,523849,512960,512960,474879,474879,308071,308071,225373,225373,67975,67975,33871,33871,0.0],[1350358,1350358,1350358,1125907,1125907,1079905,1079905,1040521,1040521,1022495,1022495,993645,993645,990352,990352,925420,925420,897381,897381,805861,805861,751911,751911,738719,738719,719367,719367,662588,662588,523073,523073,504031,504031,477181,477181,308408,308408,181429,181429,67965,67965,33896,33896,0.0],[1385962,1385962,1385962,1126251,1126251,1082459,1082459,1029491,1029491,1021119,1021119,993816,993816,986654,986654,924918,924918,899716,899716,813258,813258,766306,766306,760873,760873,727240,727240,719832,719832,524588,524588,501213,501213,475356,475356,309801,309801,181489,181489,67962,67962,33897,33897,0.0],[1384637,1384637,1384637,1137532,1137532,1105944,1105944,1088545,1088545,1018223,1018223,1003726,1003726,981089,981089,961760,961760,937956,937956,818177,818177,738401,738401,738226,738226,670243,670243,566192,566192,538463,538463,524041,524041,447370,447370,313756,313756,213903,213903,68446,68446,33575,33575,0.0],[1453669,1453669,1453669,1135298,1135298,1106368,1106368,1088630,1088630,1018847,1018847,1003709,1003709,968475,968475,963500,963500,934855,934855,826295,826295,738219,738219,677471,677471,658877,658877,596935,596935,538424,538424,514970,514970,440498,440498,312835,312835,113456,113456,68446,68446,33576,33576,0.0],[1384562,1384562,1384562,1313088,1313088,1101029,1101029,1091235,1091235,1049538,1049538,1016864,1016864,987998,987998,953681,953681,938278,938278,840875,840875,749994,749994,729164,729164,642554,642554,633099,633099,561658,561658,547196,547196,440184,440184,315842,315842,210109,210109,68450,68450,33567,33567,0.0],[1359563,1359563,1359563,1183198,1183198,1121013,1121013,1015136,1015136,1003867,1003867,981534,981534,972560,972560,932534,932534,891936,891936,792914,792914,785531,785531,763076,763076,736007,736007,714114,714114,542641,542641,491771,491771,488967,488967,316515,316515,135900,135900,128857,128857,33794,33794,0.0],[1381209,1381209,1381209,1228988,1228988,1125243,1125243,1025998,1025998,989756,989756,956288,956288,952936,952936,945702,945702,885718,885718,815487,815487,737246,737246,714434,714434,686990,686990,663052,663052,566191,566191,532550,532550,458563,458563,423380,423380,129714,129714,67973,67973,33697,33697,0.0]],"filenames":["A818","B218","B518","C218","E318","H718","D119","H44","A727","A827","RCC5417","RCC1613","RCC685","RCC1615","RCC1868","RCC4222","G11","C2","G2","E2","A8","B8","A1","C3","G5","G8","RCC4752","RCC716"]}

{"coord\_x":[[0.0,1e-10,3.1623696724261876,3.1623696725261876,5.091549768802191,5.091549768902191,6.997951061335871,6.997951061435871,8.788759858850892,8.788759858950892,10.507290499098078,10.507290499198078,11.962993224555678,11.962993224655678,13.377842799846997,13.377842799946997,14.631946061023095,14.631946061123095,15.838062573643347,15.838062573743347,17.031366810270125,17.031366810370123,18.199969440254158,18.199969440354156,19.35106174606902,19.35106174616902,20.501769491323802,20.5017694914238,21.623994117761672,21.62399411786167,22.73431018552227,22.734310185622267,23.835691629603573,23.83569162970357,24.84650906178504,24.846509061885037,25.82085092484251,25.82085092494251,26.735079563016143,26.73507956311614,27.632874646588846,27.632874646688844,28.502532715848716,28.502532715948714,29.372094644968563,29.37209464506856,30.235240822077674,30.235240822177673,31.07955635042799,31.079556350527987,31.893414682419635,31.893414682519634,32.68832058814179,32.688320588241794,33.47130511650134,33.47130511660134,34.254007633783495,34.2540076338835,35.02993547586549,35.029935475965495,35.80308166322955,35.80308166332955,36.524273718926224,36.524273719026226,37.22862202209121,37.22862202219121,37.8689153546315,37.8689153547315,38.49505685856068,38.49505685866068,39.11952552405349,39.119525524153495,39.74394291480497,39.74394291490497,40.36552096675448,40.36552096685448,40.983823844600614,40.983823844700616,41.597165891221664,41.597165891321666,42.20941194024647,42.20941194034647,42.8214913463619,42.821491346461904,43.41084963271903,43.41084963281903,43.999663124949365,43.999663125049366,44.58585519602846,44.58585519612846,45.16970144769105,45.16970144779105,45.7482151262538,45.7482151263538,46.32112703932465,46.32112703942465,46.88266236915968,46.882662369259684,47.4435567647279,47.4435567648279,47.994632047328636,47.99463204742864,48.52963269851785,48.52963269861785,49.06388986595757,49.06388986605757,49.59568584581275,49.595685845912755,50.121078892342524,50.121078892442526,50.63979340381218,50.63979340391218,51.15067569854144,51.15067569864144,51.64370156459746,51.64370156469746,52.130914156853535,52.13091415695354,52.601673966480675,52.60167396658068,53.04463645695639,53.04463645705639,53.483503377467216,53.48350337756722,53.90582778455174,53.90582778465174,54.324306586035426,54.32430658613543,54.73885646046359,54.73885646056359,55.15179118053939,55.15179118063939,55.5583614233458,55.5583614234458,55.96476502324284,55.96476502334284,56.35406849690147,56.35406849700147,56.73882133726576,56.73882133736576,57.10659582890234,57.106595829002345,57.47182581149969,57.47182581159969,57.834004946987044,57.834004947087045,58.1902618498491,58.1902618499491,58.544038337098605,58.54403833719861,58.884970501641305,58.88497050174131,59.224229827747635,59.22422982784764,59.560829276646714,59.560829276746716,59.88625083193268,59.88625083203268,60.204154228269,60.204154228369,60.521724338786576,60.52172433888658,60.83128918031173,60.83128918041173,61.125349821922825,61.12534982202283,61.41834651265102,61.41834651275102,61.70827953758387,61.70827953768387,61.983881272310924,61.983881272410926,62.256393703871964,62.256393703971966,62.52288135332501,62.522881353425014,62.78907417301534,62.78907417311534,63.054843976089565,63.05484397618957,63.319312682602174,63.319312682702176,63.57983323403125,63.57983323413125,63.832002411963835,63.83200241206384,64.07961454725941,64.07961454735941,64.32013154022144,64.32013154032144,64.55843730996298,64.55843731006298,64.79303207029972,64.79303207039972,65.02637059947351,65.02637059957351,65.25663905350929,65.25663905360929,65.47380040178888,65.47380040188888,65.68272574473998,65.68272574483998,65.8906255928642,65.8906255929642,66.09698719874808,66.09698719884808,66.3018938838463,66.3018938839463,66.50600581045369,66.50600581055369,66.70380453453302,66.70380453463302,66.88880380130422,66.88880380140422,67.07229046320576,67.07229046330576,67.25571944102327,67.25571944112328,67.43578992328273,67.43578992338273,67.60702192200291,67.60702192210292,67.77417757878621,67.77417757888621,67.93716075349258,67.93716075359258,68.09639446273813,68.09639446283813,68.25544871038898,68.25544871048898,68.41395175457038,68.41395175467038,68.57141648523954,68.57141648533954,68.72192707911385,68.72192707921386,68.87102120825851,68.87102120835851,69.01874373807222,69.01874373817222,69.16346028617458,69.16346028627459,69.3036326103253,69.3036326104253,69.44347805799994,69.44347805809994,69.58274666483445,69.58274666493445,69.71982968581915,69.71982968591915,69.84811908866325,69.84811908876326,69.9759406094926,69.9759406095926,70.10306992131378,70.10306992141378,70.22946215872814,70.22946215882814,70.34825932508083,70.34825932518083,70.46587076303993,70.46587076313993,70.583289920719,70.583289920819,70.6981902067295,70.69819020682951,70.81075749200883,70.81075749210883,70.9224723347133,70.9224723348133,71.03343728432561,71.03343728442562,71.14321009620167,71.14321009630167,71.25116906410265,71.25116906420266,71.35881397421291,71.35881397431291,71.46020336587912,71.46020336597913,71.5614004772653,71.5614004773653,71.66247581114077,71.66247581124077,71.7602887895982,71.76028878969821,71.85779411960756,71.85779411970756,71.95216528105225,71.95216528115225,72.04399193345769,72.04399193355769,72.13465849484022,72.13465849494023,72.2235304402757,72.2235304403757,72.31057572305076,72.31057572315076,72.38914785481865,72.38914785491865,72.466822678613,72.466822678713,72.54443340898067,72.54443340908067,72.6218710870963,72.6218710871963,72.69856528146244,72.69856528156244,72.77377250832959,72.77377250842959,72.84754404243908,72.84754404253908,72.92004011735763,72.92004011745763,72.9921516317161,72.9921516318161,73.06332738204502,73.06332738214502,73.13254828286018,73.13254828296019,73.20071164213512,73.20071164223512,73.26879167995537,73.26879168005537,73.33676275895026,73.33676275905026,73.40332378255818,73.40332378265818,73.46619943413195,73.46619943423195,73.52572299949033,73.52572299959033,73.5845864025539,73.5845864026539,73.64096939000491,73.64096939010491,73.69651275356641,73.69651275366641,73.75017817972618,73.75017817982618,73.80145292107073,73.80145292117074,73.85047157379614,73.85047157389614,73.89935563032552,73.89935563042552,73.94806663460287,73.94806663470287,73.99664304268417,73.99664304278417,74.04509767325479,74.04509767335479,74.09318697129332,74.09318697139332,74.13993671671423,74.13993671681423,74.18650059119777,74.18650059129777,74.23179541583302,74.23179541593302,74.27700050967093,74.27700050977093,74.32130188619263,74.32130188629263,74.36504564990221,74.36504565000222,74.40609108034855,74.40609108044855,74.44653403258407,74.44653403268407,74.48585534985268,74.48585534995269,74.52496515881326,74.52496515891326,74.56392755289247,74.56392755299247,74.60248615838357,74.60248615848357,74.64101912650402,74.64101912660402,74.67921880880571,74.67921880890572,74.71739285373675,74.71739285383676,74.75545793984243,74.75545793994243,74.79318333078669,74.79318333088669,74.83063311999622,74.83063312009622,74.86746761230962,74.86746761240963,74.90359707692954,74.90359707702954,74.93965603878011,74.93965603888012,74.97570218194534,74.97570218204534,75.01053695934631,75.01053695944631,75.04354507408688,75.04354507418688,75.07634168051939,75.0763416806194,75.10909342155324,75.10909342165324,75.14156315150969,75.14156315160969,75.17384701052876,75.17384701062876,75.20460544599283,75.20460544609283,75.2350626423515,75.2350626424515,75.26538524251414,75.26538524261414,75.29479771601792,75.29479771611793,75.32255016977066,75.32255016987067,75.3500462498167,75.3500462499167,75.37718340667331,75.37718340677331,75.40365399189244,75.40365399199244,75.42909908228468,75.42909908238468,75.45449289793558,75.45449289803558,75.47980980147446,75.47980980157446,75.50490237801995,75.50490237811995,75.5297449902014,75.5297449903014,75.55422867919343,75.55422867929343,75.57843676645074,75.57843676655074,75.60206160352524,75.60206160362524,75.62559030045973,75.62559030055974,75.6490228572542,75.6490228573542,75.67236568325131,75.67236568335132,75.69558032239506,75.69558032249506,75.71860268125877,75.71860268135877,75.74159940275182,75.74159940285182,75.7642628384261,75.76426283852611,75.7868301339604,75.7868301340604,75.80931410803998,75.80931410813999,75.83096486757273,75.83096486767273,75.85245539353876,75.85245539363876,75.87388182607813,75.87388182617813,75.8941609862799,75.8941609863799,75.9140940419776,75.91409404207761,75.93380277068192,75.93380277078192,75.95328076305019,75.95328076315019,75.97274593673312,75.97274593683312,75.99197396473734,75.99197396483734,76.01109944325886,76.01109944335886,76.02982754253496,76.02982754263496,76.04847872969904,76.04847872979904,76.06704659540846,76.06704659550846,76.08543499952314,76.08543499962315,76.10324015345505,76.10324015355505,76.12090430184826,76.12090430194826,76.13853640352811,76.13853640362811,76.15591854084393,76.15591854094393,76.17307635116634,76.17307635126635,76.19012520266341,76.19012520276341,76.20646261712433,76.20646261722433,76.2222872841718,76.2222872842718,76.23743256089645,76.23743256099645,76.25231505457171,76.25231505467171,76.26706936139361,76.26706936149361,76.28179803084484,76.28179803094484,76.29646260686938,76.29646260696938,76.31104386143925,76.31104386153925,76.32522132742102,76.32522132752102,76.33907832626939,76.33907832636939,76.35290968774709,76.35290968784709,76.36645903814738,76.36645903824738,76.37980969892497,76.37980969902497,76.3930706289052,76.39307062900521,76.40620978137476,76.40620978147476,76.41927843107496,76.41927843117496,76.43221889392179,76.43221889402179,76.4450824446566,76.44508244475661,76.45772166839804,76.45772166849804,76.47022629594345,76.47022629604345,76.48264760203418,76.48264760213418,76.49488944653018,76.49488944663018,76.50708642562752,76.50708642572752,76.51925776735419,76.51925776745419,76.53126246617148,76.53126246627149,76.54322870893277,76.54322870903277,76.55495780601535,76.55495780611535,76.56660358164324,76.56660358174324,76.57807630501908,76.57807630511908,76.58942725088423,76.58942725098423,76.60075255937872,76.60075255947872,76.61205223050253,76.61205223060253,76.62292247566756,76.62292247576757,76.63368376200727,76.63368376210727,76.64397716633218,76.64397716643218,76.6540718810344,76.6540718811344,76.66348720541379,76.66348720551379,76.6727999803105,76.6727999804105,76.68206788980854,76.68206788990854,76.69098969480248,76.69098969490248,76.69987304374042,76.69987304384043,76.70868588990903,76.70868589000904,76.7170244447202,76.7170244448202,76.72534377150335,76.72534377160335,76.73365027960116,76.73365027970117,76.74187987558697,76.74187987568698,76.74998769406207,76.74998769416207,76.75775581737578,76.75775581747578,76.7654406192348,76.7654406193348,76.77295877818445,76.77295877828445,76.78029747553938,76.78029747563939,76.78752721406897,76.78752721416897,76.79474413391321,76.79474413401321,76.80124320737865,76.80124320747865,76.80769741544539,76.80769741554539,76.81414521416947,76.81414521426947,76.82032382050149,76.82032382060149,76.82634219326681,76.82634219336681,76.83232210997612,76.83232211007612,76.83823152391608,76.83823152401608,76.84388456414932,76.84388456424932,76.84944146424253,76.84944146434253,76.85484454011171,76.85484454021172,76.86016429452621,76.86016429462622,76.86543918354204,76.86543918364204,76.87032951199778,76.87032951209778,76.87491219200545,76.87491219210546,76.87940514121577,76.87940514131577,76.88371862883137,76.88371862893138,76.88757705311755,76.88757705321756,76.89137779331972,76.89137779341972,76.89486447573115,76.89486447583116,76.90179297515533,76.90179297525533,76.90503610254538,76.90503610264538,76.9080228562287,76.9080228563287,76.913964316882,76.913964316982,76.9168164743693,76.9168164744693,76.9195853104019,76.9195853105019,76.92209136338512,76.92209136348512,76.92455896031232,76.92455896041233,76.92702014789687,76.92702014799687,76.92911600294933,76.92911600304933,76.93111571786176,76.93111571796176,76.93310902343153,76.93310902353153,76.93499337017595,76.93499337027595,76.93683926086435,76.93683926096435,76.93864669549674,76.93864669559674,76.94031312459045,76.94031312469045,76.94144116890003,76.94144116900003,76.94233206753088,76.94233206763089,76.942851224287,76.942851224387,76.9329936552635,76.9329936553635],[0.0,1e-10,2.320509034688136,2.320509034788136,4.361128466277445,4.361128466377445,6.318133179460175,6.318133179560175,8.213850150834457,8.213850150934457,9.987723983802562,9.987723983902562,11.756293090157364,11.756293090257364,13.169292676159431,13.169292676259431,14.551903120026221,14.551903120126221,15.861750291043538,15.861750291143538,17.097929902081624,17.097929902181622,18.311171187476106,18.311171187576104,19.50916058782994,19.509160587929937,20.637328832615623,20.63732883271562,21.74503598903571,21.745035989135708,22.846343085136024,22.846343085236022,23.920043950901793,23.92004395100179,24.986192108668313,24.98619210876831,26.042100169923195,26.042100170023193,27.08719499493631,27.08719499503631,27.96645502314625,27.966455023246247,28.823865691199828,28.823865691299826,29.653026938777277,29.653026938877275,30.424702271422802,30.4247022715228,31.19423151418995,31.19423151428995,31.961863027628457,31.961863027728455,32.69992689921153,32.699926899311535,33.42398705672179,33.423987056821794,34.12459306283134,34.12459306293134,34.77962809217641,34.77962809227641,35.432427876573975,35.43242787667398,36.08383302096156,36.083833021061565,36.731990373545095,36.7319903736451,37.37886771406466,37.37886771416466,38.01223169339164,38.01223169349164,38.644035459008826,38.64403545910883,39.27276337474098,39.272763374840984,39.89596367618701,39.89596367628701,40.51751460885411,40.51751460895411,41.13256995791308,41.13256995801308,41.74488697270588,41.74488697280588,42.35314743185321,42.35314743195321,42.95516066794214,42.95516066804214,43.52658097888063,43.52658097898063,44.09712247556626,44.09712247566626,44.66753023964819,44.66753023974819,45.20844041228615,45.20844041238615,45.736919820999546,45.73691982109955,46.25230617098911,46.25230617108911,46.75881522338089,46.758815223480894,47.252600573763814,47.252600573863816,47.74167981014046,47.74167981024046,48.22456276921249,48.22456276931249,48.69535884819802,48.69535884829802,49.16395789155139,49.16395789165139,49.632200314628236,49.63220031472824,50.07817944329919,50.07817944339919,50.506887961435595,50.5068879615356,50.91852328383338,50.91852328393338,51.32296251850843,51.322962518608435,51.726720353726556,51.72672035382656,52.12662541631437,52.12662541641437,52.52036604269369,52.52036604279369,52.912954021393524,52.912954021493526,53.298014764971,53.298014765071,53.68020980989783,53.680209809997834,54.056832663003966,54.05683266310397,54.42455274563543,54.42455274573543,54.790234998115324,54.790234998215325,55.153968575512785,55.15396857561279,55.516957071261075,55.51695707136108,55.87983093906334,55.87983093916334,56.23388482324084,56.233884823340844,56.56975107331559,56.56975107341559,56.89572111071678,56.89572111081678,57.22113711304551,57.22113711314551,57.54437518439975,57.54437518449975,57.86369680093144,57.863696801031445,58.178643450856484,58.178643450956486,58.4893297621209,58.489329762220905,58.795424587325066,58.79542458742507,59.09470541795992,59.094705418059924,59.38732509128683,59.387325091386835,59.67544880184184,59.67544880194184,59.959630584697415,59.95963058479742,60.24353216590714,60.24353216600714,60.521116473647005,60.52111647374701,60.79624901698576,60.79624901708576,61.07052185072932,61.07052185082932,61.343317257613,61.343317257713004,61.613992047495195,61.6139920475952,61.87914559131049,61.87914559141049,62.14177732031321,62.14177732041321,62.393493921566595,62.3934939216666,62.644350813224776,62.64435081332478,62.89150776951402,62.89150776961402,63.13559524413746,63.135595244237464,63.37484287215093,63.37484287225093,63.60891950615479,63.60891950625479,63.83672981244257,63.83672981254257,64.05808274443757,64.05808274453757,64.27621972572462,64.27621972582462,64.49129359356509,64.49129359366509,64.70196702192202,64.70196702202202,64.91209915164494,64.91209915174494,65.12112321122294,65.12112321132294,65.32477249377618,65.32477249387618,65.52763211714569,65.52763211724569,65.72947919365863,65.72947919375864,65.9271359819224,65.9271359820224,66.11704901561022,66.11704901571022,66.30689199888658,66.30689199898659,66.49171045719547,66.49171045729547,66.67564373303227,66.67564373313228,66.85539308883914,66.85539308893914,67.03465845998501,67.03465846008501,67.39152072884015,67.39152072894015,67.56716895146697,67.56716895156697,67.7399068979086,67.7399068980086,67.91226275119679,67.9122627512968,68.07830133101513,68.07830133111513,68.24131500670224,68.24131500680224,68.40370459690541,68.40370459700542,68.56605597779325,68.56605597789326,68.72408970604747,68.72408970614747,68.87861454550942,68.87861454560942,69.03016542659395,69.03016542669396,69.17670451914945,69.17670451924945,69.32274689060552,69.32274689070552,69.46738188561315,69.46738188571315,69.60692230524187,69.60692230534187,69.74225970018297,69.74225970028297,69.8734577526287,69.8734577527287,70.00060561764818,70.00060561774818,70.1269319823878,70.1269319824878,70.25089573779545,70.25089573789545,70.37199379455247,70.37199379465247,70.48991410991687,70.48991411001687,70.60701292500141,70.60701292510142,70.72406079433219,70.72406079443219,70.8407202022903,70.8407202023903,70.9548960047512,70.95489600485121,71.06535276718549,71.06535276728549,71.17430026166376,71.17430026176376,71.27643376157273,71.27643376167273,71.37715988503327,71.37715988513327,71.4776949619171,71.4776949620171,71.57593111166118,71.57593111176118,71.67281083071063,71.67281083081063,71.76957592181407,71.76957592191407,71.86081339863135,71.86081339873135,71.94968826611667,71.94968826621667,72.03803457140643,72.03803457150643,72.12281467393093,72.12281467403093,72.20357006190609,72.2035700620061,72.28370136439733,72.28370136449733,72.36176936386012,72.36176936396012,72.43887576222012,72.43887576232012,72.51596305592246,72.51596305602246,72.59231163619485,72.59231163629485,72.6672783128957,72.6672783129957,72.74058288437918,72.74058288447918,72.80962074898282,72.80962074908282,72.87837204372138,72.87837204382139,72.9460025318766,72.9460025319766,73.01274783755971,73.01274783765972,73.07900279036262,73.07900279046262,73.14513037878105,73.14513037888105,73.20802928005307,73.20802928015307,73.27050151063712,73.27050151073712,73.33120337627699,73.33120337637699,73.3897591520385,73.3897591521385,73.44718775299741,73.44718775309741,73.5029415123005,73.5029415124005,73.55677206939805,73.55677206949805,73.61041157991889,73.61041158001889,73.66189226412291,73.66189226422291,73.71285712256982,73.71285712266982,73.76219808511469,73.76219808521469,73.81088948929875,73.81088948939875,73.85928795539851,73.85928795549852,73.9075526888946,73.9075526889946,73.95569642622542,73.95569642632542,74.00313965944164,74.00313965954165,74.04958945045898,74.04958945055898,74.09586729955727,74.09586729965727,74.14147648563709,74.14147648573709,74.18650616376755,74.18650616386755,74.23149763258266,74.23149763268266,74.27538103125286,74.27538103135286,74.31910522444247,74.31910522454247,74.36184871187164,74.36184871197165,74.40385985409009,74.40385985419009,74.44564174041648,74.44564174051648,74.48737268098908,74.48737268108908,74.5289444160811,74.5289444161811,74.57050341473467,74.57050341483468,74.61128549064296,74.61128549074296,74.65180010134384,74.65180010144384,74.69218734766025,74.69218734776025,74.73237081096151,74.73237081106151,74.77142709946018,74.77142709956019,74.81015224055923,74.81015224065924,74.84869907152,74.84869907162,74.88701027836952,74.88701027846952,74.92529601234212,74.92529601244212,74.96326970357276,74.96326970367276,75.00067662329252,75.00067662339252,75.0378797599971,75.0378797600971,75.07454159806768,75.07454159816768,75.1111906996998,75.1111906997998,75.14766149119367,75.14766149129368,75.18389029035704,75.18389029045704,75.21978794212079,75.21978794222079,75.2556792256653,75.2556792257653,75.29149409057914,75.29149409067914,75.32695870343566,75.32695870353567,75.36233416122306,75.36233416132306,75.43249920062928,75.43249920072928,75.46657554169505,75.46657554179505,75.49963296768505,75.49963296778505,75.53242929668686,75.53242929678686,75.5650154744543,75.5650154745543,75.62986305080878,75.62986305090878,75.66192703459987,75.66192703469987,75.69248811865418,75.69248811875418,75.72274352818577,75.72274352828578,75.75288430977133,75.75288430987133,75.78266847108036,75.78266847118036,75.81230616334724,75.81230616344725,75.84153628958381,75.84153628968382,75.86911067882224,75.86911067892224,75.89643033929171,75.89643033939171,75.92339337948466,75.92339337958467,75.94961770624768,75.94961770634768,75.97540262588426,75.97540262598426,76.00060166935225,76.00060166945225,76.02553324761286,76.02553324771286,76.0502037288853,76.0502037289853,76.07425649289304,76.07425649299304,76.09827741580466,76.09827741590466,76.12194808665897,76.12194808675898,76.16921300973695,76.16921300983695,76.19268626579533,76.19268626589533,76.21603215746924,76.21603215756924,76.23931436695094,76.23931436705094,76.26252652602115,76.26252652612115,76.28573231687214,76.28573231697214,76.30886805731168,76.30886805741169,76.33182548761296,76.33182548771296,76.3545855031183,76.3545855032183,76.37693795259332,76.37693795269332,76.39829059165024,76.39829059175024,76.41916561426537,76.41916561436537,76.43914271796997,76.43914271806997,76.45870588742504,76.45870588752504,76.47805890564572,76.47805890574573,76.49739281920874,76.49739281930874,76.51651658153737,76.51651658163738,76.53549387482386,76.53549387492386,76.55426101687597,76.55426101697597,76.57288805810516,76.57288805820517,76.59141320782678,76.59141320792678,76.60985557069847,76.60985557079847,76.62822788315871,76.62822788325872,76.64649830411138,76.64649830421138,76.6641892171147,76.6641892172147,76.68174002929509,76.68174002939509,76.6992462639409,76.69924626404091,76.71651687447546,76.71651687457546,76.73356459733718,76.73356459743718,76.7505040604721,76.7505040605721,76.7670614304536,76.7670614305536,76.78340864920072,76.78340864930072,76.79924641040995,76.79924641050995,76.81496954367316,76.81496954377316,76.8303806341944,76.8303806342944,76.84544147265834,76.84544147275834,76.86025395057256,76.86025395067256,76.87491359122541,76.87491359132541,76.88954139078214,76.88954139088214,76.90411187636586,76.90411187646586,76.91862504797658,76.91862504807658,76.93308090561426,76.93308090571426,76.9475176585943,76.9475176586943,76.96160415951701,76.96160415961701,76.97556329605527,76.97556329615527,76.98930591313992,76.98930591323992,77.00280653789406,77.00280653799406,77.0158486508641,77.0158486509641,77.02883344986112,77.02883344996113,77.04167814803525,77.04167814813525,77.05445916401712,77.05445916411712,77.0670809745184,77.0670809746184,77.07963273460823,77.07963273470823,77.09205713031358,77.09205713041358,77.10429684766143,77.10429684776143,77.11647288281706,77.11647288291707,77.12859160399968,77.12859160409968,77.1406848523054,77.1406848524054,77.15275899595345,77.15275899605345,77.16468667055935,77.16468667065935,77.17657613584991,77.17657613594992,77.18842739182513,77.18842739192513,77.20008123300441,77.20008123310441,77.22330612851309,77.22330612861309,77.23474981845798,77.23474981855799,77.24614893086833,77.24614893096833,77.25737610135964,77.25737610145964,77.26817660116295,77.26817660126295,77.27840396123614,77.27840396133614,77.28775887527568,77.28775887537569,77.29651517670821,77.29651517680821,77.30489575320654,77.30489575330654,77.31318080641651,77.31318080651651,77.32145949140727,77.32145949150727,77.32972543995957,77.32972544005958,77.33797865207343,77.33797865217343,77.34615544555659,77.34615544565659,77.35379094040576,77.35379094050576,77.36138185772036,77.36138185782036,77.36867346873144,77.36867346883145,77.37585682001573,77.37585682011573,77.38302743486156,77.38302743496156,77.38973316970406,77.38973316980406,77.39614596646229,77.39614596656229,77.40255239500128,77.40255239510128,77.40853215285229,77.40853215295229,77.42045345923897,77.42045345933897,77.42633769380163,77.42633769390163,77.43215824617205,77.43215824627205,77.43777501552731,77.43777501562731,77.44314342433285,77.44314342443285,77.44838446875391,77.44838446885392,77.4533580479676,77.4533580480676,77.45821699923525,77.45821699933525,77.46303774118755,77.46303774128755,77.46782664204373,77.46782664214373,77.47242449632321,77.47242449642322,77.47697777306813,77.47697777316813,77.48126358460564,77.48126358470564,77.48509725257829,77.48509725267829,77.48887360657791,77.48887360667791,77.49233791783558,77.49233791793559,77.49556023676274,77.49556023686274,77.49860424555166,77.49860424565166,77.50157183570988,77.50157183580988,77.50450121655275,77.50450121665276,77.51023898207328,77.51023898217328,77.5129900527779,77.5129900528779,77.51562649553648,77.51562649563648,77.51825657007585,77.51825657017585,77.52070833447696,77.52070833457697,77.52315373065885,77.52315373075885,77.52523613834498,77.52523613844498,77.52727396849654,77.52727396859655,77.5292544846751,77.52925448477511,77.5311394775653,77.5311394776653,77.53293531538638,77.53293531548638,77.53343203648582,77.53343203658582],[0.0,1e-10,3.228468897889293,3.228468897989293,5.616302978119297,5.616302978219297,7.728198879261874,7.728198879361874,9.766662449554666,9.766662449654666,11.697872320793696,11.697872320893696,13.519996096084915,13.519996096184915,15.095753459185211,15.095753459285211,16.558155631446244,16.558155631546242,17.82014426681674,17.82014426691674,19.04070903623136,19.040709036331357,20.22999310122063,20.229993101320627,21.402532177571736,21.402532177671734,22.563979404638655,22.563979404738653,23.710013704639323,23.71001370473932,24.797404806169123,24.79740480626912,25.878668424468113,25.87866842456811,26.954038482544046,26.954038482644044,27.94036184900283,27.94036184910283,28.89143431776582,28.89143431786582,29.82085591258921,29.820855912689208,30.71193362772544,30.711933627825438,31.600373211163145,31.600373211263143,32.484959562834284,32.484959562934286,33.351754770193715,33.35175477029372,34.20328000343607,34.20328000353607,35.050393188837795,35.0503931889378,35.876771698746936,35.87677169884694,36.67533936217903,36.67533936227903,37.47266593409778,37.472665934197785,38.268478504327476,38.26847850442748,39.059495652898285,39.05949565299829,39.84541847818918,39.84541847828918,40.628469248773804,40.628469248873806,41.40686105278739,41.40686105288739,42.15338734485625,42.15338734495625,42.86366206858506,42.86366206868506,43.52335094188774,43.523350941987744,44.182377033335136,44.18237703343514,44.840909287321715,44.84090928742172,45.49617311706112,45.49617311716112,46.147837131625685,46.14783713172569,46.788760181417686,46.78876018151769,47.42229516288157,47.42229516298157,48.04684360213101,48.046843602231014,48.667649273256444,48.667649273356446,49.28765570743873,49.28765570753873,49.88657658161681,49.88657658171681,50.476445934967195,50.4764459350672,51.05904418149333,51.05904418159333,51.63739282671198,51.63739282681198,52.208262433543936,52.20826243364394,52.775298302193306,52.77529830229331,53.33081346271095,53.33081346281095,53.87730309384621,53.87730309394621,54.41279184575585,54.41279184585585,54.94534356434595,54.94534356444595,55.469662492635486,55.46966249273549,55.98680778202066,55.986807782120664,56.49265329055921,56.49265329065921,56.99835584614858,56.99835584624858,57.50217402195329,57.50217402205329,58.00152166916542,58.00152166926542,58.48142771528893,58.48142771538893,58.95931942440125,58.95931942450125,59.40461136323896,59.404611363338965,59.83293738615341,59.83293738625341,60.24911240838747,60.24911240848747,60.66394887118828,60.663948871288284,61.076121210845265,61.07612121094527,61.485629427358425,61.48562942745843,61.8932467662256,61.8932467663256,62.294853583365835,62.29485358346584,62.68935174021498,62.68935174031498,63.07738452504435,63.07738452514435,63.462863650372434,63.462863650472435,63.835691439697996,63.835691439798,64.20605004171952,64.20605004181952,64.57099592525614,64.57099592535614,64.93197161552233,64.93197161562233,65.27930829486436,65.27930829496437,65.62188204185415,65.62188204195415,65.94778227668037,65.94778227678037,66.27000472199583,66.27000472209583,66.59134995603222,66.59134995613222,66.91107072473702,66.91107072483702,67.20966694626964,67.20966694636964,67.50432546383846,67.50432546393846,67.79440298917216,67.79440298927216,68.07373305187197,68.07373305197197,68.34152291285608,68.34152291295608,68.60915682516834,68.60915682526834,68.86845398139883,68.86845398149883,69.12503503159488,69.12503503169488,69.38108975502348,69.38108975512348,69.63346668894134,69.63346668904134,69.88431012758616,69.88431012768616,70.1328208340148,70.1328208341148,70.37689999901882,70.37689999911882,70.6198615318747,70.6198615319747,70.85763127353079,70.85763127363079,71.09015074323513,71.09015074333513,71.31348873488524,71.31348873498524,71.53347383009093,71.53347383019093,71.74534309649994,71.74534309659994,71.95606224145416,71.95606224155416,72.16387684239548,72.16387684249548,72.37136655027048,72.37136655037048,72.57795955328243,72.57795955338243,72.77922431000673,72.77922431010673,72.97453702575602,72.97453702585602,73.1683162462323,73.1683162463323,73.36090635808583,73.36090635818583,73.54775236052681,73.54775236062682,73.7279055658016,73.72790556590161,73.90530367787399,73.90530367797399,74.07809480626594,74.07809480636594,74.24977480037109,74.24977480047109,74.41929100665452,74.41929100675452,74.5855647801361,74.5855647802361,74.74686768970295,74.74686768980295,74.90606529220005,74.90606529230006,75.05933684516746,75.05933684526747,75.20663686357591,75.20663686367591,75.352078493645,75.352078493745,75.49343946680109,75.4934394669011,75.63400120301405,75.63400120311405,75.772990456786,75.772990456886,75.90747019479744,75.90747019489744,76.04101424077786,76.04101424087786,76.1706010892105,76.17060108931051,76.29981106168621,76.29981106178622,76.42882609832213,76.42882609842214,76.54908851575136,76.54908851585137,76.66849971334685,76.66849971344685,76.78776146013182,76.78776146023182,76.90689324969026,76.90689324979026,77.02340640113417,77.02340640123417,77.13973761246092,77.13973761256092,77.25297584179631,77.25297584189632,77.36568124650293,77.36568124660293,77.46845142124147,77.46845142134147,77.5673098834615,77.5673098835615,77.66033976407175,77.66033976417175,77.75220002964325,77.75220002974325,77.83498278294174,77.83498278304174,77.9146075756356,77.9146075757356,77.99335515705039,77.99335515715039,78.07203126199059,78.07203126209059,78.14966121125724,78.14966121135724,78.22595909895196,78.22595909905196,78.3007559806803,78.3007559807803,78.37471463829752,78.37471463839752,78.44823793920588,78.44823793930588,78.52070208871802,78.52070208881803,78.59156126648256,78.59156126658256,78.66203707042882,78.66203707052883,78.73223346633806,78.73223346643806,78.80124075362455,78.80124075372456,78.86935783390933,78.86935783400934,78.93308235993746,78.93308236003746,78.99676140093631,78.99676140103631,79.05840011347865,79.05840011357866,79.11679639321912,79.11679639331912,79.17141091766761,79.17141091776762,79.22341330186289,79.22341330196289,79.27310244742597,79.27310244752597,79.32234973841885,79.32234973851885,79.37110319195092,79.37110319205092,79.41955124600065,79.41955124610065,79.46558859349828,79.46558859359828,79.51141800943347,79.51141800953347,79.55714345958742,79.55714345968742,79.6027974332668,79.6027974333668,79.64771065075496,79.64771065085496,79.69255888962986,79.69255888972987,79.7354252808002,79.7354252809002,79.77505573702999,79.77505573712999,79.81454973817193,79.81454973827194,79.85388779064203,79.85388779074204,79.89297892438172,79.89297892448172,79.93095892383461,79.93095892393461,79.96830863073882,79.96830863083882,80.00492407931316,80.00492407941316,80.03987607538792,80.03987607548792,80.07360647353333,80.07360647363333,80.10707045936435,80.10707045946435,80.14033301149426,80.14033301159427,80.17325117697386,80.17325117707387,80.20436943486605,80.20436943496605,80.2353772291157,80.2353772292157,80.26624856827748,80.26624856837748,80.29686649084753,80.29686649094754,80.32725698827116,80.32725698837116,80.3570821717594,80.3570821718594,80.38508795407624,80.38508795417624,80.41298327275052,80.41298327285052,80.43974146569269,80.4397414657927,80.46616176984588,80.46616176994588,80.49210773012224,80.49210773022224,80.51787175028147,80.51787175038147,80.54276505702296,80.54276505712296,80.56752840653792,80.56752840663792,80.59207082876777,80.59207082886778,80.61563857179867,80.61563857189867,80.63851754152897,80.63851754162897,80.66094815882775,80.66094815892775,80.68287194294307,80.68287194304307,80.70323624034003,80.70323624044003,80.72321716391875,80.72321716401875,80.74296416448972,80.74296416458972,80.7626916714767,80.7626916715767,80.78218525545594,80.78218525555594,80.80139293353682,80.80139293363682,80.8195934431121,80.8195934432121,80.83758602112493,80.83758602122494,80.85507826381564,80.85507826391564,80.87236257494389,80.8723625750439,80.88910106572074,80.88910106582074,80.90578107574564,80.90578107584564,80.92116151350525,80.92116151360526,80.93611959027865,80.93611959037865,80.95104517774543,80.95104517784543,80.96582781226302,80.96582781236302,80.98055846388999,80.98055846399,80.99499671175728,80.99499671185728,81.00925951736875,81.00925951746875,81.02321692349788,81.02321692359789,81.03702487881651,81.03702487891651,81.05066388974065,81.05066388984065,81.06397150973714,81.06397150983715,81.07709069175517,81.07709069185518,81.09013189943728,81.09013189953728,81.10309513278347,81.10309513288347,81.11531111207712,81.11531111217712,81.12746861061885,81.12746861071885,81.13960011771526,81.13960011781526,81.1500421808668,81.1500421809668,81.1602763124559,81.16027631255591,81.1699451301096,81.1699451302096,81.17925656539035,81.17925656549035,81.18819112471417,81.18819112481417,81.1966123529932,81.1966123530932,81.20495560693631,81.20495560703631,81.21289599347719,81.21289599357719,81.22073241423683,81.22073241433684,81.2285233499672,81.2285233500672,81.23596340118596,81.23596340128596,81.24329298876215,81.24329298886215,81.25060958061569,81.25060958071569,81.25726339061393,81.25726339071393,81.26385222199892,81.26385222209892,81.27039556835462,81.27039556845462,81.27693241684898,81.27693241694898,81.28328732522621,81.28328732532621,81.28954476568354,81.28954476578355,81.29578921041823,81.29578921051824,81.30189070220374,81.30189070230374,81.30789472606934,81.30789472616934,81.31337242316751,81.31337242326751,81.31872016303913,81.31872016313913,81.3236780312312,81.3236780313312,81.32850594219671,81.32850594229672,81.33287900286939,81.33287900296939,81.33720008065146,81.33720008075146,81.34111179316996,81.34111179326996,81.34464662973153,81.34464662983153,81.34803201548259,81.34803201558259,81.35131993331375,81.35131993341375,81.35436743027583,81.35436743037583,81.35717450636885,81.35717450646885,81.35980614020605,81.35980614030605,81.36230781681671,81.36230781691671,81.36480299556605,81.36480299566605,81.36723969356346,81.36723969366346,81.36965689797688,81.36965689807688,81.37199612805439,81.3719961281544,81.37412742656946,81.37412742666946,81.37625222722319,81.37625222732319,81.37803913908796,81.37803913918796,81.37978056592344,81.37978056602344,81.38104764888209,81.38104764898209,81.38207431097166,81.38207431107166],[0.0,1e-10,2.0354281455016205,2.0354281456016206,3.924649650338736,3.924649650438736,5.776259934647332,5.776259934747332,7.54096089054843,7.54096089064843,9.302419718634063,9.302419718734063,10.82731107562888,10.82731107572888,12.349721665547678,12.349721665647678,13.756817826804898,13.756817826904898,15.062050265797378,15.062050265897378,16.29504225996231,16.29504226006231,17.501500832357628,17.501500832457626,18.695358884515155,18.695358884615153,19.838922715159594,19.838922715259592,20.94304171482802,20.943041714928018,22.040118127656594,22.040118127756593,23.134421918459026,23.134421918559024,24.186501911586475,24.186501911686474,25.149210843248895,25.149210843348893,26.05419594151582,26.05419594161582,26.930211263646914,26.930211263746912,27.784426289938605,27.784426290038603,28.610680343074225,28.610680343174224,29.417843275668293,29.41784327576829,30.196011053435214,30.196011053535212,30.970327614795117,30.970327614895115,31.739352719015887,31.739352719115885,32.50613815372812,32.506138153828125,33.27093770584498,33.27093770594498,34.03361179256422,34.033611792664225,34.74755244728624,34.74755244738624,35.44539032236902,35.445390322469024,36.09523709217553,36.09523709227553,36.74413216105774,36.744132161157744,37.39035612267907,37.390356122779075,38.036021753091475,38.03602175319148,38.68044382762945,38.680443827729455,39.31501896993729,39.31501897003729,39.94611088686217,39.946110886962174,40.57543898474068,40.57543898484068,41.20184853311799,41.20184853321799,41.82112666923584,41.82112666933584,42.433895171031445,42.43389517113145,43.04656215806179,43.04656215816179,43.65257358329483,43.65257358339483,44.25844542572565,44.25844542582565,44.831147318608025,44.83114731870803,45.399661727423464,45.399661727523466,45.96555578636065,45.965555786460655,46.5235253489348,46.5235253490348,47.07996584535723,47.07996584545723,47.63528333468898,47.63528333478898,48.15472804384727,48.15472804394727,48.66822144989225,48.66822144999225,49.15552404650036,49.15552404660036,49.622314315853295,49.6223143159533,50.08882541960177,50.08882541970177,50.554816260178285,50.55481626027829,51.00397468374027,51.00397468384027,51.44843170473619,51.44843170483619,51.886728048415435,51.88672804851544,52.31203050214152,52.31203050224152,52.72214380911569,52.722143809215694,53.125093980466275,53.12509398056628,53.527745952193904,53.527745952293905,53.92889423646113,53.928894236561135,54.32003062700468,54.32003062710468,54.70081251149179,54.70081251159179,55.069234973308596,55.0692349734086,55.433362091620374,55.433362091720376,55.79575076957709,55.79575076967709,56.15738443146719,56.157384431567195,56.51891023391921,56.51891023401921,56.871927830107936,56.87192783020794,57.20659663247607,57.20659663257607,57.53066983122028,57.53066983132028,57.85266197727668,57.852661977376684,58.171760952523194,58.171760952623195,58.48988284815409,58.48988284825409,58.80575238493079,58.80575238503079,59.1108550119172,59.110855012017204,59.40905463486598,59.40905463496598,59.70463390793651,59.70463390803651,59.99612086703254,59.99612086713254,60.28328710393221,60.28328710403221,60.56815022459507,60.568150224695074,60.85130662826702,60.85130662836702,61.134266347081265,61.13426634718127,61.410665674190646,61.41066567429065,61.68410838376185,61.684108383861854,61.95738613183951,61.957386131939515,62.22916653712959,62.229166537229595,62.49336505838939,62.49336505848939,62.75656112134225,62.75656112144225,63.018221773470565,63.018221773570566,63.26902034571615,63.26902034581615,63.51979353927042,63.51979353937042,63.7698180614309,63.7698180615309,64.01606115858547,64.01606115868547,64.25923343409097,64.25923343419097,64.49736169469764,64.49736169479765,64.73064262526319,64.73064262536319,64.95747736823479,64.95747736833479,65.17372919692811,65.17372919702811,65.38308436625664,65.38308436635664,65.59123404774772,65.59123404784772,65.79772142495769,65.79772142505769,66.00056061529115,66.00056061539115,66.20314601871145,66.20314601881145,66.40526191634244,66.40526191644244,66.60643245772195,66.60643245782195,66.80752051835469,66.8075205184547,67.00444647361181,67.00444647371181,67.19350503456134,67.19350503466134,67.37681532192806,67.37681532202807,67.55543055140156,67.55543055150156,67.7340330915294,67.7340330916294,67.90900013412639,67.90900013422639,68.08073773825357,68.08073773835358,68.2460291547868,68.2460291548868,68.40844008985579,68.40844008995579,68.57021655764193,68.57021655774193,68.73194226804543,68.73194226814543,68.89156789174262,68.89156789184263,69.04904267135089,69.04904267145089,69.20003319532825,69.20003319542825,69.34411437059514,69.34411437069514,69.48440777618329,69.48440777628329,69.62021549808156,69.62021549818157,69.7509094136799,69.75090941377991,69.87743487922978,69.87743487932978,70.00340201357075,70.00340201367075,70.12671072999649,70.12671073009649,70.24419503676548,70.24419503686548,70.36084184672107,70.36084184682107,70.47724755910919,70.47724755920919,70.59338045056568,70.59338045066568,70.70905652557849,70.70905652567849,70.8228101647242,70.8228101648242,70.93474922744092,70.93474922754092,71.04472778625356,71.04472778635356,71.15337396377217,71.15337396387217,71.25372765390368,71.25372765400368,71.35390369319599,71.35390369329599,71.45177661625148,71.45177661635148,71.54834253670425,71.54834253680426,71.6447498403363,71.6447498404363,71.73564362328023,71.73564362338023,71.82418987727753,71.82418987737753,71.91220952343005,71.91220952353005,71.99655560401476,71.99655560411476,72.07701240015547,72.07701240025547,72.15683472901331,72.15683472911331,72.23459503920184,72.23459503930184,72.31142268248453,72.31142268258453,72.38824398109439,72.38824398119439,72.46431026363766,72.46431026373766,72.53899975217708,72.53899975227708,72.61198886839841,72.61198886849841,72.68077146653432,72.68077146663433,72.74926855439294,72.74926855449294,72.81625561011833,72.81625561021833,72.8827731600544,72.8827731601544,72.94865624270759,72.94865624280759,73.01131623156381,73.01131623166381,73.07355747201333,73.07355747211334,73.1321822489505,73.1321822490505,73.19052151561037,73.19052151571037,73.2477250858339,73.24772508593391,73.30327269644916,73.30327269654916,73.35690421587013,73.35690421597013,73.41034539510625,73.41034539520625,73.463393204627,73.463393204727,73.51416962127506,73.51416962137506,73.56332814635181,73.56332814645181,73.61184585947285,73.61184585957285,73.66006537297096,73.66006537307096,73.70815164833965,73.70815164843965,73.8042036502933,73.8042036503933,73.85150318623124,73.85150318633124,73.89778122984377,73.89778122994377,73.94400851607365,73.94400851617365,73.9894490628728,73.9894490629728,74.03431224444451,74.03431224454451,74.07909928994229,74.0790992900423,74.12282043040484,74.12282043050485,74.16638295404668,74.16638295414668,74.20968534610253,74.20968534620253,74.25154115275346,74.25154115285346,74.29315586183688,74.29315586193688,74.33473250288334,74.33473250298334,74.37615052710908,74.37615052720908,74.45897388621489,74.4589738863149,74.49960517100988,74.49960517110988,74.53991922216342,74.53991922226342,74.5799541077125,74.5799541078125,74.61915784112102,74.61915784122102,74.65810144294356,74.65810144304356,74.69693718532803,74.69693718542803,74.73559527687327,74.73559527697327,74.77417723234458,74.77417723244459,74.81232140539072,74.81232140549072,74.85037675301724,74.85037675311725,74.88795625018162,74.88795625028162,74.9254025092166,74.9254025093166,74.96279801086894,74.96279801096894,75.00013641046581,75.00013641056582,75.03720198913106,75.03720198923106,75.07372827060588,75.07372827070589,75.11024186273504,75.11024186283504,75.1467110421544,75.1467110422544,75.18303429409868,75.18303429419868,75.21911644847549,75.21911644857549,75.25512881145117,75.25512881155117,75.29103965966159,75.2910396597616,75.32680458039697,75.32680458049697,75.3625631564595,75.3625631565595,75.39795374149799,75.39795374159799,75.43320474373422,75.43320474383422,75.46796720616265,75.46796720626266,75.50262815382582,75.50262815392583,75.5355633504796,75.53556335057961,75.5682384155474,75.5682384156474,75.60070410641184,75.60070410651184,75.63284621896202,75.63284621906202,75.6647916466545,75.66479164675451,75.69607088369999,75.69607088379999,75.7265189686049,75.72651896870491,75.75666250921405,75.75666250931405,75.78669184571227,75.78669184581227,75.81636588053209,75.81636588063209,75.84548792881581,75.84548792891582,75.87331566384249,75.87331566394249,75.90078809719074,75.90078809729074,75.92800674362586,75.92800674372586,75.95487008838256,75.95487008848256,75.98099745109114,75.98099745119114,76.00615407885692,76.00615407895692,76.03125994924008,76.03125994934008,76.05609934336444,76.05609934346444,76.0806786059028,76.0806786060028,76.1046424351768,76.1046424352768,76.12857454108664,76.12857454118664,76.15215768999091,76.15215769009092,76.17566470282124,76.17566470292124,76.19914633696024,76.19914633706024,76.24609691589261,76.24609691599261,76.26948337993919,76.26948338003919,76.2926795038009,76.2926795039009,76.3158058362615,76.3158058363615,76.3387101051731,76.33871010527311,76.36157630604772,76.36157630614773,76.38384610767646,76.38384610777646,76.40511979567108,76.40511979577109,76.42580342909264,76.42580342919264,76.44604293541617,76.44604293551618,76.46623802902992,76.46623802912993,76.48614126769355,76.48614126779356,76.50563210262331,76.50563210272331,76.52491356334971,76.52491356344972,76.54417599005764,76.54417599015764,76.56312752779696,76.56312752789697,76.582015618808,76.582015618908,76.60071336963416,76.60071336973417,76.61926519298528,76.61926519308528,76.63772184624396,76.63772184634396,76.65609601875587,76.65609601885588,76.67429888510141,76.67429888520141,76.69192438621953,76.69192438631953,76.70941030453541,76.70941030463541,76.7268518101415,76.7268518102415,76.74405856285293,76.74405856295293,76.76104325201536,76.76104325211536,76.7779200817397,76.7779200818397,76.7944162310943,76.7944162311943,76.81070300624557,76.81070300634558,76.82648220757054,76.82648220767054,76.8421472047846,76.8421472048846,76.85750131303004,76.85750131313004,76.87250646426992,76.87250646436992,76.90226932450933,76.90226932460934,76.91687476136099,76.91687476146099,76.93144847484851,76.93144847494851,76.94596508628057,76.94596508638057,76.96042459565717,76.96042459575717,76.97485872634246,76.97485872644246,76.98924209964511,76.98924209974511,77.00327651594219,77.00327651604219,77.0171840387827,77.0171840388827,77.03104080424058,77.03104080434058,77.04473260820491,77.04473260830491,77.05836096544095,77.05836096554096,77.07160864230727,77.07160864240727,77.08474845973551,77.08474845983551,77.09768524763321,77.09768524773321,77.1104824527287,77.1104824528287,77.1232162110959,77.1232162111959,77.13572790591408,77.13572790601408,77.14805560522025,77.14805560532025,77.16018027499588,77.16018027509588,77.1722541873889,77.1722541874889,77.18430272109059,77.18430272119059,77.1963322207738,77.1963322208738,77.20821579298196,77.20821579308196,77.22006129715314,77.22006129725314,77.23167839310248,77.23167839320249,77.24328914437899,77.243289144479,77.25481741490874,77.25481741500874,77.26621244730907,77.26621244740907,77.27756941167245,77.27756941177245,77.28881851659773,77.28881851669773,77.30000417479472,77.30000417489472,77.31076473991219,77.31076474001219,77.32095428447508,77.32095428457508,77.33106769296403,77.33106769306403,77.3405910468799,77.3405910469799,77.350031920049,77.35003192014901,77.35935224443436,77.35935224453436,77.36806982490099,77.36806982500099,77.37641941434356,77.37641941444356,77.3846738336937,77.3846738337937,77.392921908371,77.392921908471,77.40115729370264,77.40115729380264,77.40937998968863,77.40937998978863,77.41752654960068,77.41752654970068,77.42555256072899,77.42555256082899,77.4331598234506,77.4331598235506,77.44042447383946,77.44042447393946,77.44758126479023,77.44758126489023,77.45472536639534,77.45472536649534,77.46140630688396,77.46140630698396,77.46790959653337,77.46790959663338,77.4939164104582,77.4939164105582,77.5003054959967,77.5003054960967,77.50668823686237,77.50668823696238,77.5127981567964,77.51279815689641,77.51875580458255,77.51875580468256,77.52467538433173,77.52467538443173,77.53053151735261,77.53053151745262,77.53620999953431,77.53620999963431,77.54180600096922,77.54180600106922,77.5471545601638,77.5471545602638,77.55210974964302,77.55210974974302,77.55691266697434,77.55691266707434,77.56648677827285,77.56648677837285,77.57106763205516,77.57106763215516,77.57560407312768,77.57560407322768,77.5798740379414,77.5798740380414,77.58369353098428,77.58369353108428,77.5874559219717,77.5874559220717,77.59104066211991,77.59104066221991,77.59449216413871,77.59449216423872,77.59770256859004,77.59770256869004,77.60065918612821,77.60065918622821,77.60357773562941,77.60357773572942,77.60931966453938,77.60931966463939,77.61211766525683,77.61211766535683,77.61485856391883,77.61485856401883,77.61748525846991,77.61748525856991,77.62010560834817,77.62010560844817,77.62254830738722,77.62254830748722,77.62498466175343,77.62498466185343,77.62705936976842,77.62705936986842,77.62912138843775,77.62912138853775,77.63115168374293,77.63115168384293,77.63302970690023,77.63302970700023,77.63472373454549,77.63472373464549,77.63587846500032,77.63587846510032,77.64099227130023,77.64099227140024],[0.0,1e-10,3.0467094982207885,3.0467094983207885,5.305126654098778,5.305126654198778,7.34173181315288,7.34173181325288,9.224063011162029,9.224063011262029,11.075502656394665,11.075502656494665,12.85382680043119,12.85382680053119,14.387680494386535,14.387680494486535,15.827896149622102,15.827896149722102,17.236212505488734,17.236212505588732,18.491744337097952,18.49174433719795,19.701300107188743,19.70130010728874,20.908605877026016,20.908605877126014,22.10151164524075,22.101511645340747,23.20335895249115,23.20335895259115,24.26448442416726,24.26448442426726,25.315488063716966,25.315488063816964,26.35306142006326,26.353061420163257,27.280181947062754,27.280181947162752,28.19567852909054,28.195678529190538,29.063671584639053,29.06367158473905,29.91742308928711,29.917423089387107,30.74603797138456,30.746037971484558,31.567154965313236,31.567154965413234,32.36831984431773,32.368319844417734,33.14225021321129,33.14225021331129,33.91552776512989,33.91552776522989,34.67990672449653,34.679906724596535,35.41966877968099,35.41966877978099,36.15733294730512,36.15733294740512,36.84775344763419,36.84775344773419,37.50182535231835,37.50182535241835,38.14980641124579,38.14980641134579,38.79215296249611,38.79215296259611,39.41689880753789,39.41689880763789,40.035471412447485,40.03547141254749,40.650995425464274,40.650995425564275,41.26572718487067,41.26572718497067,41.871268802396486,41.87126880249649,42.476569574824744,42.476569574924746,43.077890065114374,43.077890065214376,43.66943097683729,43.66943097693729,44.25174371850633,44.251743718606335,44.82380786747131,44.82380786757131,45.3813389162072,45.3813389163072,45.93843897897904,45.938438979079045,46.494404534580795,46.4944045346808,47.04661797708371,47.046617977183715,47.58232085434601,47.58232085444601,48.106044857019135,48.10604485711914,48.625579422600495,48.6255794227005,49.13852877617226,49.13852877627226,49.63890981846871,49.63890981856871,50.13331409952835,50.13331409962835,50.62735077491276,50.627350775012765,51.11980928110527,51.11980928120527,51.60658891341847,51.60658891351847,52.089172770611015,52.089172770711016,52.56976014870537,52.56976014880537,53.04770456875544,53.047704568855444,53.52298067864571,53.52298067874571,53.99250819633895,53.992508196438955,54.45870824886853,54.458708248968534,54.91941323035642,54.91941323045642,55.35778299806005,55.357782998160054,55.77179572076572,55.77179572086572,56.177626048183214,56.177626048283216,56.580357079476855,56.58035707957686,56.98163036412736,56.981630364227364,57.38129378944156,57.38129378954156,57.7730727068251,57.7730727069251,58.15340514404565,58.15340514414565,58.51711926953457,58.51711926963457,58.88064959218587,58.88064959228587,59.242855266800596,59.2428552669006,59.60404051876513,59.60404051886513,59.965029291834284,59.965029291934286,60.3173413033624,60.3173413034624,60.65150120016915,60.65150120026915,60.97716813827247,60.97716813837247,61.3009843719419,61.3009843720419,61.618703421825735,61.61870342192574,61.93280979524617,61.932809795346174,62.24616194322952,62.24616194332952,62.55821479529181,62.55821479539181,62.86623666098441,62.866236661084415,63.16494796224766,63.16494796234766,63.46293039018934,63.462930390289344,63.75411211313939,63.754112113239394,64.04238468083207,64.04238468093207,64.32972555827894,64.32972555837894,64.61305446344275,64.61305446354275,64.89589534038258,64.89589534048258,65.1651855115702,65.1651855116702,65.43384821789839,65.43384821799839,65.69534895158861,65.69534895168862,65.95045461413574,65.95045461423574,66.20398210749094,66.20398210759095,66.45522157241933,66.45522157251933,66.70583991051717,66.70583991061717,66.95560261471579,66.95560261481579,67.20239912139708,67.20239912149708,67.44475900786017,67.44475900796017,67.68613016181749,67.68613016191749,67.92513089297249,67.92513089307249,68.15686190499852,68.15686190509852,68.38359221223574,68.38359221233574,68.60144294100766,68.60144294110766,68.81606127504915,68.81606127514915,69.02520355213561,69.02520355223561,69.23043526540116,69.23043526550116,69.43381627423283,69.43381627433283,69.6356888321903,69.6356888322903,69.83718110841477,69.83718110851477,70.03083958093967,70.03083958103967,70.22032763045945,70.22032763055945,70.40814244035408,70.40814244045409,70.59191358782125,70.59191358792125,70.77489248167802,70.77489248177802,70.95727560081978,70.95727560091979,71.13776364932548,71.13776364942548,71.3177129653761,71.3177129654761,71.49612214040813,71.49612214050813,71.67316230120139,71.67316230130139,71.84766725044138,71.84766725054138,72.01302008597409,72.01302008607409,72.17627503394648,72.17627503404648,72.33845885503762,72.33845885513762,72.4959588727841,72.4959588728841,72.65207086220516,72.65207086230517,72.7960391882861,72.7960391883861,72.93743427464048,72.93743427474048,73.07527372679141,73.07527372689141,73.21098360123759,73.21098360133759,73.3414899539707,73.3414899540707,73.46791461610306,73.46791461620306,73.59377519366481,73.59377519376481,73.71107309420542,73.71107309430542,73.82735691012472,73.82735691022472,73.9434632612353,73.9434632613353,74.05704707685038,74.05704707695038,74.16749356816828,74.16749356826828,74.27736963688672,74.27736963698672,74.38588936742416,74.38588936752416,74.4867274069552,74.4867274070552,74.58686192528022,74.58686192538022,74.68361827421052,74.68361827431052,74.77994997520562,74.77994997530563,74.87537533807046,74.87537533817046,74.96611055956176,74.96611055966176,75.05574930205626,75.05574930215626,75.1446971994025,75.1446971995025,75.22482256054339,75.22482256064339,75.30252045662202,75.30252045672202,75.3792993385126,75.3792993386126,75.456033854201,75.456033854301,75.53186836978799,75.53186836988799,75.60621344858743,75.60621344868743,75.67918951314812,75.67918951324812,75.75086628178775,75.75086628188775,75.82192192359683,75.82192192369683,75.89258460761516,75.89258460771516,75.9612888407086,75.9612888408086,76.02969518644453,76.02969518654453,76.09700505318366,76.09700505328367,76.16400435650753,76.16400435660753,76.23073746261831,76.23073746271831,76.29291352596209,76.2929135260621,76.35191423683541,76.35191423693541,76.40263748198733,76.40263748208733,76.45313889612832,76.45313889622832,76.5011684790049,76.5011684791049,76.54875439985965,76.54875439995965,76.59595370095253,76.59595370105254,76.64163821314233,76.64163821324233,76.6865431477795,76.6865431478795,76.73137836409897,76.73137836419897,76.7760361156097,76.7760361157097,76.81971147264355,76.81971147274355,76.86152344918574,76.86152344928574,76.90305021442819,76.9030502145282,76.94282134567001,76.94282134577001,76.98170515286819,76.98170515296819,77.02034177693992,77.02034177703992,77.058471358701,77.058471358801,77.0963664333934,77.0963664334934,77.13421714188362,77.13421714198363,77.17204883628719,77.17204883638719,77.20987419266189,77.20987419276189,77.24762349268997,77.24762349278997,77.28492279266736,77.28492279276736,77.32196223346054,77.32196223356054,77.35845026574087,77.35845026584087,77.39482421350132,77.39482421360132,77.4303932315936,77.4303932316936,77.4656199961262,77.4656199962262,77.49852070405868,77.49852070415868,77.53093972179603,77.53093972189603,77.56306719020475,77.56306719030475,77.59517564452683,77.59517564462683,77.62558550710823,77.62558550720823,77.65569748233212,77.65569748243212,77.68563199274726,77.68563199284726,77.7152749538338,77.7152749539338,77.74436650640749,77.74436650650749,77.7716834108939,77.7716834109939,77.79887355480265,77.79887355490266,77.82570876909395,77.82570876919395,77.85185313823698,77.85185313833698,77.8769834227587,77.8769834228587,77.90206300304935,77.90206300314935,77.92670525934707,77.92670525944708,77.95125878324042,77.95125878334042,77.97519751833211,77.97519751843211,77.99845174630443,77.99845174640443,78.02162357990125,78.02162358000125,78.04476372335365,78.04476372345366,78.06764400762187,78.06764400772187,78.09005527775271,78.09005527785271,78.11223204081487,78.11223204091488,78.13412359257731,78.13412359267731,78.15559049640456,78.15559049650456,78.1768419072498,78.1768419073498,78.19672430385626,78.19672430395626,78.21619472858532,78.21619472868532,78.23541797018794,78.23541797028794,78.25447008501071,78.25447008511071,78.2729898054073,78.27298980550731,78.29098346940658,78.29098346950659,78.30784262623578,78.30784262633578,78.32361164209709,78.3236116421971,78.33913981286082,78.33913981296082,78.3541292511695,78.3541292512695,78.36906164721822,78.36906164731822,78.3839686911514,78.3839686912514,78.39854615758267,78.39854615768267,78.41310460992727,78.41310461002728,78.42752362563647,78.42752362573647,78.44189193711459,78.44189193721459,78.45606376969732,78.45606376979732,78.46997574309586,78.46997574319586,78.4835454629347,78.4835454630347,78.4969440559937,78.4969440560937,78.51031729693716,78.51031729703716,78.52342434066753,78.52342434076753,78.53634758156029,78.5363475816603,78.5490680055288,78.5490680056288,78.5617820914684,78.5617820915684,78.57442012106142,78.57442012116142,78.59959477178532,78.59959477188532,78.61184618161647,78.61184618171647,78.62367928154133,78.62367928164133,78.63469477574026,78.63469477584026,78.6448799881555,78.6448799882555,78.65486238364646,78.65486238374646,78.66402717341151,78.66402717351151,78.67273562509698,78.67273562519698,78.68094971052955,78.68094971062955,78.68908773961552,78.68908773971552,78.6969975996617,78.6969975997617,78.7046729526392,78.7046729527392,78.71231661547229,78.71231661557229,78.7199159121032,78.7199159122032,78.72717295517442,78.72717295527443,78.73432225175462,78.73432225185462,78.74145887227705,78.74145887237705,78.74810746457548,78.74810746467548,78.75453422586301,78.75453422596301,78.76091028291947,78.76091028301947,78.76728000194704,78.76728000204704,78.77338352376152,78.77338352386153,78.77947436951824,78.77947436961824,78.78542577863952,78.78542577873952,78.79128845535645,78.79128845545645,78.79663141370494,78.79663141380495,78.8018476114758,78.8018476115758,78.80680395006242,78.80680395016242,78.81175395062016,78.81175395072016,78.816589866658,78.81658986675801,78.82140043058033,78.82140043068033,78.82619831844488,78.82619831854488,78.83576240402957,78.83576240412957,78.84007860169899,78.840078601799,78.84434409513736,78.84434409523736,78.84815958852502,78.84815958862502,78.85160747623746,78.85160747633746,78.85501099774771,78.85501099784771,78.85822437839148,78.85822437849149,78.86119691393768,78.86119691403768,78.86398564664627,78.86398564674627,78.86670466103715,78.86670466113715,78.86913846412828,78.86913846422829,78.8714962108728,78.87149621097281,78.87377790127076,78.87377790137076,78.87585043671554,78.87585043681554,78.87785325384262,78.87785325394262,78.8798180427964,78.8798180428964,78.88168142328804,78.88168142338805,78.88338001502873,78.88338001512874,78.88481240955633,78.88481240965633,78.88604832518854,78.88604832528854,78.88693564923219,78.88693564933219],[0.0,1e-10,3.1137574681405327,3.1137574682405327,5.4269491848409395,5.4269491849409395,7.51674603276389,7.51674603286389,9.39356995350998,9.39356995360998,11.261429320387066,11.261429320487066,13.078843639914599,13.078843640014599,14.639148854473811,14.639148854573811,16.160357214153475,16.160357214253473,17.60001015637317,17.600010156473168,18.88310079254688,18.88310079264688,20.11926354376328,20.11926354386328,21.35310095188794,21.353100951987937,22.57228027807217,22.57228027817217,23.698419971471473,23.69841997157147,24.782722921055832,24.78272292115583,25.856850065634266,25.856850065734264,26.917238901862877,26.917238901962875,27.86472876848405,27.864728768584047,28.800352260330644,28.800352260430643,29.687389683845318,29.687389683945316,30.565669826412815,30.565669826512814,31.438146327169168,31.438146327269166,32.277290751184275,32.277290751284276,33.1005399330068,33.1005399331068,33.895587042903294,33.895587043003296,34.68649517164206,34.68649517174206,35.47680739073057,35.47680739083057,36.25799312397991,36.25799312407991,37.031315440757446,37.03131544085745,37.78732399128366,37.78732399138366,38.492919880854345,38.49291988095435,39.15511800241758,39.15511800251758,39.812620096845684,39.812620096945686,40.469072872107034,40.469072872207036,41.11218893030461,41.11218893040461,41.74040076996709,41.74040077006709,42.36317169543162,42.36317169553162,42.98201091266024,42.982010912760245,43.60066228880347,43.60066228890347,44.21518116106777,44.21518116116777,44.821350820949746,44.82135082104975,45.42590763840876,45.42590763850876,46.020988196973015,46.02098819707302,46.601617946518665,46.60161794661867,47.17094484454596,47.17094484464596,47.73911878694559,47.73911878704559,48.30317965730272,48.30317965740272,48.85047733010713,48.850477330207134,49.381400442087234,49.381400442187235,49.9056130932232,49.905613093323204,50.41699425504233,50.416994255142335,50.92222847927355,50.92222847937355,51.42711940772799,51.427119407827995,51.93164113129043,51.93164113139043,52.434919217321905,52.43491921742191,52.932393661542235,52.93239366164224,53.42559310174989,53.42559310184989,53.916771630969734,53.916771631069736,54.40243151864595,54.40243151874595,54.88779992877584,54.88779992887584,55.36763026552568,55.367630265625685,55.84409889457464,55.844098894674644,56.291665905586406,56.29166590568641,56.72592858592842,56.725928586028424,57.15993865239696,57.15993865249696,57.58727712169421,57.58727712179421,58.01042479160327,58.01042479170327,58.425171430899546,58.42517143099955,58.83674420358035,58.836744203680354,59.2464320881283,59.2464320882283,59.65487633514529,59.65487633524529,60.04355840452187,60.04355840462187,60.41527000342389,60.415270003523894,60.78470160018582,60.78470160028582,61.153809333007395,61.1538093331074,61.51793603842633,61.51793603852633,61.87800149003321,61.878001490133215,62.2324835273084,62.232483527408405,62.57398509785419,62.57398509795419,62.90492222666563,62.904922226765635,63.22961525870705,63.22961525880705,63.55062271910723,63.55062271920723,63.86953154117393,63.86953154127393,64.18542195131651,64.18542195141652,64.49230894391727,64.49230894401727,64.79001763244837,64.79001763254837,65.08366506716744,65.08366506726745,65.3772153427044,65.3772153428044,65.67057129987715,65.67057129997715,65.9597105485465,65.9597105486465,66.24594797631026,66.24594797641026,66.5279622182918,66.5279622183918,66.80320122664115,66.80320122674115,67.07777307527337,67.07777307537337,67.34499321245954,67.34499321255954,67.60570368444459,67.6057036845446,67.86480131400668,67.86480131410669,68.12155416864061,68.12155416874062,68.37769168178788,68.37769168188788,68.63293533045976,68.63293533055976,68.88061999476376,68.88061999486376,69.12546113367148,69.12546113377148,69.36888374852043,69.36888374862043,69.60570601626496,69.60570601636496,69.83842816652462,69.83842816662462,70.06105223912404,70.06105223922404,70.27112982267413,70.27112982277413,70.47896626775697,70.47896626785698,70.68601248482535,70.68601248492536,70.89232029210973,70.89232029220973,71.0982588945021,71.0982588946021,71.30334249609193,71.30334249619193,71.50348393395197,71.50348393405197,71.69806786659552,71.69806786669552,71.89106486593134,71.89106486603134,72.0830060688216,72.0830060689216,72.26999215342445,72.26999215352446,72.45637585109824,72.45637585119825,72.63882136325732,72.63882136335732,72.82115028439787,72.82115028449788,72.99946976995685,72.99946977005685,73.16845549675482,73.16845549685482,73.33530372154645,73.33530372164645,73.50105080894087,73.50105080904088,73.66205652824851,73.66205652834851,73.82301690660451,73.82301690670451,73.97014181742857,73.97014181752857,74.11518104447676,74.11518104457676,74.25967618010515,74.25967618020515,74.40054403960157,74.40054403970157,74.53926791981284,74.53926791991285,74.67262861317185,74.67262861327185,74.80185032537315,74.80185032547315,74.93047612792418,74.93047612802418,75.05887522571697,75.05887522581698,75.17875022459964,75.17875022469964,75.29756942703676,75.29756942713676,75.4136616950961,75.4136616951961,75.5265282783095,75.5265282784095,75.63881838370908,75.63881838380908,75.74972882872274,75.74972882882274,75.85991381851002,75.85991381861002,75.9629738016095,75.9629738017095,76.06530832948259,76.06530832958259,76.16417751319389,76.1641775132939,76.26264510561916,76.26264510571916,76.35764735388263,76.35764735398263,76.45037607728482,76.45037607738482,76.54199070873219,76.54199070883219,76.63287340767435,76.63287340777435,76.71473973451707,76.71473973461707,76.7941446954131,76.7941446955131,76.87261045088208,76.87261045098208,76.95103734267823,76.95103734277824,77.02855093816258,77.02855093826258,77.10452941856965,77.10452941866966,77.1791088067544,77.1791088068544,77.2523603527837,77.2523603528837,77.3249771254899,77.3249771255899,77.39719878418886,77.39719878428886,77.46741896373643,77.46741896383644,77.53732823390128,77.53732823400128,77.6061298893901,77.6061298894901,77.67460120365976,77.67460120375976,77.73814330875724,77.73814330885725,77.79829779703861,77.79829779713862,77.857545466287,77.857545466387,77.90938312858007,77.90938312868008,77.96099408611491,77.96099408621491,78.01007890491498,78.01007890501498,78.05871679147735,78.05871679157735,78.10696604131131,78.10696604141131,78.1527863115926,78.15278631169261,78.19842521806731,78.19842521816732,78.24306014632691,78.24306014642691,78.28579075461722,78.28579075471723,78.32823636264004,78.32823636274004,78.37011844740664,78.37011844750664,78.40985655288809,78.40985655298809,78.44934204449605,78.44934204459605,78.48830935379947,78.48830935389947,78.52698518555655,78.52698518565656,78.56555738085272,78.56555738095273,78.60282764310867,78.60282764320867,78.64000074618252,78.64000074628252,78.67635123484786,78.67635123494786,78.71233899590001,78.71233899600001,78.74603380025445,78.74603380035445,78.77967030909963,78.77967030919963,78.8130282949561,78.8130282950561,78.84615957605433,78.84615957615434,78.878973470491,78.878973470591,78.90974702210346,78.90974702220346,78.94004125508418,78.94004125518418,78.96977196480869,78.96977196490869,78.99768903646721,78.99768903656721,79.02547656254958,79.02547656264959,79.05290136101878,79.05290136111878,79.07962013609799,79.07962013619799,79.10593731989117,79.10593731999117,79.13161973036125,79.13161973046125,79.15725032260087,79.15725032270088,79.1824339826028,79.1824339827028,79.20738446056768,79.20738446066768,79.23184914262204,79.23184914272204,79.25553007394073,79.25553007404073,79.27917861886539,79.27917861896539,79.3011430713002,79.3011430714002,79.32286138714035,79.32286138724035,79.3443141345494,79.34431413464941,79.36463983544603,79.36463983554603,79.38453803594139,79.38453803604139,79.40418362256327,79.40418362266327,79.42363489082094,79.42363489092094,79.44255502221642,79.44255502231643,79.45978458384326,79.45978458394326,79.47589357623647,79.47589357633647,79.49121234061522,79.49121234071522,79.50609065003509,79.50609065013509,79.5208264593212,79.5208264594212,79.53551692765566,79.53551692775567,79.5500001230683,79.5500001231683,79.56432786378956,79.56432786388956,79.5785454907711,79.57854549087111,79.59271777680101,79.59271777690101,79.60653381249651,79.60653381259651,79.62025268900992,79.62025268910992,79.63394565640809,79.63394565650809,79.64761271469102,79.64761271479102,79.6612150001859,79.6612150002859,79.67473308105627,79.67473308115628,79.68812809362933,79.68812809372933,79.70133526511697,79.70133526521697,79.71433516368278,79.71433516378278,79.7272508576241,79.7272508577241,79.7401535970078,79.7401535971078,79.7526741769419,79.7526741770419,79.76482555198399,79.76482555208399,79.7769186315168,79.77691863161681,79.78841580139938,79.78841580149938,79.79988706216672,79.79988706226672,79.81056161764079,79.81056161774079,79.82097060468377,79.82097060478377,79.83117231880493,79.83117231890493,79.84053846395997,79.84053846405997,79.84945119959852,79.84945119969852,79.85776802558681,79.85776802568681,79.86585166953805,79.86585166963805,79.87369565417342,79.87369565427342,79.88150725241476,79.88150725251477,79.88927350970445,79.88927350980445,79.89668999393857,79.89668999403857,79.90398340987534,79.90398340997534,79.91077807534397,79.91077807544397,79.91734603605435,79.91734603615436,79.92386217853428,79.92386217863428,79.93037184373539,79.93037184383539,79.93660946322662,79.93660946332662,79.94283412816021,79.94283412826022,79.94891629296006,79.94891629306007,79.95490129857781,79.95490129867781,79.96036164461218,79.96036164471218,79.96542687663931,79.96542687673931,79.97047267683004,79.97047267693004,79.97541484055984,79.97541484065984,79.98033109517442,79.98033109527442,79.98474212204204,79.98474212214204,79.9891013306792,79.9891013307792,79.99300065252106,79.99300065262106,79.99652429219212,79.99652429229212,80.00000259091152,80.00000259101152,80.00328657126671,80.00328657136671,80.00632441502724,80.00632441512724,80.00910316763547,80.00910316773547,80.0115969199762,80.0115969200762,80.01408419503812,80.01408419513812,80.01649374275435,80.01649374285435,80.01866363115472,80.01866363125473,80.02078170132464,80.02078170142464,80.02282852142768,80.02282852152769,80.02483647785787,80.02483647795788,80.02657238857817,80.02657238867818,80.02828886746205,80.02828886756205],[0.0,1e-10,3.048899661668964,3.048899661768964,5.329488985942183,5.329488986042183,7.380093518846096,7.380093518946096,9.266609278484667,9.266609278584667,11.147482798794696,11.147482798894696,12.929115174878921,12.929115174978921,14.48333607820652,14.48333607830652,15.896882230707607,15.896882230807607,17.145577145169227,17.145577145269225,18.367496838312736,18.367496838412734,19.58123909675373,19.58123909685373,20.68599972347944,20.68599972357944,21.74956819078292,21.74956819088292,22.801000760431535,22.801000760531533,23.841231451773595,23.841231451873593,24.849311545139948,24.849311545239946,25.73347205109124,25.73347205119124,26.5918167705652,26.591816770665197,27.447518278822184,27.447518278922182,28.297132776632385,28.297132776732383,29.123415852354288,29.123415852454286,29.92658989154701,29.92658989164701,30.705326934728948,30.705326934828946,31.481395351201442,31.48139535130144,32.25654245607186,32.256542456171864,33.02665729343305,33.02665729353305,33.793525301631064,33.793525301731066,34.55457951592636,34.554579516026365,35.29598755454165,35.29598755464165,35.987962460301,35.987962460401,36.63763963271563,36.637639632815635,37.284730778771355,37.28473077887136,37.92966160796705,37.92966160806705,38.57417943541009,38.57417943551009,39.20079840228048,39.200798402380485,39.8170669098421,39.8170669099421,40.42411594750989,40.42411594760989,41.0307773989175,41.0307773990175,41.63309280111259,41.63309280121259,42.22166477575403,42.22166477585403,42.798571039351316,42.79857103945132,43.372141519561794,43.372141519661795,43.93864013899225,43.93864013909225,44.4976157726513,44.4976157727513,45.0562101739233,45.0562101740233,45.61374983225775,45.613749832357755,46.16472593966159,46.16472593976159,46.70301971632244,46.70301971642244,47.22242342820429,47.22242342830429,47.73652165603283,47.73652165613283,48.24876455291099,48.24876455301099,48.75515553264773,48.75515553274773,49.254163311821664,49.254163311921666,49.742096290151395,49.742096290251396,50.22522574040413,50.22522574050413,50.704816083330286,50.70481608343029,51.18244307946306,51.182443079563065,51.653195184882456,51.65319518498246,52.12051619881828,52.12051619891828,52.577238952393735,52.57723895249374,53.01662834010402,53.01662834020402,53.44222346927572,53.44222346937572,53.85776677117517,53.85776677127517,54.26897673160833,54.268976731708335,54.672936922814166,54.67293692291417,55.07640786578993,55.076407865889934,55.469166178689214,55.469166178789216,55.85041762737228,55.85041762747228,56.215460345732026,56.21546034583203,56.58007100071977,56.58007100081977,56.94457999373762,56.94457999383762,57.3070176241191,57.3070176242191,57.669112145352216,57.66911214545222,58.03034889371445,58.03034889381445,58.389450740709115,58.38945074080912,58.742630777958055,58.74263077805806,59.09326926595991,59.09326926605991,59.43607978228073,59.43607978238073,59.775827731758795,59.7758277318588,60.10947596304384,60.109475963143844,60.43458458885866,60.434584588958664,60.75323767958587,60.753237679685874,61.068103861934915,61.06810386203492,61.3823410108453,61.382341010945304,61.69521207703537,61.69521207713537,62.00508411511388,62.00508411521388,62.303868644601955,62.30386864470196,62.59576557563041,62.59576557573041,62.88382476729575,62.88382476739575,63.17147095720844,63.17147095730844,63.455152330295675,63.45515233039568,63.73348374221778,63.73348374231778,64.00312305571482,64.00312305581483,64.2652457373136,64.26524573741361,64.51882881344216,64.51882881354216,64.77070269770203,64.77070269780204,65.02208097985873,65.02208097995873,65.27246170393595,65.27246170403595,65.51768308304156,65.51768308314156,65.7621610589924,65.7621610590924,66.00574949270677,66.00574949280677,66.24406421173342,66.24406421183342,66.47639993615628,66.47639993625629,66.70374151630861,66.70374151640861,66.92213684311118,66.92213684321118,67.137253571385,67.137253571485,67.3508580778568,67.3508580779568,67.56052318299561,67.56052318309561,67.76660470369602,67.76660470379602,67.96970625790422,67.96970625800422,68.17207076283076,68.17207076293076,68.37407309698959,68.37407309708959,68.5681457974975,68.5681457975975,68.75646824283385,68.75646824293385,68.94348179030796,68.94348179040796,69.12999973567888,69.12999973577888,69.31451621101772,69.31451621111772,69.49790805081471,69.49790805091472,69.67880281847646,69.67880281857646,69.85777236258352,69.85777236268352,70.03663389084758,70.03663389094758,70.21411662864509,70.21411662874509,70.38915312529228,70.38915312539228,70.55501462915747,70.55501462925747,70.71940838833245,70.71940838843246,70.88199764754202,70.88199764764202,71.04057125894117,71.04057125904117,71.19826168197696,71.19826168207696,71.35556451875257,71.35556451885257,71.51204770589598,71.51204770599598,71.65635687214584,71.65635687224584,71.79862009125178,71.79862009135178,71.93686766254733,71.93686766264733,72.07375550499567,72.07375550509568,72.20975380520753,72.20975380530753,72.34314701744113,72.34314701754113,72.4699004322667,72.4699004323667,72.59607564463855,72.59607564473855,72.71367312830147,72.71367312840147,72.83024763839244,72.83024763849244,72.94664424003611,72.94664424013611,73.06051200017893,73.06051200027893,73.17123459312846,73.17123459322846,73.281417106863,73.281417106963,73.39002386006433,73.39002386016433,73.49040870145133,73.49040870155133,73.58707017319134,73.58707017329134,73.68234650059168,73.68234650069168,73.77443953756003,73.77443953766003,73.86546512384461,73.86546512394462,73.9560967699959,73.9560967700959,74.04595959749993,74.04595959759993,74.13511079121479,74.13511079131479,74.21300292176501,74.21300292186501,74.2899483252207,74.28994832532071,74.36688102093017,74.36688102103017,74.44290511278379,74.44290511288379,74.51743604445474,74.51743604455474,74.59088681769569,74.59088681779569,74.66402625115386,74.66402625125386,74.73714662299268,74.73714662309268,74.8094092219606,74.80940922206061,74.881265173049,74.881265173149,74.95249844457186,74.95249844467186,75.02333777596142,75.02333777606142,75.09215657569953,75.09215657579954,75.16073392825918,75.16073392835918,75.2279007209867,75.2279007210867,75.2902322162716,75.2902322163716,75.34924063591595,75.34924063601595,75.40716889713029,75.40716889723029,75.45839382220541,75.45839382230541,75.5092438687666,75.5092438688666,75.55847367768276,75.55847367778276,75.60706174541403,75.60706174551403,75.6552177497733,75.6552177498733,75.70292898301432,75.70292898311432,75.7492995490275,75.7492995491275,75.79467891083432,75.79467891093432,75.83963891701536,75.83963891711537,75.88458621545018,75.88458621555019,75.92887271108074,75.92887271118074,75.97275255883179,75.97275255893179,76.01612409673339,76.0161240968334,76.05918429485224,76.05918429495225,76.10138036622708,76.10138036632708,76.14301729676755,76.14301729686755,76.18449538048009,76.18449538058009,76.26495447576988,76.26495447586989,76.30281720567844,76.30281720577844,76.34067358171386,76.34067358181386,76.37841558803316,76.37841558813317,76.415808131331,76.415808131431,76.4529719351966,76.4529719352966,76.49011032356972,76.49011032366973,76.52686747955578,76.52686747965578,76.5634340193483,76.56343401944831,76.59995608202901,76.59995608212901,76.63610326619578,76.63610326629578,76.67219961937758,76.67219961947758,76.7080100482691,76.7080100483691,76.74330581343808,76.74330581353809,76.77773109798994,76.77773109808994,76.81173702691602,76.81173702701602,76.84569212485717,76.84569212495717,76.87942483723918,76.87942483733919,76.91275725561479,76.9127572557148,76.94579104195387,76.94579104205387,76.978773997308,76.978773997408,77.01149008999117,77.01149009009117,77.04399015098836,77.04399015108837,77.07617887220277,77.07617887230278,77.10790376067959,77.10790376077959,77.13838964389845,77.13838964399845,77.16860231057325,77.16860231067325,77.19878956175558,77.19878956185558,77.22884338160243,77.22884338170243,77.25859856941275,77.25859856951276,77.28776284702313,77.28776284712313,77.3168890013948,77.3168890014948,77.34456011882251,77.34456011892252,77.37194531195992,77.37194531205992,77.39920342763497,77.39920342773497,77.4262328038778,77.4262328039778,77.45313510265825,77.45313510275825,77.47975147714843,77.47975147724843,77.50608828122141,77.50608828132141,77.53226623846645,77.53226623856645,77.55823451789861,77.55823451799861,77.5834276248104,77.5834276249104,77.60856990073725,77.60856990083725,77.63327375941898,77.63327375951899,77.6579013716233,77.6579013717233,77.68207150496313,77.68207150506313,77.7060700837288,77.7060700838288,77.72993523115899,77.72993523125899,77.7536669472537,77.75366694735371,77.77690306124524,77.77690306134524,77.80013282136365,77.80013282146365,77.82318467303477,77.82318467313478,77.84621110921343,77.84621110931343,77.86916129891466,77.86916129901466,77.89209878086966,77.89209878096966,77.9145851378333,77.9145851379333,77.93588967439705,77.93588967449705,77.9561585296426,77.9561585297426,77.9764083232688,77.9764083233688,77.99634042323912,77.99634042333912,78.01585952145678,78.01585952155678,78.03530872707015,78.03530872717015,78.05458002423622,78.05458002433622,78.0736416435894,78.0736416436894,78.09265878583076,78.09265878593077,78.11122480308076,78.11122480318076,78.12939688019746,78.12939688029746,78.14629818269063,78.14629818279063,78.16276106793866,78.16276106803866,78.17856315038246,78.17856315048246,78.19363453741771,78.19363453751771,78.20865509346801,78.20865509356801,78.22328170938502,78.22328170948502,78.23787655593645,78.23787655603645,78.25233161727928,78.25233161737928,78.26673584763718,78.26673584773718,78.28094310792841,78.28094310802841,78.29490256716807,78.29490256726807,78.30883025704212,78.30883025714212,78.32246566875277,78.32246566885277,78.3358088023,78.3358088024,78.34900579676552,78.34900579686553,78.36213925249987,78.36213925259987,78.37509479978692,78.37509479988692,78.3879232696116,78.3879232697116,78.40073267781695,78.40073267791695,78.41348490116424,78.41348490126424,78.42617993965345,78.42617993975345,78.43822688308467,78.43822688318467,78.44993071736752,78.44993071746752,78.46118342665902,78.46118342675902,78.47158471695275,78.47158471705275,78.48179539105293,78.48179539115293,78.49194888029507,78.49194888039507,78.50127636603189,78.50127636613189,78.51048312817947,78.51048312827947,78.51967082870772,78.51967082880772,78.52878228275854,78.52878228285854,78.53751885829541,78.53751885839542,78.545753477856,78.545753477956,78.55396268192409,78.55396268202409,78.56212105500725,78.56212105510726,78.57009516577001,78.57009516587001,78.57775793674999,78.57775793684999,78.58538893836437,78.58538893846438,78.59300723223254,78.59300723233254,78.60028241695234,78.60028241705234,78.60744958582913,78.60744958592913,78.6146040469597,78.6146040470597,78.6214471683075,78.6214471684075,78.62822675092411,78.62822675102412,78.63477124023537,78.63477124033537,78.64129666792728,78.64129666802728,78.6476886642837,78.64768866438371,78.65406159902079,78.6540615991208,78.66042817988476,78.66042817998476,78.66654695969713,78.66654695979713,78.67251324655467,78.67251324665467,78.67838422531545,78.67838422541546,78.68414083436012,78.68414083446012,78.68949714939836,78.68949714949837,78.69445952430331,78.69445952440331,78.69930752949213,78.69930752959213,78.70411741144225,78.70411741154226,78.70889552402679,78.70889552412679,78.71317168063503,78.71317168073503,78.72160962413537,78.72160962423537,78.72562527194577,78.72562527204578,78.72945030356264,78.72945030366265,78.73318638095587,78.73318638105587,78.73664288793191,78.73664288803191,78.74005491779613,78.74005491789613,78.74335257794424,78.74335257804424,78.7465676377418,78.7465676378418,78.74956031198025,78.74956031208025,78.7554567062335,78.7554567063335,78.75831594913649,78.75831594923649,78.7611180071814,78.7611180072814,78.76384381874891,78.76384381884891,78.76630276764547,78.76630276774547,78.7687490087958,78.7687490088958,78.771188896073,78.771188896173,78.77353347525344,78.77353347535345,78.7758145157027,78.77581451580271,78.7779303554509,78.77793035555091,78.78000807196041,78.78000807206041,78.78200318811938,78.78200318821938,78.78399195040522,78.78399195050523,78.78569478840078,78.78569478850078,78.78613955951901,78.78613955961902],[0.0,1e-10,3.199978665864146,3.199978665964146,5.5296121928193624,5.5296121929193625,7.4557007490652145,7.4557007491652145,9.36579514488661,9.36579514498661,11.165824208781832,11.165824208881832,12.726907086231135,12.726907086331135,14.23172375695163,14.23172375705163,15.666991917763605,15.666991917863605,16.898735514430946,16.898735514530944,18.11887803058099,18.11887803068099,19.25632500443173,19.25632500453173,20.338291630422685,20.338291630522683,21.41641270112834,21.41641270122834,22.483892469771487,22.483892469871485,23.538598811059945,23.538598811159943,24.481307380580372,24.48130738068037,25.37678067889961,25.37678067899961,26.264569308114392,26.26456930821439,27.150805543989545,27.150805544089543,28.026361829005356,28.026361829105355,28.899760222864423,28.89976022296442,29.76726467520582,29.767264675305817,30.609222272837833,30.60922227293783,31.44894468171943,31.448944681819427,32.279597506276566,32.27959750637657,33.06873508156442,33.06873508166442,33.8558886852813,33.8558886853813,34.63481009527186,34.63481009537186,35.41150919944428,35.411509199544284,36.17394045611764,36.17394045621764,36.89894035305418,36.89894035315418,37.597556004683206,37.59755600478321,38.25611217839048,38.25611217849048,38.90929616933738,38.90929616943738,39.56208078938364,39.562080789483645,40.208514123816315,40.208514123916316,40.84428039032193,40.84428039042193,41.47302545873545,41.47302545883545,42.097486952166186,42.09748695226619,42.71714955332297,42.71714955342297,43.33517602208032,43.33517602218032,43.950542165322034,43.950542165422036,44.565244837551376,44.56524483765138,45.17392473894026,45.17392473904026,45.76574732344199,45.765747323541994,46.355824270619905,46.35582427071991,46.93768190700378,46.93768190710378,47.51494677803017,47.514946778130174,48.08075228079433,48.08075228089433,48.64654490062621,48.64654490072621,49.20885268727661,49.20885268737661,49.75805209033306,49.75805209043306,50.29769879910459,50.29769879920459,50.82560271510374,50.82560271520374,51.33847869059937,51.338478690699375,51.847953571973335,51.84795357207334,52.349286440146926,52.34928644024693,52.84581397458043,52.84581397468043,53.341684479467695,53.3416844795677,53.833548392416176,53.83354839251618,54.31537005965313,54.31537005975313,54.789178543012504,54.789178543112506,55.22391953423562,55.22391953433562,55.654995331225344,55.654995331325345,56.07153273913814,56.071532739238144,56.487438883369265,56.48743888346927,56.90010497013221,56.90010497023221,57.31245542505432,57.31245542515432,57.72142411025317,57.72142411035317,58.12949743165862,58.12949743175862,58.535432186305755,58.53543218640576,58.940819416331024,58.940819416431026,59.34524042643537,59.34524042653537,59.72015308015451,59.72015308025451,60.08982238043608,60.089822380536084,60.45706324798305,60.45706324808305,60.824188169139504,60.824188169239505,61.190920160861445,61.19092016096145,61.55491452947409,61.55491452957409,61.91285391991557,61.91285392001557,62.25603591143139,62.25603591153139,62.59577171856255,62.595771718662554,62.93049606503716,62.93049606513716,63.25352270900239,63.25352270910239,63.57275532941143,63.57275532951143,63.889643256145675,63.88964325624568,64.20513338472765,64.20513338482765,64.51248794157539,64.51248794167539,64.8194624519209,64.8194624520209,65.12221136047887,65.12221136057887,65.42435477121974,65.42435477131974,65.72006959875334,65.72006959885334,66.00627038079887,66.00627038089887,66.29192363822253,66.29192363832253,66.57670729771735,66.57670729781735,66.85718805783097,66.85718805793097,67.13413245303398,67.13413245313399,67.40455808450379,67.40455808460379,67.67298686147035,67.67298686157035,67.9386780153276,67.9386780154276,68.1943720137363,68.1943720138363,68.44969884857504,68.44969884867504,68.7043750953337,68.7043750954337,68.95700295585998,68.95700295595998,69.20703490553204,69.20703490563204,69.45422616863655,69.45422616873655,69.69616763683736,69.69616763693736,69.93179002675528,69.93179002685528,70.1646103789025,70.1646103790025,70.38599068718591,70.38599068728591,70.60675905618608,70.60675905628608,70.8193081143922,70.8193081144922,71.02792143678705,71.02792143688706,71.23385510926788,71.23385510936788,71.43904801314265,71.43904801324265,71.64203793559771,71.64203793569772,71.84180712498299,71.841807125083,72.04006901129164,72.04006901139164,72.23616012351125,72.23616012361126,72.42825752672435,72.42825752682435,72.6192212318969,72.6192212319969,72.80865830959438,72.80865830969438,72.99578934241391,72.99578934251392,73.18188974065112,73.18188974075112,73.36321701247893,73.36321701257893,73.54337837893547,73.54337837903547,73.71148776224497,73.71148776234497,73.87631843928943,73.87631843938944,74.04055650144907,74.04055650154908,74.2005560788889,74.2005560789889,74.36051700753188,74.36051700763188,74.51870653298649,74.51870653308649,74.67661263393096,74.67661263403096,74.8305379088012,74.8305379089012,74.97698464148345,74.97698464158346,75.12121550981371,75.12121550991371,75.26131095688237,75.26131095698237,75.39899729561486,75.39899729571486,75.5316914980892,75.5316914981892,75.66351610263472,75.66351610273472,75.79195893745697,75.79195893755697,75.91984780619123,75.91984780629123,76.03906646150168,76.03906646160168,76.15791151177604,76.15791151187604,76.27633786675132,76.27633786685132,76.39433908496139,76.39433908506139,76.50975727524951,76.50975727534951,76.62146518104127,76.62146518114128,76.73175596428233,76.73175596438233,76.83364063421129,76.8336406343113,76.93290362742148,76.93290362752148,77.03089765180216,77.03089765190217,77.12317165425095,77.12317165435095,77.21425398546393,77.21425398556393,77.3046341968677,77.3046341969677,77.39398377368913,77.39398377378913,77.47296903149207,77.47296903159207,77.55101383523863,77.55101383533864,77.62900710725609,77.62900710735609,77.70610501548013,77.70610501558014,77.78166341329684,77.78166341339684,77.8557982470967,77.8557982471967,77.92804573131768,77.92804573141768,77.99933343708388,77.99933343718388,78.06918469590096,78.06918469600096,78.13873320580949,78.13873320590949,78.20714801767744,78.20714801777744,78.27468034868429,78.27468034878429,78.33796775350518,78.33796775360518,78.39778964954301,78.39778964964302,78.45643919874345,78.45643919884346,78.51049598258639,78.5104959826864,78.56204059463492,78.56204059473492,78.61129848620392,78.61129848630392,78.66011835807544,78.66011835817544,78.7081652540102,78.7081652541102,78.75524593002403,78.75524593012403,78.80079353709667,78.80079353719667,78.84627672950789,78.8462767296079,78.89072284587066,78.89072284597066,78.93471161813751,78.93471161823751,78.97706425800494,78.97706425810495,79.01927518561729,79.01927518571729,79.06128642777931,79.06128642787931,79.10080482254534,79.10080482264534,79.13995605374141,79.13995605384142,79.17887539215646,79.17887539225646,79.21760792805347,79.21760792815347,79.2559733003805,79.2559733004805,79.29388132861165,79.29388132871165,79.3315123737988,79.3315123738988,79.36859589436408,79.36859589446408,79.40566009053094,79.40566009063095,79.44230559139874,79.44230559149874,79.47889956053741,79.47889956063742,79.51520366369981,79.51520366379981,79.55048357374602,79.55048357384602,79.58569906913084,79.58569906923084,79.61918825159027,79.61918825169028,79.65262590232058,79.65262590242058,79.68579945293916,79.68579945303917,79.71667340014594,79.71667340024594,79.74727680577485,79.74727680587485,79.77740354290944,79.77740354300944,79.80725329700003,79.80725329710003,79.83681962658048,79.83681962668048,79.86627645123656,79.86627645133656,79.89552070751003,79.89552070761003,79.92315459724861,79.92315459734861,79.95074339672423,79.95074339682424,79.97801656435901,79.97801656445901,80.00446522432793,80.00446522442793,80.02996698877433,80.02996698887434,80.05545587028845,80.05545587038846,80.080410110113,80.080410110213,80.104739527722,80.104739527822,80.12827664499585,80.12827664509585,80.15170425734533,80.15170425744533,80.1750803379657,80.1750803380657,80.19810857941452,80.19810857951452,80.2208856036839,80.2208856037839,80.24264487630323,80.24264487640323,80.26424311226909,80.26424311236909,80.28467544286369,80.28467544296369,80.30488232214338,80.30488232224339,80.32468983052244,80.32468983062245,80.34426544612049,80.34426544622049,80.36382173732011,80.36382173742011,80.38320410893395,80.38320410903395,80.40252850735254,80.40252850745254,80.42136979581068,80.42136979591068,80.4400629305476,80.4400629306476,80.45806682840758,80.45806682850758,80.47554896751028,80.47554896761028,80.49268326744144,80.49268326754144,80.5092185110216,80.5092185111216,80.52525776170904,80.52525776180904,80.54049182912902,80.54049182922903,80.55528787685154,80.55528787695154,80.56989068058985,80.56989068068985,80.58422294275032,80.58422294285032,80.61261692549337,80.61261692559337,80.62611179705569,80.62611179715569,80.63934900997242,80.63934901007242,80.65235433010814,80.65235433020814,80.66528235265018,80.66528235275018,80.67756622857826,80.67756622867826,80.68977924837881,80.68977924847881,80.70180546566132,80.70180546576132,80.7130458240748,80.7130458241748,80.72339726016102,80.72339726026102,80.73369716451813,80.73369716461814,80.7438424736879,80.74384247378791,80.7535562046263,80.75355620472631,80.76288988906249,80.7628898891625,80.7720496539129,80.7720496540129,80.78090666985476,80.78090666995476,80.78962841500768,80.78962841510769,80.79797655512452,80.79797655522452,80.8062989293768,80.8062989294768,80.81456977189995,80.81456977199996,80.82229308980125,80.82229308990125,80.83748850842441,80.83748850852442,80.84486398715417,80.84486398725417,80.85212996095956,80.85212996105956,80.85938305183268,80.85938305193268,80.8666232597735,80.8666232598735,80.87378617012067,80.87378617022067,80.88031781678615,80.88031781688615,80.88679793172253,80.88679793182253,80.89327160519275,80.89327160529275,80.89947473708511,80.89947473718512,80.90552327379014,80.90552327389014,80.91147518850306,80.91147518860306,80.91690534445868,80.91690534455869,80.92220667109153,80.92220667119153,80.9272374561465,80.9272374562465,80.93215229481096,80.93215229491096,80.93705425054314,80.93705425064314,80.94193044041077,80.94193044051077,80.95605657565476,80.95605657575476,80.96039168236666,80.96039168246666,80.96472034761243,80.96472034771243,80.96860455169457,80.96860455179457,80.97248231431057,80.97248231441057,80.97617971587466,80.97617971597467,80.97968387345456,80.97968387355456,80.98309140904237,80.98309140914238,80.98635079090897,80.98635079100897,80.98944913612209,80.98944913622209,80.99228982268963,80.99228982278963,80.9950725360619,80.9950725361619,80.99767488838228,80.99767488848228,81.000154852846,81.000154852946,81.00262837584357,81.00262837594357,81.00503748417977,81.00503748427977,81.00714384360738,81.00714384370738,81.00901831025399,81.009018310354,81.01074462317938,81.01074462327938,81.01200070907659,81.01200070917659,81.01301846072664,81.01301846082664,81.01372058053585,81.01372058063585,81.01426810515771,81.01426810525771],[0.0,1e-10,3.166908869208585,3.166908869308585,5.0758367987189645,5.0758367988189645,6.974405591049092,6.974405591149092,8.75821965302571,8.75821965312571,10.29788870188969,10.29788870198969,11.735102501205056,11.735102501305056,13.167969675492847,13.167969675592847,14.430578079427455,14.430578079527455,15.680906150685043,15.680906150785043,16.881340583410267,16.881340583510266,18.054182563867943,18.05418256396794,19.225045904445224,19.225045904545222,20.39162644708785,20.39162644718785,21.5345845826423,21.534584582742298,22.67265994558868,22.67265994568868,23.771909287914315,23.771909288014314,24.850312701693614,24.850312701793612,25.919020780058986,25.919020780158984,26.8308099504906,26.8308099505906,27.73315271117066,27.73315271127066,28.627261776864568,28.627261776964566,29.508318202057172,29.50831820215717,30.374266754356583,30.37426675445658,31.23355814086486,31.23355814096486,32.020910010947624,32.020910011047626,32.80646195701016,32.80646195711016,33.59000973235513,33.59000973245513,34.369402363951274,34.369402364051275,35.14336969264957,35.14336969274957,35.91339250700566,35.91339250710566,36.63169622797199,36.631696228071995,37.33330278143153,37.33330278153153,37.9708907606964,37.9708907607964,38.60720858081225,38.60720858091225,39.24046908317742,39.240469083277425,39.87012335479285,39.870123354892854,40.49195880752608,40.491958807626084,41.11369213691064,41.113692137010645,41.73266175317195,41.732661753271955,42.34699752248756,42.346997522587564,42.95647605003227,42.956476050132274,43.56583968880973,43.56583968890973,44.16362509293225,44.16362509303225,44.73973481630052,44.73973481640052,45.31181066739651,45.31181066749651,45.880056892917544,45.880056893017546,46.443445876667674,46.443445876767676,47.00347755533043,47.00347755543043,47.56267948178528,47.56267948188528,48.11368600950973,48.11368600960973,48.65819493917533,48.65819493927533,49.197380689291755,49.19738068939176,49.72769447349286,49.72769447359286,50.24422160562413,50.244221605724135,50.7524065367113,50.7524065368113,51.24901961585281,51.24901961595281,51.73917339319123,51.73917339329123,52.225778384163526,52.22577838426353,52.7027646322334,52.7027646323334,53.17227034501352,53.172270345113525,53.64107395977157,53.64107395987157,54.0782384845712,54.0782384846712,54.5031354421124,54.503135442212404,54.91981785279532,54.91981785289532,55.33256213184533,55.33256213194533,55.74394689381623,55.74394689391623,56.149197872157906,56.14919787225791,56.55381057957043,56.55381057967043,56.94140060130228,56.94140060140228,57.324465282146384,57.324465282246386,57.690596635239906,57.69059663533991,58.056153544497185,58.05615354459719,58.42085517070939,58.42085517080939,58.78451003259777,58.784510032697774,59.14728408060392,59.14728408070392,59.50202868032124,59.50202868042124,59.83986548312514,59.83986548322514,60.17505346157303,60.17505346167303,60.50096098071094,60.500960980810945,60.82134107360234,60.82134107370234,61.13766176338429,61.13766176348429,61.445091339123046,61.44509133922305,61.74308727052883,61.743087270628834,62.03595150366419,62.03595150376419,62.324679741178585,62.32467974127859,62.60865286027075,62.60865286037075,62.881086040963716,62.88108604106372,63.15136186591613,63.15136186601613,63.420788790532754,63.420788790632756,63.68587547283111,63.68587547293111,63.94920691007427,63.94920691017427,64.20263876520616,64.20263876530616,64.45417495567847,64.45417495577847,64.70523882566319,64.70523882576319,64.95369216754766,64.95369216764766,65.19991794388935,65.19991794398935,65.44459272187319,65.4445927219732,65.6844230235047,65.68442302360471,65.91803018357693,65.91803018367693,66.150386332628,66.150386332728,66.37870860940679,66.37870860950679,66.59502501000809,66.59502501010809,66.80548208347972,66.80548208357972,67.01355840638557,67.01355840648557,67.22061349580477,67.22061349590477,67.42466871185692,67.42466871195693,67.62791970653835,67.62791970663835,67.83023882566319,67.83023882576319,68.0252178291027,68.0252178292027,68.21554383746864,68.21554383756865,68.40421034141877,68.40421034151878,68.58830044280684,68.58830044290684,68.7710246444065,68.7710246445065,68.94710444563361,68.94710444573361,69.11359103479546,69.11359103489546,69.27691818285798,69.27691818295798,69.43829222187728,69.43829222197728,69.59687063422685,69.59687063432685,69.7553150096813,69.7553150097813,69.90910000735288,69.90910000745288,70.05618954297759,70.0561895430776,70.20105151305954,70.20105151315954,70.34519861970082,70.34519861980083,70.48909680067973,70.48909680077973,70.63198013088129,70.63198013098129,70.77125084762379,70.77125084772379,70.9100747747159,70.9100747748159,71.04656901291656,71.04656901301657,71.17811026870696,71.17811026880696,71.30539425739985,71.30539425749986,71.43198891348926,71.43198891358927,71.54975628262841,71.54975628272841,71.66745344196535,71.66745344206535,71.78412936781562,71.78412936791563,71.89998830687658,71.89998830697658,72.01441113634692,72.01441113644692,72.12652342505372,72.12652342515372,72.2358337043815,72.23583370448151,72.33763153487308,72.33763153497308,72.43859961315675,72.43859961325676,72.5393634447431,72.5393634448431,72.6378741799495,72.63787418004951,72.73497433640249,72.73497433650249,72.8264194124231,72.82641941252311,72.91670921806194,72.91670921816194,72.99693987385724,72.99693987395725,73.07518550706286,73.07518550716286,73.15255670909552,73.15255670919552,73.22983855319809,73.2298385532981,73.30622681799986,73.30622681809986,73.38109599799022,73.38109599809022,73.45456736464571,73.45456736474571,73.52677495486148,73.52677495496148,73.59896977965866,73.59896977975866,73.67079440731693,73.67079440741693,73.74237649202212,73.74237649212212,73.81157144345227,73.81157144355227,73.87936219883825,73.87936219893825,73.94696147294549,73.94696147304549,74.01387779715851,74.01387779725852,74.07649217530903,74.07649217540903,74.13576839650003,74.13576839660003,74.194387198634,74.194387198734,74.24815514170636,74.24815514180636,74.30160394931414,74.30160394941414,74.35265924093757,74.35265924103757,74.40317200227122,74.40317200237122,74.45198058022402,74.45198058032402,74.50048917084011,74.50048917094011,74.54886372456107,74.54886372466108,74.5971170068055,74.5971170069055,74.64517880777117,74.64517880787118,74.69173428934404,74.69173428944404,74.73810467234745,74.73810467244745,74.78326872329474,74.78326872339474,74.82828597192834,74.82828597202834,74.87244155480757,74.87244155490757,74.91649501433812,74.91649501443813,74.9600825360904,74.9600825361904,75.00338283592454,75.00338283602454,75.04630655591049,75.04630655601049,75.08827925221199,75.08827925231199,75.13010514619978,75.13010514629978,75.17175232432741,75.17175232442742,75.21337397161788,75.21337397171789,75.25424884192122,75.25424884202123,75.29452373755116,75.29452373765116,75.33368165905507,75.33368165915508,75.37262895115236,75.37262895125237,75.41142305822665,75.41142305832665,75.45019801717307,75.45019801727307,75.48859639627128,75.48859639637128,75.52696924453231,75.52696924463231,75.56453148871333,75.56453148881333,75.6018192763948,75.60181927649481,75.63898579259974,75.63898579269974,75.67587146959583,75.67587146969584,75.71266778866186,75.71266778876186,75.74934921896063,75.74934921906063,75.7856604521205,75.7856604522205,75.82163978439719,75.8216397844972,75.85761273396459,75.85761273406459,75.8935090910205,75.8935090911205,75.92911822615828,75.92911822625828,75.96198917900998,75.96198917910998,75.99470056412937,75.99470056422938,76.02736088757445,76.02736088767445,76.05997653205448,76.05997653215448,76.09231133732568,76.09231133742568,76.12444827860884,76.12444827870884,76.15640012132253,76.15640012142254,76.18702436050359,76.18702436060359,76.21734861234793,76.21734861244794,76.24753882729716,76.24753882739716,76.27739075865394,76.27739075875394,76.30669377701163,76.30669377711163,76.33433090824421,76.33433090834421,76.36171273110514,76.36171273120515,76.3880733204794,76.38807332057941,76.41419774960988,76.41419774970988,76.43948604382317,76.43948604392317,76.46474242449,76.46474242459,76.48973073136658,76.48973073146658,76.51445734716219,76.5144573472622,76.53856484015654,76.53856484025654,76.56264041960442,76.56264041970442,76.58669046821512,76.58669046831513,76.61047882574489,76.6104788258449,76.6340054921937,76.6340054922937,76.65743641800312,76.65743641810312,76.68077160317317,76.68077160327317,76.70408764021536,76.70408764031536,76.72733346745534,76.72733346755534,76.7505090848931,76.7505090849931,76.77362725794724,76.77362725804724,76.79659862868768,76.79659862878768,76.81956361671882,76.81956361681883,76.84213287677389,76.84213287687389,76.8645106555502,76.86451065565021,76.88622463256019,76.88622463266019,76.90762585681489,76.90762585691489,76.92891857501164,76.92891857511164,76.94927941765182,76.94927941775182,76.96912964354867,76.96912964364867,76.9887564746203,76.9887564747203,77.00813438002957,77.00813438012958,77.02728250790435,77.02728250800435,77.04634766055833,77.04634766065833,77.06499793710836,77.06499793720836,77.08357162114689,77.08357162124689,77.10205594725534,77.10205594735534,77.1203679402129,77.1203679403129,77.13809910662495,77.13809910672495,77.15568985343258,77.15568985353258,77.17324868669374,77.17324868679374,77.19055859429253,77.19055859439253,77.20764510706611,77.20764510716612,77.22462311378175,77.22462311388175,77.24149899714871,77.24149899724871,77.2579089427374,77.2579089428374,77.29007779756701,77.29007779766701,77.30516652233233,77.30516652243233,77.31985951912158,77.31985951922158,77.33452060236436,77.33452060246437,77.34912424122352,77.34912424132352,77.36364490486189,77.36364490496189,77.37810812411664,77.37810812421664,77.39230965229042,77.39230965239042,77.40642820524342,77.40642820534342,77.42045101755704,77.42045101765704,77.43439723735918,77.43439723745918,77.44817112401043,77.44817112411043,77.46166417145285,77.46166417155285,77.50210501752437,77.50210501762437,77.51518957157212,77.51518957167212,77.52820391581768,77.52820391591769,77.5410906058774,77.5410906059774,77.55390070342563,77.55390070352563,77.56648102343935,77.56648102353935,77.57893368926725,77.57893368936725,77.59113104672348,77.59113104682348,77.60327734250536,77.60327734260537,77.6153981074501,77.6153981075501,77.62735292195325,77.62735292205325,77.63926944020065,77.63926944030065,77.65102639071576,77.65102639081576,77.66271951413796,77.66271951423796,77.67439987214156,77.67439987224157,77.68599725492439,77.68599725502439,77.69742230455634,77.69742230465634,77.70872608271175,77.70872608281175,77.71997879919282,77.71997879929282,77.73080387415135,77.73080387425135,77.74106088798294,77.74106088808294,77.75128598826807,77.75128598836807,77.76133875540232,77.76133875550232,77.77083622682821,77.77083622692821,77.78021242677755,77.78021242687755,77.78948650337824,77.78948650347824,77.79871590101388,77.79871590111388,77.80760063234777,77.80760063244777,77.81637047491442,77.81637047501442,77.82467437970278,77.82467437980279,77.83295913636327,77.83295913646327,77.84123112760517,77.84123112770517,77.8494775880099,77.8494775881099,77.8576729867403,77.8576729868403,77.86574711399416,77.86574711409416,77.87344466139983,77.87344466149983,77.8887950772461,77.8887950773461,77.89622455086152,77.89622455096152,77.90353275300038,77.90353275310038,77.9107324490813,77.9107324491813,77.91791937974362,77.91791937984362,77.92438506425601,77.92438506435602,77.93081245251264,77.93081245261264,77.94338638981708,77.94338638991708,77.94937975384188,77.94937975394188,77.95533482161093,77.95533482171093,77.96096437120612,77.96096437130612,77.96649818016192,77.96649818026192,77.97195539660623,77.97195539670624,77.97738708221338,77.97738708231338,77.98276770614619,77.9827677062462,77.98802067589317,77.98802067599317,77.99302471997778,77.99302472007778,77.99798408509734,77.99798408519734,78.00293068479833,78.00293068489833,78.00780069198781,78.00780069208781,78.01260048937509,78.01260048947509,78.01716412651858,78.01716412661858,78.02145968987182,78.02145968997182,78.0295976442186,78.0295976443186,78.03338259082851,78.03338259092851,78.03685478468313,78.03685478478313,78.04028229957271,78.04028229967271,78.04325664210259,78.04325664220259,78.04614800941168,78.04614800951168,78.04902022859291,78.04902022869291,78.0518350033905,78.0518350034905,78.05459233380446,78.05459233390447,78.05704967688172,78.05704967698172,78.0595006372497,78.0595006373497,78.06163884486237,78.06163884496237,78.06377066976577,78.06377066986578,78.06585781570412,78.06585781580412,78.0677726284916,78.0677726285916,78.06966191044191,78.06966191054191,78.07054910703344,78.07054910713344,78.07114269899756,78.07114269909756,78.07165969845018,78.07165969855018],[0.0,1e-10,3.1936472058966174,3.1936472059966174,5.115990229976095,5.115990230076095,7.030570684663126,7.030570684763126,8.829484993427885,8.829484993527885,10.382256336905295,10.382256337005295,11.83160399464653,11.83160399474653,13.27712186068917,13.27712186078917,14.550395649665589,14.550395649765589,15.811259626213618,15.811259626313618,17.02187287370126,17.021872873801257,18.204596226348542,18.20459622644854,19.385349971836547,19.385349971936545,20.561810488647307,20.561810488747305,21.714410437682112,21.71441043778211,22.862112115317537,22.862112115417535,23.99547299311352,23.995472993213518,25.104001372803317,25.104001372903316,26.181724066662063,26.18172406676206,27.101208778809447,27.101208778909445,28.011360385136726,28.011360385236724,28.91311219622591,28.91311219632591,29.80161743367525,29.80161743377525,30.668180217510283,30.66818021761028,31.53335912703406,31.53335912713406,32.327361840586306,32.32736184068631,33.11954298934745,33.11954298944745,33.909696601326985,33.90969660142699,34.69565997062453,34.695659970724535,35.476171518798196,35.4761715188982,36.25271810615449,36.25271810625449,36.97711516016285,36.977115160262855,37.684648257447996,37.684648257548,38.32760913570741,38.327609135807414,38.969314872149646,38.96931487224965,39.607943901983596,39.6079439020836,40.24291692898594,40.24291692908594,40.86998578085237,40.86998578095237,41.49698382984705,41.496983829947055,42.12118194709571,42.12118194719571,42.740700638184904,42.740700638284906,43.35531462125002,43.35531462135002,43.969832054944575,43.96983205504458,44.572621958429316,44.57262195852932,45.153598140793825,45.15359814089383,45.730506376345716,45.73050637644572,46.30355907370019,46.303559073800194,46.871713499655286,46.87171349975529,47.43647582439163,47.43647582449163,48.00040138791651,48.00040138801651,48.556068761946776,48.55606876204678,49.10517077877945,49.105170778879454,49.6489110872341,49.6489110873341,50.183704487350845,50.183704487450846,50.70458834148328,50.70458834158328,51.21706596375292,51.217065963852924,51.71787398543508,51.71787398553508,52.21212952316905,52.21212952326905,52.70285135394079,52.70285135404079,53.1838345715361,53.183834571636105,53.6573062481024,53.6573062482024,54.130063459326614,54.130063459426616,54.57092719514488,54.570927195244884,54.99940686503357,54.99940686513357,55.41960259892883,55.419602599028835,55.83583337200672,55.83583337210672,56.25069314402276,56.250693144122764,56.65936731969872,56.65936731979872,57.06739139627962,57.06739139637962,57.458236121526824,57.458236121626825,57.84454302635804,57.84454302645804,58.21376069260142,58.21376069270142,58.58240549924618,58.58240549934618,58.950187798180664,58.950187798280666,59.31691449066377,59.31691449076377,59.682752928937795,59.6827529290378,60.04049409333479,60.040494093434795,60.38119751214157,60.38119751224157,60.71922329507168,60.71922329517168,61.04788378905816,61.04788378915816,61.3709766026761,61.3709766027761,61.6899692863579,61.6899692864579,61.9999957518277,61.9999957519277,62.3005410691092,62.3005410692092,62.595892030254966,62.59589203035497,62.887059185343446,62.88705918544345,63.17663649413015,63.17663649423015,63.46300836381451,63.46300836391451,63.7377492526596,63.7377492527596,64.00944561801992,64.00944561811993,64.27678438845597,64.27678438855597,64.54235952372325,64.54235952382325,64.79793214420125,64.79793214430126,65.05159308714231,65.05159308724231,65.30172675974583,65.30172675984583,65.55002599430887,65.55002599440887,65.79676756569364,65.79676756579364,66.03863661217785,66.03863661227786,66.27421707632672,66.27421707642672,66.508181947675,66.508181947775,66.73843932319406,66.73843932329406,66.95658297102109,66.9565829711211,67.1688177973703,67.1688177974703,67.37865176270508,67.37865176280508,67.5874558680873,67.5874558681873,67.79323475985886,67.79323475995886,67.9982155101672,67.9982155102672,68.2022436400194,68.2022436401194,68.39886965146256,68.39886965156256,68.59080336349686,68.59080336359686,68.78106355310824,68.78106355320824,68.9667086828072,68.9667086829072,69.15097637481959,69.1509763749196,69.32854354051562,69.32854354061563,69.50106439244409,69.5010643925441,69.66895087458603,69.66895087468603,69.83365766412443,69.83365766422443,69.99639484650356,69.99639484660356,70.15631278726254,70.15631278736254,70.31609555890275,70.31609555900275,70.47117959450938,70.47117959460938,70.61951161080202,70.61951161090202,70.76559724507314,70.76559724517314,70.91096197737745,70.91096197747746,71.05595982207367,71.05595982217368,71.20005010268669,71.20005010278669,71.34049725371752,71.34049725381752,71.4804938410191,71.4804938411191,71.6181410603039,71.6181410604039,71.75078702219406,71.75078702229406,71.87913975540697,71.87913975550697,72.00680376977661,72.00680376987661,72.12556593218127,72.12556593228128,72.2442508550895,72.2442508551895,72.36204752378863,72.36204752388863,72.479709023369,72.479709023469,72.59509839442903,72.59509839452903,72.70815770734637,72.70815770744638,72.81839134201887,72.81839134211887,72.92103619616591,72.92103619626592,73.02285716235092,73.02285716245092,73.12447215654541,73.12447215664541,73.22381502221955,73.22381502231956,73.32173539383422,73.32173539393422,73.41395291596156,73.41395291606156,73.50500540901757,73.50500540911757,73.58592021816553,73.58592021826553,73.66658399895006,73.66658399905006,73.74549058119052,73.74549058129053,73.8235153458466,73.8235153459466,73.90144999775684,73.90144999785684,73.97848995883328,73.97848995893328,74.05399156660557,74.05399156670558,74.1280706803184,74.1280706804184,74.20088821558934,74.20088821568935,74.27369287761087,74.27369287771087,74.34612421539958,74.34612421549959,74.41831096144958,74.41831096154958,74.48808397323575,74.48808397333575,74.55644736421183,74.55644736431184,74.62461765644683,74.62461765654683,74.69209922983855,74.69209922993855,74.75524251818008,74.75524251828008,74.81501945180169,74.81501945190169,74.87413341307882,74.87413341317882,74.92835553958129,74.92835553968129,74.98224939822387,74.98224939832387,75.03373595922733,75.03373595932733,75.084675407131,75.084675407231,75.13389627623881,75.13389627633882,75.18281462398558,75.18281462408558,75.23159780261356,75.23159780271357,75.28025868537219,75.28025868547219,75.3287264693897,75.3287264694897,75.37567520997717,75.37567521007718,75.42243728844826,75.42243728854827,75.46798284485037,75.46798284495037,75.5133803588843,75.5133803589843,75.55790892858327,75.55790892868328,75.60233451228697,75.60233451238697,75.64629022238732,75.64629022248732,75.689956284376,75.689956284476,75.73324258550718,75.73324258560719,75.77556982955754,75.77556982965754,75.81774903123971,75.81774903133972,75.85974800743018,75.85974800753019,75.90136722276316,75.90136722286316,75.9425873673645,75.9425873674645,75.98320246924372,75.98320246934372,76.02269116179981,76.02269116189981,76.0619674457407,76.06196744584071,76.1010892506887,76.1010892507887,76.14019174576259,76.1401917458626,76.17890804335427,76.17890804345427,76.21760503107184,76.21760503117184,76.25548456745206,76.25548456755206,76.29308732897003,76.29308732907003,76.330516301621,76.330516301721,76.36750758379215,76.36750758389215,76.40419634460221,76.40419634470221,76.44087866878758,76.44087866888758,76.47749019010121,76.47749019020121,76.51403090854309,76.5140309086431,76.55031416199684,76.55031416209684,76.58659097882588,76.58659097892588,76.62279055615849,76.6227905562585,76.65870048537943,76.65870048547943,76.69184266597786,76.69184266607786,76.72483036758341,76.72483036768341,76.75776657619133,76.75776657629133,76.79065772842631,76.79065772852631,76.82327854242376,76.82327854252377,76.85568694780599,76.855686947906,76.88790869107181,76.88790869117182,76.9187916163993,76.9187916164993,76.94937202036574,76.94937202046574,76.97981725521339,76.97981725531339,77.00992134895176,77.00992134905177,77.03947189296562,77.03947189306562,77.06734247793187,77.06734247803188,77.09495559790997,77.09495559800997,77.12220826690468,77.12220826700468,77.14879152693022,77.14879152703023,77.17513663184174,77.17513663194174,77.20063853891709,77.20063853901709,77.22583792463138,77.22583792473138,77.25076697210811,77.25076697220811,77.27507810361331,77.27507810371331,77.29935705199499,77.29935705209499,77.32361025387786,77.32361025397786,77.34733565253504,77.34733565263504,77.37104817794281,77.37104817804281,77.39467702722943,77.39467702732944,77.4182093271455,77.4182093272455,77.44172231718747,77.44172231728747,77.46516450435769,77.46516450445769,77.48851657878207,77.48851657888207,77.51183003345822,77.51183003355823,77.53498900914148,77.53498900924149,77.58090788977641,77.58090788987641,77.60347469598695,77.60347469608695,77.62537209322834,77.62537209332834,77.64695409585923,77.64695409595923,77.66842667587018,77.66842667597018,77.68895307204971,77.68895307214972,77.70897097487766,77.70897097497766,77.72876359584099,77.72876359594099,77.74830518844088,77.74830518854088,77.76761506255144,77.76761506265144,77.78684126054085,77.78684126064086,77.80564264129984,77.80564264139984,77.8243732191871,77.8243732192871,77.84301368432848,77.84301368442848,77.86148036060288,77.86148036070288,77.87936130402927,77.87936130412928,77.89710064171217,77.89710064181217,77.91480779627156,77.91480779637156,77.93226392246751,77.93226392256751,77.94949476679884,77.94949476689884,77.9666161885102,77.9666161886102,77.98316475062295,77.98316475072295,77.99905677701595,77.99905677711595,78.01427295781507,78.01427295791507,78.02909006788258,78.02909006798258,78.04387499482657,78.04387499492657,78.05860199214821,78.05860199224821,78.07324531334874,78.07324531344874,78.08783714155162,78.08783714165162,78.1020749553958,78.10207495549581,78.11621621986943,78.11621621996943,78.13028024484663,78.13028024494663,78.14417048095683,78.14417048105683,78.1578289985777,78.1578289986777,78.17143602320094,78.17143602330094,78.19861145269921,78.19861145279921,78.21180653334143,78.21180653344143,78.2249308111119,78.2249308112119,78.23792635638831,78.23792635648832,78.25084466216828,78.25084466226828,78.26353124945892,78.26353124955892,78.27608910425549,78.27608910435549,78.28838949406392,78.28838949416392,78.30063839087472,78.30063839097473,78.3128615411867,78.31286154128671,78.3249173392564,78.3249173393564,78.33693451757787,78.33693451767788,78.34879078028176,78.34879078038176,78.36058267673862,78.36058267683862,78.37236169994605,78.37236170004606,78.38405704703236,78.38405704713236,78.39557860525166,78.39557860535166,78.4069778676016,78.4069778677016,78.4183256369539,78.4183256370539,78.42924215245107,78.42924215255107,78.43996556920713,78.43996556930713,78.4503092251057,78.4503092252057,78.46062069788073,78.46062069798073,78.47075838178878,78.47075838188879,78.48033607934762,78.48033607944762,78.48979148103709,78.48979148113709,78.49914389673131,78.49914389683131,78.5084512560526,78.5084512561526,78.5174110376399,78.5174110377399,78.52626139660724,78.52626139670724,78.53463544534652,78.53463544544653,78.54299018421169,78.54299018431169,78.55133204982745,78.55133204992745,78.55959667594678,78.55959667604678,78.56773900619673,78.56773900629673,78.57550157558917,78.57550157568917,78.59098165800114,78.59098165810114,78.59847388915604,78.59847388925604,78.60584382444158,78.60584382454158,78.61310433710715,78.61310433720715,78.62035197652331,78.62035197662331,78.62687871397269,78.62687871407269,78.63337326829854,78.63337326839854,78.63984851275028,78.63984851285028,78.64604698233977,78.64604698243977,78.65209097293638,78.65209097303638,78.65809634378476,78.65809634388476,78.66377344677326,78.66377344687326,78.66935400039122,78.66935400049123,78.67483156801391,78.67483156811392,78.68573521026168,78.68573521036168,78.69103255239267,78.69103255249267,78.70137620829124,78.70137620839124,78.70637746568588,78.70637746578588,78.7113658498311,78.7113658499311,78.71627699447988,78.71627699457989,78.72111733625694,78.72111733635694,78.72571952291995,78.72571952301995,78.73005137134543,78.73005137144543,78.73825806784241,78.73825806794241,78.7420749862916,78.7420749863916,78.7455700735056,78.7455700736056,78.7490265409714,78.7490265410714,78.75202600808325,78.75202600818325,78.75494179907393,78.75494179917393,78.75783828019053,78.75783828029053,78.76067683168478,78.76067683178478,78.7634574535567,78.7634574536567,78.76593555406755,78.76593555416756,78.76840721795371,78.76840721805371,78.77056348722938,78.77056348732938,78.77266826350743,78.77266826360743,78.77457350441968,78.77457350451968,78.77632426633903,78.77632426643903,78.77896971909217,78.77896971919218,78.77956832518962,78.77956832528962,78.7800896917906,78.7800896918906],[0.0,1e-10,3.0134048968634253,3.0134048969634253,5.606919611259227,5.606919611359227,7.807295284678898,7.807295284778898,9.9682205022335,9.9682205023335,11.928824276927505,11.928824277027505,13.82981995389362,13.82981995399362,15.434192679615805,15.434192679715805,16.91324989760243,16.91324989770243,18.37449686363349,18.374496863733487,19.80642421445182,19.806424214551818,21.200936380986715,21.200936381086713,22.590031994079116,22.590031994179114,23.95826119661094,23.95826119671094,25.317341430723094,25.31734143082309,26.666069740368922,26.66606974046892,27.99302129433779,27.99302129443779,29.294905845280475,29.294905845380473,30.580085563337487,30.580085563437486,31.83591965632131,31.835919656421307,33.0593779754874,33.0593779755874,34.19218542845685,34.192185428556854,35.31551228728567,35.31551228738567,36.40676248569214,36.40676248579214,37.48210765442233,37.48210765452233,38.55455272371032,38.554552723810325,39.62485848197481,39.62485848207481,40.67286078488785,40.672860784987854,41.71505638645139,41.71505638655139,42.75338302127028,42.75338302137028,43.76258511482996,43.76258511492996,44.7419278885722,44.7419278886722,45.70990435328985,45.70990435338985,46.674031358658226,46.67403135875823,47.62810555295565,47.628105553055654,48.54501165664409,48.545011656744094,49.450206820656746,49.45020682075675,50.28016196080015,50.28016196090015,51.07707807568388,51.07707807578388,51.86429901507811,51.86429901517811,52.64536862247162,52.645368622571624,53.40633911018837,53.40633911028837,54.134927321622264,54.134927321722266,54.851583509566396,54.8515835096664,55.55971496574209,55.55971496584209,56.249021785403954,56.249021785503956,56.93802949167044,56.93802949177044,57.62493690170412,57.62493690180412,58.31078440992373,58.31078441002373,58.991208862235716,58.99120886233572,59.662848484174596,59.6628484842746,60.32699726629864,60.32699726639864,60.98774525916647,60.987745259266475,61.63932477621929,61.63932477631929,62.264224678896895,62.2642246789969,62.88824024631858,62.88824024641858,63.51054566541449,63.51054566551449,64.13080280799852,64.13080280809852,64.73626684244131,64.73626684254131,65.33977363488385,65.33977363498386,65.92980081713878,65.92980081723879,66.51776021548649,66.51776021558649,67.10237734676404,67.10237734686405,67.67614186376126,67.67614186386126,68.23914480098978,68.23914480108978,68.7911975869612,68.7911975870612,69.33701450888539,69.33701450898539,69.88017192257671,69.88017192267671,70.3935155337064,70.39351553380641,70.90154012837037,70.90154012847037,71.40948669345292,71.40948669355292,71.91679601695401,71.91679601705401,72.41928701380337,72.41928701390337,72.88403770311344,72.88403770321344,73.34587528805108,73.34587528815108,73.80232232940669,73.8023223295067,74.2545687782967,74.2545687783967,74.70455236932597,74.70455236942597,75.15431487654128,75.15431487664128,75.60156743222139,75.60156743232139,76.0407244188308,76.04072441893081,76.46040652241469,76.46040652251469,76.8780793642774,76.8780793643774,77.28817683427876,77.28817683437876,77.6938396230703,77.6938396231703,78.0852490083253,78.08524900842531,78.465474153579,78.465474153679,78.84468491427442,78.84468491437443,79.22156128999285,79.22156129009285,79.59084278645456,79.59084278655456,79.95925945505572,79.95925945515572,80.31363730146917,80.31363730156917,80.6551012521602,80.6551012522602,80.99577840457206,80.99577840467207,81.3303367373088,81.3303367374088,81.65715063409114,81.65715063419114,81.96820255542977,81.96820255552977,82.27502137197722,82.27502137207722,82.5713972295467,82.5713972296467,82.85478116181235,82.85478116191236,83.12430183844846,83.12430183854846,83.38676083796747,83.38676083806747,83.64424545167195,83.64424545177195,83.8991225768645,83.8991225769645,84.1452018667537,84.1452018668537,84.38046755715325,84.38046755725325,84.61548615387836,84.61548615397837,84.83823459892763,84.83823459902763,85.06069043304662,85.06069043314662,85.28233345902598,85.28233345912598,85.50274751909822,85.50274751919822,85.72061911530972,85.72061911540972,85.91423001416952,85.91423001426952,86.10605923758726,86.10605923768726,86.28753653653868,86.28753653663868,86.46617876069908,86.46617876079908,86.63344167090465,86.63344167100465,86.79492388962117,86.79492388972118,86.9547219698724,86.9547219699724,87.10948714212302,87.10948714222302,87.26242512167575,87.26242512177575,87.41524605685639,87.41524605695639,87.56158403431533,87.56158403441533,87.7066735384718,87.7066735385718,87.85167200811664,87.85167200821664,87.9966054531103,87.99660545321031,88.14103820828996,88.14103820838996,88.28164101151567,88.28164101161568,88.41724341906638,88.41724341916638,88.55244267377984,88.55244267387984,88.68335030151606,88.68335030161606,88.8109871892984,88.8109871893984,88.93255727712655,88.93255727722655,89.05395830086165,89.05395830096165,89.17340858506164,89.17340858516164,89.28822261163319,89.28822261173319,89.39845890276233,89.39845890286233,89.5071736170541,89.5071736171541,89.59927453297182,89.59927453307182,89.69088776400578,89.69088776410578,89.77812483601596,89.77812483611596,89.86337215370033,89.86337215380033,89.94832686045443,89.94832686055443,90.02796905320791,90.02796905330791,90.10458109721687,90.10458109731687,90.17698604629508,90.17698604639509,90.2479799604429,90.2479799605429,90.31825860342785,90.31825860352785,90.3884982316221,90.3884982317221,90.4565075142812,90.4565075143812,90.52402260959141,90.52402260969141,90.59073790169222,90.59073790179222,90.65476117323458,90.65476117333458,90.70863409672924,90.70863409682924,90.76142110854936,90.76142110864936,90.81409107599737,90.81409107609737,90.8633732591194,90.8633732592194,90.91260342252049,90.91260342262049,90.96157998978202,90.96157998988203,91.01005586722954,91.01005586732954,91.05813509430493,91.05813509440493,91.10567461677559,91.10567461687559,91.14961177357142,91.14961177367142,91.18792429804101,91.18792429814101,91.22623032004547,91.22623032014548,91.26403565223595,91.26403565233595,91.301041181217,91.301041181317,91.33769557708175,91.33769557718175,91.37228218903928,91.37228218913928,91.4054967808571,91.40549678095711,91.43848378639584,91.43848378649584,91.46998172742278,91.46998172752278,91.50131060435666,91.50131060445666,91.53188519533698,91.53188519543698,91.56099673166595,91.56099673176595,91.58999772608794,91.58999772618795,91.61885566627737,91.61885566637737,91.6470308476295,91.6470308477295,91.67410060991172,91.67410061001172,91.6998893865659,91.6998893866659,91.72494988712695,91.72494988722696,91.7492756091298,91.7492756092298,91.77286655257441,91.77286655267442,91.79632094425158,91.79632094435158,91.81957375951009,91.8195737596101,91.84229987509414,91.84229987519414,91.8644147589572,91.8644147590572,91.88593791849459,91.88593791859459,91.90630363924116,91.90630363934116,91.92598009868532,91.92598009878532,91.94552650882713,91.94552650892713,91.96394149003858,91.96394149013858,91.98197282580813,91.98197282590813,91.99866465376357,91.99866465386357,92.01534347678877,92.01534347688877,92.03189875297674,92.03189875307675,92.04827846260656,92.04827846270656,92.0645866451201,92.0645866452201,92.08087532023828,92.08087532033828,92.09692990661225,92.09692990671225,92.11297148805598,92.11297148815598,92.12846686242987,92.12846686252988,92.1438907096875,92.1438907097875,92.15930155201488,92.15930155211488,92.17467988201668,92.17467988211668,92.1896485567161,92.1896485568161,92.20447417718295,92.20447417728295,92.21851299937063,92.21851299947063,92.23223320076758,92.23223320086758,92.24553074193192,92.24553074203192,92.25854217463112,92.25854217473112,92.2715145925396,92.2715145926396,92.28402533542479,92.28402533552479,92.29630198956575,92.29630198966575,92.30825352045088,92.30825352055088,92.31934022347542,92.31934022357542,92.32973766519757,92.32973766529757,92.3395563875243,92.3395563876243,92.34916052850215,92.34916052860216,92.35865412757303,92.35865412767303,92.36694477059726,92.36694477069726,92.37439659562138,92.37439659572138,92.38169886394782,92.38169886404782,92.38888408790214,92.38888408800214,92.39602379460065,92.39602379470065,92.40308547171776,92.40308547181776,92.41011463650928,92.41011463660928,92.41700074706822,92.41700074716822,92.4236722762783,92.4236722763783,92.43032429809303,92.43032429819303,92.4369568125124,92.43695681261241,92.4434397702341,92.4434397703341,92.44990972302554,92.44990972312554,92.46251800288748,92.46251800298748,92.46862381763238,92.46862381773238,92.47392982916789,92.47392982926789,92.47905377168011,92.47905377178012,92.48415820679699,92.48415820689699,92.48924313451852,92.48924313461852,92.49365180586787,92.49365180596787,92.49802796489163,92.49802796499164,92.50224806475259,92.5022480648526,92.50634461777632,92.50634461787632,92.51033062889307,92.51033062899307,92.514271122754,92.514271122854,92.51814659196374,92.51814659206374,92.52188550940605,92.52188550950605,92.52551388494135,92.52551388504135,92.52897319638362,92.52897319648362,92.53208137470956,92.53208137480956,92.53825221410563,92.53825221420563,92.54130187024552,92.54130187034552,92.54424098447842,92.54424098457842,92.54707605926944,92.54707605936945,92.5498070946186,92.5498070947186,92.5524731053166,92.5524731054166,92.55508059382852,92.55508059392852,92.55764906754975,92.55764906764975,92.56017852648029,92.56017852658029,92.56268197555035,92.56268197565035,92.56740926769042,92.56740926779042,92.56958109103952,92.56958109113953,92.57162286508627,92.57162286518627,92.57361261941209,92.57361261951209,92.57553734908673,92.57553734918673,92.5774360689009,92.5774360690009,92.57928276899413,92.57928276909414,92.58110996169202,92.58110996179202,92.59193006364679,92.59193006374679,92.59343213308881,92.59343213318881,92.59482366062386,92.59482366072386,92.59609164132168,92.59609164142168,92.59734011462416,92.59734011472416,92.59839351397312,92.59839351407312,92.59921932704297,92.59921932714298,92.5998760760198,92.5998760761198,92.60043528801987,92.60043528811987],[0.0,1e-10,2.4298561284698055,2.4298561285698055,4.849363041997334,4.849363042097334,7.133277151265534,7.133277151365534,9.372331742082318,9.372331742182318,11.51947083339928,11.51947083349928,13.541211290980582,13.541211291080582,15.557667317960895,15.557667318060895,17.558470318845618,17.558470318945616,19.533361521269995,19.533361521369994,21.379798422875254,21.379798422975252,23.19573033511882,23.19573033521882,25.009724192129752,25.00972419222975,26.68431373625717,26.68431373635717,28.34371538754483,28.343715387644828,29.973335590090983,29.97333559019098,31.56361973159873,31.56361973169873,33.134898011458425,33.13489801155843,34.516427763674834,34.516427763774836,35.86875748371953,35.86875748381953,37.20668745338573,37.206687453485735,38.54048290522232,38.54048290532232,39.87403287006276,39.874032870162765,41.15402790864137,41.15402790874137,42.41653522883749,42.41653522893749,43.66658085388776,43.66658085398776,44.8617665954862,44.861766595586204,46.014819016327145,46.01481901642715,47.09412843556628,47.094128435666285,48.1637863397469,48.1637863398469,49.23066636476073,49.23066636486073,50.27774592548113,50.27774592558113,51.29231137958201,51.29231137968201,52.30112726982606,52.30112726992606,53.28703534722036,53.28703534732036,54.249098885069124,54.249098885169126,55.177711589602595,55.1777115897026,56.04196794004432,56.041967940144325,56.87514434473868,56.87514434483868,57.702215875450236,57.70221587555024,58.52470713562866,58.52470713572866,59.341494053239025,59.34149405333903,60.135276255238,60.135276255338,60.91321808580223,60.91321808590223,61.68799442615315,61.687994426253155,62.43693649025304,62.43693649035304,63.16382994598963,63.163829946089635,63.88883056778235,63.88883056788235,64.602603389594,64.602603389694,65.30262893962215,65.30262893972215,65.97063135702304,65.97063135712304,66.62726385039247,66.62726385049247,67.2575775537027,67.2575775538027,67.88043620455139,67.88043620465139,68.5022741463109,68.5022741464109,69.11809765666511,69.11809765676512,69.73281647553674,69.73281647563674,70.3421991823345,70.3421991824345,70.9348500122905,70.9348500123905,71.51558180256579,71.51558180266579,72.01992837593879,72.01992837603879,72.50609599122966,72.50609599132966,72.97906545038627,72.97906545048627,73.45081393474632,73.45081393484632,73.91516550830183,73.91516550840183,74.37881938197357,74.37881938207357,74.83769271940481,74.83769271950482,75.28657861220387,75.28657861230387,75.73473450419864,75.73473450429864,76.18006083555375,76.18006083565375,76.62256406645334,76.62256406655334,77.05597781831185,77.05597781841185,77.48311873140072,77.48311873150072,77.90108618305513,77.90108618315513,78.31283893759688,78.31283893769688,78.71433291977388,78.71433291987388,79.11538760943148,79.11538760953148,79.51389698655022,79.51389698665022,79.8899378433386,79.8899378434386,80.2634204672199,80.2634204673199,80.61835590252487,80.61835590262487,80.96726398605634,80.96726398615634,81.29673337560448,81.29673337570448,81.61444523007461,81.61444523017461,81.92438548306188,81.92438548316188,82.23272361039017,82.23272361049017,82.53992474531532,82.53992474541532,82.84104684699409,82.84104684709409,83.13655504868234,83.13655504878234,83.4311200634907,83.4311200635907,83.72156994102176,83.72156994112176,84.00500405861067,84.00500405871067,84.28053737103454,84.28053737113454,84.5483443032643,84.5483443033643,84.81421318026142,84.81421318036142,85.07154815405084,85.07154815415085,85.32652516064056,85.32652516074056,85.57810411038905,85.57810411048905,85.79651647492302,85.79651647502303,86.01070387904984,86.01070387914984,86.22323101586072,86.22323101596072,86.43500231113086,86.43500231123086,86.64397634668191,86.64397634678191,86.85200073516896,86.85200073526896,87.05749919166946,87.05749919176947,87.25697675658058,87.25697675668059,87.45478759399164,87.45478759409164,87.64940710045296,87.64940710055296,87.84301235800919,87.84301235810919,88.03206318576873,88.03206318586874,88.218781887065,88.21878188716501,88.40508067639419,88.4050806764942,88.58978380024853,88.58978380034853,88.77275559476169,88.77275559486169,88.95444181263721,88.95444181273722,89.12649589600655,89.12649589610655,89.29573333910443,89.29573333920443,89.46042927277385,89.46042927287385,89.62394945293546,89.62394945303546,89.78415555864926,89.78415555874926,89.9441613986557,89.9441613987557,90.09709333706301,90.09709333716302,90.24998651436567,90.24998651446568,90.3999080069783,90.3999080070783,90.54659940753652,90.54659940763652,90.68509283446069,90.68509283456069,90.81333394920422,90.81333394930422,90.92555380735796,90.92555380745796,91.03708242581206,91.03708242591206,91.14648564369436,91.14648564379436,91.2467993825356,91.2467993826356,91.34370214416741,91.34370214426741,91.44018499383213,91.44018499393214,91.52947119839968,91.52947119849968,91.60647659297642,91.60647659307642,91.679315168803,91.679315168903,91.75113949572449,91.75113949582449,91.82171700711298,91.82171700721298,91.89159035843363,91.89159035853363,91.95717414750602,91.95717414760603,92.0212656340493,92.0212656341493,92.08189446191027,92.08189446201027,92.14059815490681,92.14059815500681,92.19905636090724,92.19905636100724,92.25147429476593,92.25147429486593,92.30044895049464,92.30044895059464,92.34836413602952,92.34836413612952,92.39558808186474,92.39558808196475,92.44073184841695,92.44073184851695,92.48449959575397,92.48449959585398,92.52712389050376,92.52712389060376,92.56955437973026,92.56955437983027,92.60977548599158,92.60977548609158,92.64906632574122,92.64906632584122,92.68789849241914,92.68789849251914,92.72672419891295,92.72672419901295,92.76542716190869,92.76542716200869,92.8041107443521,92.8041107444521,92.8427814064273,92.84278140652731,92.88106445745598,92.88106445755598,92.91871441044198,92.91871441054198,92.95591215054039,92.95591215064039,92.99310343045467,92.99310343055467,93.03024948908019,93.03024948918019,93.0672469634712,93.06724696357121,93.10419921657348,93.10419921667348,93.14109978820287,93.14109978830287,93.17668894245817,93.17668894255817,93.21123154688786,93.21123154698786,93.24421724694731,93.24421724704732,93.2758204676075,93.2758204677075,93.30733970587428,93.30733970597429,93.33840673125343,93.33840673135343,93.36795561336703,93.36795561346703,93.39678095486043,93.39678095496043,93.42542541119879,93.42542541129879,93.45272614924252,93.45272614934252,93.47969741789672,93.47969741799672,93.50625523476793,93.50625523486794,93.5321799535965,93.5321799536965,93.5578462650607,93.5578462651607,93.58294408032334,93.58294408042335,93.60782224932629,93.60782224942629,93.6315117444532,93.63151174455321,93.65476194706073,93.65476194716074,93.67785710524964,93.67785710534964,93.70058403294435,93.70058403304435,93.72268432278051,93.72268432288051,93.7440675321806,93.7440675322806,93.76528277679385,93.76528277689386,93.78631067606796,93.78631067616796,93.80682822079747,93.80682822089747,93.82671266748432,93.82671266758432,93.8458218920781,93.84582189217811,93.86300598180748,93.86300598190748,93.88003502711824,93.88003502721824,93.89676690396,93.89676690406,93.91282692165444,93.91282692175444,93.92871251437795,93.92871251447795,93.9441846553185,93.9441846554185,93.95945653055166,93.95945653065166,93.97468318449607,93.97468318459607,93.98904417376991,93.98904417386991,94.00308861402242,94.00308861412242,94.01704261169739,94.0170426117974,94.03097076863594,94.03097076873594,94.04427874790004,94.04427874800004,94.05592645984818,94.05592645994818,94.0674191273777,94.0674191274777,94.07787816544983,94.07787816554983,94.08804649523707,94.08804649533707,94.09812438244677,94.09812438254677,94.10765961419135,94.10765961429135,94.11640024329053,94.11640024339053,94.12489538539359,94.12489538549359,94.13305459792299,94.133054598023,94.14110398732254,94.14110398742254,94.1490241730399,94.1490241731399,94.1568926772844,94.1568926773844,94.16428312790487,94.16428312800487,94.17147331281794,94.17147331291794,94.17853429404884,94.17853429414885,94.18539500957237,94.18539500967238,94.19185519368116,94.19185519378117,94.19824431576475,94.19824431586476,94.20431042864291,94.20431042874291,94.21036362115284,94.21036362125284,94.21639743311044,94.21639743321045,94.22236664322696,94.22236664332696,94.2282777116865,94.2282777117865,94.23409187738442,94.23409187748442,94.23957657369277,94.23957657379277,94.24499666816004,94.24499666826004,94.25014543489475,94.25014543499475,94.25493889150347,94.25493889160347,94.25931889632923,94.25931889642924,94.26799492358732,94.26799492368733,94.27225218491502,94.27225218501502,94.27645776476984,94.27645776486985,94.28055998167892,94.28055998177892,94.28429396809379,94.2842939681938,94.29167149834603,94.29167149844604,94.29506955518725,94.29506955528726,94.29845469166025,94.29845469176026,94.30174292537163,94.30174292547163,94.30495363687369,94.30495363697369,94.30780257806568,94.30780257816568,94.31044479336617,94.31044479346617,94.31303532719379,94.31303532729379,94.315541878628,94.315541878728,94.31801612914167,94.31801612924167,94.32045161855068,94.32045161865068,94.32284188667093,94.32284188677093,94.32518693350242,94.32518693360242,94.3275255201498,94.32752552024981,94.32979950495609,94.32979950505609,94.3318667638709,94.33186676397091,94.33387588112873,94.33387588122874,94.33586561783424,94.33586561793425,94.33768738975292,94.33768738985292,94.33947040056695,94.33947040066695,94.34301058145856,94.34301058155856,94.34474191079971,94.34474191089971,94.34562695602261,94.34562695612262],[0.0,1e-10,2.3584282105919088,2.3584282106919088,4.333513962292278,4.333513962392278,6.252786055820811,6.252786055920811,7.949073299407212,7.949073299507212,9.345029607401484,9.345029607501484,10.686401964620691,10.686401964720691,11.913003722813663,11.913003722913663,13.1384447878005,13.1384447879005,14.297305980876418,14.297305980976418,15.455916213259119,15.455916213359119,16.549157459075683,16.54915745917568,17.575091046970996,17.575091047070995,18.586042281481152,18.58604228158115,19.595180324982806,19.595180325082804,20.574899501221896,20.574899501321894,21.546111109960876,21.546111110060874,22.47833597500833,22.47833597510833,23.407022294281404,23.407022294381402,24.27853349362193,24.278533493721927,25.133161312326166,25.133161312426164,25.981747252341165,25.981747252441163,26.8263868354553,26.826386835555297,27.664168917627233,27.66416891772723,28.48062561489291,28.48062561499291,29.258139486588377,29.258139486688375,30.029322874797405,30.029322874897403,30.800267850347872,30.80026785044787,31.5702779973161,31.5702779974161,32.331931153200145,32.33193115330015,33.09130056677591,33.09130056687591,33.84462810166242,33.844628101762424,34.57846853872051,34.57846853882051,35.306994883099684,35.306994883199685,36.032409314882955,36.03240931498296,36.757271633141144,36.757271633241146,37.46910909142128,37.46910909152128,38.169640770471915,38.16964077057192,38.86328985251103,38.86328985261103,39.550639821150355,39.55063982125036,40.236885562739516,40.23688556283952,40.91052680351177,40.91052680361177,41.583791603244194,41.583791603344196,42.2557137632679,42.2557137633679,42.92112349330258,42.92112349340258,43.586012479898834,43.586012479998836,44.237901702586356,44.23790170268636,44.8882098729066,44.8882098730066,45.53515516993772,45.53515517003772,46.174672030449564,46.174672030549566,46.80182280280305,46.801822802903054,47.40986919238527,47.409869192485274,48.00312144910225,48.003121449202254,48.58546318968155,48.58546318978155,49.16089723718,49.160897237280004,49.732453941968224,49.732453942068226,50.298815760406825,50.29881576050683,50.86367808870344,50.86367808880344,51.4201896999332,51.420189700033205,51.97391564746824,51.97391564756824,52.5156833179714,52.5156833180714,53.05294624403129,53.05294624413129,53.5809863646154,53.5809863647154,54.096302778053314,54.096302778153316,54.60460484011577,54.60460484021577,55.10887898305206,55.10887898315206,55.605065917649384,55.605065917749386,56.0881338820087,56.0881338821087,56.55980823089588,56.559808230995884,57.02865299796703,57.02865299806703,57.49656921047327,57.49656921057327,57.95724520698015,57.95724520708015,58.41638406924106,58.41638406934106,58.87494572190757,58.874945722007574,59.314773158825304,59.314773158925306,59.74882849979901,59.74882849989901,60.18257641392353,60.18257641402353,60.61604199726817,60.61604199736817,61.04822768107739,61.048227681177394,61.4802439664187,61.4802439665187,61.90668892437055,61.90668892447055,62.32980237911992,62.32980237921992,62.7466982826948,62.7466982827948,63.16343733583641,63.163437335936415,63.57745973947394,63.57745973957394,63.9887905896767,63.9887905897767,64.39645741375845,64.39645741385846,64.79449989525529,64.79449989535529,65.18509511818084,65.18509511828084,65.55809799651178,65.55809799661178,65.92755605505099,65.92755605515099,66.29623613544123,66.29623613554124,66.6624944451419,66.6624944452419,67.02786184438166,67.02786184448166,67.38368646326177,67.38368646336177,67.73892132451282,67.73892132461282,68.09268806570854,68.09268806580855,68.44417106459596,68.44417106469596,68.79294996201396,68.79294996211397,69.13601322964391,69.13601322974391,69.460474035889,69.46047403598901,69.78417568603713,69.78417568613713,70.10568143011959,70.10568143021959,70.4247904995818,70.4247904996818,70.74058688789354,70.74058688799354,71.04381641949234,71.04381641959235,71.3440720668765,71.34407206697651,71.63607738147111,71.63607738157111,71.92608128453729,71.92608128463729,72.20464765401003,72.20464765411003,72.48104948750375,72.48104948760376,72.75487897389199,72.75487897399199,73.02676978892512,73.02676978902512,73.29679722081109,73.29679722091109,73.5591703515539,73.5591703516539,73.81984949761747,73.81984949771747,74.07256064167136,74.07256064177136,74.32323900411018,74.32323900421018,74.57111288080226,74.57111288090226,74.81896793544237,74.81896793554237,75.06649674118128,75.06649674128128,75.30802758635228,75.30802758645228,75.54845420447312,75.54845420457312,75.78475251919049,75.78475251929049,76.01964545402585,76.01964545412585,76.24248600156939,76.24248600166939,76.46202641599709,76.46202641609709,76.67954032282705,76.67954032292705,76.89648956809728,76.89648956819728,77.11108605686857,77.11108605696857,77.32543785896398,77.32543785906398,77.53875444819987,77.53875444829987,77.75143108766804,77.75143108776804,77.9580533004123,77.9580533005123,78.16436808630739,78.16436808640739,78.37037544535328,78.37037544545328,78.57593107515137,78.57593107525138,78.78047658815926,78.78047658825926,78.98234936978437,78.98234936988437,79.1835069134338,79.1835069135338,79.37756226946516,79.37756226956516,79.56878176966313,79.56878176976313,79.7563560657921,79.7563560658921,79.94194149842733,79.94194149852733,80.12375624664693,80.12375624674694,80.30413424489785,80.30413424499785,80.48444322895816,80.48444322905816,80.66377974033223,80.66377974043223,80.8363842311107,80.8363842312107,81.00869384307465,81.00869384317465,81.18092816683064,81.18092816693064,81.35161908232332,81.35161908242333,81.515778745775,81.515778745875,81.67894084047113,81.67894084057113,81.8404026764707,81.8404026765707,81.99936117955541,81.99936117965541,82.15747269030051,82.15747269040051,82.31447369997812,82.31447370007812,82.47038930465756,82.47038930475756,82.62623589514637,82.62623589524637,82.78127313739954,82.78127313749954,82.93368156639124,82.93368156649125,83.08265183388585,83.08265183398585,83.22755653815032,83.22755653825033,83.37050374900768,83.37050374910768,83.51294903847861,83.51294903857861,83.65509317511767,83.65509317521767,83.79642168950377,83.79642168960378,83.93759335345662,83.93759335355662,84.07861444099352,84.07861444109352,84.21797291393786,84.21797291403786,84.35275135423096,84.35275135433096,84.48555975308228,84.48555975318229,84.61706315632887,84.61706315642887,84.74777603339183,84.74777603349183,84.87534562777222,84.87534562787222,85.00253250709544,85.00253250719544,85.12636278714687,85.12636278724688,85.2496597757252,85.24965977582521,85.37096162679246,85.37096162689247,85.49210035340913,85.49210035350913,85.61278107676065,85.61278107686066,85.73139137439314,85.73139137449314,85.8488848969408,85.8488848970408,85.96623411708985,85.96623411718986,86.08334492458035,86.08334492468035,86.19937032707269,86.19937032717269,86.31392760950968,86.31392760960968,86.42453226102849,86.42453226112849,86.531410146253,86.531410146353,86.63818764720021,86.63818764730021,86.7412509298878,86.7412509299878,86.83862367885665,86.83862367895665,86.93481691256737,86.93481691266737,87.0294479159628,87.02944791606281,87.12144383207944,87.12144383217944,87.21314486938155,87.21314486948155,87.3007364253322,87.3007364254322,87.38805192452031,87.38805192462031,87.4738804816011,87.4738804817011,87.55526076039459,87.55526076049459,87.63484039621423,87.63484039631423,87.71374871217952,87.71374871227952,87.7925127257462,87.79251272584621,87.8709065722904,87.8709065723904,87.94853498872025,87.94853498882026,88.02453843466154,88.02453843476154,88.10004623323371,88.10004623333371,88.17320754932429,88.17320754942429,88.24604261651368,88.24604261661368,88.31839458436862,88.31839458446862,88.38912158173498,88.38912158183498,88.45940939788821,88.45940939798821,88.52956545967749,88.52956545977749,88.59962113718949,88.59962113728949,88.66878590424055,88.66878590434055,88.73766834051175,88.73766834061175,88.80594847111922,88.80594847121922,88.87385216068687,88.87385216078687,88.94126647690274,88.94126647700274,89.00773341650171,89.00773341660171,89.07283889433997,89.07283889443997,89.13410467357197,89.13410467367197,89.19532653468268,89.19532653478268,89.25646055955075,89.25646055965075,89.31549278861311,89.31549278871312,89.373395694556,89.373395694656,89.43120449023891,89.43120449033891,89.48885016147125,89.48885016157125,89.54603782943846,89.54603782953846,89.60190795376626,89.60190795386626,89.65652327462799,89.65652327472799,89.70968929748636,89.70968929758637,89.76283649829274,89.76283649839274,89.81544413360871,89.81544413370871,89.86653973074803,89.86653973084803,89.91573430063623,89.91573430073623,89.96468418384853,89.96468418394853,90.01242945573338,90.01242945583338,90.10771923094852,90.10771923104852,90.20256982495053,90.20256982505053,90.24954966672107,90.24954966682107,90.29481042774306,90.29481042784306,90.33918027830411,90.33918027840411,90.38304193346141,90.38304193356142,90.42645185937091,90.42645185947092,90.46907125909678,90.46907125919678,90.5107558302404,90.5107558303404,90.55095973329404,90.55095973339404,90.59016606759212,90.59016606769212,90.62841875125598,90.62841875135598,90.66574915437226,90.66574915447227,90.70306700945387,90.70306700955388,90.73860931763097,90.73860931773098,90.77273369789138,90.77273369799138,90.80656319933726,90.80656319943726,90.8387363602079,90.8387363603079,90.87079658876658,90.87079658886658,90.90216040140159,90.90216040150159,90.93322933522207,90.93322933532207,90.96415396664393,90.96415396674394,90.99419396162222,90.99419396172222,91.02362537691944,91.02362537701944,91.05180198875055,91.05180198885056,91.07996605254702,91.07996605264702,91.10653024192423,91.10653024202423,91.13262388000165,91.13262388010165,91.1580461982247,91.1580461983247,91.18322382977186,91.18322382987186,91.20759838710072,91.20759838720072,91.23167806561506,91.23167806571506,91.25515543846566,91.25515543856567,91.27833793250174,91.27833793260174,91.30138239815655,91.30138239825655,91.32410061491017,91.32410061501017,91.34679373559446,91.34679373569446,91.36919825148156,91.36919825158157,91.39120750427684,91.39120750437684,91.41256425926976,91.41256425936976,91.43380808195072,91.43380808205072,91.45456880529723,91.45456880539723,91.47495308760392,91.47495308770392,91.49518051947734,91.49518051957735,91.51285442629727,91.51285442639727,91.52982564317618,91.52982564327618,91.54602515592345,91.54602515602345,91.56193606387352,91.56193606397352,91.57726976222918,91.57726976232918,91.5921015391984,91.5921015392984,91.62119415755977,91.62119415765977,91.63478995311489,91.63478995321489,91.6483418305487,91.6483418306487,91.66173058353192,91.66173058363192,91.67473662145798,91.67473662155798,91.6877363853667,91.6877363854667,91.7132904579537,91.7132904580537,91.72536794131484,91.72536794141485,91.73743915065867,91.73743915075868,91.76105455189055,91.76105455199055,91.77170783331768,91.77170783341768,91.7820097697743,91.7820097698743,91.79191016912178,91.79191016922178,91.80160352589735,91.80160352599735,91.8209086772232,91.8209086773232,91.8304640056175,91.8304640057175,91.83973700323193,91.83973700333193,91.84895980870772,91.84895980880772,91.8581198740102,91.8581198741102,91.866602345441,91.866602345541,91.87507226883713,91.87507226893713,91.88336024573067,91.88336024583067,91.89146627612163,91.89146627622164,91.89910175521281,91.89910175531281,91.90663057600938,91.90663057610938,91.91403391645933,91.91403391655933,91.92141843485729,91.92141843495729,91.92867119889131,91.92867119899131,91.93585494873469,91.93585494883469,91.94276264181553,91.94276264191554,91.94960759472308,91.94960759482308,91.9560572845388,91.9560572846388,91.96218699947066,91.96218699957066,91.967984191484,91.96798419158401,91.97352414878682,91.97352414888682,91.97905783207229,91.97905783217229,91.98378844113945,91.98378844123945,91.9877724421443,91.9877724422443,91.9916435108372,91.99164351093721,91.99535145507951,91.99535145517952,91.99893391897521,91.99893391907521,92.00247873876692,92.00247873886693,92.00598591445466,92.00598591455466,92.00942407595174,92.00942407605174,92.01284968941418,92.01284968951418,92.01965699821774,92.01965699831774,92.0230261455242,92.0230261456242,92.02635137470935,92.02635137480935,92.02964523380784,92.02964523390784,92.0328073385424,92.0328073386424,92.03590670310365,92.03590670320365,92.03892450543961,92.03892450553961,92.04486599985155,92.04486599995155,92.0476955816676,92.0476955817676,92.05039340911969,92.0503934092197,92.05298457827718,92.05298457837718,92.0607518117323,92.0607518118323,92.0633053367858,92.0633053368858,92.06584631380464,92.06584631390464,92.06823671440755,92.06823671450755,92.07061456697579,92.0706145670758,92.07297987150938,92.07297987160938,92.07532007997365,92.07532008007365,92.07759127424727,92.07759127434727,92.07973698817429,92.07973698827429,92.08186388004933,92.08186388014933,92.08398449790703,92.08398449800703,92.08811280131047,92.08811280141047,92.0900953907869,92.0900953908869,92.092052884194,92.092052884294,92.09592395288689,92.0959239529869,92.09776851398206,92.09776851408206,92.10141371805106,92.10141371815106,92.10314534683427,92.10314534693427,92.10483305749617,92.10483305759617,92.10648312405408,92.10648312415408,92.10811436856,92.10811436866,92.10946328228604,92.10946328238605,92.110297726591,92.110297726691],[0.0,1e-10,2.888001201662334,2.888001201762334,5.555280037566229,5.555280037666229,8.010145322468347,8.010145322568347,10.437890116703795,10.437890116803795,12.784805722384583,12.784805722484583,14.880911965121673,14.880911965221673,16.861545156525736,16.861545156625734,18.7901443955162,18.7901443956162,20.707591647200456,20.707591647300454,22.610011628445903,22.6100116285459,24.467596466313015,24.467596466413013,26.291647447123157,26.291647447223156,28.100749052632835,28.100749052732834,29.903852732490048,29.903852732590046,31.674123611535386,31.674123611635384,33.43772147372928,33.437721473829285,35.18640241693035,35.18640241703035,36.68829857933549,36.68829857943549,38.15473298000964,38.15473298010964,39.59648111309037,39.59648111319037,40.94633894797163,40.946338948071634,42.29125044156807,42.29125044166807,43.63129348901802,43.63129348911802,44.934706347661844,44.934706347761846,46.223604745527616,46.22360474562762,47.491997248224415,47.49199724832442,48.7468814356801,48.746881435780104,49.97712479437306,49.97712479447306,51.1860833065136,51.186083306613604,52.38620721171366,52.38620721181366,53.5647736373771,53.5647736374771,54.694318055976225,54.69431805607623,55.77447046560391,55.774470465703914,56.814532844030694,56.814532844130696,57.83445932919563,57.83445932929563,58.84838139744657,58.848381397546575,59.838908959148284,59.838908959248286,60.829248274265666,60.82924827436567,61.796387820679676,61.79638782077968,62.66740476637755,62.66740476647755,63.51245666596085,63.51245666606085,64.34320182513503,64.34320182523503,65.1609839350365,65.1609839351365,65.96505000932794,65.96505000942794,66.76261833082923,66.76261833092923,67.53931075525439,67.53931075535439,68.30324135951423,68.30324135961423,69.05946034507807,69.05946034517807,69.80075592038753,69.80075592048753,70.52956879977742,70.52956879987742,71.25131269536263,71.25131269546263,71.96178776093411,71.96178776103412,72.64725848709726,72.64725848719726,73.31992844552592,73.31992844562592,73.98558135024211,73.98558135034212,74.61973865402135,74.61973865412135,75.24579486341283,75.24579486351283,75.85453238704588,75.85453238714588,76.4405504953287,76.4405504954287,77.0255235105054,77.0255235106054,77.54033897108052,77.54033897118052,78.0404582155548,78.04045821565481,78.5260759817741,78.5260759818741,79.01044742577989,79.01044742587989,79.46899663142474,79.46899663152475,79.91982772711204,79.91982772721204,80.36968513357004,80.36968513367005,80.81709533443174,80.81709533453174,81.25505425850774,81.25505425860774,81.67654485208557,81.67654485218557,82.09001873767554,82.09001873777554,82.49359994069583,82.49359994079583,82.86432886911967,82.86432886921968,83.22415896943693,83.22415896953693,83.55521330739771,83.55521330749771,83.88455395231493,83.88455395241493,84.19865960642443,84.19865960652443,84.51030507241457,84.51030507251457,84.81234347134232,84.81234347144232,85.10432041490067,85.10432041500067,85.3901306600068,85.3901306601068,85.6713775482583,85.67137754835831,85.95059916291288,85.95059916301288,86.18553739141905,86.18553739151905,86.41738577943758,86.41738577953758,86.63719287732047,86.63719287742047,86.8509111718895,86.85091117198951,87.04494796150371,87.04494796160371,87.23789421918109,87.23789421928109,87.43080802055081,87.43080802065082,87.62261181619915,87.62261181629916,87.81337701000291,87.81337701010291,88.00331132233099,88.00331132243099,88.19084386789348,88.19084386799348,88.3782011493947,88.3782011494947,88.5590736606288,88.5590736607288,88.72897593987949,88.72897593997949,88.89273099446257,88.89273099456257,89.05457761815622,89.05457761825622,89.21554143028197,89.21554143038198,89.37605734536224,89.37605734546224,89.53635904881207,89.53635904891208,89.69493407669529,89.69493407679529,89.84870557104732,89.84870557114732,89.99881599389718,89.99881599399718,90.1257331393052,90.1257331394052,90.24842447345807,90.24842447355807,90.36374822577594,90.36374822587594,90.47632617446719,90.47632617456719,90.58423690611936,90.58423690621936,90.69158289801851,90.69158289811851,90.79121727122165,90.79121727132166,90.89013760565665,90.89013760575665,90.98749354606322,90.98749354616322,91.08430422050141,91.08430422060141,91.17733048946839,91.17733048956839,91.2634305823843,91.2634305824843,91.34950471025411,91.34950471035411,91.42838652035017,91.42838652045018,91.50611937715567,91.50611937725567,91.58339135439263,91.58339135449263,91.65927420166247,91.65927420176247,91.73263194819765,91.73263194829765,91.80388003473604,91.80388003483604,91.8717916128487,91.8717916129487,91.93910599490073,91.93910599500073,92.00551160033874,92.00551160043874,92.07166404657714,92.07166404667714,92.13510963675809,92.13510963685809,92.19751013383294,92.19751013393294,92.2576062330651,92.2576062331651,92.31752057697449,92.31752057707449,92.37566929774806,92.37566929784806,92.43272099532331,92.43272099542331,92.48779285813231,92.48779285823231,92.53935294845432,92.53935294855432,92.59062742326907,92.59062742336907,92.64159031753044,92.64159031763045,92.69080706244061,92.69080706254061,92.73887485406021,92.73887485416022,92.78413842069943,92.78413842079944,92.82838935054019,92.8283893506402,92.87234168235064,92.87234168245064,92.91583313459255,92.91583313469255,92.9592921305268,92.9592921306268,93.00165410326272,93.00165410336272,93.0436655478761,93.0436655479761,93.0841645285533,93.0841645286533,93.1235080646784,93.1235080647784,93.16226089600441,93.16226089610441,93.20100723606888,93.20100723616888,93.23965620721042,93.23965620731042,93.27744833183017,93.27744833193017,93.31520150888076,93.31520150898076,93.35179275011772,93.35179275021773,93.38762451375582,93.38762451385583,93.42097012422846,93.42097012432846,93.45222554848888,93.45222554858888,93.48327325238037,93.48327325248037,93.51404832328767,93.51404832338767,93.54450532238006,93.54450532248006,93.57396915845858,93.57396915855858,93.60340702949097,93.60340702959097,93.6326501626775,93.63265016277751,93.66008223389755,93.66008223399756,93.686131666412,93.686131666512,93.71208373000351,93.71208373010352,93.73637403064369,93.73637403074369,93.76037222451508,93.76037222461508,93.78409129414074,93.78409129424074,93.80748580068997,93.80748580078998,93.82960801997959,93.82960802007959,93.8510940956394,93.8510940957394,93.87228157326888,93.87228157336888,93.89220974616181,93.89220974626181,93.91194967247041,93.91194967257042,93.93087819108794,93.93087819118794,93.94814494675413,93.94814494685413,93.96523643835904,93.96523643845904,93.9813152931655,93.9813152932655,93.9971799363415,93.9971799364415,94.01258369994896,94.01258370004896,94.02798097229491,94.02798097239491,94.04332631454862,94.04332631464862,94.05793165298806,94.05793165308806,94.07247857007376,94.07247857017376,94.08672688912912,94.08672688922913,94.10080643538475,94.10080643548476,94.11487949037884,94.11487949047884,94.12894605411141,94.12894605421141,94.14298665279787,94.14298665289787,94.15700777769973,94.15700777779973,94.17091205989409,94.1709120599941,94.1848033595654,94.1848033596654,94.1969290361009,94.1969290362009,94.20686066623972,94.20686066633972,94.21653913717893,94.21653913727893,94.22606830910297,94.22606830920297,94.23548712958103,94.23548712968103,94.24464629959795,94.24464629969795,94.25318230850812,94.25318230860812,94.26169235237217,94.26169235247217,94.26959870891405,94.26959870901405,94.27738822274841,94.27738822284842,94.28508036765986,94.28508036775986,94.29237654561805,94.29237654571806,94.29919237022312,94.29919237032313,94.30576152689011,94.30576152699011,94.31184383894245,94.31184383904245,94.31787422090257,94.31787422100257,94.32341775824803,94.32341775834803,94.32891585676279,94.32891585686279,94.33439448149296,94.33439448159297,94.33984714117702,94.33984714127702,94.34515050184592,94.34515050194592,94.3502915809766,94.3502915810766,94.3550496756771,94.3550496757771,94.3595351373934,94.3595351374934,94.3638907738791,94.36389077397911,94.37249169540456,94.37249169550456,94.37672399792123,94.37672399802123,94.3808394577304,94.3808394578304,94.38452000301714,94.38452000311715,94.38816809199623,94.38816809209624,94.39174477709852,94.39174477719853,94.39873586576486,94.39873586586486,94.40207886545211,94.40207886555211,94.40541537387784,94.40541537397785,94.40863503959605,94.40863503969605,94.4118417227912,94.4118417228912,94.4179370173666,94.4179370174666,94.42078018991614,94.42078019001615,94.42345458966594,94.42345458976594,94.42603811175434,94.42603811185434,94.42846584356604,94.42846584366605,94.43081568023942,94.43081568033942,94.43312656934361,94.43312656944362,94.43522324681737,94.43522324691737,94.43730045050653,94.43730045060653,94.43935168914959,94.43935168924959,94.44133801517735,94.44133801527735,94.44323995480524,94.44323995490524,94.44500557794103,94.44500557804103,94.44673225350765,94.44673225360765,94.44840699898204,94.44840699908204,94.45315211115948,94.45315211125948,94.45449580229591,94.45449580239591,94.45562528180189,94.45562528190189,94.45672879626176,94.45672879636176,94.45867617472035,94.45867617482035,94.46007828721054,94.46007828731054],[0.0,1e-10,5.248244285476673,5.248244285576673,8.95402772154319,8.95402772164319,12.582958104917836,12.582958105017836,15.871427233547024,15.871427233647024,18.946263363776833,18.94626336387683,21.937947742346747,21.937947742446745,24.66719695591784,24.66719695601784,27.297190659131434,27.297190659231433,29.902620711409998,29.902620711509996,32.087210969885525,32.08721096998553,34.2068570518557,34.2068570519557,36.1945619659693,36.1945619660693,38.13867325301472,38.138673253114725,39.957065568754984,39.957065568854986,41.75355269253762,41.75355269263762,43.54416188064361,43.54416188074361,45.334057046194516,45.33405704629452,47.074595402625526,47.07459540272553,48.765955455575394,48.765955455675396,50.3099336029151,50.309933603015104,51.81855488337557,51.81855488347557,53.18382976070235,53.183829760802354,54.535812343142155,54.535812343242156,55.87329134243189,55.87329134253189,57.20591243826654,57.20591243836654,58.484044687866636,58.48404468796664,59.68082299259731,59.68082299269731,60.87019331331903,60.87019331341903,62.053099179836714,62.053099179936716,63.23282382086347,63.232823820963475,64.38975711694013,64.38975711704013,65.54295454500539,65.54295454510539,66.64492722994504,66.64492723004504,67.64061893260131,67.64061893270132,68.63622775763957,68.63622775773958,69.60854159681837,69.60854159691837,70.56429266279986,70.56429266289986,71.48493549475612,71.48493549485612,72.40355101267205,72.40355101277206,73.26690628498591,73.26690628508591,74.07534557257246,74.07534557267246,74.87774754444774,74.87774754454774,75.6782114551021,75.6782114552021,76.46758251534717,76.46758251544718,77.23056661706123,77.23056661716123,77.97857537072274,77.97857537082274,78.68332200778808,78.68332200788808,79.34977918533724,79.34977918543724,79.97488043150423,79.97488043160423,80.59716383865921,80.59716383875922,81.1960311311284,81.1960311312284,81.75994956960697,81.75994956970698,82.31748643149952,82.31748643159952,82.8087148237904,82.8087148238904,83.29308349939139,83.29308349949139,83.75594224552061,83.75594224562062,84.21281467754964,84.21281467764965,84.65294583074261,84.65294583084261,85.09085841385372,85.09085841395373,85.49655710436824,85.49655710446824,85.88158101679234,85.88158101689234,86.22510874340382,86.22510874350382,86.5607822219094,86.56078222200941,86.86252687864594,86.86252687874594,87.15400746115318,87.15400746125319,87.43831594210272,87.43831594220272,87.71459804450897,87.71459804460898,87.98634737873076,87.98634737883076,88.25239090771329,88.2523909078133,88.46798746813913,88.46798746823913,88.67773796889526,88.67773796899526,88.88372072563307,88.88372072573307,89.07094763989726,89.07094763999726,89.24015823504844,89.24015823514844,89.40442167392516,89.40442167402516,89.56436910146445,89.56436910156445,89.72276735506729,89.72276735516729,89.8809233510175,89.8809233511175,90.03647826480278,90.03647826490278,90.18773629285482,90.18773629295482,90.33785315985901,90.33785315995901,90.47693455326637,90.47693455336638,90.61378462618912,90.61378462628912,90.74480138984491,90.74480138994491,90.87579265269515,90.87579265279516,91.00391507492232,91.00391507502232,91.12761310738854,91.12761310748854,91.24806616234999,91.24806616244999,91.36241814958632,91.36241814968632,91.47514446046958,91.47514446056958,91.58141269235018,91.58141269245019,91.68235763107462,91.68235763117463,91.77588821058872,91.77588821068872,91.85686601857644,91.85686601867644,91.9307354770203,91.93073547712031,92.00416504656862,92.00416504666862,92.075988065368,92.075988065468,92.14730106805662,92.14730106815662,92.21594923656644,92.21594923666645,92.28455915386797,92.28455915396798,92.34738038831225,92.34738038841225,92.40610236826619,92.40610236836619,92.45950743026533,92.45950743036533,92.5120327144734,92.51203271457341,92.55940683596269,92.55940683606269,92.60394399283581,92.60394399293581,92.64806038641753,92.64806038651753,92.69127150140264,92.69127150150264,92.73245530234743,92.73245530244743,92.77129302918267,92.77129302928267,92.80991399917085,92.80991399927085,92.84624627186192,92.84624627196192,92.88250204213638,92.88250204223638,92.91694088001623,92.91694088011623,92.95106733302822,92.95106733312822,92.98083314829312,92.98083314839312,93.009107166434,93.00910716653401,93.03469722479196,93.03469722489196,93.05979001744191,93.05979001754191,93.0834165137734,93.0834165138734,93.10588272345288,93.10588272355288,93.12793454504236,93.12793454514237,93.14959747934738,93.14959747944738,93.17013200300731,93.17013200310731,93.18886234467539,93.18886234477539,93.20607538841392,93.20607538851392,93.2232693065483,93.2232693066483,93.23984483014839,93.23984483024839,93.25613984488753,93.25613984498753,93.27226910439067,93.27226910449068,93.28753771170689,93.28753771180689,93.30167790837802,93.30167790847803,93.31575435303532,93.31575435313532,93.32889364308907,93.32889364318908,93.34133803619189,93.34133803629189,93.35348279482963,93.35348279492963,93.3652450413843,93.3652450414843,93.37643351981436,93.37643351991436,93.38756462143193,93.38756462153194,93.39773944284183,93.39773944294183,93.40786963784203,93.40786963794203,93.41799345764083,93.41799345774083,93.42638959786441,93.42638959796442,93.43462635805336,93.43462635815337,93.44265273659663,93.44265273669663,93.45056436151498,93.45056436161498,93.457608959045,93.457608959145,93.46423916848504,93.46423916858504,93.47081837631399,93.47081837641399,93.47734658253187,93.47734658263187,93.48371540871511,93.48371540881512,93.49002685808591,93.49002685818591,93.49631280665118,93.49631280675118,93.50247125118874,93.50247125128874,93.50848306609446,93.50848306619446,93.51397211448663,93.51397211458664,93.51940378606635,93.51940378616635,93.5242425639173,93.5242425640173,93.52902396495577,93.52902396505577,93.53379261559148,93.53379261569148,93.53834450938012,93.53834450948013,93.54286452716184,93.54286452726184,93.54728891692278,93.54728891702278,93.5514264226214,93.5514264227214,93.55547467550066,93.55547467560066,93.55915954190098,93.55915954200098,93.56282528269715,93.56282528279715,93.56635714426424,93.56635714436425,93.56987625542857,93.56987625552857,93.5726877192392,93.5726877193392,93.57547368224428,93.57547368234428,93.57805563880505,93.57805563890506,93.5805674681506,93.5805674682506,93.58302192068369,93.5830219207837,93.58535524439047,93.58535524449047,93.58767581769449,93.58767581779449,93.5898306357625,93.5898306358625,93.59197270342773,93.59197270352773,93.5941083958916,93.5941083959916,93.59614846033467,93.59614846043468,93.59816302397222,93.59816302407222,93.600164837207,93.600164837307,93.60215390003901,93.60215390013902,93.60405371005163,93.60405371015163,93.60587064244626,93.60587064254626,93.60765569883395,93.60765569893395,93.60941525441612,93.60941525451612,93.61116205959551,93.61116205969552,93.61287698876798,93.61287698886798,93.61455366673214,93.61455366683214,93.61569482777999,93.61569482787999,93.616772236814,93.616772236914,93.61775401782722,93.61775401792723,93.61869117243077,93.61869117253077,93.61955819981908,93.61955819991908,93.62019571995755,93.62019572005755,93.62079498888771,93.62079498898771],[0.0,1e-10,7.387357074460425,7.387357074560425,14.454346892583077,14.454346892683077,21.41758413614381,21.417584136243807,28.108766240177303,28.1087662402773,34.62441212578843,34.62441212588843,40.780652721709835,40.78065272180984,46.483898111193696,46.4838981112937,52.10948210200623,52.109482102106234,57.33560255477633,57.33560255487633,62.02684731975697,62.02684731985697,66.32055708214824,66.32055708224824,70.3393748491069,70.3393748492069,73.50221248432489,73.50221248442489,76.30457195859428,76.30457195869428,79.08061646787613,79.08061646797613,81.78400850503881,81.78400850513881,83.90579242534461,83.90579242544462,85.98362153419801,85.98362153429801,88.0033049700399,88.00330497013991,89.95241585595775,89.95241585605775,91.89069271677067,91.89069271687067,93.33236182537131,93.33236182547131,94.31956793433976,94.31956793443976,95.30113982369105,95.30113982379105,95.94147283239856,95.94147283249856,96.54418952672292,96.54418952682292,97.11571397015285,97.11571397025286,97.49883432208622,97.49883432218623,97.75840334641052,97.75840334651052,98.01218018234336,98.01218018244336,98.23234257950898,98.23234257960898,98.44888485892993,98.44888485902993,98.63891471239546,98.63891471249546,98.8029587024866,98.80295870258661,98.87752654599431,98.87752654609432,98.94823073656363,98.94823073666363,99.0175197901964,99.0175197902964,99.04393348566789,99.0439334857679,99.06273835184332,99.06273835194332,99.08037819830821,99.08037819840821,99.0959512866425,99.0959512867425,99.10926015587832,99.10926015597832,99.11373593781717,99.11373593791717,99.11773781343308,99.11773781353308,99.1216672866941,99.1216672867941,99.1245699629221,99.1245699630221,99.12680785389152,99.12680785399152],[0.0,1e-10,6.210396688045817,6.210396688145817,12.046997913822594,12.046997913922594,17.181789469228963,17.18178946932896,21.272866252805,21.272866252904997,24.93987297462786,24.93987297472786,28.211556610248987,28.211556610348985,31.35765483112554,31.35765483122554,33.59069745784261,33.59069745794261,35.799003859907536,35.79900386000754,37.84843887553675,37.84843887563675,39.75838453997046,39.75838454007046,41.539236197815974,41.539236197915976,43.19340682115905,43.19340682125905,44.8423145567367,44.842314556836705,46.409305150775864,46.409305150875866,47.88866572882485,47.88866572892485,49.19407058412902,49.194070584229024,50.47117192902184,50.471171929121844,51.67804926467284,51.678049264772845,52.85872302778172,52.85872302788172,54.01509750983237,54.015097509932374,55.15269646043733,55.152696460537335,56.27926747645575,56.27926747655575,57.39128240140547,57.39128240150547,58.50109304374712,58.50109304384712,59.60490386360525,59.60490386370525,60.687213145715404,60.687213145815406,61.706341688455694,61.706341688555696,62.71079675229609,62.71079675239609,63.69412200652064,63.69412200662064,64.67583643883928,64.67583643893929,65.59587660895511,65.59587660905511,66.49769557905037,66.49769557915037,67.38116943974767,67.38116943984767,68.25941954195659,68.25941954205659,69.03882256874965,69.03882256884965,69.81437136175167,69.81437136185167,70.55713634211823,70.55713634221823,71.29346455640736,71.29346455650736,72.0102281321633,72.01022813226331,72.70731620310103,72.70731620320103,73.39545671268291,73.39545671278292,74.07373012289789,74.07373012299789,74.74872971693163,74.74872971703164,75.41934683193395,75.41934683203395,76.06575596752452,76.06575596762453,76.69128963380018,76.69128963390018,77.31061478811463,77.31061478821464,77.92719437148827,77.92719437158827,78.50363383813796,78.50363383823796,79.07834509505055,79.07834509515055,79.6271853782761,79.6271853783761,80.13875502509589,80.13875502519589,80.64813343239997,80.64813343249998,81.14729257684354,81.14729257694354,81.62222417723684,81.62222417733685,82.08876906924559,82.08876906934559,82.5495489144332,82.5495489145332,82.9988834460789,82.99888344617891,83.4285685590911,83.4285685591911,83.85640807453501,83.85640807463501,84.27551523964694,84.27551523974694,84.66384070679996,84.66384070689996,85.04656416578631,85.04656416588631,85.41544890378353,85.41544890388353,85.7747078396224,85.7747078397224,86.12615396314035,86.12615396324036,86.41715187513691,86.41715187523691,86.70772588663192,86.70772588673192,86.98997840520414,86.98997840530414,87.26375291374531,87.26375291384531,87.51262815897991,87.51262815907991,87.75751221795373,87.75751221805373,87.99269221621508,87.99269221631508,88.21267049033615,88.21267049043615,88.42722283803734,88.42722283813734,88.63581449253208,88.63581449263208,88.83991932325654,88.83991932335654,89.04061990687623,89.04061990697623,89.23442721618606,89.23442721628606,89.42273686206806,89.42273686216807,89.60142722733791,89.60142722743791,89.7682157708334,89.7682157709334,89.93017184861118,89.93017184871118,90.08931713998636,90.08931714008637,90.2356475931223,90.2356475932223,90.37955203108012,90.37955203118013,90.5200717865717,90.5200717866717,90.66000460290729,90.6600046030073,90.78900078630282,90.78900078640282,90.91555791142788,90.91555791152788,91.03645433447068,91.03645433457068,91.13455795362229,91.1345579537223,91.23205506897938,91.23205506907938,91.32136112233722,91.32136112243722,91.40520864154432,91.40520864164432,91.47940427240837,91.47940427250838,91.55304557184732,91.55304557194732,91.61968273069138,91.61968273079138,91.68090048466175,91.68090048476175,91.73757924249242,91.73757924259242,91.79130373990456,91.79130374000457,91.84429782414482,91.84429782424482,91.89722669292328,91.89722669302328,91.94853821824967,91.94853821834967,91.9948607607501,91.9948607608501,92.04039419616302,92.04039419626302,92.08505374438813,92.08505374448814,92.12815464307675,92.12815464317676,92.17033600375433,92.17033600385433,92.21030003873146,92.21030003883146,92.25002277650003,92.25002277660003,92.28923031212055,92.28923031222055,92.32532707021429,92.32532707031429,92.35998908814894,92.35998908824894,92.39321636592447,92.39321636602448,92.42481325715559,92.4248132572556,92.45625363127843,92.45625363137843,92.48717228170706,92.48717228180706,92.51640837322182,92.51640837332182,92.54265107504102,92.54265107514102,92.56882203985225,92.56882203995225,92.59436041468425,92.59436041478425,92.6187705620275,92.61877056212751,92.64289376133893,92.64289376143893,92.66693218055005,92.66693218065005,92.69059235008287,92.69059235018287,92.71423295497713,92.71423295507714,92.73736487926955,92.73736487936955,92.75936857607321,92.75936857617322,92.77937667974649,92.7793766798465,92.7993782618736,92.7993782619736,92.81834943970462,92.81834943980462,92.83692932476498,92.83692932486498,92.85504618004671,92.85504618014672,92.87310434141285,92.87310434151286,92.89114293814046,92.89114293824046,92.9452522067771,92.9452522068771,92.98125766322435,92.98125766332436,92.99895061800454,92.99895061810454,93.01642184021469,93.01642184031469,93.0328170073055,93.0328170074055,93.04891870481832,93.04891870491832,93.06472041120696,93.06472041130696,93.07971344586957,93.07971344596957,93.09463474352422,93.09463474362423,93.10941256716296,93.10941256726296,93.12403387369343,93.12403387379344,93.13814649962204,93.13814649972204,93.15221999627359,93.15221999637359,93.16603263107802,93.16603263117803,93.17948005929651,93.17948005939651,93.19278401349908,93.19278401359908,93.20608144615548,93.20608144625548,93.21842673306992,93.21842673316992,93.23010030072805,93.23010030082806,93.24170213137825,93.24170213147825,93.25319961728958,93.25319961738958,93.26386886683635,93.26386886693635,93.27419247443571,93.27419247453571,93.28396827215612,93.28396827225612,93.2935484234912,93.2935484235912,93.30276988978652,93.30276988988652,93.31195222680476,93.31195222690476,93.32072370641382,93.32072370651382,93.32947562138433,93.32947562148433,93.33796015296156,93.33796015306156,93.34622947351492,93.34622947361493,93.35443357860652,93.35443357870652,93.37082874569732,93.37082874579733,93.3790067646042,93.3790067647042,93.38697609403339,93.38697609413339,93.39491933727787,93.39491933737787,93.40238650765137,93.40238650775137,93.40983411338634,93.40983411348634,93.41704042191273,93.41704042201273,93.42421412270824,93.42421412280824,93.44567000963298,93.44567000973298,93.45266762868168,93.45266762878168,93.45946960134505,93.45946960144505,93.46599114752279,93.46599114762279,93.47205618546808,93.47205618556808,93.47810165877483,93.47810165887483,93.48359280065648,93.48359280075648,93.48890786079133,93.48890786089133,93.494105533095,93.494105533195,93.49889234798944,93.49889234808944,93.5035617750527,93.5035617751527,93.50817250820036,93.50817250830036,93.51254846568561,93.51254846578561,93.51689833698616,93.51689833708616,93.5211895143711,93.5211895144711,93.5253502608325,93.5253502609325,93.52949144265536,93.52949144275536,93.53339784881582,93.53339784891583,93.53726512569922,93.53726512579922,93.5411063163979,93.5411063164979,93.54484316235774,93.54484316245774,93.54771916422212,93.54771916432212,93.5504516920706,93.5504516921706,93.55314509064199,93.55314509074199,93.55583196766722,93.55583196776722,93.55844710768449,93.55844710778449,93.56101659687852,93.56101659697852,93.56354695679548,93.56354695689548,93.56601210125065,93.56601210135065,93.56818377612784,93.56818377622784,93.5703032786356,93.57030327873561,93.57240973805101,93.57240973815101,93.5744901112817,93.5744901113817,93.57863781465075,93.57863781475075,93.58062688623495,93.58062688633495,93.5825181346265,93.5825181347265,93.58439633992568,93.58439634002568,93.5862354159478,93.5862354160478,93.58802884114668,93.58802884124668,93.5898092232532,93.5898092233532,93.59156351917501,93.59156351927501,93.59327868581975,93.59327868591976,93.59486342154094,93.59486342164094,93.5963112047924,93.5963112048924,93.59770681567443,93.59770681577443,93.5990893834641,93.5990893835641,93.60008065848312,93.60008065858312,93.60103280422507,93.60103280432507,93.60195234223613,93.60195234233613,93.60461965462282,93.60461965472282,93.60510224903997,93.60510224913997],[0.0,1e-10,6.2779972003870705,6.2779972004870706,12.183180663226286,12.183180663326286,17.37346555683157,17.373465556931567,22.171083545910086,22.171083546010085,25.940080742803737,25.940080742903735,29.641127641428604,29.641127641528602,33.23652827168097,33.23652827178097,36.09195021969981,36.09195021979981,38.171088291687056,38.17108829178706,40.101472621184236,40.10147262128424,41.977385977270494,41.977385977370496,43.84596974448539,43.84596974458539,45.67885472273688,45.67885472283688,47.508483570680404,47.508483570780406,49.308543591576324,49.308543591676326,50.95853159860245,50.95853159870245,52.58372292097563,52.58372292107563,54.16734255538866,54.16734255548866,55.66417379351638,55.664173793616385,57.0799379331469,57.0799379332469,58.42837795341401,58.42837795351401,59.765902686656844,59.765902686756846,61.05670129084474,61.056701290944744,62.27622096153001,62.27622096163001,63.46731870126804,63.46731870136804,64.61892103046901,64.61892103056901,65.75754497793642,65.75754497803642,66.87707376446828,66.87707376456828,67.98593773553802,67.98593773563802,69.03261225426844,69.03261225436844,70.06267655361412,70.06267655371413,71.06875490520541,71.06875490530541,72.06264583789098,72.06264583799098,73.05482960913979,73.05482960923979,74.003062212588,74.00306221268801,74.91531259872617,74.91531259882618,75.80815803015453,75.80815803025453,76.55545311854561,76.55545311864562,77.2813394797297,77.2813394798297,77.98588961863265,77.98588961873266,78.6813634590861,78.6813634591861,79.36687775926559,79.3668777593656,80.04792311946775,80.04792311956776,80.7285400414715,80.7285400415715,81.40620403558462,81.40620403568462,82.0509507933456,82.05095079344561,82.66997807648782,82.66997807658782,83.25260129547847,83.25260129557847,83.83347121353405,83.83347121363406,84.4074399809167,84.40743998101671,84.92418917906166,84.92418917916166,85.43668695200695,85.43668695210695,85.94142010652556,85.94142010662556,86.42150817759989,86.42150817769989,86.8977007567471,86.8977007568471,87.35429064047753,87.35429064057753,87.80930518990918,87.80930519000918,88.25849957119928,88.25849957129928,88.69098486275114,88.69098486285114,89.09722996248925,89.09722996258925,89.49234885178234,89.49234885188234,89.88690088438213,89.88690088448213,90.25923345287637,90.25923345297637,90.62485602004774,90.62485602014775,90.9879606888838,90.9879606889838,91.3431425467276,91.34314254682761,91.61980156983711,91.61980156993711,91.89256510101947,91.89256510111947,92.13457336091246,92.13457336101246,92.35800717706488,92.35800717716488,92.57484963631859,92.57484963641859,92.78129752574304,92.78129752584304,92.98417949108529,92.98417949118529,93.18236181895875,93.18236181905876,93.37279930247625,93.37279930257625,93.55344202964226,93.55344202974226,93.73233145587321,93.73233145597321,93.90853819984638,93.90853819994638,94.07157541273594,94.07157541283594,94.22687437262641,94.22687437272641,94.368305117602,94.36830511770201,94.49632245128872,94.49632245138872,94.62081340903462,94.62081340913463,94.74308967086257,94.74308967096258,94.8643640464419,94.86436404654191,94.96365624676406,94.96365624686406,95.05392487949187,95.05392487959188,95.13866995513857,95.13866995523857,95.22239996182286,95.22239996192286,95.29940678447048,95.29940678457048,95.37439665190708,95.37439665200708,95.4488262540073,95.4488262541073,95.51078500885515,95.51078500895515,95.57213076751144,95.57213076761144,95.62941625031812,95.62941625041812,95.68299739054771,95.68299739064771,95.72981579859925,95.72981579869925,95.77583665246603,95.77583665256603,95.82097426450838,95.82097426460838,95.86460904717784,95.86460904727784,95.9071826213866,95.9071826214866,95.94761400460327,95.94761400470327,95.9877619594733,95.9877619595733,96.02781763534676,96.02781763544677,96.0628704713341,96.0628704714341,96.09645343473301,96.09645343483301,96.12836878483655,96.12836878493655,96.16014571644523,96.16014571654523,96.19127010372094,96.19127010382094,96.21762893995887,96.21762894005887,96.24344069357421,96.24344069367422,96.26782212274253,96.26782212284253,96.29173556557105,96.29173556567105,96.31510851713387,96.31510851723387,96.33735434666701,96.33735434676701,96.35912559850344,96.35912559860344,96.37936765553938,96.37936765563938,96.39957675579082,96.39957675589082,96.41782163168645,96.41782163178645,96.43605991622518,96.43605991632518,96.4542850180501,96.4542850181501,96.47085568929346,96.47085568939346,96.48729453339884,96.48729453349884,96.5032653911644,96.5032653912644,96.51893304651263,96.51893304661263,96.53386906124511,96.53386906134511,96.54838982049296,96.54838982059296,96.56262715139417,96.56262715149417,96.57659423666254,96.57659423676255,96.59018561458767,96.59018561468767,96.60363198266104,96.60363198276104,96.61417815369897,96.61417815379897,96.62461227166963,96.62461227176964,96.63466409094016,96.63466409104016,96.64445884729165,96.64445884739165,96.6528166878392,96.65281668793921,96.66111520617467,96.66111520627467,96.66939395043946,96.66939395053946,96.67742222314209,96.67742222324209,96.6849693267911,96.6849693268911,96.69249665636943,96.69249665646943,96.6997801057425,96.6997801058425,96.70685263169482,96.70685263179482,96.71390538357643,96.71390538367643,96.72078676017868,96.72078676027868,96.7273781170774,96.7273781171774,96.7339562912623,96.7339562913623,96.74050810001962,96.74050810011963,96.74661828786472,96.74661828796472,96.75223412394243,96.75223412404243,96.75765221931316,96.75765221941316,96.76245731849232,96.76245731859233,96.76711740781971,96.76711740791971,96.77154020829875,96.77154020839875,96.7758575470674,96.7758575471674,96.78006283276878,96.78006283286878,96.78420879625806,96.78420879635806,96.78830862024905,96.78830862034906,96.79236230474176,96.79236230484176,96.79633030159478,96.79633030169478,96.80021261080813,96.80021261090813,96.80386422253001,96.80386422263001,96.80727854540353,96.80727854550354,96.81065991149258,96.81065991159258,96.81357329124181,96.81357329134181,96.81640098335136,96.81640098345136,96.8192154927471,96.8192154928471,96.82183226143589,96.82183226153589,96.82442925605397,96.82442925615398,96.82698670253068,96.82698670263068,96.8294320959401,96.82943209604011,96.83187089799262,96.83187089809262,96.83426356054686,96.83426356064686,96.83655076139071,96.83655076149071,96.84107902358012,96.84107902368012,96.84329371949808,96.84329371959808,96.84548864134536,96.84548864144536,96.84763742369434,96.84763742379434,96.84976643197261,96.84976643207261,96.8518822575371,96.8518822576371,96.85610072595227,96.85610072605228,96.85819018608916,96.85819018618916,96.86027305486915,96.86027305496916,96.86232296686465,96.86232296696465,96.86435969614635,96.86435969624635,96.86632392050217,96.86632392060217,96.868222231289,96.868222231389,96.87008099393444,96.87008099403444,96.87191998250917,96.87191998260917,96.87368646615803,96.87368646625804,96.875380444881,96.875380444981,96.87698214460738,96.87698214470738,96.87837951226992,96.87837951236992,96.87967800957895,96.87967800967895,96.88091059331902,96.88091059341902,96.88208385484698,96.88208385494698,96.88317802009217,96.88317802019218,96.88421945448216,96.88421945458217,96.88525429751526,96.88525429761526,96.88725807001246,96.88725807011247],[0.0,1e-10,6.278657620449483,6.278657620549483,12.134443744774046,12.134443744874046,17.331511478680284,17.331511478780282,22.03163001224119,22.03163001234119,25.795589992208352,25.79558999230835,29.391554323836644,29.391554323936642,32.56434375141604,32.56434375151604,35.53842737209097,35.538427372190974,37.79756319904609,37.79756319914609,39.87911304893502,39.87911304903502,41.893992305443135,41.89399230554314,43.74656811009908,43.746568110199085,45.58388117701432,45.58388117711432,47.41094433275882,47.41094433285882,49.21375464205879,49.21375464215879,50.899113105659104,50.899113105759106,52.55670314839147,52.55670314849147,54.20844269485071,54.20844269495071,55.844233537828075,55.844233537928076,57.45557365962789,57.45557365972789,59.040227075766964,59.040227075866966,60.603872791083134,60.603872791183136,61.98257818508899,61.98257818518899,63.273512943349054,63.273512943449056,64.55299075456693,64.55299075466694,65.77466935626798,65.77466935636798,66.9856363390879,66.9856363391879,68.17727755631564,68.17727755641565,69.34680957003997,69.34680957013997,70.49832179141009,70.4983217915101,71.63989410535787,71.63989410545787,72.6584213144858,72.65842131458581,73.6653068934874,73.6653068935874,74.66018807201876,74.66018807211876,75.61137191367358,75.61137191377358,76.53642969474458,76.53642969484459,77.43057284669253,77.43057284679253,78.32280980537898,78.32280980547898,79.11109657098258,79.11109657108258,79.8626775736099,79.8626775737099,80.59487344822512,80.59487344832512,81.29989452617147,81.29989452627147,81.94665468689061,81.94665468699061,82.59069736794285,82.59069736804285,83.22419332572483,83.22419332582483,83.80748846373817,83.80748846383817,84.38845527572624,84.38845527582625,84.95182442812389,84.95182442822389,85.49264905260546,85.49264905270546,86.01052680388048,86.01052680398048,86.52767901446774,86.52767901456774,87.0338557731965,87.03385577329651,87.49031320734034,87.49031320744034,87.94547785989506,87.94547785999507,88.39206794068534,88.39206794078534,88.82498487742353,88.82498487752353,89.2239069350279,89.2239069351279,89.61931341820885,89.61931341830885,89.9834359060984,89.98343590619841,90.33888488485702,90.33888488495703,90.63319057111067,90.63319057121068,90.91003711008702,90.91003711018702,91.17015004247381,91.17015004257381,91.42689910439917,91.42689910449917,91.67432167057459,91.67432167067459,91.89788054394708,91.89788054404708,92.11646616569621,92.11646616579621,92.33347538540556,92.33347538550557,92.53175905954625,92.53175905964625,92.72218051096137,92.72218051106137,92.90844659298295,92.90844659308296,93.09301095230052,93.09301095240052,93.27378271256842,93.27378271266842,93.45406638182818,93.45406638192819,93.6331232277432,93.6331232278432,93.79836182146857,93.79836182156858,93.95395731987117,93.95395731997117,94.10920983540318,94.10920983550318,94.25074303611207,94.25074303621207,94.37872841343314,94.37872841353314,94.50261778378056,94.50261778388057,94.62491756043936,94.62491756053936,94.74143279906936,94.74143279916936,94.84375382351698,94.84375382361698,94.94309353532034,94.94309353542035,95.02994735146979,95.02994735156979,95.11025151395607,95.11025151405607,95.18529220854387,95.18529220864387,95.25977225805477,95.25977225815477,95.33003757575221,95.33003757585222,95.38734209843648,95.38734209853648,95.44094636361316,95.44094636371317,95.49146378295464,95.49146378305464,95.53832051973514,95.53832051983514,95.58493321101157,95.58493321111158,95.63103802380657,95.63103802390657,95.6770900700061,95.6770900701061,95.72177018472333,95.72177018482333,95.76610731656996,95.76610731666996,95.80974529102657,95.80974529112657,95.8502040781059,95.8502040782059,95.89037924473458,95.89037924483458,95.9305280280655,95.9305280281655,95.96553866416218,95.96553866426218,95.99914438965439,95.99914438975439,96.03110115903806,96.03110115913806,96.0628996286353,96.0628996287353,96.0941704322778,96.09417043237781,96.1253818735004,96.1253818736004,96.15185091695481,96.15185091705482,96.17768016543911,96.17768016553912,96.20222322815873,96.20222322825873,96.22662118274081,96.22662118284082,96.25055083378805,96.25055083388806,96.27437495164436,96.27437495174436,96.29777034091244,96.29777034101244,96.31956294484301,96.31956294494302,96.33979233838268,96.33979233848268,96.36001513609791,96.36001513619792,96.3788594065064,96.3788594066064,96.39674728237193,96.39674728247194,96.41332918499948,96.41332918509949,96.429014055504,96.429014055604,96.44354465673251,96.44354465683251,96.45787738322802,96.45787738332803,96.47206500158596,96.47206500168596,96.48552048343171,96.48552048353172,96.49838893690283,96.49838893700283,96.5089158726998,96.5089158727998,96.51939004190129,96.51939004200129,96.52983123198062,96.52983123208062,96.538161758241,96.538161758341,96.54647249702809,96.5464724971281,96.55475685251743,96.55475685261743,96.56302142053347,96.56302142063348,96.57127279690064,96.57127279700065,96.57945821502348,96.57945821512348,96.58749192918432,96.58749192928433,96.59541351432979,96.5954135144298,96.60270190032958,96.60270190042958,96.60989794478728,96.60989794488728,96.6170412226495,96.6170412227495,96.6241185422674,96.6241185423674,96.63100458297669,96.63100458307669,96.63759381158646,96.63759381168646,96.64409729447857,96.64409729457857,96.65046886088199,96.65046886098199,96.6565831901325,96.6565831902325,96.66214347013052,96.66214347023052,96.66639118106613,96.66639118116613,96.67044101726873,96.67044101736873,96.6743127662116,96.6743127663116,96.67812515273457,96.67812515283457,96.6817396645245,96.6817396646245,96.68532119719227,96.68532119729227,96.68884996326456,96.68884996336456,96.69232596274138,96.69232596284138,96.69527429626346,96.69527429636346,96.69799177593035,96.69799177603035,96.70062350987959,96.7006235099796,96.70322226470665,96.70322226480666,96.70562314480071,96.70562314490071,96.70785253345946,96.70785253355946,96.71004894299605,96.71004894309606,96.71438899547374,96.71438899557374,96.71651285094156,96.71651285104156,96.7186235147605,96.7186235148605,96.720727582755,96.720727582855,96.72281845910065,96.72281845920065,96.72487635632412,96.72487635642412,96.72688808277654,96.72688808287654,96.72885363845793,96.72885363855794,96.73075323589498,96.73075323599498,96.73261985420986,96.73261985430986,96.7344403017537,96.7344403018537,96.7362541534731,96.7362541535731,96.73802843024589,96.73802843034589,96.73976313207208,96.73976313217209,96.74097016794354,96.74097016804355,96.74184741259329,96.74184741269329],[0.0,1e-10,5.206711759279064,5.206711759379064,10.326775893186605,10.326775893286605,14.103725867708226,14.103725867808226,17.862747836187435,17.862747836287433,21.570945162628945,21.570945162728943,25.17415069539393,25.17415069549393,28.33323843072213,28.333238430822128,31.491170159765353,31.49117015986535,34.06716832495218,34.06716832505218,36.30242868332998,36.30242868342998,38.36185718288799,38.36185718298799,40.3793986204287,40.379398620528704,42.37905829217841,42.37905829227841,44.343060124349286,44.34306012444929,46.27781169463519,46.27781169473519,48.14686247343332,48.146862473533325,49.95093909326566,49.95093909336566,51.678237078525065,51.67823707862507,53.37856378571845,53.37856378581845,54.96581326099068,54.96581326109068,56.466071611881,56.466071611981,57.8469046049081,57.8469046050081,59.06871737337404,59.068717373474044,60.238959050029706,60.23895905012971,61.380069368304,61.380069368404,62.51502312739203,62.51502312749203,63.64959375296846,63.64959375306846,64.77664042906726,64.77664042916726,65.89263568508169,65.89263568518169,66.95663708127306,66.95663708137306,68.00622473052833,68.00622473062833,69.05472903675083,69.05472903685083,70.08696338023108,70.08696338033108,71.08305766436791,71.08305766446792,72.07753353970578,72.07753353980578,73.05468913801963,73.05468913811963,74.00506502502324,74.00506502512324,74.89986012984527,74.89986012994527,75.73323496930902,75.73323496940903,76.52349407721832,76.52349407731832,77.30948587049849,77.3094858705985,78.03695732275814,78.03695732285814,78.75813349507733,78.75813349517733,79.45515904466583,79.45515904476584,80.14242790120916,80.14242790130916,80.78944792178474,80.78944792188474,81.43499486006577,81.43499486016577,82.05557206259134,82.05557206269134,82.63946092850689,82.6394609286069,83.18586216203826,83.18586216213826,83.70400399621464,83.70400399631464,84.21790493876271,84.21790493886272,84.72473112284673,84.72473112294674,85.2257112180039,85.2257112181039,85.70669570730608,85.70669570740608,86.18386207299275,86.18386207309275,86.64100640982365,86.64100640992365,87.0908712099343,87.09087121003431,87.52432072079833,87.52432072089833,87.95673312888096,87.95673312898096,88.38870295170021,88.38870295180021,88.80649683459056,88.80649683469056,89.20930227027743,89.20930227037744,89.58280459807776,89.58280459817776,89.94693997209467,89.94693997219467,90.30291063886456,90.30291063896456,90.62383119507402,90.62383119517402,90.90810965492486,90.90810965502486,91.18535959656306,91.18535959666306,91.45873196283394,91.45873196293394,91.71964588422789,91.71964588432789,91.96769180423252,91.96769180433252,92.19158710150647,92.19158710160647,92.41440566149784,92.41440566159784,92.63172163157274,92.63172163167275,92.83844858407726,92.83844858417726,93.04174054647785,93.04174054657786,93.23437743380609,93.23437743390609,93.42511847082696,93.42511847092696,93.60611602630304,93.60611602640304,93.78552820173115,93.78552820183116,93.94889500992382,93.94889501002382,94.10452648463244,94.10452648473245,94.25274630761685,94.25274630771685,94.39451231265598,94.39451231275598,94.52419640057956,94.52419640067956,94.65249328096118,94.65249328106118,94.75194945597023,94.75194945607024,94.8368795862898,94.8368795863898,94.91730459497305,94.91730459507305,94.99245160924663,94.99245160934663,95.06686538524801,95.06686538534801,95.13850474616547,95.13850474626547,95.20672893422959,95.20672893432959,95.27404152876613,95.27404152886614,95.33143228650249,95.33143228660249,95.38679507892749,95.3867950790275,95.43841241098917,95.43841241108917,95.48847739175389,95.48847739185389,95.53814602750661,95.53814602760662,95.5850666711762,95.5850666712762,95.63174290208838,95.63174290218838,95.67796994198699,95.67796994208699,95.72409128988238,95.72409128998238,95.7696511490108,95.7696511491108,95.81488732637942,95.81488732647942,95.85929117922285,95.85929117932285,95.9029485822937,95.9029485823937,95.94570760333738,95.94570760343738,95.98619424631231,95.98619424641231,96.02647611103103,96.02647611113103,96.06581335347119,96.06581335357119,96.10092291578343,96.10092291588343,96.1345792130517,96.1345792131517,96.16658407277001,96.16658407287001,96.19843039448351,96.19843039458351,96.22974825618104,96.22974825628104,96.25773681977783,96.25773681987783,96.28423248382946,96.28423248392946,96.31010060161209,96.3101006017121,96.33527511562374,96.33527511572375,96.35970978561302,96.35970978571302,96.3836754473381,96.38367544743811,96.40761468606239,96.40761468616239,96.43104528202127,96.43104528212127,96.45131172363443,96.45131172373443,96.4715649537472,96.4715649538472,96.4898430645502,96.4898430646502,96.52472803135565,96.52472803145565,96.5407337640899,96.5407337641899,96.55634975756236,96.55634975766236,96.57164867502523,96.57164867512523,96.5866173049781,96.58661730507811,96.60136133942419,96.60136133952419,96.61608555661967,96.61608555671967,96.63063802430996,96.63063802440996,96.64490644474165,96.64490644484165,96.65890402941514,96.65890402951514,96.67252508632724,96.67252508642724,96.68600081673496,96.68600081683496,96.69713150582172,96.69713150592172,96.70766107164029,96.70766107174029,96.71811797420666,96.71811797430667,96.72801338800605,96.72801338810605,96.73669334376866,96.73669334386867,96.74506943502207,96.74506943512208,96.7534323147751,96.7534323148751,96.76174895427671,96.76174895437671,96.77005898802814,96.77005898812814,96.77810479177155,96.77810479187156,96.78566837575039,96.78566837585039,96.79321214247864,96.79321214257864,96.80051149644947,96.80051149654948,96.80771176416732,96.80771176426732,96.81479973413177,96.81479973423177,96.82143190733242,96.82143190743243,96.82802444603188,96.82802444613188,96.83414797646715,96.83414797656715,96.83974304688643,96.83974304698643,96.84530508855471,96.84530508865471,96.85063592896599,96.85063592906599,96.85577520262147,96.85577520272147,96.86071630377097,96.86071630387097,96.86557153016787,96.86557153026787,96.87042015081457,96.87042015091457,96.87526216571106,96.87526216581107,96.87993243110236,96.87993243120236,96.88414689972987,96.88414689982987,96.88802447509718,96.88802447519718,96.8916510319569,96.8916510320569,96.89511244506163,96.89511244516163,96.89853422366515,96.89853422376515,96.90192297351769,96.90192297361769,96.90483610935581,96.90483610945581,96.90770300494255,96.90770300504255,96.91036512227309,96.9103651223731,96.91300081660285,96.91300081670285,96.91560348218158,96.91560348228158,96.91816651325912,96.91816651335913,96.92061724658328,96.92061724668328,96.92294247065362,96.92294247075363,96.92519503147177,96.92519503157177,96.92740795778873,96.92740795788873,96.92960767260529,96.92960767270529,96.93178096442104,96.93178096452104,96.93388819873479,96.9338881988348,96.93598222154814,96.93598222164815,96.9378846776057,96.9378846777057,96.93970786466086,96.93970786476086,96.94152444596583,96.94152444606583,96.94308340301299,96.943083403113,96.94423940929796,96.94423940939797,96.94536899258213,96.94536899268213,96.94643912411452,96.94643912421452,96.94731768889109,96.94731768899109,96.94782633165649,96.94782633175649],[0.0,1e-10,6.177384034957883,6.177384035057883,11.294703728128201,11.294703728228201,14.987999436916875,14.987999437016875,18.634670097605618,18.634670097705616,21.764519234033013,21.76451923413301,24.65792018666634,24.657920186766336,27.425763207709853,27.42576320780985,30.17992504815834,30.179925048258337,32.87294343396465,32.87294343406465,35.48299856951185,35.48299856961185,38.001380708899326,38.00138070899933,40.11799174547137,40.11799174557137,42.13959386234201,42.13959386244201,44.05848328277202,44.058483282872025,45.96979223881163,45.96979223891163,47.81696579321934,47.81696579331934,49.59021034944967,49.59021034954967,51.25123417813325,51.251234178233254,52.84087313709925,52.84087313719925,54.40120918791225,54.40120918801225,55.96078589426148,55.96078589436148,57.49640106419855,57.49640106429855,58.9848005847861,58.9848005848861,60.18592776349626,60.18592776359626,61.33691873722703,61.33691873732703,62.4730213673691,62.4730213674691,63.58118401531877,63.58118401541877,64.62831354072783,64.62831354082783,65.63752127381397,65.63752127391398,66.64023239315455,66.64023239325455,67.63097572453631,67.63097572463631,68.62153084233303,68.62153084243303,69.59848266101818,69.59848266111818,70.56806818939245,70.56806818949245,71.46536023074206,71.46536023084207,72.34986672855649,72.3498667286565,73.22965490649776,73.22965490659776,74.04362025066546,74.04362025076546,74.73717433127551,74.73717433137551,75.41850101898186,75.41850101908186,76.06755232191637,76.06755232201637,76.71387128280604,76.71387128290604,77.33264615904424,77.33264615914425,77.93112641871669,77.93112641881669,78.52799063760727,78.52799063770728,79.10226922629354,79.10226922639355,79.67439958406096,79.67439958416097,80.20542149880708,80.20542149890709,80.71448732534023,80.71448732544023,81.22285870864583,81.22285870874583,81.70812525185735,81.70812525195736,82.18074254412976,82.18074254422976,82.65167889438408,82.65167889448408,83.12135616065574,83.12135616075574,83.58632808730151,83.58632808740151,84.0493270163662,84.04932701646621,84.50781530951367,84.50781530961368,84.95725637033216,84.95725637043216,85.39973352850433,85.39973352860433,85.82584908502562,85.82584908512563,86.22621832113872,86.22621832123872,86.61639806316539,86.6163980632654,86.98333667250193,86.98333667260194,87.29077382847265,87.29077382857265,87.58608094339398,87.58608094349398,87.85866613551495,87.85866613561495,88.10604368748835,88.10604368758835,88.33937661194389,88.33937661204389,88.56716048070268,88.56716048080268,88.78696798753013,88.78696798763013,89.00049954481518,89.00049954491519,89.20871569085386,89.20871569095387,89.40386721804161,89.40386721814161,89.59125006725391,89.59125006735391,89.77412228054897,89.77412228064897,89.95200358877875,89.95200358887875,90.1287426352511,90.1287426353511,90.30496247183368,90.30496247193368,90.46337989932131,90.46337989942131,90.61652734642793,90.61652734652793,90.74397390399173,90.74397390409173,90.8686881195107,90.8686881196107,90.97326997155444,90.97326997165445,91.07104368391877,91.07104368401878,91.16806454194298,91.16806454204298,91.24704285629821,91.24704285639821,91.32122496929685,91.32122496939685,91.39486191191124,91.39486191201124,91.46830415081698,91.46830415091698,91.53929961311724,91.53929961321724,91.60989917787657,91.60989917797657,91.67903846482096,91.67903846492096,91.74342698127411,91.74342698137411,91.80604369397847,91.80604369407847,91.86008695337826,91.86008695347826,91.91362398313554,91.91362398323554,91.96636921781095,91.96636921791095,92.017232316636,92.017232316736,92.06641447344296,92.06641447354296,92.11228017707913,92.11228017717913,92.15766561156727,92.15766561166727,92.202577267031,92.202577267131,92.24654785456957,92.24654785466957,92.29011605444357,92.29011605454357,92.33308067282073,92.33308067292073,92.37565588378058,92.37565588388058,92.41758857250186,92.41758857260186,92.45710693523577,92.45710693533577,92.4961255584508,92.4961255585508,92.53204839269817,92.53204839279817,92.56654339974871,92.56654339984871,92.59798804869696,92.59798804879696,92.62919905319481,92.62919905329481,92.65991031817377,92.65991031827377,92.68595518426856,92.68595518436857,92.71137050837203,92.71137050847203,92.73602648801173,92.73602648811173,92.76031253060499,92.760312530705,92.78423512627542,92.78423512637542,92.80778129477576,92.80778129487577,92.83130150278163,92.83130150288163,92.85432197126862,92.85432197136862,92.87422069029344,92.87422069039344,92.89310045990963,92.89310046000963,92.91164274309747,92.91164274319748,92.93013310529635,92.93013310539635,92.94816266871807,92.94816266881807,92.9660494494201,92.9660494495201,92.9836571548064,92.9836571549064,93.0012388996982,93.0012388997982,93.01862594088134,93.01862594098134,93.03550026229834,93.03550026239834,93.05193974543269,93.05193974553269,93.06825591621822,93.06825591631822,93.08428003144076,93.08428003154076,93.10000560097669,93.10000560107669,93.11543911494962,93.11543911504963,93.13078176719186,93.13078176729186,93.14563117003884,93.14563117013884,93.16018851732282,93.16018851742282,93.17448625966193,93.17448625976193,93.18876453163017,93.18876453173017,93.20278319865353,93.20278319875354,93.21678888542965,93.21678888552965,93.23063231911523,93.23063231921523,93.24387217130396,93.24387217140396,93.25710553336907,93.25710553346907,93.26939133738537,93.26939133748537,93.28153435868201,93.28153435878201,93.29351512688808,93.29351512698808,93.30513244817134,93.30513244827134,93.31667837809474,93.31667837819474,93.3281204660402,93.3281204661402,93.33851764408253,93.33851764418253,93.34879150977604,93.34879150987604,93.35868894829947,93.35868894839948,93.3682229399001,93.3682229400001,93.37761414878102,93.37761414888102,93.38607077986039,93.38607077996039,93.39451443069252,93.39451443079253,93.40284774942307,93.40284774952308,93.41101881506309,93.41101881516309,93.4191314695905,93.4191314696905,93.42708836115098,93.42708836125098,93.43499333172248,93.43499333182248,93.44228823067353,93.44228823077353,93.44946630739939,93.44946630749939,93.45663789400162,93.45663789410162,93.46360179664794,93.46360179674794,93.47008543014624,93.47008543024624,93.4765236327792,93.4765236328792,93.48255944774759,93.48255944784759,93.48825777628764,93.48825777638764,93.49357318753401,93.49357318763401,93.49881720742052,93.49881720752052,93.50404175693618,93.50404175703618,93.50868868544947,93.50868868554947,93.51327720285018,93.51327720295018,93.5177229375312,93.5177229376312,93.52187661664924,93.52187661674924,93.52601731552002,93.52601731562002,93.53423381202538,93.53423381212538,93.53830311953632,93.53830311963632,93.54215176284413,93.54215176294413,93.54597444565745,93.54597444575745,93.54963487538022,93.54963487548022,93.55313305201244,93.55313305211244,93.55653387679033,93.55653387689033,93.55988278057924,93.55988278067925,93.56306294115399,93.56306294125399,93.56592508567125,93.56592508577125,93.5686898783342,93.5686898784342,93.57142871050264,93.57142871060265,93.57398581920968,93.57398581930968,93.576503987175,93.576503987275,93.57898321439858,93.57898321449858,93.58126124778987,93.58126124788987,93.58346788982132,93.58346788992132,93.58564208123467,93.58564208133467,93.58780329240078,93.58780329250078,93.58988662208341,93.58988662218341,93.59402083083057,93.59402083093057,93.59598733828803,93.59598733838803,93.59786947413838,93.59786947423838,93.59970617912339,93.59970617922339,93.60333415822805,93.60333415832805,93.60687776572561,93.60687776582562,93.6156329424916,93.6156329425916,93.62086398213087,93.62086398223087,93.6224994932836,93.6224994933836,93.62398573159302,93.62398573169303,93.62537461804811,93.62537461814811,93.62658827116542,93.62658827126542,93.62762669094492,93.62762669104492,93.62845093664491,93.62845093674491],[0.0,1e-10,6.270092792873457,6.270092792973457,12.102658000147759,12.102658000247759,17.166698036428354,17.166698036528352,20.85944638477915,20.85944638487915,24.322648338568168,24.322648338668166,27.496766041817416,27.496766041917414,30.395246497578718,30.395246497678716,33.04090487541358,33.04090487551358,35.11592744223463,35.11592744233463,37.124050510995986,37.12405051109599,39.0624781050357,39.0624781051357,40.99970178441862,40.99970178451862,42.832388830487695,42.832388830587696,44.65383933976005,44.65383933986005,46.34418814804572,46.34418814814572,48.02984629430792,48.029846294407925,49.676992329090005,49.67699232919001,51.2599361609451,51.259936161045104,52.84093925605837,52.84093925615837,54.40142975062585,54.40142975072585,55.88839592713698,55.888395927236985,57.26357566731904,57.26357566741904,58.607848352541474,58.607848352641476,59.884721553289374,59.884721553389376,61.114122359701675,61.11412235980168,62.33157612230668,62.33157612240668,63.537128892484695,63.5371288925847,64.70394587304963,64.70394587314964,65.811955240953,65.811955241053,66.89305744521548,66.89305744531548,67.95456149776983,67.95456149786983,68.95865263660929,68.95865263670929,69.95039542675583,69.95039542685583,70.94069746657541,70.94069746667542,71.91557229398964,71.91557229408964,72.87302654210777,72.87302654220777,73.81997449674559,73.81997449684559,74.73657459175615,74.73657459185615,75.62840562292617,75.62840562302617,76.50516469519611,76.50516469529612,77.30839944940969,77.3083994495097,78.09464124428725,78.09464124438725,78.79563535543409,78.79563535553409,79.48960992046729,79.48960992056729,80.13412530304275,80.13412530314275,80.76530552163287,80.76530552173287,81.35071724695901,81.35071724705901,81.9324053892483,81.9324053893483,82.51367249034615,82.51367249044615,83.07406515862431,83.07406515872431,83.62760932877285,83.62760932887285,84.17181164748615,84.17181164758615,84.71006675937029,84.7100667594703,85.23500644357492,85.23500644367492,85.75641990780119,85.75641990790119,86.2722151036291,86.2722151037291,86.75535329204541,86.75535329214541,87.23442580145684,87.23442580155684,87.71100495756266,87.71100495766267,88.14291427476346,88.14291427486346,88.54083767329216,88.54083767339216,88.93546510874408,88.93546510884408,89.32994123251783,89.32994123261783,89.72420025692723,89.72420025702723,90.08850614782638,90.08850614792638,90.42029976422351,90.42029976432352,90.69648962827523,90.69648962837523,90.95631151600946,90.95631151610947,91.20919280285345,91.20919280295345,91.43216700756749,91.43216700766749,91.65020713581927,91.65020713591927,91.86663546576004,91.86663546586004,92.0690970699283,92.0690970700283,92.25916427402335,92.25916427412335,92.44919200550669,92.44919200560669,92.62947658066922,92.62947658076922,92.80862960762974,92.80862960772974,92.98730896324987,92.98730896334988,93.16167264665769,93.1616726467577,93.32491821110386,93.32491821120387,93.48764405282935,93.48764405292935,93.64296877986145,93.64296877996145,93.77725460485843,93.77725460495843,93.90631688757404,93.90631688767404,94.03397131380575,94.03397131390575,94.15824425296866,94.15824425306866,94.27447793688238,94.27447793698238,94.38437626661855,94.38437626671855,94.48347883705529,94.48347883715529,94.58200247585378,94.58200247595379,94.67513810315549,94.67513810325549,94.75475436095059,94.75475436105059,94.82960101149884,94.82960101159884,94.90388846671469,94.90388846681469,94.97397208878469,94.9739720888847,95.0412005252752,95.0412005253752,95.09540299990533,95.09540300000533,95.14937521763389,95.14937521773389,95.20284087017899,95.20284087027899,95.25424736814716,95.25424736824716,95.3041078554905,95.3041078555905,95.35084342774107,95.35084342784107,95.39734874309008,95.39734874319008,95.44336065079284,95.44336065089284,95.48792522940002,95.48792522950002,95.53123984197008,95.53123984207008,95.57271239932757,95.57271239942757,95.6138362819484,95.6138362820484,95.6541904486411,95.6541904487411,95.69424857074607,95.69424857084607,95.72782002699532,95.72782002709532,95.76066781869677,95.76066781879678,95.79254195264299,95.792541952743,95.82425819614244,95.82425819624244,95.85538892923502,95.85538892933502,95.88645387464143,95.88645387474143,95.91599912449747,95.91599912459748,95.9423997229549,95.9423997230549,95.96816218085654,95.96816218095654,95.99264177887798,95.99264177897798,96.01697664398986,96.01697664408987,96.0410483583571,96.0410483584571,96.06491613089723,96.06491613099723,96.0886786431395,96.0886786432395,96.1120135354217,96.1120135355217,96.13374320816152,96.13374320826152,96.1539729216568,96.1539729217568,96.17051194595831,96.17051194605831,96.1869193948875,96.1869193949875,96.20247818266517,96.20247818276518,96.21734619973813,96.21734619983813,96.23155633994945,96.23155634004945,96.24503623684436,96.24503623694436,96.25845692482172,96.25845692492172,96.2717657737326,96.2717657738326,96.2846009513031,96.2846009514031,96.29507435094034,96.29507435104034,96.30548854166003,96.30548854176003,96.31552774256858,96.31552774266858,96.32530379273247,96.32530379283247,96.33359304118908,96.33359304128908,96.34187571087706,96.34187571097706,96.35011232918474,96.35011232928474,96.35812526935946,96.35812526945946,96.3660263704677,96.3660263705677,96.37355906053342,96.37355906063343,96.3808285998545,96.3808285999545,96.38806524533248,96.38806524543249,96.39524268189292,96.39524268199293,96.40230170061827,96.40230170071827,96.40928835288884,96.40928835298884,96.41615658732431,96.41615658742431,96.42276167101512,96.42276167111513,96.42845888463688,96.42845888473688,96.43352453647148,96.43352453657148,96.43802441420506,96.43802441430506,96.442261141194,96.442261141294,96.44643865926538,96.44643865936538,96.45057670472507,96.45057670482507,96.45457659604381,96.45457659614381,96.45845149075883,96.45845149085883,96.46231322793663,96.46231322803663,96.4660762835852,96.4660762836852,96.46959592479494,96.46959592489495,96.47297083309513,96.47297083319513,96.4758983851294,96.4758983852294,96.47879962208921,96.47879962218921,96.48168770151179,96.48168770161179,96.48456262339714,96.48456262349714,96.4873651788277,96.4873651789277,96.49000326504286,96.49000326514286,96.49525970116733,96.49525970126733,96.49785173600218,96.49785173610218,96.50040429822532,96.50040429832532,96.50291738783676,96.50291738793676,96.50510811778601,96.50510811788601,96.50721990251186,96.50721990261187,96.5093251084691,96.5093251085691,96.5114171568891,96.5114171569891,96.51350262654047,96.51350262664047,96.51558151742324,96.51558151752324,96.51758146308262,96.51758146318262,96.51955509366752,96.51955509376752,96.52144977902903,96.52144977912903,96.52331157054746,96.52331157064746,96.52697594466677,96.52697594476678,96.53053505848821,96.53053505858821,96.53198238758381,96.53198238768381,96.53325208992676,96.53325209002676,96.53412706615275,96.53412706625275,96.53583754599299,96.53583754609299,96.53649542285463,96.53649542295463,96.53714672094765,96.53714672104765,96.53771249504865,96.53771249514865,96.53827169038104,96.53827169048104,96.53872562541557,96.53872562551557],[0.0,1e-10,5.843557620983446,5.843557621083446,10.98723529648483,10.98723529658483,16.047206790466678,16.047206790566676,20.778027890450954,20.778027890550952,24.135308427015794,24.135308427115792,27.25379089407972,27.25379089417972,30.03593547984402,30.03593547994402,32.811192600462256,32.81119260056226,35.35111495081495,35.351114950914955,37.40815164111141,37.40815164121141,39.40017483465029,39.40017483475029,41.374542376531586,41.37454237663159,43.28483562144796,43.28483562154796,45.065649739394885,45.06564973949489,46.77121699617687,46.77121699627687,48.45003010331002,48.45003010341002,50.08202018744732,50.08202018754732,51.678085879515926,51.67808587961593,53.27412548270141,53.27412548280141,54.84130425892538,54.841304259025385,56.32299628735626,56.32299628745626,57.52977018759407,57.52977018769407,58.669149980488775,58.66914998058878,59.795804920637295,59.7958049207373,60.908632752727414,60.908632752827415,62.016647185880224,62.016647185980226,63.06716824283741,63.06716824293741,64.10348390293083,64.10348390303083,65.13916038538758,65.13916038548759,66.13457519895546,66.13457519905546,67.11806087071255,67.11806087081256,68.09983119840393,68.09983119850394,68.98399649208878,68.98399649218878,69.76351275332225,69.76351275342225,70.50675894478556,70.50675894488556,71.24338508215487,71.24338508225487,71.97331942100159,71.97331942110159,72.69159202908962,72.69159202918962,73.38873916406392,73.38873916416392,74.07702260099512,74.07702260109512,74.75875120604015,74.75875120614015,75.4290398358331,75.4290398359331,76.09839578805418,76.09839578815418,76.73633420276711,76.73633420286711,77.36224564235755,77.36224564245755,77.98162833894494,77.98162833904495,78.55847311159043,78.55847311169043,79.09793251471201,79.09793251481202,79.60924201293734,79.60924201303735,80.11685993419992,80.11685993429992,80.61745342368,80.61745342378,81.10376324964693,81.10376324974693,81.57537198997065,81.57537199007065,82.04038024286272,82.04038024296273,82.49178966542377,82.49178966552377,82.94311429911465,82.94311429921466,83.3728373375747,83.3728373376747,83.8008058986443,83.8008058987443,84.2217500279314,84.22175002803141,84.62090341758498,84.62090341768499,85.01230188672847,85.01230188682847,85.40066752320818,85.40066752330819,85.76959041953599,85.76959041963599,86.12781687378087,86.12781687388087,86.42857604071327,86.42857604081327,86.71918010988767,86.71918010998768,87.00113571430481,87.00113571440481,87.27493854274412,87.27493854284413,87.54484108315565,87.54484108325565,87.8024492373926,87.8024492374926,88.05669844792669,88.0566984480267,88.30559943741932,88.30559943751932,88.54471057351779,88.54471057361779,88.76577472471173,88.76577472481173,88.98034274400655,88.98034274410655,89.18907337570124,89.18907337580124,89.39768660742186,89.39768660752186,89.59945802954188,89.59945802964188,89.7932853867491,89.7932853868491,89.98172538959018,89.98172538969018,90.16043423902413,90.16043423912413,90.33894089961383,90.33894089971383,90.50574669612014,90.50574669622014,90.6691609378197,90.6691609379197,90.83048806886892,90.83048806896892,90.970461449077,90.97046144917701,91.09853829857826,91.09853829867826,91.22366710428594,91.22366710438594,91.34466082201766,91.34466082211766,91.46166946285142,91.46166946295142,91.55978323007896,91.55978323017897,91.65734260854002,91.65734260864002,91.74119880113756,91.74119880123756,91.82060683916158,91.82060683926159,91.89481014500136,91.89481014510136,91.96845906207464,91.96845906217465,92.03510311402779,92.03510311412779,92.08883316883205,92.08883316893206,92.14183925712949,92.14183925722949,92.19481273432301,92.19481273442301,92.2457773675156,92.2457773676156,92.29517666772044,92.29517666782044,92.3414844352745,92.3414844353745,92.38661820308774,92.38661820318774,92.43128237100466,92.43128237110466,92.47512473910301,92.47512473920301,92.51823009625298,92.51823009635298,92.56036364250639,92.56036364260639,92.60033181146008,92.60033181156008,92.6398499471798,92.6398499472798,92.67906153852275,92.67906153862275,92.71389019750002,92.71389019760002,92.74855580095773,92.74855580105773,92.78178651584335,92.78178651594335,92.81338667553341,92.81338667563341,92.84483030192469,92.84483030202469,92.87575215065338,92.87575215075339,92.90462602205646,92.90462602215646,92.93219544930307,92.93219544940307,92.95827781021133,92.95827781031133,92.98381882679466,92.98381882689466,93.00827063250736,93.00827063260736,93.03231153831078,93.03231153841078,93.05597415530882,93.05597415540882,93.0791084724235,93.0791084725235,93.10163622300543,93.10163622310543,93.12166596302775,93.12166596312775,93.14166309194616,93.14166309204616,93.16063623220175,93.16063623230175,93.17927673919746,93.17927673929746,93.19785854620612,93.19785854630612,93.21597727553925,93.21597727563925,93.23395251601515,93.23395251611515,93.25164730099742,93.25164730109742,93.2691203304731,93.2691203305731,93.28551719351971,93.28551719361971,93.30162055663111,93.30162055673111,93.31742389758652,93.31742389768652,93.33284242751579,93.33284242761579,93.3478370130942,93.34783701319421,93.36275985424402,93.36275985434402,93.37753920653662,93.37753920663663,93.39216202553045,93.39216202563045,93.40653047791378,93.40653047801378,93.42079457476457,93.42079457486457,93.43488257165426,93.43488257175426,93.44895752410238,93.44895752420238,93.4624063433554,93.4624063434554,93.4757116737512,93.47571167385121,93.48901048192624,93.48901048202625,93.50135704586707,93.50135704596707,93.51341010987268,93.51341010997268,93.52508488507293,93.52508488517293,93.53668791584457,93.53668791594457,93.54818659108369,93.5481865911837,93.55861562211453,93.55861562221453,93.56894029761284,93.56894029771284,93.57871710656546,93.57871710666547,93.5882982488946,93.5882982489946,93.59707063584673,93.59707063594674,93.60582345613653,93.60582345623654,93.61430886537426,93.61430886547426,93.6225659968846,93.6225659969846,93.63081008395338,93.63081008405338,93.63902155991823,93.63902156001824,93.65541190074406,93.65541190084406,93.66357772116345,93.66357772126345,93.67155439718016,93.67155439728016,93.67949846209295,93.67949846219295,93.68716859373289,93.6871685938329,93.69463653652858,93.69463653662858,93.70184359049297,93.70184359059297,93.70884193339234,93.70884193349234,93.71565113188902,93.71565113198902,93.72221248599598,93.72221248609598,93.72876079566137,93.72876079576137,93.73482646098887,93.73482646108887,93.7404420930824,93.7404420931824,93.74593380298106,93.74593380308106,93.7508124241262,93.7508124242262,93.75560625640115,93.75560625650115,93.76027616648125,93.76027616658125,93.76488737657431,93.76488737667431,93.76928987560234,93.76928987570234,93.77352279689002,93.77352279699002,93.7776839737491,93.77768397384911,93.78571282753207,93.78571282763207,93.78955441557282,93.78955441567282,93.7932003369901,93.7932003370901,93.79661798068001,93.79661798078001,93.7999638799413,93.7999638800413,93.80293801261801,93.80293801271802,93.805814311983,93.805814312083,93.81146907740121,93.81146907750121,93.81421493235055,93.81421493245055,93.81685643176736,93.81685643186736,93.81942618675558,93.81942618685558,93.82196985286066,93.82196985296066,93.82450047452417,93.82450047462417,93.82701152952535,93.82701152962535,93.82949649564338,93.82949649574338,93.83193580621595,93.83193580631595,93.83435555012618,93.83435555022618,93.83659267185449,93.83659267195449,93.83878413803733,93.83878413813733,93.84095603755782,93.84095603765782,93.84310837041596,93.84310837051596,93.8451889588455,93.8451889589455,93.84725650283347,93.84725650293348,93.84931752460068,93.84931752470068,93.85336782370646,93.85336782380647,93.85531796772035,93.85531796782035,93.85720288952643,93.85720288962644,93.8609662109178,93.8609662110178,93.86279243273682,93.86279243283683,93.86458604345195,93.86458604355195,93.86637313194629,93.86637313204629,93.86815369821984,93.86815369831984,93.8716626530007,93.8716626531007,93.87318233044297,93.87318233054297,93.87437589684612,93.87437589694612,93.87554989658693,93.87554989668693,93.87671737410696,93.87671737420696,93.87781962941915,93.87781962951915,93.87882405141963,93.87882405151963,93.87971107344602,93.87971107354602,93.88032416219954,93.88032416229954],[0.0,1e-10,0.05767744775168155,0.05767744785168155,0.10226154587201414,0.10226154597201413,0.14313738762822342,0.14313738772822343,0.17282959907993387,0.17282959917993387,0.1986631310698581,0.19866313116985812,0.21512465006191872,0.21512465016191873,0.23155346838057436,0.23155346848057437,0.24794958602582504,0.24794958612582504,0.2634300848157366,0.2634300849157366,0.2786162775450035,0.2786162776450035,0.2918535101393343,0.2918535102393343,0.3013040047533699,0.3013040048533699,0.31055175519229467,0.3105517552922947,0.31962292199483266,0.3196229220948327,0.3277065284605406,0.3277065285605406,0.33575089411816256,0.33575089421816257,0.3434682530417349,0.3434682531417349,0.3510744296757303,0.35107442977573033,0.35856288388546786,0.3585628839854679,0.3658616741894566,0.3658616742894566,0.37310160328131636,0.37310160338131637,0.3802238099489183,0.3802238100489183,0.3873002356737532,0.3873002357737532,0.3943701212639072,0.3943701213639072,0.4012765034870363,0.4012765035870363,0.40809132382463154,0.40809132392463154,0.41471648025647795,0.41471648035647796,0.4211781333212995,0.4211781334212995,0.42734548032547637,0.4273454804254764,0.4334997470602912,0.4334997471602912,0.4395559117748912,0.4395559118748912,0.44551397446927626,0.44551397456927627,0.45112541002556866,0.45112541012556867,0.4563313572316395,0.4563313573316395,0.46136726093600444,0.46136726103600445,0.4663377632935595,0.46633776339355953,0.4712886452470716,0.4712886453470716,0.47623298706590267,0.4762329871659027,0.48107268672983783,0.48107268682983784,0.4857881238348341,0.4857881239348341,0.4903269773034436,0.4903269774034436,0.4948396702333291,0.4948396703333291,0.4992411808736377,0.49924118097363773,0.5035838303018174,0.5035838304018174,0.5078741586525491,0.5078741587525492,0.5121383264645569,0.5121383265645569,0.5161474290240059,0.5161474291240059,0.5200191887551541,0.5200191888551541,0.5237797661967253,0.5237797662967253,0.5312682204064628,0.5312682205064628,0.5347998931341993,0.5347998932341993,0.5382988651885309,0.5382988652885309,0.5413531080845548,0.5413531081845548,0.5443288693644068,0.5443288694644068,0.547147667411915,0.547147667511915,0.5499403049206992,0.5499403050206992,0.5555124996689057,0.5555124997689057,0.5610454536090261,0.5610454537090261,0.5637923101750434,0.5637923102750434,0.5664737653942508,0.5664737654942508,0.5689917172464333,0.5689917173464333,0.5714573480211678,0.5714573481211678,0.5739164386612213,0.5739164387612213,0.5763624490319129,0.5763624491319129,0.5787757587291995,0.5787757588291995,0.5810713460022282,0.5810713461022282,0.583314612197809,0.583314612297809,0.5855513382587088,0.5855513383587088,0.5900051699764653,0.5900051700764654,0.5922091953639601,0.5922091954639601,0.594367439808688,0.594367439908688,0.5965126039840538,0.5965126040840538,0.5986381477553767,0.5986381478553767,0.6007113704492517,0.6007113705492517,0.6027257319309977,0.6027257320309977,0.6046681519312527,0.6046681520312527,0.6065059297766119,0.6065059298766119,0.6083240872179281,0.6083240873179281,0.6101291643898823,0.6101291644898823,0.6119211612924745,0.6119211613924745,0.6135888956361278,0.6135888957361278,0.6152108490370142,0.6152108491370142,0.6167543208217287,0.6167543209217287,0.6182847123370812,0.6182847124370812,0.6197954834483906,0.6197954835483906,0.6210577294418224,0.6210577295418224,0.6223134353005731,0.6223134354005732,0.6233140759067651,0.6233140760067651,0.6252368755029772,0.6252368756029772,0.6261524943583163,0.6261524944583163,0.6270354125402504,0.6270354126402504,0.6279117905875036,0.6279117906875036,0.6287489278266707,0.6287489279266707,0.6295664446617949,0.6295664447617949,0.6303774213622381,0.6303774214622381,0.6311818579280003,0.6311818580280003,0.6319797543590815,0.6319797544590815,0.633529766278477,0.633529766378477,0.6342688014974293,0.6342688015974293,0.6350012965817005,0.6350012966817005,0.6356814705885239,0.6356814706885239,0.6376958320702698,0.6376958321702698,0.6389515379290206,0.6389515380290206,0.639546690184991,0.639546690284991,0.6413125265488592,0.6413125266488592,0.6424439698486711,0.6424439699486711,0.6429933411618746,0.6429933412618746,0.643523092071035,0.643523092171035,0.6440463028455145,0.6440463029455145,0.644549893215951,0.644549893315951,0.6450469434517065,0.6450469435517066,0.645537453552781,0.645537453652781,0.6460083432498125,0.6460083433498125,0.6464726928121631,0.6464726929121631],[0.0,1e-10,0.058045120125886626,0.05804512022588663,0.10291342536436822,0.10291342546436821,0.14404983547739594,0.14404983557739595,0.17393132378349926,0.17393132388349927,0.19993611680055087,0.19993611690055088,0.2165025718812694,0.2165025719812694,0.2330361178338975,0.2330361179338975,0.24953675465843517,0.24953675475843518,0.265115935896441,0.265115935996441,0.2803989349816331,0.2803989350816331,0.293720550032636,0.293720550132636,0.303231288050768,0.303231288150768,0.3125379894747394,0.3125379895747394,0.3216669816070225,0.3216669817070225,0.32980211807097487,0.3298021181709749,0.3378977635812187,0.3378977636812187,0.34566431781055834,0.34566431791055835,0.3533189810043905,0.35331898110439053,0.3608551713370972,0.3608551714370972,0.36820048872687944,0.36820048882687945,0.37536151499935533,0.37536151509935534,0.3824830503181227,0.3824830504181227,0.38959800381127196,0.389598003911272,0.3964562661053159,0.39645626620531593,0.40313023728205355,0.40313023738205356,0.40963308099272094,0.40963308109272095,0.4158397425505746,0.4158397426505746,0.42203324045719204,0.42203324055719205,0.42812801097953823,0.42812801107953824,0.4341240541176131,0.43412405421761313,0.4397712604979296,0.4397712605979296,0.4450103936899248,0.4450103937899248,0.4500783994158498,0.4500783995158498,0.455080586885594,0.455080586985594,0.4600630288784839,0.4600630289784839,0.46503888904575574,0.46503888914575575,0.4699094400031382,0.46990944010313823,0.4746549362737771,0.4746549363737771,0.4792227232527277,0.4792227233527277,0.483764182929206,0.483764183029206,0.48819375157017675,0.48819375167017676,0.49256408378058486,0.49256408388058487,0.4968817613860482,0.49688176148604823,0.5011731116890393,0.5011731117890393,0.505207770792925,0.505207770892925,0.509104211558831,0.509104211658831,0.5128887612892296,0.5128887613892296,0.5204249516219362,0.5204249517219363,0.5239791374557018,0.5239791375557018,0.5270528500193473,0.5270528501193473,0.5300475806755757,0.5300475807755757,0.5328843475169701,0.5328843476169701,0.5356947870558921,0.5356947871558921,0.5413025024825001,0.5413025025825001,0.5468707269553995,0.5468707270553995,0.549635093714995,0.549635093814995,0.5523336422184096,0.5523336423184096,0.5548676450813721,0.5548676451813721,0.5573489933393899,0.5573489934393899,0.5598237597717897,0.5598237598717897,0.5622853625529532,0.5622853626529533,0.5647140562060264,0.5647140563060264,0.5670242769979741,0.5670242770979741,0.569281843184977,0.569281843284977,0.5715328275463619,0.5715328276463619,0.5760150507922774,0.5760150508922774,0.5782331260255719,0.5782331261255719,0.5803919648283036,0.5803919649283036,0.5825310581541809,0.582531058254181,0.5846174968751138,0.5846174969751138,0.5866446991654838,0.5866446992654838,0.5885995013740548,0.5885995014740548,0.5904489943727366,0.5904489944727366,0.592278741894564,0.592278741994564,0.5940953257651553,0.5940953258651553,0.5958987459845104,0.5958987460845104,0.597577111517122,0.597577111617122,0.5992094042704069,0.5992094043704069,0.6007627151162749,0.6007627152162749,0.6023028623109066,0.6023028624109066,0.6038232640286841,0.6038232641286841,0.6052712656646627,0.6052712657646627,0.606541558008953,0.606541558108953,0.6078052685276252,0.6078052686276252,0.6089307607083176,0.6089307608083177,0.6099377800278846,0.6099377801278846,0.6118662549339833,0.6118662550339833,0.6127548013924247,0.6127548014924247,0.6135972750715395,0.6135972751715395,0.6144200032738001,0.6144200033738001,0.6152361496504425,0.6152361497504425,0.6160457142014669,0.6160457143014669,0.6168486969268732,0.6168486970268732,0.6184085895983592,0.6184085896983592,0.6191457540675847,0.6191457541675847,0.6198302639318655,0.6198302640318655,0.6218574662222355,0.6218574663222355,0.6224629941790992,0.6224629942790992,0.6230619403103449,0.6230619404103449,0.6248390332272277,0.6248390333272277,0.6259776890591563,0.6259776891591563,0.6265305624110754,0.6265305625110754,0.6270636902861403,0.6270636903861403,0.627590236335587,0.627590236435587,0.6280970369081795,0.6280970370081795,0.6285972556551539,0.6285972557551539,0.6290908925765103,0.6290908926765103,0.6295647840210123,0.6295647841210124,0.6300320936398963,0.6300320937398963],[0.0,1e-10,0.05649317555648309,0.05649317565648309,0.10365197179953956,0.10365197189953955,0.1471846467719032,0.14718464687190322,0.18722573854192126,0.18722573864192127,0.21632440075302878,0.2163244008530288,0.24166240362509622,0.24166240372509623,0.25778775210271787,0.2577877522027179,0.27294570780317595,0.27294570790317596,0.28591261496197856,0.28591261506197857,0.2968294182221563,0.2968294183221563,0.3058883148242052,0.3058883149242052,0.31380684113264395,0.31380684123264396,0.32168692799298354,0.32168692809298355,0.3292466861191629,0.3292466862191629,0.33658221413143025,0.33658221423143025,0.34373195147788466,0.34373195157788466,0.35040760229778206,0.35040760239778207,0.35673729808478655,0.35673729818478656,0.36277869801104684,0.36277869811104685,0.3688072847879408,0.3688072848879408,0.37473977294458666,0.37473977304458667,0.3801917679999923,0.38019176809999233,0.3852914014478218,0.3852914015478218,0.39022446395388805,0.39022446405388805,0.39509346071312224,0.39509346081312224,0.3999432377483068,0.3999432378483068,0.4047866082088082,0.4047866083088082,0.40952747347437835,0.40952747357437835,0.4141466138209676,0.41414661392096763,0.4186376226738929,0.4186376227738929,0.42308378550403564,0.42308378560403564,0.42750432203544564,0.42750432213544565,0.4318992322681228,0.43189923236812283,0.4362428899033344,0.4362428900033344,0.44055451466512996,0.44055451476512997,0.44480848025477665,0.44480848035477666,0.44901119324695776,0.44901119334695777,0.45318827994040606,0.45318828004040607,0.45698097215286215,0.45698097225286216,0.46066475259570383,0.46066475269570384,0.4643165001651295,0.4643165002651295,0.46784652281557426,0.46784652291557427,0.4783661184453934,0.4783661185453934,0.4816655044072429,0.4816655045072429,0.4849264509209932,0.4849264510209932,0.4879183212980489,0.4879183213980489,0.49083331277890624,0.49083331287890625,0.49359454646736667,0.4935945465673667,0.49631734070772787,0.4963173408077279,0.5017373028897175,0.5017373029897175,0.5044280642566628,0.5044280643566628,0.5070739796008256,0.5070739797008256,0.5097006752209388,0.5097006753209388,0.5122440853701703,0.5122440854701703,0.5147106166232034,0.5147106167232034,0.5171258952787708,0.5171258953787709,0.5195347673596552,0.5195347674596552,0.521930826291173,0.521930826391173,0.5242948523492749,0.5242948524492749,0.5265563732124455,0.5265563733124455,0.5287538283287841,0.5287538284287842,0.5309448768704396,0.5309448769704396,0.5331167056880453,0.5331167057880453,0.5352180621841358,0.5352180622841358,0.5373001989561768,0.5373001990561768,0.5393310831307521,0.5393310832307521,0.5413555607306442,0.5413555608306442,0.5452827910114476,0.5452827911114476,0.5470830384974276,0.5470830385974276,0.548864066259358,0.548864066359358,0.550632280871922,0.550632280971922,0.5522659574161387,0.5522659575161387,0.553854787937573,0.553854788037573,0.5553667395628089,0.5553667396628089,0.5568658780386784,0.5568658781386784,0.5583457967904982,0.5583457968904982,0.5595758591296732,0.5595758592296732,0.5607482622966994,0.5607482623966994,0.5618565997168935,0.5618565998168935,0.5628496187927899,0.56284961889279,0.5638106049952704,0.5638106050952704,0.564745964899018,0.564745964999018,0.565662105078716,0.565662105178716,0.5665269926609484,0.5665269927609484,0.5673470342203983,0.5673470343203983,0.5681478560557987,0.5681478561557987,0.5689294581671495,0.5689294582671495,0.5704798492404846,0.5704798493404846,0.5712294184784193,0.5712294185784194,0.571946954842938,0.571946954942938,0.5726132386099911,0.5726132387099911,0.5732667092276779,0.5732667093276779,0.5739137732706813,0.5739137733706813,0.5751438356098563,0.5751438357098563,0.5757076141819781,0.5757076142819781,0.5762585796047336,0.5762585797047336,0.5768031384528058,0.5768031385528058,0.5773412907261949,0.5773412908261949,0.5778602232755343,0.5778602233755343,0.5783727492501906,0.5783727493501906,0.5788660555007972,0.5788660556007972,0.5793529551767206,0.5793529552767206,0.5798334482779608,0.5798334483779608,0.5802947216551514,0.5802947217551514,0.5807495884576588,0.5807495885576588],[0.0,1e-10,2.9391814716607194,2.9391814717607194,5.420614643423599,5.420614643523599,7.612159183670287,7.612159183770287,9.734255976768367,9.734255976868367,11.83282668768616,11.83282668778616,13.877971556635291,13.877971556735291,15.909894780393346,15.909894780493346,17.9230867987038,17.923086798803798,19.7754295442892,19.775429544389198,21.60049736775561,21.60049736785561,23.425552196976135,23.425552197076133,25.21147485470484,25.211474854804838,26.982031816670943,26.98203181677094,28.742804111483352,28.74280411158335,30.467049581104494,30.467049581204492,32.151253282021656,32.15125328212166,33.75005879896264,33.75005879906264,35.139936333392846,35.13993633349285,36.481553238594884,36.481553238694886,37.788020708669784,37.788020708769785,39.07557505385465,39.07557505395465,40.3457690865331,40.345769086633105,41.61224676488757,41.612246764987574,42.82208902253904,42.82208902263904,44.021061593505166,44.02106159360517,45.21577854894296,45.21577854904296,46.40016507889976,46.40016507899976,47.519333488744515,47.51933348884452,48.62376642375224,48.62376642385224,49.71499720493771,49.71499720503771,50.80099130503031,50.80099130513031,51.88273628671752,51.88273628681752,52.96071293709719,52.960712937197194,54.01089489552236,54.010894895622364,55.02543363747665,55.025433637576654,56.017297420356314,56.017297420456316,56.99935054759052,56.99935054769052,57.96142502177186,57.96142502187186,58.80296487109578,58.80296487119578,59.63464208779069,59.63464208789069,60.4327421731109,60.432742173210904,61.216035315241704,61.216035315341706,61.99902958971708,61.999029589817084,62.72980648709139,62.72980648719139,63.460037626138394,63.460037626238396,64.17724203368265,64.17724203378265,64.89173714095924,64.89173714105924,65.57123224693642,65.57123224703642,66.23966275253991,66.23966275263992,66.9028630741735,66.9028630742735,67.56329562143291,67.56329562153292,68.19717442722013,68.19717442732014,68.81858525407779,68.81858525417779,69.4310885253792,69.4310885254792,70.03744551837555,70.03744551847555,70.63841639645132,70.63841639655132,71.2348262942204,71.2348262943204,71.83059947394095,71.83059947404095,72.41409309129732,72.41409309139732,72.91388427376589,72.91388427386589,73.4050017971042,73.4050017972042,73.88129288588428,73.88129288598428,74.35610912775604,74.35610912785604,74.82155651834255,74.82155651844255,75.28311862940852,75.28311862950852,75.74015874290546,75.74015874300547,76.19096811549909,76.19096811559909,76.64134218085547,76.64134218095548,77.09028038204123,77.09028038214123,77.5350734187887,77.5350734188887,77.96554679656754,77.96554679666754,78.39170608471157,78.39170608481157,78.8054233824178,78.8054233825178,79.20974584034698,79.20974584044698,79.61089770227947,79.61089770237948,80.00194653803403,80.00194653813404,80.39043550734864,80.39043550744864,80.77422055965178,80.77422055975178,81.15215170418236,81.15215170428236,81.50287939494633,81.50287939504634,81.84590799502165,81.84590799512165,82.17722877955184,82.17722877965184,82.50151317993367,82.50151318003367,82.82038547690311,82.82038547700311,83.13877048965178,83.13877048975178,83.44920952103989,83.44920952113989,83.75900533725655,83.75900533735656,84.06241448161924,84.06241448171924,84.36513493094988,84.36513493104988,84.6583500894134,84.6583500895134,84.95111044927086,84.95111044937086,85.24106405201648,85.24106405211649,85.5225713949349,85.5225713950349,85.79924487838295,85.79924487848295,86.07437854369329,86.07437854379329,86.34434049914017,86.34434049924018,86.61267817385105,86.61267817395105,86.87554527104308,86.87554527114308,87.13801604373552,87.13801604383552,87.39448347482768,87.39448347492768,87.90426073526122,87.90426073536122,88.13647440640335,88.13647440650335,88.35602518492762,88.35602518502762,88.5697805297858,88.5697805298858,88.78113843627764,88.78113843637765,88.9890138848715,88.9890138849715,89.18500609560074,89.18500609570074,89.3809853120841,89.38098531218411,89.57515832838902,89.57515832848902,89.76282122750403,89.76282122760404,89.94857397247351,89.94857397257351,90.12168331619392,90.12168331629393,90.2915246070735,90.2915246071735,90.45716875653123,90.45716875663123,90.61920700275503,90.61920700285503,90.78031616039786,90.78031616049786,90.94121091298351,90.94121091308351,91.09497832169959,91.09497832179959,91.24578304235318,91.24578304245318,91.38914206011295,91.38914206021295,91.5284273817868,91.5284273818868,91.65226904222273,91.65226904232273,91.77486975217634,91.77486975227635,91.89070046002229,91.89070046012229,92.00544614833659,92.00544614843659,92.11829467675125,92.11829467685125,92.23046100725676,92.23046100735677,92.34048328719076,92.34048328729077,92.44795219780777,92.44795219790777,92.55115249865055,92.55115249875055,92.6517799388075,92.6517799389075,92.74926277145954,92.74926277155954,92.84604391483363,92.84604391493363,92.93155254989985,92.93155254999985,93.01597616543442,93.01597616553443,93.10034780398544,93.10034780408544,93.17772204112575,93.17772204122575,93.25000253387795,93.25000253397795,93.3211980070985,93.3211980071985,93.39214658964717,93.39214658974717,93.46239348291789,93.46239348301789,93.53159433939463,93.53159433949463,93.5991319323977,93.5991319324977,93.66400570499371,93.66400570509371,93.72846366172132,93.72846366182132,93.7901603411977,93.7901603412977,93.84896580096402,93.84896580106403,93.90330124014484,93.90330124024484,93.95482992221385,93.95482992231385,94.0040781141295,94.0040781142295,94.05331980892221,94.05331980902221,94.10076829778238,94.10076829788238,94.14618318716106,94.14618318726106,94.19018170373795,94.19018170383795,94.2339073411512,94.2339073412512,94.27548243086996,94.27548243096996,94.31679763567097,94.31679763577097,94.35721623750571,94.35721623760571,94.39676422486595,94.39676422496595,94.43609131004608,94.43609131014608,94.47511303044786,94.47511303054786,94.51293928022793,94.51293928032793,94.55036270838548,94.55036270848548,94.58767568545296,94.58767568555297,94.6247287776027,94.6247287777027,94.66147000785112,94.66147000795112,94.69745107471509,94.69745107481509,94.73115165142572,94.73115165152572,94.76253275524532,94.76253275534532,94.7937774194831,94.79377741958311,94.82461926209835,94.82461926219835,94.85497382049282,94.85497382059282,94.8846981579617,94.8846981580617,94.91396769682449,94.91396769692449,94.94295136227775,94.94295136237776,94.97040820383917,94.97040820393917,94.99676703162305,94.99676703172305,95.02258010107964,95.02258010117964,95.04776294961066,95.04776294971066,95.07214665201955,95.07214665211956,95.09645888607606,95.09645888617607,95.12028383591178,95.12028383601178,95.14405680876395,95.14405680886395,95.16758938806717,95.16758938816717,95.19080360834616,95.19080360844616,95.21303026593763,95.21303026603763,95.23453574288234,95.23453574298235,95.25591127736816,95.25591127746816,95.27590942178985,95.27590942188985,95.2953553107613,95.2953553108613,95.31346928952921,95.31346928962921,95.33094655024861,95.33094655034861,95.34824838864854,95.34824838874854,95.36507593707354,95.36507593717354,95.3818969883756,95.3818969884756,95.39856860584996,95.39856860594996,95.41444757432514,95.41444757442514,95.4298067729648,95.4298067730648,95.46047969038351,95.46047969048351,95.47457844717202,95.47457844727202,95.48842381616572,95.48842381626572,95.5008788008494,95.50087880094941,95.5122292746326,95.5122292747326,95.52351477718636,95.52351477728637,95.53472881138774,95.53472881148774,95.54577392039258,95.54577392049258,95.55558457603804,95.55558457613805,95.56412179558647,95.56412179568648,95.57261353527431,95.57261353537432,95.58108578359331,95.58108578369331,95.58899927933912,95.58899927943912,95.59655543332302,95.59655543342302,95.61160277006137,95.61160277016137,95.61878209091451,95.61878209101451,95.6258834462923,95.62588344639231,95.63278339085886,95.63278339095886,95.63898164614746,95.63898164624746,95.64507594746894,95.64507594756894,95.65116375166748,95.65116375176748,95.65698517382533,95.65698517392534,95.6625012312048,95.6625012313048,95.66795231735483,95.66795231745483,95.67274719408752,95.67274719418752,95.67715224344356,95.67715224354356,95.68127791651301,95.68127791661301,95.68531262986124,95.68531262996125,95.68897700720167,95.68897700730167,95.69239449387022,95.69239449397023,95.695785992047,95.69578599214701,95.69902155927312,95.69902155937312,95.70208170417976,95.70208170427976,95.70498591813573,95.70498591823574,95.70785114935404,95.70785114945404,95.71061892372819,95.71061892382819,95.71328274413524,95.71328274423524,95.71590108468168,95.71590108478168,95.71835050003158,95.71835050013158,95.72064398443081,95.72064398453081,95.72293097170711,95.72293097180712,95.72507502227863,95.72507502237863,95.72716059874367,95.72716059884367,95.72923967808575,95.72923967818575,95.7311368379854,95.7311368380854,95.73296902665561,95.73296902675561,95.73265066763136,95.73265066773136],[0.0,1e-10,0.05754803093639073,0.05754803103639073,0.10139601458510036,0.10139601468510036,0.14137352274948659,0.1413735228494866,0.18062654056183247,0.18062654066183248,0.198764907123274,0.198764907223274,0.21158381749631147,0.21158381759631148,0.22085207245034058,0.2208520725503406,0.2300615849433935,0.2300615850433935,0.23925151661612096,0.23925151671612097,0.24827827478613665,0.24827827488613666,0.255901740832831,0.255901740932831,0.2622132919177227,0.2622132920177227,0.2683290347993602,0.2683290348993602,0.2736158562872219,0.2736158563872219,0.2785306421889007,0.27853064228890073,0.2831908774263492,0.2831908775263492,0.2877205738616282,0.2877205739616282,0.292191527835931,0.292191527935931,0.29626433846361705,0.29626433856361706,0.300017329025988,0.300017329125988,0.30372463100759967,0.3037246311075997,0.30711863986400467,0.3071186399640047,0.3104669601396504,0.3104669602396504,0.31350198729008955,0.31350198739008955,0.3165304875004202,0.3165304876004202,0.3194284489085814,0.3194284490085814,0.3221762906942478,0.3221762907942478,0.3248131744980701,0.32481317459807013,0.32744353136178406,0.32744353146178407,0.33001514576452173,0.33001514586452174,0.3325084368859577,0.3325084369859577,0.334936458606309,0.334936458706309,0.3373448995063349,0.3373448996063349,0.33966849018495066,0.33966849028495066,0.3419855539234579,0.3419855540234579,0.3442569290812059,0.34425692918120593,0.34646956177797766,0.34646956187797767,0.34864955977420703,0.34864955987420704,0.35078386918967713,0.35078386928967714,0.35290512472493024,0.35290512482493025,0.35501985332007496,0.35501985342007497,0.3568473965504469,0.3568473966504469,0.3582180539732258,0.3582180540732258,0.35947775341416077,0.3594777535141608,0.3606982912144449,0.3606982913144449,0.3631328398749047,0.3631328399749047,0.3642163119329109,0.3642163120329109,0.3652867301107002,0.3652867302107002,0.36613523232480144,0.36613523242480145,0.36689888431749257,0.3668988844174926,0.3676560093700752,0.3676560094700752,0.36833481114135624,0.36833481124135625,0.36900708597252874,0.36900708607252874,0.36964019916305046,0.3696401992630505,0.3701036119107519,0.3701036120107519,0.37052786301780255,0.37052786311780256]],"coord\_y":[[493400,493400,493400,300995,300995,297441,297441,279406,279406,268129,268129,227122,227122,220748,220748,195668,195668,188181,188181,186182,186182,182328,182328,179596,179596,179536,179536,175092,175092,173234,173234,171840,171840,157710,157710,152019,152019,142640,142640,140076,140076,135686,135686,135671,135671,134670,134670,131732,131732,126980,126980,124023,124023,122163,122163,122119,122119,121062,121062,120628,120628,112522,112522,109894,109894,99900,99900,97692,97692,97431,97431,97423,97423,96980,96980,96469,96469,95695,95695,95524,95524,95498,95498,91953,91953,91868,91868,91459,91459,91093,91093,90261,90261,89387,89387,87612,87612,87512,87512,85980,85980,83472,83472,83356,83356,82972,82972,81973,81973,80931,80931,79709,79709,76923,76923,76016,76016,73449,73449,69112,69112,68473,68473,65892,65892,65292,65292,64679,64679,64427,64427,63434,63434,63408,63408,60740,60740,60030,60030,57381,57381,56984,56984,56508,56508,55584,55584,55197,55197,53193,53193,52932,52932,52517,52517,50773,50773,49600,49600,49548,49548,48299,48299,45880,45880,45714,45714,45236,45236,43000,43000,42518,42518,41578,41578,41532,41532,41466,41466,41263,41263,40647,40647,39344,39344,38633,38633,37526,37526,37181,37181,36602,36602,36406,36406,35927,35927,33882,33882,32597,32597,32437,32437,32197,32197,31970,31970,31846,31846,30861,30861,28864,28864,28628,28628,28619,28619,28095,28095,26716,26716,26080,26080,25429,25429,24844,24844,24816,24816,24730,24730,24568,24568,23483,23483,23262,23262,23048,23048,22579,22579,21870,21870,21819,21819,21729,21729,21388,21388,20016,20016,19943,19943,19835,19835,19720,19720,18535,18535,18350,18350,18320,18320,17927,17927,17563,17563,17430,17430,17313,17313,17127,17127,16844,16844,16795,16795,15819,15819,15789,15789,15770,15770,15261,15261,15213,15213,14724,14724,14327,14327,14146,14146,13866,13866,13581,13581,12259,12259,12119,12119,12109,12109,12082,12082,11966,11966,11734,11734,11510,11510,11311,11311,11251,11251,11105,11105,10800,10800,10635,10635,10622,10622,10605,10605,10385,10385,9810,9810,9287,9287,9184,9184,8797,8797,8666,8666,8373,8373,8000,8000,7648,7648,7627,7627,7600,7600,7579,7579,7560,7560,7503,7503,7294,7294,7265,7265,7067,7067,7053,7053,6912,6912,6825,6825,6404,6404,6310,6310,6135,6135,6102,6102,6079,6079,6016,6016,6012,6012,5960,5960,5956,5956,5939,5939,5886,5886,5843,5843,5747,5747,5637,5637,5626,5626,5624,5624,5435,5435,5150,5150,5117,5117,5110,5110,5066,5066,5037,5037,4799,4799,4752,4752,4731,4731,4589,4589,4330,4330,4290,4290,4234,4234,4130,4130,3970,3970,3962,3962,3950,3950,3915,3915,3876,3876,3820,3820,3777,3777,3686,3686,3671,3671,3656,3656,3642,3642,3622,3622,3592,3592,3588,3588,3536,3536,3521,3521,3508,3508,3378,3378,3353,3353,3343,3343,3164,3164,3110,3110,3075,3075,3039,3039,3037,3037,3000,3000,2984,2984,2922,2922,2910,2910,2897,2897,2869,2869,2778,2778,2756,2756,2751,2751,2712,2712,2677,2677,2660,2660,2549,2549,2469,2469,2363,2363,2322,2322,2302,2302,2298,2298,2288,2288,2275,2275,2212,2212,2162,2162,2158,2158,2114,2114,2083,2083,2069,2069,2050,2050,2039,2039,2019,2019,2007,2007,1972,1972,1951,1951,1938,1938,1910,1910,1903,1903,1899,1899,1873,1873,1867,1867,1830,1830,1817,1817,1790,1790,1771,1771,1767,1767,1763,1763,1696,1696,1679,1679,1606,1606,1575,1575,1469,1469,1453,1453,1446,1446,1392,1392,1386,1386,1375,1375,1301,1301,1298,1298,1296,1296,1284,1284,1265,1265,1212,1212,1199,1199,1173,1173,1145,1145,1128,1128,1126,1126,1014,1014,1007,1007,1006,1006,964,964,939,939,933,933,922,922,882,882,867,867,843,843,830,830,823,823,763,763,715,715,701,701,673,673,602,602,593,593,544,544,537,537,506,506,466,466,461,461,445,445,432,432,391,391,385,385,384,384,327,327,312,312,311,311,294,294,288,288,282,282,260,260,176,176,139,139,81,81,-1538,-1538,0.0],[364389,364389,364389,320438,320438,307308,307308,297684,297684,278551,278551,277718,277718,221883,221883,217111,217111,205685,205685,194117,194117,190515,190515,188120,188120,177156,177156,173943,173943,172938,172938,168603,168603,167417,167417,165809,165809,164111,164111,138070,138070,134639,134639,130203,130203,121176,121176,120839,120839,120541,120541,115898,115898,113699,113699,110016,110016,102860,102860,102509,102509,102290,102290,101780,101780,101579,101579,99457,99457,99212,99212,98729,98729,97861,97861,97602,97602,96582,96582,96152,96152,95515,95515,94534,94534,89730,89730,89592,89592,89571,89571,84939,84939,82987,82987,80931,80931,79537,79537,77539,77539,76800,76800,75827,75827,73929,73929,73584,73584,73528,73528,70032,70032,67320,67320,64639,64639,63509,63509,63402,63402,62797,62797,61829,61829,61648,61648,60466,60466,60016,60016,59141,59141,57743,57743,57423,57423,57117,57117,57000,57000,56982,56982,55597,55597,52741,52741,51187,51187,51100,51100,50758,50758,50143,50143,49456,49456,48787,48787,48066,48066,46996,46996,45950,45950,45244,45244,44625,44625,44581,44581,43589,43589,43204,43204,43069,43069,42837,42837,42504,42504,41637,41637,41241,41241,39527,39527,39392,39392,38811,38811,38329,38329,37569,37569,36757,36757,35773,35773,34759,34759,34254,34254,33773,33773,33082,33082,32997,32997,32823,32823,31979,31979,31855,31855,31696,31696,31038,31038,29822,29822,29811,29811,29022,29022,28883,28883,28226,28226,28150,28150,27888,27888,27582,27582,27125,27125,27065,27065,26073,26073,25598,25598,25500,25500,25494,25494,24816,24816,24265,24265,23798,23798,23011,23011,22933,22933,22712,22712,21912,21912,21252,21252,20602,20602,19966,19966,19837,19837,19466,19466,19016,19016,18517,18517,18388,18388,18380,18380,18319,18319,17929,17929,17345,17345,17108,17108,16038,16038,15817,15817,15787,15787,15426,15426,15213,15213,15195,15195,14327,14327,13956,13956,13873,13873,13313,13313,12681,12681,12583,12583,12259,12259,12108,12108,12105,12105,11989,11989,11772,11772,11511,11511,10841,10841,10796,10796,10620,10620,10481,10481,10404,10404,10384,10384,9877,9877,9810,9810,9532,9532,9195,9195,9018,9018,8755,8755,8453,8453,8423,8423,8084,8084,8003,8003,7748,7748,7646,7646,7600,7600,7579,7579,7560,7560,7450,7450,7294,7294,7267,7267,7162,7162,7071,7071,7065,7065,6891,6891,6866,6866,6712,6712,6597,6597,6561,6561,6553,6553,6528,6528,6526,6526,6404,6404,6362,6362,6342,6342,6310,6310,6133,6133,6081,6081,6053,6053,6016,6016,6012,6012,5963,5963,5874,5874,5842,5842,5757,5757,5755,5755,5727,5727,5689,5689,5637,5637,5636,5636,5624,5624,5569,5569,5555,5555,5463,5463,5351,5351,5191,5191,5150,5150,5117,5117,5066,5066,5035,5035,4799,4799,4751,4751,4733,4733,4677,4677,4654,4654,4590,4590,4330,4330,4290,4290,4234,4234,4118,4118,4049,4049,3957,3957,3915,3915,3874,3874,3777,3777,3772,3772,3717,3717,3705,3705,3686,3686,3666,3666,3656,3656,3645,3645,3644,3644,3633,3633,3605,3605,3574,3574,3510,3510,3353,3353,3278,3278,3137,3137,3072,3072,3039,3039,3036,3036,3003,3003,2980,2980,2947,2947,2925,2925,2909,2909,2896,2896,2885,2885,2869,2869,2778,2778,2756,2756,2749,2749,2712,2712,2677,2677,2660,2660,2600,2600,2567,2567,2487,2487,2469,2469,2420,2420,2365,2365,2326,2326,2302,2302,2297,2297,2288,2288,2279,2279,2270,2270,2267,2267,2212,2212,2192,2192,2158,2158,2120,2120,2048,2048,2039,2039,2017,2017,2007,2007,1982,1982,1971,1971,1951,1951,1922,1922,1912,1912,1903,1903,1899,1899,1896,1896,1873,1873,1867,1867,1861,1861,1830,1830,1817,1817,1797,1797,1790,1790,1763,1763,1696,1696,1606,1606,1469,1469,1375,1375,1316,1316,1301,1301,1300,1300,1298,1298,1296,1296,1284,1284,1199,1199,1192,1192,1145,1145,1128,1128,1126,1126,1053,1053,1007,1007,1006,1006,939,939,933,933,924,924,914,914,882,882,843,843,823,823,781,781,763,763,757,757,752,752,722,722,715,715,673,673,602,602,593,593,544,544,506,506,478,478,466,466,460,460,441,441,432,432,414,414,413,413,385,385,384,384,327,327,320,320,311,311,296,296,282,282,78,78,0.0],[496851,496851,496851,367480,367480,325014,325014,313713,313713,297207,297207,280419,280419,242504,242504,225059,225059,194216,194216,187841,187841,183027,183027,180450,180450,178743,178743,176371,176371,167346,167346,166403,166403,165496,165496,151792,151792,146367,146367,143035,143035,137134,137134,136728,136728,136135,136135,133397,133397,131047,131047,130368,130368,127177,127177,122897,122897,122706,122706,122473,122473,121735,121735,120951,120951,120509,120509,119792,119792,114888,114888,109309,109309,101524,101524,101422,101422,101346,101346,100843,100843,100289,100289,98636,98636,97499,97499,96116,96116,95540,95540,95417,95417,92172,92172,90779,90779,89660,89660,89006,89006,87855,87855,87265,87265,85492,85492,84103,84103,82410,82410,81958,81958,80691,80691,79587,79587,77848,77848,77826,77826,77536,77536,76848,76848,73856,73856,73546,73546,68529,68529,65918,65918,64048,64048,63842,63842,63432,63432,63022,63022,62731,62731,61806,61806,60712,60712,59717,59717,59324,59324,57377,57377,56997,56997,56164,56164,55553,55553,53454,53454,52721,52721,50155,50155,49589,49589,49454,49454,49204,49204,45953,45953,45347,45347,44642,44642,42988,42988,41212,41212,41188,41188,39905,39905,39487,39487,39406,39406,38840,38840,38604,38604,38245,38245,37563,37563,37391,37391,36592,36592,35784,35784,34371,34371,33855,33855,32606,32606,32429,32429,31982,31982,31932,31932,31794,31794,30974,30974,30058,30058,29822,29822,29639,29639,28755,28755,27725,27725,27301,27301,26592,26592,26421,26421,26088,26088,25589,25589,24824,24824,24500,24500,23588,23588,22669,22669,22383,22383,21755,21755,21632,21632,21390,21390,20696,20696,20552,20552,19943,19943,19885,19885,19855,19855,18508,18508,18377,18377,18354,18354,18334,18334,17931,17931,17903,17903,17427,17427,17345,17345,15816,15816,15214,15214,14317,14317,14137,14137,12740,12740,12254,12254,12119,12119,12108,12108,11947,11947,11742,11742,11511,11511,11382,11382,11315,11315,11152,11152,10905,10905,10846,10846,10803,10803,10620,10620,10483,10483,9807,9807,9800,9800,9486,9486,8987,8987,8405,8405,8003,8003,7647,7647,7579,7579,7503,7503,7456,7456,7085,7085,7053,7053,7037,7037,7026,7026,6912,6912,6902,6902,6597,6597,6099,6099,6078,6078,6054,6054,6016,6016,5845,5845,5748,5748,5635,5635,5379,5379,5191,5191,5150,5150,5119,5119,5066,5066,4789,4789,4772,4772,4751,4751,4712,4712,4677,4677,4590,4590,4310,4310,4293,4293,4118,4118,4066,4066,3993,3993,3965,3965,3831,3831,3811,3811,3777,3777,3627,3627,3521,3521,3452,3452,3374,3374,3134,3134,3075,3075,3039,3039,3036,3036,3000,3000,2956,2956,2801,2801,2769,2769,2692,2692,2660,2660,2576,2576,2567,2567,2367,2367,2302,2302,2297,2297,2275,2275,2267,2267,2222,2222,2195,2195,2148,2148,2125,2125,2099,2099,2048,2048,2019,2019,2007,2007,1995,1995,1880,1880,1871,1871,1867,1867,1607,1607,1575,1575,1488,1488,1433,1433,1375,1375,1296,1296,1284,1284,1222,1222,1206,1206,1199,1199,1145,1145,1128,1128,1126,1126,1024,1024,1014,1014,1007,1007,1006,1006,978,978,963,963,961,961,939,939,924,924,843,843,823,823,763,763,743,743,673,673,665,665,602,602,544,544,521,521,506,506,469,469,432,432,405,405,385,385,384,384,375,375,372,372,360,360,328,328,327,327,275,275,268,268,195,195,158,158,0.0],[320809,320809,320809,297765,297765,291837,291837,278139,278139,277628,277628,240342,240342,239951,239951,221776,221776,205721,205721,194335,194335,190153,190153,188167,188167,180240,180240,174023,174023,172913,172913,172476,172476,165821,165821,151735,151735,142637,142637,138071,138071,134635,134635,130228,130228,127219,127219,122649,122649,122042,122042,121208,121208,120855,120855,120542,120542,120207,120207,112526,112526,109988,109988,102424,102424,102274,102274,101853,101853,101765,101765,101569,101569,100017,100017,99468,99468,99190,99190,98730,98730,97606,97606,96580,96580,96564,96564,95515,95515,95493,95493,90265,90265,89605,89605,89192,89192,87943,87943,87702,87702,87525,87525,81871,81871,80933,80933,76805,76805,73572,73572,73528,73528,73446,73446,70793,70793,70052,70052,69081,69081,67033,67033,64639,64639,63510,63510,63463,63463,63226,63226,61648,61648,60016,60016,58068,58068,57391,57391,57117,57117,56998,56998,56981,56981,55640,55640,52748,52748,51078,51078,50750,50750,50294,50294,50140,50140,49785,49785,48088,48088,47000,47000,46587,46587,45942,45942,45261,45261,44898,44898,44629,44629,44598,44598,43564,43564,43098,43098,43072,43072,42836,42836,41641,41641,41483,41483,41241,41241,39529,39529,39525,39525,39407,39407,38811,38811,38327,38327,37532,37532,36768,36768,35752,35752,34084,34084,32997,32997,32807,32807,32545,32545,31970,31970,31930,31930,31856,31856,31707,31707,31694,31694,31038,31038,29798,29798,28892,28892,28152,28152,28150,28150,27577,27577,27068,27068,26052,26052,25598,25598,25498,25498,25490,25490,25159,25159,24820,24820,23798,23798,22709,22709,22112,22112,21405,21405,20599,20599,19942,19942,19854,19854,19435,19435,18517,18517,18385,18385,18347,18347,18304,18304,18232,18232,17929,17929,17643,17643,17334,17334,17124,17124,15817,15817,15789,15789,15426,15426,15220,15220,15195,15195,14326,14326,13956,13956,13873,13873,13294,13294,12681,12681,12581,12581,12256,12256,12109,12109,12108,12108,11989,11989,11772,11772,11504,11504,10841,10841,10796,10796,10558,10558,10484,10484,10384,10384,9876,9876,9810,9810,9240,9240,9195,9195,9016,9016,8755,8755,8453,8453,8423,8423,8361,8361,8003,8003,7748,7748,7647,7647,7600,7600,7579,7579,7560,7560,7455,7455,7294,7294,7286,7286,7162,7162,7071,7071,7059,7059,6891,6891,6866,6866,6825,6825,6597,6597,6559,6559,6553,6553,6528,6528,6526,6526,6404,6404,6354,6354,6310,6310,6179,6179,6138,6138,6121,6121,6093,6093,6081,6081,6012,6012,5998,5998,5923,5923,5902,5902,5894,5894,5885,5885,5842,5842,5757,5757,5755,5755,5748,5748,5725,5725,5687,5687,5676,5676,5660,5660,5637,5637,5636,5636,5578,5578,5556,5556,5479,5479,5463,5463,5191,5191,5150,5150,5117,5117,5066,5066,5035,5035,4930,4930,4799,4799,4751,4751,4733,4733,4677,4677,4590,4590,4386,4386,4330,4330,4290,4290,4234,4234,4118,4118,3965,3965,3957,3957,3915,3915,3874,3874,3777,3777,3772,3772,3717,3717,3705,3705,3701,3701,3699,3699,3686,3686,3656,3656,3645,3645,3610,3610,3604,3604,3510,3510,3353,3353,3260,3260,3190,3190,3183,3183,3137,3137,3072,3072,3039,3039,3036,3036,2987,2987,2977,2977,2947,2947,2924,2924,2909,2909,2896,2896,2869,2869,2778,2778,2756,2756,2749,2749,2712,2712,2677,2677,2660,2660,2600,2600,2567,2567,2487,2487,2469,2469,2420,2420,2365,2365,2326,2326,2302,2302,2297,2297,2288,2288,2279,2279,2275,2275,2267,2267,2212,2212,2192,2192,2184,2184,2158,2158,2148,2148,2088,2088,2071,2071,2039,2039,2017,2017,2007,2007,1972,1972,1943,1943,1911,1911,1903,1903,1899,1899,1896,1896,1873,1873,1867,1867,1831,1831,1830,1830,1817,1817,1796,1796,1790,1790,1773,1773,1763,1763,1696,1696,1606,1606,1594,1594,1501,1501,1488,1488,1469,1469,1374,1374,1316,1316,1301,1301,1300,1300,1298,1298,1296,1296,1284,1284,1265,1265,1199,1199,1145,1145,1128,1128,1126,1126,1053,1053,1025,1025,1024,1024,1007,1007,1006,1006,963,963,939,939,933,933,923,923,895,895,882,882,843,843,781,781,757,757,752,752,722,722,715,715,673,673,602,602,593,593,565,565,544,544,506,506,466,466,460,460,445,445,441,441,432,432,414,414,413,413,385,385,384,384,327,327,325,325,320,320,296,296,267,267,182,182,78,78,0.0],[480703,480703,480703,356328,356328,321331,321331,296990,296990,292116,292116,280580,280580,242008,242008,227234,227234,222201,222201,198095,198095,190841,190841,190486,190486,188214,188214,173847,173847,167422,167422,165825,165825,163706,163706,146279,146279,144445,144445,136950,136950,134703,134703,130737,130737,129554,129554,126406,126406,122109,122109,122006,122006,120602,120602,116718,116718,116387,116387,108933,108933,103198,103198,102237,102237,101348,101348,98571,98571,97597,97597,97116,97116,96991,96991,95541,95541,95503,95503,94875,94875,93332,93332,91876,91876,90259,90259,87966,87966,87898,87898,87719,87719,87127,87127,84522,84522,82632,82632,81971,81971,80932,80932,78949,78949,78006,78006,77948,77948,77699,77699,76803,76803,76141,76141,75826,75826,75409,75409,74988,74988,74081,74081,73556,73556,72689,72689,69165,69165,65322,65322,64031,64031,63542,63542,63312,63312,63058,63058,61814,61814,60008,60008,57386,57386,57357,57357,57148,57148,56987,56987,56956,56956,55587,55587,52723,52723,51383,51383,51091,51091,50129,50129,49559,49559,49440,49440,49235,49235,48599,48599,47130,47130,47015,47015,45942,45942,45483,45483,45336,45336,44703,44703,44626,44626,42488,42488,42389,42389,41259,41259,40250,40250,40001,40001,39640,39640,39542,39542,39407,39407,38939,38939,38239,38239,38083,38083,37709,37709,36562,36562,35773,35773,34372,34372,33862,33862,32998,32998,32381,32381,32089,32089,31851,31851,31791,31791,30555,30555,29897,29897,29633,29633,28995,28995,28870,28870,28776,28776,28477,28477,28392,28392,28149,28149,27933,27933,27533,27533,26089,26089,25758,25758,25589,25589,24850,24850,24631,24631,22715,22715,22309,22309,21748,21748,21412,21412,20591,20591,19947,19947,19858,19858,18507,18507,18347,18347,18319,18319,17921,17921,17426,17426,17336,17336,17122,17122,15910,15910,15799,15799,15266,15266,15199,15199,15056,15056,14316,14316,14143,14143,14034,14034,12642,12642,12259,12259,12114,12114,12107,12107,11965,11965,11730,11730,11514,11514,11309,11309,11211,11211,11149,11149,10840,10840,10793,10793,10620,10620,10571,10571,10529,10529,9810,9810,9309,9309,8003,8003,7968,7968,7578,7578,7508,7508,7447,7447,7208,7208,7085,7085,7074,7074,7046,7046,6891,6891,6597,6597,6552,6552,6275,6275,6135,6135,6096,6096,6016,6016,5979,5979,5972,5972,5969,5969,5968,5968,5956,5956,5885,5885,5844,5844,5757,5757,5739,5739,5612,5612,5558,5558,5191,5191,5115,5115,5069,5069,5066,5066,4798,4798,4751,4751,4723,4723,4677,4677,4590,4590,4310,4310,4290,4290,4234,4234,4125,4125,3965,3965,3957,3957,3888,3888,3874,3874,3777,3777,3669,3669,3656,3656,3651,3651,3610,3610,3536,3536,3499,3499,3454,3454,3387,3387,3353,3353,3137,3137,3072,3072,3033,3033,3006,3006,2922,2922,2839,2839,2660,2660,2488,2488,2450,2450,2365,2365,2356,2356,2352,2352,2300,2300,2297,2297,2275,2275,2267,2267,2236,2236,2195,2195,2141,2141,2114,2114,2110,2110,2068,2068,2039,2039,2007,2007,2006,2006,1994,1994,1978,1978,1933,1933,1867,1867,1738,1738,1607,1607,1575,1575,1446,1446,1374,1374,1296,1296,1284,1284,1248,1248,1211,1211,1206,1206,1199,1199,1145,1145,1128,1128,1126,1126,1049,1049,1014,1014,1006,1006,1005,1005,963,963,961,961,939,939,925,925,843,843,823,823,782,782,781,781,763,763,759,759,757,757,752,752,681,681,673,673,602,602,544,544,537,537,507,507,469,469,440,440,429,429,384,384,372,372,360,360,327,327,316,316,310,310,294,294,268,268,226,226,195,195,140,140,0.0],[480720,480720,480720,357124,357124,322635,322635,289755,289755,288371,288371,280583,280583,240889,240889,234853,234853,222262,222262,198091,198091,190846,190846,190487,190487,188224,188224,173860,173860,167401,167401,165830,165830,163709,163709,146279,146279,144447,144447,136946,136946,135594,135594,134698,134698,129552,129552,127098,127098,122744,122744,122105,122105,122013,122013,120604,120604,119390,119390,116717,116717,108934,108934,102234,102234,101509,101509,101347,101347,99288,99288,96987,96987,96147,96147,95540,95540,95511,95511,94873,94873,93584,93584,93335,93335,91872,91872,89641,89641,87896,87896,87718,87718,87083,87083,84495,84495,81967,81967,80931,80931,78950,78950,78001,78001,77948,77948,77891,77891,77699,77699,76803,76803,76143,76143,75831,75831,74979,74979,74934,74934,74079,74079,73560,73560,69098,69098,67044,67044,67005,67005,65975,65975,65328,65328,64031,64031,63541,63541,63250,63250,63058,63058,60007,60007,57387,57387,57035,57035,56985,56985,56216,56216,55589,55589,54727,54727,52723,52723,51092,51092,50128,50128,49559,49559,49235,49235,48769,48769,47379,47379,45962,45962,45335,45335,45320,45320,45290,45290,44639,44639,44191,44191,43539,43539,42493,42493,42390,42390,41255,41255,40250,40250,40001,40001,39639,39639,39544,39544,39406,39406,38239,38239,37800,37800,37581,37581,36562,36562,35929,35929,34370,34370,32433,32433,32087,32087,31965,31965,31851,31851,31794,31794,31662,31662,30899,30899,30041,30041,29796,29796,29633,29633,28868,28868,28775,28775,28167,28167,28149,28149,27530,27530,26089,26089,25759,25759,25589,25589,24857,24857,24850,24850,22714,22714,22392,22392,22308,22308,21748,21748,21417,21417,20589,20589,19950,19950,19858,19858,19823,19823,18507,18507,18344,18344,17923,17923,17425,17425,17336,17336,17123,17123,17011,17011,15911,15911,15799,15799,15264,15264,15202,15202,14667,14667,14316,14316,14144,14144,14031,14031,12639,12639,12259,12259,12114,12114,12108,12108,11967,11967,11730,11730,11514,11514,11309,11309,11211,11211,11150,11150,10841,10841,10793,10793,10622,10622,10571,10571,9810,9810,9287,9287,9147,9147,8003,8003,7968,7968,7578,7578,7509,7509,7449,7449,7074,7074,7046,7046,6891,6891,6597,6597,6553,6553,6466,6466,6135,6135,6096,6096,6016,6016,5971,5971,5955,5955,5754,5754,5739,5739,5612,5612,5556,5556,5202,5202,5193,5193,5150,5150,5115,5115,5066,5066,4751,4751,4677,4677,4590,4590,4310,4310,4290,4290,4234,4234,4125,4125,4063,4063,3965,3965,3957,3957,3888,3888,3852,3852,3777,3777,3656,3656,3651,3651,3391,3391,3353,3353,3312,3312,3138,3138,3072,3072,3033,3033,3003,3003,2921,2921,2660,2660,2487,2487,2365,2365,2297,2297,2275,2275,2268,2268,2236,2236,2212,2212,2195,2195,2188,2188,2133,2133,2118,2118,2114,2114,2110,2110,2100,2100,2087,2087,2068,2068,2039,2039,2007,2007,1994,1994,1992,1992,1933,1933,1876,1876,1867,1867,1775,1775,1771,1771,1648,1648,1607,1607,1575,1575,1446,1446,1376,1376,1284,1284,1248,1248,1211,1211,1206,1206,1199,1199,1145,1145,1126,1126,1049,1049,1014,1014,1006,1006,1005,1005,963,963,961,961,939,939,924,924,843,843,782,782,779,779,763,763,759,759,681,681,673,673,602,602,544,544,537,537,507,507,469,469,429,429,385,385,384,384,372,372,335,335,327,327,316,316,310,310,268,268,265,265,0.0],[479849,479849,479849,358929,358929,322733,322733,296908,296908,296020,296020,280401,280401,244610,244610,222470,222470,196525,196525,192311,192311,191024,191024,173872,173872,167389,167389,165479,165479,163716,163716,158656,158656,139153,139153,135090,135090,134674,134674,133716,133716,130044,130044,126407,126407,122561,122561,122141,122141,121996,121996,121204,121204,120693,120693,119778,119778,116686,116686,108906,108906,102249,102249,101842,101842,101502,101502,101437,101437,98620,98620,96991,96991,95540,95540,95479,95479,94795,94795,92632,92632,90796,90796,90271,90271,89158,89158,87974,87974,87914,87914,87748,87748,86715,86715,84719,84719,81746,81746,80911,80911,80619,80619,79698,79698,78536,78536,76793,76793,76037,76037,75480,75480,75171,75171,74089,74089,73549,73549,71881,71881,69153,69153,66982,66982,65400,65400,64718,64718,63577,63577,63500,63500,61814,61814,60003,60003,57452,57452,57384,57384,57368,57368,57042,57042,56988,56988,56853,56853,56517,56517,55585,55585,55185,55185,53953,53953,53471,53471,52511,52511,51167,51167,50151,50151,49555,49555,49456,49456,49241,49241,48769,48769,47024,47024,45940,45940,45336,45336,45271,45271,44647,44647,43805,43805,42437,42437,41254,41254,39910,39910,39641,39641,39563,39563,39406,39406,38594,38594,38477,38477,38337,38337,37507,37507,36566,36566,35780,35780,34372,34372,33856,33856,33618,33618,32998,32998,32434,32434,31965,31965,31849,31849,31792,31792,30544,30544,29639,29639,29433,29433,29355,29355,29040,29040,28863,28863,28470,28470,28167,28167,28150,28150,27933,27933,27548,27548,26104,26104,25873,25873,25589,25589,24957,24957,24818,24818,24757,24757,24628,24628,22712,22712,22390,22390,21758,21758,21544,21544,21404,21404,20994,20994,19949,19949,19858,19858,18508,18508,18347,18347,18319,18319,17921,17921,17426,17426,17341,17341,17093,17093,15799,15799,15213,15213,14995,14995,14494,14494,14326,14326,14264,14264,14143,14143,14031,14031,12259,12259,12110,12110,12108,12108,11965,11965,11730,11730,11560,11560,11511,11511,11508,11508,11373,11373,11309,11309,11211,11211,11149,11149,10831,10831,10793,10793,10571,10571,9810,9810,9287,9287,9117,9117,8062,8062,8003,8003,7748,7748,7647,7647,7579,7579,7509,7509,7298,7298,7142,7142,7076,7076,7074,7074,6970,6970,6906,6906,6826,6826,6777,6777,6641,6641,6553,6553,6528,6528,6135,6135,5959,5959,5958,5958,5940,5940,5885,5885,5849,5849,5845,5845,5785,5785,5755,5755,5748,5748,5689,5689,5681,5681,5636,5636,5555,5555,5418,5418,5352,5352,5344,5344,5309,5309,5246,5246,5199,5199,5191,5191,5149,5149,5115,5115,5066,5066,4993,4993,4798,4798,4755,4755,4751,4751,4730,4730,4683,4683,4590,4590,4584,4584,4355,4355,4310,4310,4290,4290,4254,4254,4234,4234,4189,4189,4145,4145,4120,4120,4087,4087,3965,3965,3957,3957,3888,3888,3876,3876,3804,3804,3777,3777,3756,3756,3735,3735,3657,3657,3656,3656,3628,3628,3624,3624,3612,3612,3610,3610,3539,3539,3353,3353,3190,3190,3187,3187,3137,3137,3072,3072,3061,3061,3033,3033,3000,3000,2993,2993,2922,2922,2860,2860,2660,2660,2591,2591,2487,2487,2372,2372,2364,2364,2302,2302,2297,2297,2275,2275,2267,2267,2236,2236,2197,2197,2192,2192,2146,2146,2100,2100,2077,2077,2067,2067,2039,2039,2019,2019,2016,2016,2007,2007,1998,1998,1896,1896,1842,1842,1771,1771,1637,1637,1607,1607,1598,1598,1468,1468,1449,1449,1446,1446,1434,1434,1375,1375,1296,1296,1292,1292,1284,1284,1255,1255,1206,1206,1201,1201,1199,1199,1145,1145,1128,1128,1126,1126,1077,1077,1067,1067,1030,1030,1027,1027,1006,1006,1003,1003,1002,1002,963,963,939,939,924,924,906,906,843,843,781,781,763,763,757,757,752,752,673,673,655,655,632,632,602,602,588,588,544,544,537,537,519,519,506,506,471,471,457,457,450,450,441,441,429,429,387,387,385,385,384,384,369,369,359,359,333,333,327,327,314,314,313,313,268,268,70,70,0.0],[496778,496778,496778,361662,361662,299014,299014,296531,296531,279444,279444,242349,242349,233614,233614,222817,222817,191221,191221,189420,189420,176582,176582,167969,167969,167372,167372,165720,165720,163737,163737,146350,146350,139017,139017,137824,137824,137583,137583,135925,135925,135590,135590,134675,134675,130709,130709,130362,130362,128954,128954,122509,122509,122201,122201,120923,120923,120578,120578,118363,118363,112552,112552,108456,108456,102237,102237,101403,101403,101341,101341,100355,100355,98699,98699,97609,97609,96944,96944,96199,96199,95945,95945,95532,95532,95429,95429,94494,94494,91877,91877,91606,91606,90330,90330,89617,89617,87838,87838,87836,87836,87295,87295,85260,85260,83777,83777,81954,81954,79621,79621,79093,79093,77829,77829,77083,77083,76981,76981,76359,76359,74800,74800,73556,73556,67491,67491,66922,66922,64665,64665,64567,64567,64064,64064,64015,64015,63490,63490,63351,63351,63019,63019,62934,62934,62784,62784,58203,58203,57389,57389,57012,57012,56994,56994,56933,56933,56508,56508,55568,55568,53277,53277,52742,52742,51964,51964,50148,50148,49559,49559,49195,49195,48978,48978,47715,47715,47656,47656,47000,47000,46906,46906,45908,45908,44431,44431,44346,44346,44211,44211,43543,43543,42994,42994,41982,41982,41672,41672,41247,41247,39695,39695,39638,39638,39537,39537,39219,39219,38816,38816,38375,38375,37560,37560,36579,36579,36144,36144,34368,34368,34273,34273,32997,32997,32386,32386,31970,31970,31855,31855,31513,31513,31013,31013,30779,30779,30442,30442,29822,29822,29646,29646,29409,29409,29051,29051,28891,28891,28150,28150,27969,27969,26098,26098,25589,25589,25497,25497,24839,24839,24833,24833,24558,24558,24514,24514,23896,23896,22735,22735,22391,22391,21749,21749,21375,21375,20600,20600,20465,20465,19940,19940,19854,19854,18508,18508,18450,18450,18385,18385,18319,18319,17918,17918,17342,17342,17122,17122,15817,15817,15410,15410,15213,15213,14325,14325,14140,14140,14031,14031,13871,13871,12262,12262,12116,12116,12108,12108,11969,11969,11730,11730,11509,11509,11216,11216,11067,11067,10844,10844,10797,10797,10621,10621,10484,10484,9825,9825,9287,9287,9105,9105,8392,8392,8002,8002,7647,7647,7579,7579,7459,7459,7309,7309,7071,7071,7061,7061,6900,6900,6829,6829,6575,6575,6553,6553,6522,6522,6135,6135,6078,6078,6042,6042,6013,6013,5956,5956,5885,5885,5842,5842,5757,5757,5754,5754,5689,5689,5681,5681,5636,5636,5477,5477,5467,5467,5199,5199,5191,5191,5150,5150,4793,4793,4751,4751,4677,4677,4634,4634,4590,4590,4573,4573,4540,4540,4290,4290,4283,4283,4234,4234,4106,4106,3959,3959,3957,3957,3874,3874,3777,3777,3654,3654,3637,3637,3629,3629,3575,3575,3536,3536,3378,3378,3353,3353,3172,3172,3137,3137,3075,3075,3039,3039,3036,3036,3009,3009,3000,3000,2925,2925,2902,2902,2795,2795,2714,2714,2660,2660,2567,2567,2490,2490,2365,2365,2297,2297,2267,2267,2225,2225,2183,2183,2095,2095,2055,2055,2019,2019,2007,2007,1907,1907,1896,1896,1867,1867,1745,1745,1607,1607,1599,1599,1575,1575,1508,1508,1449,1449,1422,1422,1375,1375,1354,1354,1296,1296,1292,1292,1284,1284,1199,1199,1160,1160,1145,1145,1128,1128,1126,1126,1124,1124,1112,1112,1014,1014,1006,1006,1005,1005,963,963,939,939,924,924,843,843,823,823,781,781,763,763,761,761,757,757,679,679,673,673,672,672,603,603,602,602,574,574,544,544,529,529,506,506,481,481,441,441,432,432,404,404,385,385,384,384,374,374,327,327,291,291,268,268,195,195,158,158,109,109,85,85,0.0],[496170,496170,496170,299078,299078,297455,297455,279476,279476,241225,241225,225173,225173,224492,224492,197817,197817,195893,195893,188076,188076,183753,183753,183443,183443,182772,182772,179071,179071,178306,178306,172223,172223,168957,168957,167438,167438,142853,142853,141373,141373,140083,140083,138038,138038,135671,135671,134628,134628,123357,123357,123075,123075,122761,122761,122110,122110,121260,121260,120642,120642,112539,112539,109923,109923,99893,99893,99694,99694,99215,99215,98650,98650,97425,97425,97409,97409,96976,96976,96250,96250,95489,95489,95471,95471,93657,93657,90261,90261,89629,89629,89029,89029,88268,88268,87742,87742,87612,87612,86328,86328,85310,85310,84476,84476,83086,83086,80926,80926,79619,79619,77806,77806,76794,76794,76238,76238,74731,74731,73559,73559,73449,73449,68492,68492,66570,66570,65283,65283,64666,64666,64453,64453,63492,63492,63392,63392,60725,60725,60016,60016,57363,57363,57273,57273,57139,57139,56975,56975,56837,56837,55579,55579,52930,52930,52515,52515,51061,51061,50195,50195,49559,49559,48166,48166,46688,46688,45884,45884,45236,45236,44491,44491,42683,42683,42345,42345,42212,42212,41532,41532,41257,41257,39706,39706,39409,39409,39335,39335,38926,38926,38577,38577,38334,38334,37575,37575,36600,36600,36404,36404,35772,35772,33891,33891,32973,32973,32600,32600,32440,32440,31970,31970,31844,31844,31698,31698,30548,30548,29819,29819,29559,29559,28842,28842,28628,28628,27587,27587,26084,26084,25589,25589,25283,25283,24845,24845,24824,24824,24094,24094,23045,23045,22696,22696,22584,22584,22545,22545,22386,22386,21820,21820,21750,21750,21385,21385,20609,20609,19942,19942,19834,19834,18451,18451,18440,18440,18280,18280,18152,18152,17927,17927,17565,17565,17126,17126,15949,15949,15819,15819,15787,15787,15434,15434,15213,15213,14327,14327,14146,14146,12570,12570,12259,12259,12122,12122,12108,12108,11968,11968,11730,11730,11511,11511,11313,11313,11311,11311,11253,11253,11215,11215,10841,10841,10621,10621,10591,10591,10484,10484,9810,9810,9287,9287,9184,9184,8424,8424,8374,8374,7999,7999,7914,7914,7647,7647,7600,7600,7579,7579,7560,7560,7530,7530,7294,7294,7265,7265,7076,7076,7053,7053,6918,6918,6902,6902,6829,6829,6784,6784,6725,6725,6576,6576,6553,6553,6525,6525,6521,6521,6404,6404,6310,6310,6135,6135,6102,6102,6078,6078,6075,6075,6016,6016,6012,6012,5885,5885,5842,5842,5823,5823,5779,5779,5765,5765,5747,5747,5689,5689,5637,5637,5636,5636,5624,5624,5579,5579,5150,5150,5125,5125,5117,5117,5110,5110,5066,5066,5035,5035,5006,5006,4798,4798,4751,4751,4730,4730,4677,4677,4591,4591,4330,4330,4290,4290,4130,4130,4093,4093,3962,3962,3957,3957,3915,3915,3874,3874,3777,3777,3772,3772,3768,3768,3727,3727,3686,3686,3671,3671,3656,3656,3653,3653,3642,3642,3631,3631,3622,3622,3599,3599,3598,3598,3536,3536,3506,3506,3402,3402,3353,3353,3336,3336,3190,3190,3110,3110,3075,3075,3036,3036,3000,3000,2987,2987,2922,2922,2910,2910,2896,2896,2869,2869,2778,2778,2756,2756,2751,2751,2712,2712,2677,2677,2660,2660,2644,2644,2571,2571,2469,2469,2364,2364,2302,2302,2297,2297,2288,2288,2275,2275,2266,2266,2225,2225,2212,2212,2197,2197,2185,2185,2158,2158,2114,2114,2108,2108,2050,2050,2039,2039,2019,2019,2007,2007,1971,1971,1951,1951,1911,1911,1903,1903,1899,1899,1873,1873,1867,1867,1842,1842,1832,1832,1830,1830,1817,1817,1790,1790,1771,1771,1763,1763,1696,1696,1607,1607,1602,1602,1575,1575,1488,1488,1469,1469,1453,1453,1446,1446,1392,1392,1374,1374,1301,1301,1298,1298,1296,1296,1292,1292,1284,1284,1265,1265,1206,1206,1199,1199,1164,1164,1145,1145,1128,1128,1126,1126,1013,1013,1007,1007,963,963,939,939,933,933,882,882,867,867,855,855,851,851,843,843,823,823,784,784,777,777,775,775,763,763,752,752,715,715,673,673,602,602,593,593,544,544,537,537,466,466,453,453,450,450,441,441,432,432,385,385,384,384,335,335,334,334,327,327,300,300,296,296,139,139,93,93,81,81,0.0],[496168,496168,496168,298657,298657,297451,297451,279481,279481,241240,241240,225172,225172,224577,224577,197817,197817,195889,195889,188082,188082,183749,183749,183443,183443,182776,182776,179069,179069,178308,178308,176080,176080,172222,172222,167436,167436,142852,142852,141402,141402,140097,140097,138039,138039,134630,134630,134415,134415,123357,123357,123074,123074,122759,122759,122108,122108,121261,121261,120645,120645,112543,112543,109923,109923,99891,99891,99696,99696,99218,99218,98650,98650,97422,97422,97411,97411,96976,96976,96249,96249,95487,95487,95472,95472,93650,93650,90261,90261,89629,89629,89030,89030,88269,88269,87742,87742,87612,87612,86329,86329,85309,85309,84476,84476,83086,83086,80925,80925,79619,79619,77806,77806,76788,76788,76239,76239,74726,74726,73559,73559,73448,73448,68493,68493,66569,66569,65282,65282,64666,64666,64453,64453,63492,63492,63391,63391,60722,60722,60017,60017,57362,57362,57273,57273,57139,57139,56975,56975,56837,56837,55579,55579,52932,52932,52516,52516,51061,51061,50196,50196,49559,49559,48166,48166,46693,46693,45886,45886,45236,45236,44989,44989,44491,44491,42684,42684,42211,42211,41534,41534,41260,41260,39706,39706,39409,39409,38861,38861,38576,38576,38334,38334,37577,37577,36600,36600,36349,36349,35773,35773,33891,33891,32973,32973,32600,32600,32440,32440,31970,31970,31846,31846,31698,31698,30548,30548,29819,29819,29559,29559,28842,28842,28628,28628,27587,27587,26803,26803,26083,26083,25589,25589,25283,25283,24845,24845,24824,24824,24094,24094,23045,23045,22696,22696,22584,22584,22527,22527,22386,22386,21820,21820,21750,21750,21385,21385,20608,20608,19941,19941,19834,19834,18451,18451,18439,18439,18301,18301,18280,18280,17927,17927,17565,17565,17126,17126,15947,15947,15819,15819,15787,15787,15434,15434,15213,15213,14327,14327,14146,14146,12571,12571,12532,12532,12259,12259,12122,12122,12108,12108,11969,11969,11730,11730,11509,11509,11313,11313,11311,11311,11253,11253,11215,11215,10840,10840,10621,10621,10591,10591,10484,10484,9810,9810,9287,9287,9184,9184,8424,8424,8373,8373,7999,7999,7914,7914,7647,7647,7600,7600,7579,7579,7560,7560,7530,7530,7294,7294,7265,7265,7076,7076,7053,7053,6918,6918,6902,6902,6829,6829,6784,6784,6725,6725,6576,6576,6553,6553,6525,6525,6466,6466,6404,6404,6310,6310,6135,6135,6102,6102,6078,6078,6075,6075,6015,6015,6012,6012,5885,5885,5842,5842,5815,5815,5747,5747,5700,5700,5699,5699,5688,5688,5677,5677,5637,5637,5636,5636,5624,5624,5579,5579,5149,5149,5125,5125,5117,5117,5110,5110,5068,5068,5035,5035,5006,5006,4798,4798,4751,4751,4730,4730,4677,4677,4591,4591,4330,4330,4290,4290,4234,4234,4130,4130,4093,4093,3962,3962,3915,3915,3873,3873,3777,3777,3772,3772,3768,3768,3686,3686,3684,3684,3671,3671,3656,3656,3653,3653,3642,3642,3628,3628,3622,3622,3598,3598,3536,3536,3506,3506,3402,3402,3353,3353,3336,3336,3189,3189,3110,3110,3075,3075,3036,3036,3000,3000,2987,2987,2921,2921,2910,2910,2896,2896,2869,2869,2778,2778,2756,2756,2751,2751,2712,2712,2677,2677,2660,2660,2571,2571,2469,2469,2364,2364,2302,2302,2297,2297,2288,2288,2275,2275,2267,2267,2212,2212,2197,2197,2185,2185,2158,2158,2122,2122,2114,2114,2108,2108,2050,2050,2039,2039,2019,2019,2007,2007,1971,1971,1951,1951,1911,1911,1903,1903,1899,1899,1873,1873,1867,1867,1842,1842,1832,1832,1830,1830,1817,1817,1790,1790,1771,1771,1763,1763,1696,1696,1666,1666,1607,1607,1602,1602,1575,1575,1488,1488,1469,1469,1453,1453,1446,1446,1392,1392,1375,1375,1301,1301,1298,1298,1296,1296,1284,1284,1265,1265,1206,1206,1199,1199,1164,1164,1145,1145,1128,1128,1126,1126,1014,1014,1009,1009,1006,1006,963,963,939,939,933,933,882,882,867,867,851,851,843,843,823,823,784,784,777,777,775,775,763,763,752,752,715,715,673,673,602,602,593,593,543,543,537,537,466,466,453,453,450,450,441,441,432,432,385,385,384,384,335,335,327,327,296,296,272,272,139,139,93,93,81,81,0.0],[463425,463425,463425,398851,398851,338391,338391,332324,332324,301517,301517,292350,292350,246733,246733,227461,227461,224722,224722,220213,220213,214459,214459,213626,213626,210417,210417,209010,209010,207418,207418,204069,204069,200214,200214,197645,197645,193132,193132,188153,188153,174212,174212,172754,172754,167821,167821,165375,165375,164929,164929,164600,164600,161170,161170,160277,160277,159682,159682,155203,155203,150611,150611,148863,148863,148271,148271,146725,146725,141009,141009,139208,139208,127637,127637,122556,122556,121065,121065,120119,120119,117028,117028,112048,112048,110213,110213,108902,108902,106007,106007,105961,105961,105638,105638,105475,105475,104641,104641,103290,103290,102138,102138,101615,101615,100205,100205,96102,96102,95966,95966,95703,95703,95388,95388,93113,93113,92812,92812,90739,90739,90421,90421,89907,89907,88238,88238,86583,86583,84899,84899,83940,83940,83531,83531,78946,78946,78128,78128,78116,78116,78018,78018,77277,77277,71473,71473,71025,71025,70196,70196,69550,69550,69202,69202,69168,69168,68782,68782,67537,67537,64542,64542,64233,64233,63068,63068,62386,62386,60194,60194,58474,58474,58318,58318,57959,57959,56791,56791,56658,56658,54499,54499,52513,52513,52392,52392,51451,51451,50260,50260,47836,47836,47185,47185,45579,45579,43581,43581,41449,41449,40363,40363,39598,39598,39197,39197,37844,37844,36181,36181,36143,36143,34256,34256,34211,34211,34086,34086,33897,33897,33506,33506,29775,29775,29501,29501,27909,27909,27473,27473,25723,25723,24834,24834,24575,24575,23801,23801,23520,23520,23502,23502,22505,22505,22313,22313,22299,22299,22289,22289,22212,22212,21623,21623,20854,20854,20792,20792,20132,20132,19629,19629,18696,18696,18670,18670,18370,18370,17657,17657,16953,16953,16719,16719,14164,14164,14089,14089,13416,13416,13110,13110,13065,13065,12248,12248,11782,11782,11135,11135,10918,10918,10808,10808,10802,10802,10459,10459,10383,10383,10260,10260,9846,9846,8285,8285,8118,8118,8100,8100,7579,7579,7571,7571,7532,7532,7455,7455,7394,7394,7311,7311,6757,6757,5892,5892,5891,5891,5814,5814,5691,5691,5637,5637,5319,5319,5108,5108,5073,5073,4844,4844,4818,4818,4702,4702,4477,4477,4460,4460,4438,4438,4333,4333,4163,4163,3966,3966,3854,3854,3741,3741,3628,3628,3607,3607,3576,3576,3495,3495,3401,3401,3310,3310,3132,3132,3026,3026,3006,3006,2832,2832,2773,2773,2567,2567,2565,2565,2546,2546,2519,2519,2508,2508,2505,2505,2469,2469,2467,2467,2383,2383,2372,2372,2370,2370,2365,2365,2302,2302,2280,2280,2159,2159,2110,2110,2045,2045,2001,2001,1995,1995,1924,1924,1888,1888,1838,1838,1705,1705,1599,1599,1510,1510,1477,1477,1460,1460,1275,1275,1146,1146,1123,1123,1105,1105,1098,1098,1086,1086,1081,1081,1059,1059,1026,1026,1023,1023,1020,1020,997,997,995,995,944,944,939,939,816,816,788,788,785,785,782,782,678,678,673,673,649,649,630,630,613,613,606,606,596,596,575,575,558,558,532,532,478,478,471,471,469,469,452,452,436,436,420,420,410,410,401,401,395,395,389,389,385,385,342,342,334,334,314,314,306,306,296,296,292,292,284,284,281,281,259,259,231,231,214,214,195,195,192,192,162,162,127,127,101,101,86,86,0.0],[376128,376128,376128,374526,374526,353537,353537,346593,346593,332365,332365,312954,312954,312136,312136,309713,309713,305702,305702,285818,285818,281096,281096,280796,280796,259217,259217,256866,256866,252256,252256,246167,246167,243225,243225,213853,213853,209333,209333,207104,207104,206464,206464,206426,206426,198136,198136,195429,195429,193500,193500,185008,185008,178486,178486,167071,167071,165577,165577,165147,165147,162082,162082,157049,157049,156159,156159,152613,152613,148922,148922,143744,143744,133782,133782,128971,128971,128026,128026,127317,127317,126434,126434,122873,122873,120421,120421,119931,119931,115932,115932,112519,112519,112226,112226,110488,110488,108360,108360,103403,103403,101643,101643,97569,97569,96415,96415,96257,96257,95326,95326,95155,95155,94329,94329,91739,91739,89894,89894,78070,78070,75256,75256,73213,73213,73024,73024,71879,71879,71771,71771,71031,71031,69485,69485,69372,69372,68934,68934,68497,68497,67090,67090,66119,66119,64699,64699,63737,63737,62149,62149,62081,62081,61687,61687,58209,58209,57813,57813,54942,54942,54009,54009,51000,51000,49180,49180,47977,47977,47729,47729,47553,47553,46612,46612,45743,45743,45597,45597,44960,44960,43874,43874,42651,42651,41455,41455,41155,41155,39834,39834,39469,39469,38943,38943,33809,33809,33155,33155,32898,32898,32781,32781,32348,32348,32201,32201,31810,31810,30878,30878,30620,30620,30126,30126,29969,29969,29264,29264,28903,28903,28838,28838,28591,28591,28323,28323,28124,28124,26633,26633,26197,26197,25494,25494,25312,25312,24799,24799,24768,24768,23673,23673,23667,23667,23207,23207,22707,22707,21438,21438,19851,19851,17371,17371,17264,17264,16935,16935,15528,15528,15000,15000,14935,14935,13821,13821,11920,11920,11275,11275,11118,11118,10925,10925,10816,10816,10152,10152,9921,9921,9385,9385,9087,9087,9049,9049,8114,8114,7581,7581,7417,7417,7310,7310,6988,6988,6775,6775,6598,6598,6568,6568,6226,6226,6082,6082,6011,6011,6010,6010,5991,5991,5988,5988,5986,5986,5926,5926,5828,5828,5758,5758,5757,5757,5750,5750,5727,5727,5720,5720,5712,5712,5509,5509,5347,5347,5106,5106,4892,4892,4879,4879,4809,4809,4574,4574,4462,4462,4434,4434,4226,4226,4175,4175,4111,4111,4013,4013,3973,3973,3885,3885,3851,3851,3667,3667,3599,3599,3575,3575,3518,3518,3421,3421,3310,3310,3284,3284,3255,3255,3176,3176,3078,3078,2958,2958,2660,2660,2636,2636,2590,2590,2486,2486,2459,2459,2395,2395,2364,2364,2357,2357,2223,2223,2174,2174,2160,2160,2156,2156,2060,2060,1803,1803,1779,1779,1619,1619,1574,1574,1560,1560,1476,1476,1353,1353,1315,1315,1263,1263,1246,1246,1226,1226,1218,1218,1144,1144,1113,1113,1093,1093,1062,1062,1000,1000,989,989,939,939,937,937,934,934,924,924,915,915,900,900,849,849,839,839,797,797,742,742,678,678,665,665,659,659,651,651,635,635,578,578,564,564,526,526,524,524,509,509,497,497,441,441,409,409,401,401,388,388,383,383,377,377,370,370,363,363,362,362,352,352,320,320,311,311,308,308,282,282,276,276,272,272,268,268,137,137,0.0],[375904,375904,375904,314804,314804,305908,305908,270367,270367,222498,222498,213798,213798,195505,195505,195320,195320,184708,184708,184668,184668,174249,174249,163521,163521,161133,161133,160844,160844,156155,156155,154799,154799,148585,148585,148021,148021,138908,138908,136217,136217,135254,135254,134625,134625,133532,133532,130133,130133,123926,123926,122917,122917,122879,122879,122730,122730,121398,121398,121034,121034,120071,120071,116965,116965,116118,116118,115622,115622,115534,115534,113458,113458,111656,111656,110559,110559,109555,109555,109379,109379,107370,107370,107310,107310,107096,107096,106058,106058,105975,105975,103903,103903,103651,103651,103115,103115,101931,101931,99960,99960,96915,96915,94557,94557,92818,92818,91717,91717,91099,91099,90271,90271,90032,90032,88701,88701,88257,88257,86351,86351,85633,85633,84163,84163,82135,82135,81017,81017,80375,80375,79086,79086,76995,76995,75179,75179,74728,74728,74580,74580,73426,73426,73181,73181,73089,73089,70103,70103,69183,69183,69134,69134,69089,69089,68885,68885,68858,68858,67970,67970,67439,67439,66448,66448,66423,66423,65990,65990,65561,65561,64977,64977,63443,63443,62256,62256,59452,59452,58887,58887,58763,58763,58377,58377,58235,58235,56714,56714,56620,56620,56386,56386,56022,56022,55591,55591,54680,54680,51715,51715,51594,51594,51244,51244,50862,50862,50334,50334,48331,48331,47857,47857,46542,46542,46223,46223,44400,44400,44055,44055,43645,43645,43336,43336,43039,43039,41819,41819,41549,41549,40279,40279,39955,39955,39508,39508,39505,39505,39453,39453,38497,38497,38321,38321,37663,37663,37439,37439,35518,35518,34992,34992,34669,34669,34579,34579,34204,34204,34165,34165,34000,34000,33898,33898,32933,32933,32884,32884,32835,32835,32763,32763,32602,32602,32176,32176,32062,32062,30930,30930,30478,30478,29897,29897,29580,29580,28979,28979,28750,28750,28739,28739,28584,28584,27511,27511,27464,27464,27452,27452,27206,27206,26165,26165,26006,26006,25735,25735,25336,25336,25201,25201,25024,25024,24851,24851,24840,24840,24711,24711,24292,24292,23744,23744,23096,23096,22784,22784,22704,22704,22656,22656,22526,22526,22501,22501,22477,22477,22212,22212,21482,21482,21168,21168,20960,20960,20834,20834,20333,20333,20272,20272,19737,19737,19652,19652,19334,19334,19308,19308,19235,19235,18905,18905,18727,18727,18704,18704,18666,18666,18493,18493,18259,18259,17629,17629,17035,17035,17019,17019,16427,16427,15520,15520,15332,15332,15083,15083,14663,14663,14616,14616,13961,13961,13917,13917,13680,13680,12971,12971,12684,12684,12577,12577,12554,12554,12495,12495,12373,12373,12114,12114,12035,12035,11661,11661,11609,11609,11532,11532,11273,11273,11203,11203,11182,11182,11166,11166,11024,11024,10979,10979,10883,10883,10823,10823,10745,10745,10594,10594,10377,10377,9765,9765,9758,9758,9744,9744,9409,9409,9229,9229,9214,9214,9188,9188,9115,9115,8905,8905,8705,8705,8474,8474,8471,8471,8385,8385,8144,8144,7841,7841,7802,7802,7610,7610,7578,7578,7540,7540,7488,7488,7214,7214,7072,7072,6991,6991,6919,6919,6793,6793,6644,6644,6408,6408,6249,6249,6097,6097,5950,5950,5948,5948,5665,5665,5439,5439,5392,5392,5128,5128,5110,5110,4999,4999,4952,4952,4929,4929,4788,4788,4691,4691,4491,4491,4489,4489,4234,4234,4159,4159,4052,4052,4013,4013,3885,3885,3838,3838,3742,3742,3695,3695,3673,3673,3621,3621,3617,3617,3571,3571,3508,3508,3404,3404,3386,3386,3309,3309,3249,3249,3224,3224,2817,2817,2705,2705,2582,2582,2536,2536,2444,2444,2364,2364,2273,2273,2167,2167,2160,2160,2134,2134,2073,2073,2072,2072,2001,2001,1925,1925,1924,1924,1840,1840,1698,1698,1642,1642,1578,1578,1545,1545,1532,1532,1523,1523,1478,1478,1470,1470,1460,1460,1352,1352,1350,1350,1321,1321,1292,1292,1217,1217,1200,1200,1180,1180,1177,1177,1156,1156,1145,1145,1101,1101,1091,1091,1028,1028,977,977,924,924,883,883,882,882,754,754,635,635,617,617,591,591,571,571,565,565,559,559,548,548,546,546,539,539,537,537,530,530,525,525,504,504,494,494,481,481,466,466,451,451,430,430,413,413,412,412,407,407,405,405,381,381,379,379,377,377,373,373,362,362,342,342,339,339,338,338,320,320,316,316,312,312,305,305,294,294,287,287,276,276,269,269,263,263,260,260,215,215,133,133,0.0],[444906,444906,444906,410903,410903,378180,378180,374002,374002,361550,361550,322912,322912,305123,305123,297107,297107,295389,295389,293074,293074,286167,286167,281001,281001,278698,278698,277774,277774,272716,272716,271688,271688,269390,269390,231372,231372,225909,225909,222106,222106,207950,207950,207188,207188,206438,206438,200795,200795,198559,198559,195400,195400,193319,193319,189523,189523,186244,186244,184883,184883,181562,181562,174010,174010,166401,166401,160225,160225,157123,157123,156198,156198,152594,152594,152565,152565,148991,148991,134183,134183,130183,130183,127979,127979,125982,125982,123869,123869,122868,122868,119652,119652,117686,117686,116498,116498,114199,114199,112276,112276,111187,111187,109451,109451,105599,105599,103627,103627,102546,102546,97694,97694,96446,96446,93778,93778,90278,90278,90117,90117,79309,79309,77045,77045,74811,74811,74619,74619,70641,70641,69452,69452,69302,69302,68925,68925,67469,67469,64932,64932,63697,63697,62173,62173,57112,57112,55433,55433,51000,51000,50736,50736,48389,48389,48010,48010,46530,46530,44980,44980,44030,44030,43327,43327,43015,43015,36193,36193,35717,35717,33862,33862,32924,32924,29892,29892,29724,29724,29719,29719,29548,29548,29388,29388,29260,29260,28890,28890,28863,28863,27864,27864,26174,26174,25227,25227,24933,24933,24797,24797,24728,24728,24695,24695,24429,24429,23689,23689,23125,23125,19552,19552,18901,18901,17766,17766,17343,17343,16624,16624,16537,16537,15349,15349,15239,15239,14998,14998,14914,14914,14331,14331,13264,13264,13260,13260,12152,12152,11975,11975,11904,11904,11690,11690,11301,11301,10976,10976,10462,10462,10370,10370,10230,10230,10191,10191,9774,9774,9613,9613,9258,9258,9230,9230,8958,8958,8789,8789,8484,8484,7943,7943,7899,7899,7851,7851,7582,7582,7405,7405,6973,6973,6817,6817,6771,6771,6700,6700,6695,6695,6526,6526,6472,6472,6239,6239,6061,6061,5970,5970,5969,5969,5954,5954,5822,5822,5816,5816,5637,5637,5520,5520,5137,5137,4815,4815,4783,4783,4741,4741,4692,4692,4539,4539,4535,4535,4505,4505,4226,4226,4013,4013,3998,3998,3742,3742,3697,3697,3654,3654,3604,3604,3408,3408,3310,3310,3264,3264,3070,3070,3041,3041,2916,2916,2660,2660,2633,2633,2477,2477,2444,2444,2373,2373,2372,2372,2364,2364,2250,2250,2241,2241,2195,2195,2169,2169,2168,2168,2167,2167,2163,2163,2160,2160,2142,2142,2140,2140,1868,1868,1530,1530,1491,1491,1468,1468,1451,1451,1411,1411,1315,1315,1311,1311,1218,1218,1200,1200,1185,1185,1124,1124,1050,1050,1012,1012,937,937,929,929,854,854,847,847,844,844,840,840,817,817,792,792,733,733,691,691,671,671,654,654,652,652,634,634,567,567,562,562,551,551,526,526,515,515,514,514,496,496,494,494,445,445,438,438,412,412,398,398,374,374,362,362,356,356,323,323,320,320,316,316,306,306,293,293,272,272,266,266,258,258,215,215,207,207,174,174,170,170,130,130,86,86,0.0],[823228,823228,823228,581281,581281,569226,569226,515822,515822,482312,482312,469269,469269,428104,428104,412535,412535,408682,408682,342670,342670,332483,332483,311787,311787,304949,304949,285229,285229,281793,281793,280871,280871,280759,280759,273017,273017,265303,265303,242185,242185,236639,236639,214154,214154,212069,212069,209794,209794,209032,209032,200485,200485,187724,187724,186562,186562,185548,185548,185049,185049,181474,181474,180888,180888,172853,172853,156182,156182,156169,156169,152515,152515,149917,149917,144410,144410,144092,144092,135424,135424,126810,126810,125863,125863,125559,125559,123819,123819,119680,119680,117331,117331,110545,110545,104539,104539,98052,98052,97610,97610,93937,93937,88455,88455,87454,87454,77053,77053,75977,75977,72603,72603,71664,71664,69038,69038,68690,68690,63637,63637,60394,60394,53885,53885,52653,52653,47331,47331,45721,45721,44596,44596,43337,43337,42626,42626,41731,41731,33818,33818,32901,32901,32310,32310,29368,29368,26542,26542,25766,25766,25089,25089,24846,24846,24808,24808,24400,24400,23726,23726,23547,23547,21816,21816,21466,21466,20551,20551,20547,20547,20097,20097,19403,19403,18894,18894,17937,17937,17682,17682,16669,16669,15834,15834,14671,14671,12702,12702,11587,11587,11518,11518,11266,11266,11186,11186,10768,10768,10762,10762,9854,9854,9211,9211,8377,8377,8239,8239,7431,7431,6986,6986,6920,6920,6778,6778,6460,6460,6092,6092,6058,6058,5699,5699,5687,5687,5402,5402,5353,5353,4669,4669,4435,4435,4014,4014,3936,3936,3706,3706,3524,3524,3459,3459,3398,3398,3221,3221,2938,2938,2700,2700,2697,2697,2600,2600,2556,2556,2530,2530,2395,2395,2218,2218,2208,2208,2061,2061,1952,1952,1905,1905,1845,1845,1755,1755,1746,1746,1596,1596,1589,1589,1588,1588,1317,1317,1292,1292,1259,1259,1241,1241,1105,1105,1040,1040,1032,1032,1024,1024,999,999,990,990,986,986,966,966,943,943,861,861,852,852,759,759,750,750,748,748,714,714,709,709,694,694,649,649,635,635,578,578,575,575,554,554,552,552,441,441,437,437,405,405,394,394,385,385,366,366,364,364,338,338,336,336,335,335,320,320,316,316,314,314,312,312,298,298,285,285,280,280,276,276,274,274,269,269,263,263,179,179,169,169,154,154,147,147,136,136,100,100,94,94,0.0],[1122352,1122352,1122352,1073679,1073679,1057916,1057916,1016583,1016583,989914,989914,935310,935310,866487,866487,854688,854688,793998,793998,712735,712735,652338,652338,610574,610574,480526,480526,425759,425759,421761,421761,410723,410723,322360,322360,315682,315682,306848,306848,296126,296126,294480,294480,219031,219031,149985,149985,149129,149129,97285,97285,91570,91570,86831,86831,58207,58207,39436,39436,38556,38556,33449,33449,32899,32899,28871,28871,24923,24923,11329,11329,10742,10742,10527,10527,4013,4013,2857,2857,2680,2680,2366,2366,2022,2022,680,680,608,608,597,597,441,441,340,340,0.0],[952289,952289,952289,894972,894972,787358,787358,627317,627317,562291,562291,501673,501673,482416,482416,342410,342410,338617,338617,314256,314256,292867,292867,273072,273072,253647,253647,252840,252840,240279,240279,226842,226842,200168,200168,195828,195828,185060,185060,181042,181042,177316,177316,174437,174437,172746,172746,170514,170514,170176,170176,169256,169256,165959,165959,156271,156271,154021,154021,150781,150781,150534,150534,141077,141077,138283,138283,135470,135470,134669,134669,119512,119512,118921,118921,113894,113894,112907,112907,109907,109907,106890,106890,105518,105518,104005,104005,103503,103503,102831,102831,99119,99119,95918,95918,94966,94966,94545,94545,88390,88390,88125,88125,84158,84158,78443,78443,78107,78107,76540,76540,72825,72825,71539,71539,70655,70655,68900,68900,65887,65887,65604,65604,64265,64265,59545,59545,58686,58686,56564,56564,55088,55088,53890,53890,44621,44621,44556,44556,43280,43280,41980,41980,38162,38162,37550,37550,36062,36062,33731,33731,32899,32899,31985,31985,31297,31297,30775,30775,29718,29718,28875,28875,27400,27400,25575,25575,24834,24834,24403,24403,22438,22438,22066,22066,21547,21547,21457,21457,19780,19780,19406,19406,18538,18538,15043,15043,14950,14950,13694,13694,12857,12857,11377,11377,11292,11292,10218,10218,9387,9387,8691,8691,8238,8238,8126,8126,8116,8116,7868,7868,7103,7103,6982,6982,6848,6848,6609,6609,6468,6468,6128,6128,6091,6091,6012,6012,5535,5535,5315,5315,5095,5095,4845,4845,4821,4821,4741,4741,4483,4483,4024,4024,4013,4013,3916,3916,3743,3743,3699,3699,3686,3686,3628,3628,3625,3625,3547,3547,3374,3374,3068,3068,3067,3067,2909,2909,2849,2849,2778,2778,2769,2769,2766,2766,2765,2765,2756,2756,2713,2713,2679,2679,2514,2514,2469,2469,2423,2423,2299,2299,2288,2288,2266,2266,2242,2242,2164,2164,2158,2158,2118,2118,2062,2062,2040,2040,2039,2039,1893,1893,1790,1790,1779,1779,1763,1763,1636,1636,1583,1583,1499,1499,1469,1469,1414,1414,1408,1408,1345,1345,1342,1342,1301,1301,1268,1268,1258,1258,1256,1256,1254,1254,1222,1222,1218,1218,1145,1145,1142,1142,1105,1105,1100,1100,1090,1090,1073,1073,1043,1043,1000,1000,930,930,927,927,842,842,815,815,797,797,734,734,716,716,707,707,671,671,667,667,658,658,638,638,635,635,599,599,593,593,589,589,573,573,441,441,419,419,413,413,412,412,401,401,394,394,388,388,378,378,333,333,325,325,323,323,319,319,317,317,305,305,290,290,288,288,282,282,275,275,273,273,269,269,263,263,243,243,222,222,214,214,212,212,152,152,146,146,141,141,127,127,74,74,0.0],[952459,952459,952459,895898,895898,787438,787438,727865,727865,571809,571809,561500,561500,545472,545472,433207,433207,315434,315434,292866,292866,284602,284602,283490,283490,278074,278074,277580,277580,273094,273094,250326,250326,246564,246564,240257,240257,227090,227090,214791,214791,204577,204577,202921,202921,195832,195832,185018,185018,180706,180706,174714,174714,172745,172745,169848,169848,168230,168230,158795,158795,156275,156275,152636,152636,150787,150787,150528,150528,143860,143860,138401,138401,135457,135457,113375,113375,110127,110127,106890,106890,105513,105513,104002,104002,103324,103324,103259,103259,102811,102811,97817,97817,93915,93915,88392,88392,88126,88126,87079,87079,78398,78398,77753,77753,76575,76575,72836,72836,72245,72245,69271,69271,69032,69032,68149,68149,65614,65614,61633,61633,59945,59945,59859,59859,56488,56488,55470,55470,55088,55088,53886,53886,41973,41973,41382,41382,36716,36716,33898,33898,32898,32898,31321,31321,30780,30780,30067,30067,28892,28892,27406,27406,27140,27140,26733,26733,24735,24735,23561,23561,21457,21457,19422,19422,18887,18887,18551,18551,18399,18399,15064,15064,13695,13695,12857,12857,12703,12703,11683,11683,11377,11377,11292,11292,9400,9400,9307,9307,8691,8691,8129,8129,7103,7103,6982,6982,6848,6848,6620,6620,6459,6459,6134,6134,6091,6091,6077,6077,5318,5318,5095,5095,4842,4842,4821,4821,4722,4722,3999,3999,3916,3916,3699,3699,3628,3628,3546,3546,3375,3375,3303,3303,3071,3071,3066,3066,2768,2768,2767,2767,2765,2765,2514,2514,2494,2494,2423,2423,2377,2377,2266,2266,2203,2203,2160,2160,2119,2119,2062,2062,2040,2040,1600,1600,1583,1583,1525,1525,1486,1486,1268,1268,1259,1259,1256,1256,1218,1218,1145,1145,1142,1142,1105,1105,1073,1073,1070,1070,1044,1044,1000,1000,998,998,994,994,927,927,852,852,822,822,729,729,707,707,671,671,655,655,638,638,629,629,622,622,615,615,602,602,589,589,554,554,518,518,513,513,442,442,429,429,427,427,397,397,394,394,388,388,371,371,370,370,363,363,347,347,340,340,336,336,333,333,326,326,323,323,321,321,319,319,317,317,316,316,311,311,309,309,298,298,288,288,282,282,279,279,268,268,257,257,243,243,212,212,197,197,187,187,178,178,166,166,158,158,157,157,147,147,0.0],[951914,951914,951914,887802,887802,787933,787933,712590,712590,570658,570658,545188,545188,481030,481030,450904,450904,342510,342510,315586,315586,305478,305478,280871,280871,278557,278557,277003,277003,273326,273326,255519,255519,251309,251309,250422,250422,248004,248004,244297,244297,240251,240251,237066,237066,209027,209027,195720,195720,193983,193983,185220,185220,183596,183596,180666,180666,177314,177314,174582,174582,173075,173075,154420,154420,152655,152655,150835,150835,144210,144210,140249,140249,135562,135562,135273,135273,119513,119513,113948,113948,111009,111009,106889,106889,98056,98056,97644,97644,96045,96045,88434,88434,88081,88081,85413,85413,81995,81995,78516,78516,78406,78406,76742,76742,69204,69204,69008,69008,67708,67708,65635,65635,60481,60481,59948,59948,55205,55205,53890,53890,44620,44620,41973,41973,39436,39436,38926,38926,37512,37512,33894,33894,33140,33140,32901,32901,30062,30062,28870,28870,28240,28240,27982,27982,27407,27407,27333,27333,27147,27147,25052,25052,23590,23590,23538,23538,21458,21458,19404,19404,18783,18783,18542,18542,17665,17665,15513,15513,15061,15061,13168,13168,12175,12175,11377,11377,11292,11292,10653,10653,8688,8688,8127,8127,7659,7659,7104,7104,7067,7067,6990,6990,6982,6982,6774,6774,6722,6722,6616,6616,6134,6134,6091,6091,6087,6087,5308,5308,5095,5095,4845,4845,4821,4821,4741,4741,4732,4732,4013,4013,3916,3916,3721,3721,3699,3699,3628,3628,3612,3612,3547,3547,3304,3304,3067,3067,3066,3066,2857,2857,2712,2712,2514,2514,2378,2378,2203,2203,2173,2173,2151,2151,2040,2040,1951,1951,1596,1596,1588,1588,1583,1583,1263,1263,1260,1260,1256,1256,1253,1253,1251,1251,1241,1241,1218,1218,1201,1201,1105,1105,1091,1091,1083,1083,1073,1073,1044,1044,999,999,986,986,966,966,927,927,843,843,644,644,614,614,587,587,578,578,548,548,543,543,535,535,527,527,447,447,412,412,399,399,394,394,364,364,338,338,333,333,325,325,322,322,320,320,319,319,317,317,312,312,305,305,298,298,288,288,283,283,276,276,275,275,269,269,263,263,183,183,133,133,0.0],[788209,788209,788209,775092,775092,571767,571767,569053,569053,561359,561359,545465,545465,478233,478233,478058,478058,389963,389963,338381,338381,311763,311763,305422,305422,302715,302715,297317,297317,292889,292889,282943,282943,273107,273107,261484,261484,257401,257401,240283,240283,227114,227114,209035,209035,184962,184962,177155,177155,172745,172745,171813,171813,171755,171755,170616,170616,168943,168943,161072,161072,158890,158890,158726,158726,156263,156263,150792,150792,150547,150547,147925,147925,143871,143871,135457,135457,126159,126159,119632,119632,118986,118986,110127,110127,109174,109174,105518,105518,104041,104041,97948,97948,97725,97725,93945,93945,88391,88391,82716,82716,78438,78438,77796,77796,76725,76725,75840,75840,72813,72813,72235,72235,69204,69204,68102,68102,65617,65617,65460,65460,65393,65393,63247,63247,60978,60978,56542,56542,55124,55124,53888,53888,48582,48582,43035,43035,41971,41971,41384,41384,39498,39498,37550,37550,33894,33894,33731,33731,32898,32898,31295,31295,30775,30775,29162,29162,28875,28875,27400,27400,27160,27160,24731,24731,23560,23560,22438,22438,21461,21461,19632,19632,19422,19422,15056,15056,12857,12857,12175,12175,11376,11376,11265,11265,10845,10845,10328,10328,10190,10190,8688,8688,8381,8381,7814,7814,7579,7579,7519,7519,7103,7103,7066,7066,6998,6998,6982,6982,6897,6897,6848,6848,6722,6722,6609,6609,6473,6473,6129,6129,6098,6098,5955,5955,5315,5315,5095,5095,4845,4845,4821,4821,4741,4741,4237,4237,4011,4011,3916,3916,3811,3811,3699,3699,3628,3628,3624,3624,3547,3547,3068,3068,3066,3066,2767,2767,2514,2514,2423,2423,2364,2364,2316,2316,2266,2266,2232,2232,2229,2229,2203,2203,2160,2160,2119,2119,2062,2062,2040,2040,1685,1685,1594,1594,1583,1583,1498,1498,1314,1314,1268,1268,1266,1266,1259,1259,1258,1258,1218,1218,1145,1145,1142,1142,1105,1105,1090,1090,1073,1073,1004,1004,998,998,927,927,847,847,842,842,807,807,778,778,748,748,735,735,734,734,733,733,707,707,638,638,587,587,549,549,524,524,518,518,513,513,441,441,434,434,403,403,399,399,394,394,388,388,371,371,352,352,341,341,335,335,333,333,329,329,319,319,317,317,288,288,276,276,275,275,236,236,175,175,171,171,162,162,133,133,77,77,0.0],[951813,951813,951813,788478,788478,569064,569064,561880,561880,482248,482248,445816,445816,426470,426470,424362,424362,414941,414941,402158,402158,388033,388033,326128,326128,311489,311489,295663,295663,294495,294495,284613,284613,273222,273222,255931,255931,244932,244932,240417,240417,240300,240300,236608,236608,229333,229333,185070,185070,177345,177345,175051,175051,170746,170746,161342,161342,155499,155499,154498,154498,152654,152654,152625,152625,150529,150529,149394,149394,138255,138255,136285,136285,135558,135558,125416,125416,106863,106863,104979,104979,100006,100006,99585,99585,95341,95341,92214,92214,91965,91965,88485,88485,88154,88154,81820,81820,78437,78437,78330,78330,74770,74770,72821,72821,72562,72562,72368,72368,71643,71643,71339,71339,70644,70644,69250,69250,68177,68177,65656,65656,61689,61689,60119,60119,56538,56538,47370,47370,45501,45501,42000,42000,38116,38116,35952,35952,35097,35097,33868,33868,32901,32901,32082,32082,30069,30069,28872,28872,28177,28177,27408,27408,27232,27232,27152,27152,24409,24409,23597,23597,19637,19637,19216,19216,16114,16114,15065,15065,14949,14949,12169,12169,11430,11430,11346,11346,11316,11316,10939,10939,10878,10878,10653,10653,9921,9921,9648,9648,8327,8327,8249,8249,8127,8127,7837,7837,7578,7578,7067,7067,6993,6993,6920,6920,6775,6775,6713,6713,6620,6620,6560,6560,6461,6461,6089,6089,6012,6012,5535,5535,5315,5315,4845,4845,4809,4809,4732,4732,4013,4013,3916,3916,3799,3799,3742,3742,3686,3686,3628,3628,3624,3624,3547,3547,3066,3066,2909,2909,2857,2857,2849,2849,2778,2778,2756,2756,2713,2713,2709,2709,2679,2679,2600,2600,2533,2533,2514,2514,2469,2469,2423,2423,2378,2378,2364,2364,2288,2288,2243,2243,2203,2203,2200,2200,2160,2160,2158,2158,2133,2133,2040,2040,2039,2039,1893,1893,1871,1871,1846,1846,1790,1790,1779,1779,1763,1763,1602,1602,1583,1583,1525,1525,1469,1469,1447,1447,1303,1303,1301,1301,1284,1284,1259,1259,1250,1250,1226,1226,1218,1218,1124,1124,1106,1106,1105,1105,1073,1073,999,999,992,992,930,930,878,878,819,819,808,808,805,805,716,716,707,707,685,685,640,640,638,638,628,628,627,627,593,593,589,589,564,564,539,539,524,524,516,516,490,490,441,441,426,426,422,422,394,394,388,388,382,382,351,351,340,340,335,335,333,333,321,321,316,316,303,303,290,290,283,283,276,276,270,270,269,269,268,268,252,252,229,229,214,214,187,187,160,160,127,127,0.0],[953080,953080,953080,886574,886574,769755,769755,561313,561313,526421,526421,482479,482479,440581,440581,402151,402151,315412,315412,305243,305243,294649,294649,294466,294466,278576,278576,276868,276868,256940,256940,256227,256227,250373,250373,240614,240614,240319,240319,237201,237201,226025,226025,209033,209033,204335,204335,194090,194090,186874,186874,185058,185058,183249,183249,177361,177361,168422,168422,164332,164332,161353,161353,152626,152626,150749,150749,150530,150530,148185,148185,145537,145537,143940,143940,139327,139327,135562,135562,133271,133271,122095,122095,119512,119512,106554,106554,105487,105487,97969,97969,95942,95942,88985,88985,88419,88419,88355,88355,85182,85182,84141,84141,82721,82721,81817,81817,79793,79793,79257,79257,78403,78403,73439,73439,72821,72821,72442,72442,65652,65652,60486,60486,59985,59985,59962,59962,59929,59929,55376,55376,50434,50434,41982,41982,39494,39494,38439,38439,33893,33893,33143,33143,32898,32898,30775,30775,28891,28891,28885,28885,27404,27404,27232,27232,27160,27160,26504,26504,24814,24814,24735,24735,23610,23610,20412,20412,19618,19618,19404,19404,18890,18890,17668,17668,16705,16705,15064,15064,14976,14976,14157,14157,12102,12102,11377,11377,11292,11292,10653,10653,10219,10219,8239,8239,8204,8204,8127,8127,7814,7814,7579,7579,7104,7104,7069,7069,6994,6994,6774,6774,6584,6584,6304,6304,6251,6251,6134,6134,6089,6089,5103,5103,4993,4993,4845,4845,4821,4821,4732,4732,4722,4722,4491,4491,4013,4013,3916,3916,3721,3721,3699,3699,3659,3659,3628,3628,3612,3612,3547,3547,3303,3303,3075,3075,2514,2514,2494,2494,2365,2365,2260,2260,2160,2160,2049,2049,2040,2040,2023,2023,1951,1951,1592,1592,1583,1583,1526,1526,1486,1486,1260,1260,1259,1259,1252,1252,1218,1218,1201,1201,1145,1145,1105,1105,1100,1100,1091,1091,1073,1073,1062,1062,1044,1044,1004,1004,866,866,770,770,684,684,644,644,635,635,629,629,608,608,589,589,587,587,572,572,535,535,513,513,445,445,441,441,439,439,437,437,426,426,401,401,398,398,394,394,388,388,382,382,333,333,321,321,320,320,318,318,317,317,316,316,304,304,300,300,288,288,283,283,274,274,267,267,220,220,193,193,133,133,127,127,100,100,99,99,86,86,85,85,69,69,0.0],[895946,895946,895946,788639,788639,775805,775805,725339,725339,514745,514745,478132,478132,426564,426564,425508,425508,389426,389426,315389,315389,305421,305421,302714,302714,292890,292890,273038,273038,261501,261501,257399,257399,250220,250220,244712,244712,244708,244708,240283,240283,227176,227176,185025,185025,174692,174692,172741,172741,170621,170621,169883,169883,161068,161068,158890,158890,158792,158792,152619,152619,150790,150790,150527,150527,135562,135562,119517,119517,113956,113956,112941,112941,111915,111915,110127,110127,106888,106888,105529,105529,104524,104524,102770,102770,102627,102627,97810,97810,95966,95966,94965,94965,88443,88443,82711,82711,78395,78395,77829,77829,76752,76752,74562,74562,72308,72308,71296,71296,69211,69211,69198,69198,65886,65886,65617,65617,64540,64540,61199,61199,60010,60010,59545,59545,56564,56564,54924,54924,46113,46113,44556,44556,43230,43230,41980,41980,41382,41382,39497,39497,38982,38982,38162,38162,36661,36661,33894,33894,32898,32898,32003,32003,31985,31985,30936,30936,29718,29718,28892,28892,27400,27400,27369,27369,25575,25575,25055,25055,24735,24735,21461,21461,19637,19637,19185,19185,18551,18551,17940,17940,15043,15043,14958,14958,12857,12857,12175,12175,11377,11377,11292,11292,10218,10218,8238,8238,8127,8127,8122,8122,7814,7814,7574,7574,7100,7100,6920,6920,6848,6848,6722,6722,6609,6609,6460,6460,6128,6128,6059,6059,6012,6012,5340,5340,5315,5315,5095,5095,4845,4845,4821,4821,4741,4741,4427,4427,4227,4227,3999,3999,3916,3916,3749,3749,3686,3686,3628,3628,3547,3547,3454,3454,3071,3071,3066,3066,2909,2909,2858,2858,2849,2849,2778,2778,2756,2756,2713,2713,2679,2679,2514,2514,2469,2469,2423,2423,2364,2364,2299,2299,2288,2288,2266,2266,2242,2242,2203,2203,2187,2187,2160,2160,2158,2158,2062,2062,2040,2040,2039,2039,1893,1893,1848,1848,1790,1790,1779,1779,1763,1763,1599,1599,1583,1583,1499,1499,1469,1469,1345,1345,1342,1342,1301,1301,1266,1266,1264,1264,1259,1259,1254,1254,1252,1252,1223,1223,1218,1218,1176,1176,1145,1145,1105,1105,1073,1073,1044,1044,1006,1006,1004,1004,930,930,861,861,842,842,748,748,735,735,716,716,707,707,675,675,649,649,638,638,593,593,589,589,559,559,524,524,513,513,456,456,441,441,426,426,421,421,405,405,394,394,390,390,388,388,385,385,381,381,374,374,371,371,343,343,336,336,333,333,330,330,319,319,317,317,316,316,305,305,299,299,289,289,288,288,280,280,275,275,274,274,273,273,265,265,233,233,183,183,180,180,179,179,169,169,154,154,136,136,94,94,0.0],[8819,8819,8819,6817,6817,6250,6250,4540,4540,3950,3950,2517,2517,2512,2512,2507,2507,2367,2367,2322,2322,2024,2024,1445,1445,1414,1414,1387,1387,1236,1236,1230,1230,1180,1180,1163,1163,1145,1145,1116,1116,1107,1107,1089,1089,1082,1082,1081,1081,1056,1056,1042,1042,1013,1013,988,988,943,943,941,941,926,926,911,911,858,858,796,796,770,770,760,760,757,757,756,756,740,740,721,721,694,694,690,690,673,673,664,664,656,656,652,652,613,613,592,592,575,575,570,570,540,540,535,535,467,467,455,455,431,431,427,427,425,425,421,421,420,420,410,410,385,385,377,377,376,376,374,374,369,369,351,351,343,343,342,342,339,339,337,337,330,330,328,328,325,325,317,317,308,308,297,297,281,281,278,278,276,276,274,274,255,255,248,248,236,236,234,234,231,231,193,193,192,192,153,153,141,141,140,140,135,135,134,134,128,128,125,125,124,124,123,123,122,122,115,115,113,113,112,112,104,104,100,100,92,92,91,91,88,88,85,85,84,84,81,81,80,80,77,77,76,76,75,75,72,72,71,71,0.0],[8819,8819,8819,6817,6817,6250,6250,4540,4540,3951,3951,2517,2517,2512,2512,2507,2507,2367,2367,2322,2322,2024,2024,1445,1445,1414,1414,1387,1387,1236,1236,1230,1230,1180,1180,1163,1163,1145,1145,1116,1116,1088,1088,1082,1082,1081,1081,1042,1042,1014,1014,988,988,943,943,941,941,926,926,911,911,858,858,796,796,770,770,760,760,757,757,756,756,740,740,721,721,694,694,690,690,673,673,664,664,656,656,652,652,613,613,592,592,575,575,570,570,540,540,467,467,455,455,431,431,427,427,425,425,421,421,420,420,410,410,385,385,377,377,376,376,374,374,369,369,351,351,343,343,342,342,339,339,337,337,328,328,325,325,317,317,308,308,297,297,281,281,278,278,276,276,274,274,255,255,248,248,236,236,234,234,231,231,220,220,193,193,192,192,171,171,153,153,140,140,135,135,128,128,125,125,124,124,123,123,122,122,115,115,112,112,104,104,100,100,92,92,91,91,88,88,85,85,84,84,81,81,80,80,77,77,76,76,75,75,72,72,71,71,0.0],[8818,8818,8818,7361,7361,6795,6795,6250,6250,4542,4542,3955,3955,2517,2517,2366,2366,2024,2024,1704,1704,1414,1414,1236,1236,1230,1230,1180,1180,1145,1145,1116,1116,1042,1042,988,988,943,943,941,941,926,926,851,851,796,796,770,770,760,760,757,757,756,756,740,740,721,721,701,701,694,694,690,690,686,686,678,678,673,673,664,664,656,656,652,652,592,592,575,575,570,570,551,551,540,540,515,515,509,509,467,467,455,455,431,431,425,425,421,421,420,420,413,413,410,410,397,397,385,385,377,377,376,376,374,374,369,369,353,353,343,343,342,342,339,339,328,328,325,325,317,317,316,316,297,297,281,281,278,278,276,276,255,255,248,248,236,236,234,234,231,231,192,192,183,183,173,173,155,155,150,150,146,146,143,143,135,135,128,128,125,125,122,122,120,120,117,117,112,112,104,104,102,102,101,101,91,91,88,88,86,86,85,85,84,84,81,81,80,80,77,77,76,76,75,75,72,72,71,71,0.0],[452382,452382,452382,381928,381928,337310,337310,326621,326621,323000,323000,314777,314777,312742,312742,309859,309859,285102,285102,280904,280904,280902,280902,274879,274879,272514,272514,271008,271008,265386,265386,259223,259223,246079,246079,213922,213922,206494,206494,201084,201084,198173,198173,195501,195501,194929,194929,186212,186212,184539,184539,183884,183884,182294,182294,172256,172256,169988,169988,167956,167956,167150,167150,166496,166496,165916,165916,161638,161638,156152,156152,152662,152662,151152,151152,148077,148077,129525,129525,128007,128007,122839,122839,120560,120560,120514,120514,112477,112477,112393,112393,110388,110388,109971,109971,104584,104584,102881,102881,102076,102076,101650,101650,97563,97563,95644,95644,94273,94273,93327,93327,92498,92498,91796,91796,91698,91698,89808,89808,76925,76925,75590,75590,73308,73308,73081,73081,71639,71639,71041,71041,70345,70345,69386,69386,69319,69319,69098,69098,68460,68460,66256,66256,65592,65592,63677,63677,62231,62231,61743,61743,60188,60188,59794,59794,59070,59070,58169,58169,53982,53982,52797,52797,50995,50995,49912,49912,49079,49079,49004,49004,47781,47781,47682,47682,46699,46699,46593,46593,45130,45130,45060,45060,44628,44628,43328,43328,42584,42584,42347,42347,41551,41551,41301,41301,40459,40459,40398,40398,39474,39474,38988,38988,35741,35741,33792,33792,32900,32900,32531,32531,31995,31995,30166,30166,30164,30164,29886,29886,28884,28884,28590,28590,26644,26644,26141,26141,25495,25495,24940,24940,24797,24797,24764,24764,23667,23667,23211,23211,22065,22065,21438,21438,19061,19061,18870,18870,17828,17828,17661,17661,17369,17369,17264,17264,16934,16934,16541,16541,15884,15884,15488,15488,15004,15004,14896,14896,13161,13161,12994,12994,12986,12986,11909,11909,11125,11125,10958,10958,10920,10920,10812,10812,10651,10651,10395,10395,9985,9985,9921,9921,9496,9496,9051,9051,
[truncated: 769,951 more chars]
