# Supplementary figures and images for: Biological and genomic resources for the cosmopolitan phytoplankton Bathycoccus: insights into genetic diversity and function of outlier chromosomes
Source: Plant J. 2026 Jun 7;126(5):e70982. doi: 10.1111/tpj.70982 (PMC13242883; doi:10.1111/tpj.70982)

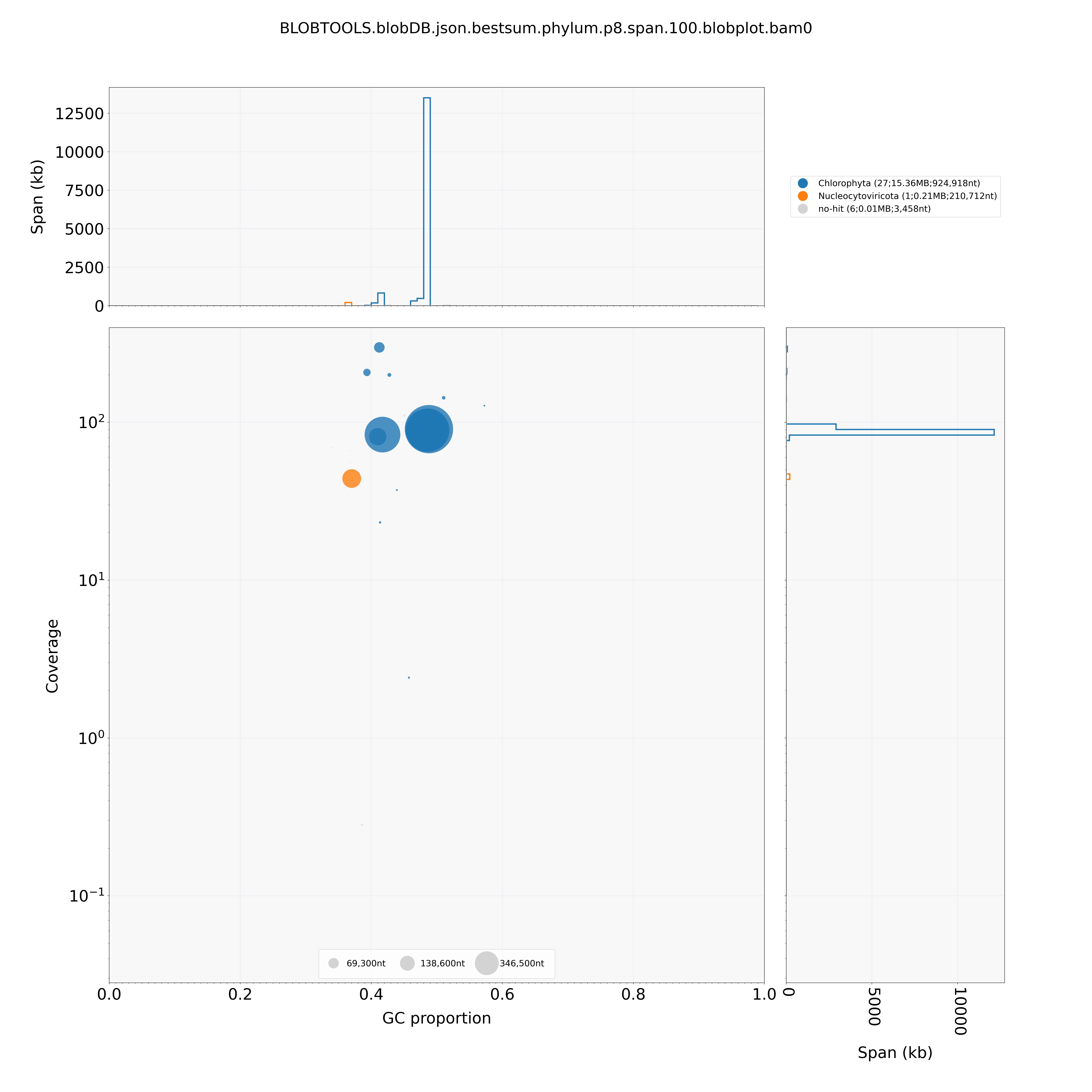

Supplement: Supplementary file 2 — Data S2. Taxonomic partitioning of assembled contigs. [file TPJ-126-0-s002.zip › blobtoolsA1/BLOBTOOLS.blobDB.json.bestsum.phylum.p8.span.100.blobplot.bam0.png]

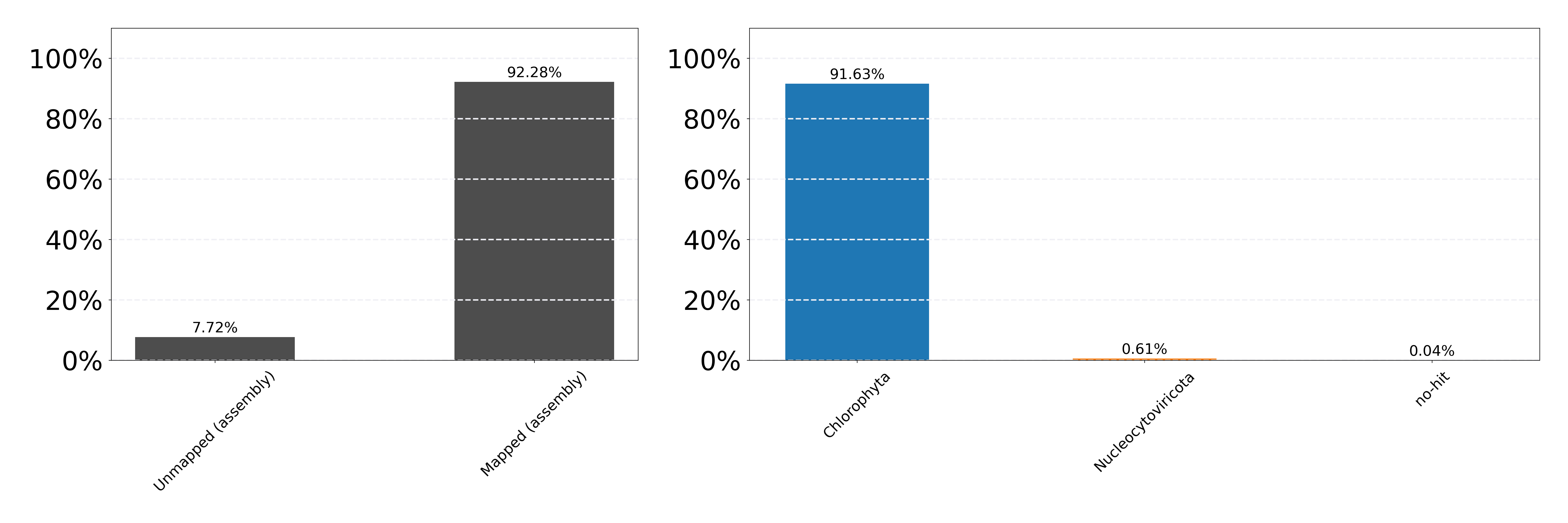

Supplement: Supplementary file 2 — Data S2. Taxonomic partitioning of assembled contigs. [file TPJ-126-0-s002.zip › blobtoolsA1/BLOBTOOLS.blobDB.json.bestsum.phylum.p8.span.100.blobplot.read_cov.bam0.png]

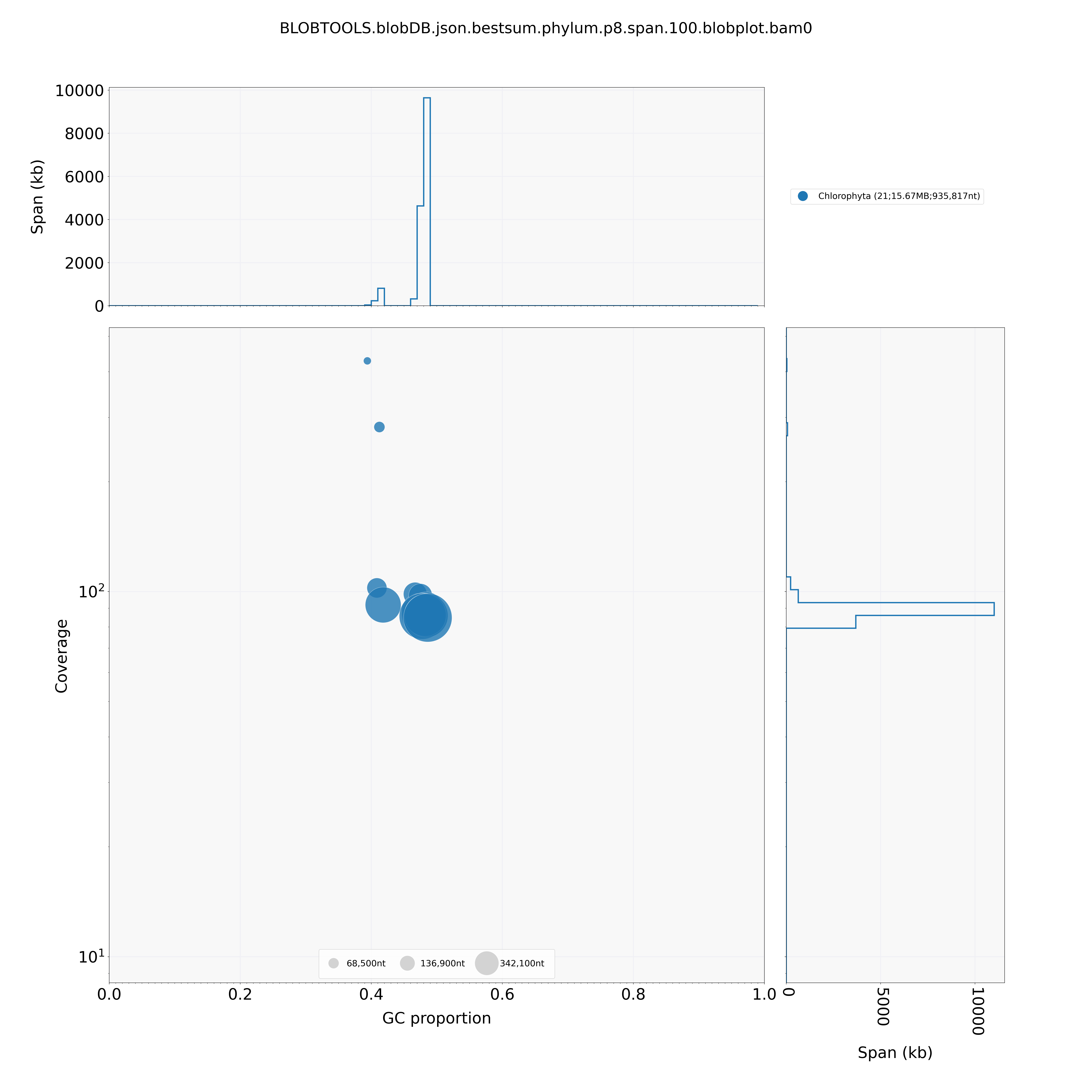

Supplement: Supplementary file 2 — Data S2. Taxonomic partitioning of assembled contigs. [file TPJ-126-0-s002.zip › blobtoolsA727/BLOBTOOLS.blobDB.json.bestsum.phylum.p8.span.100.blobplot.bam0.png]

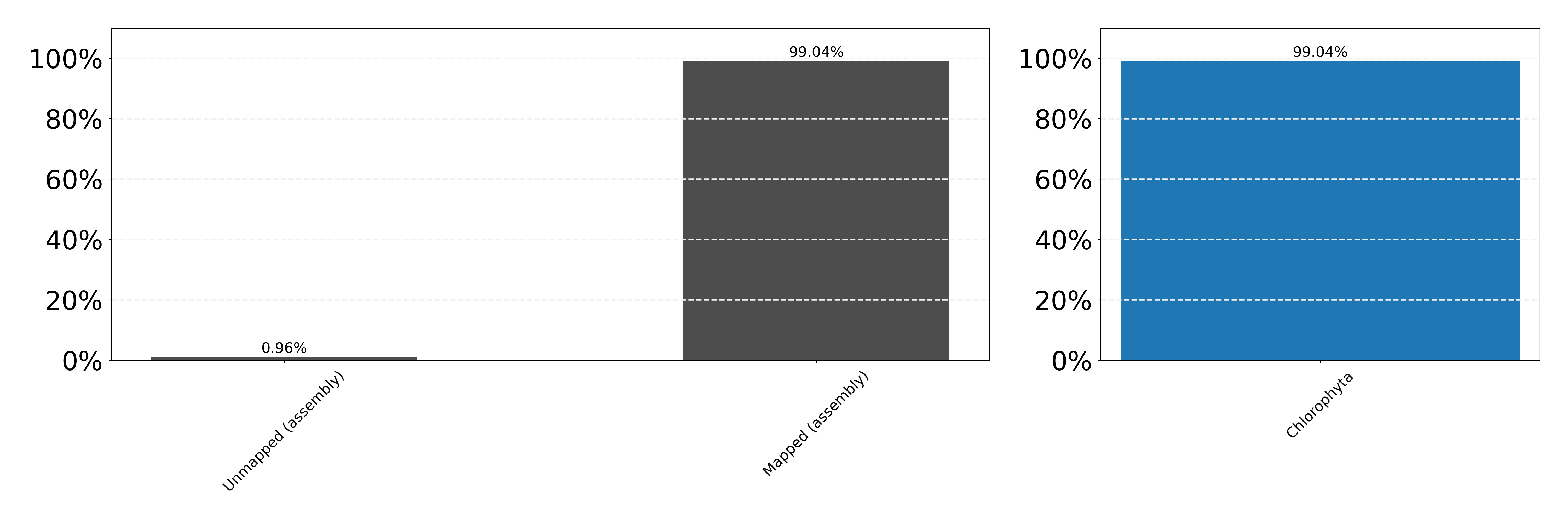

Supplement: Supplementary file 2 — Data S2. Taxonomic partitioning of assembled contigs. [file TPJ-126-0-s002.zip › blobtoolsA727/BLOBTOOLS.blobDB.json.bestsum.phylum.p8.span.100.blobplot.read_cov.bam0.png]

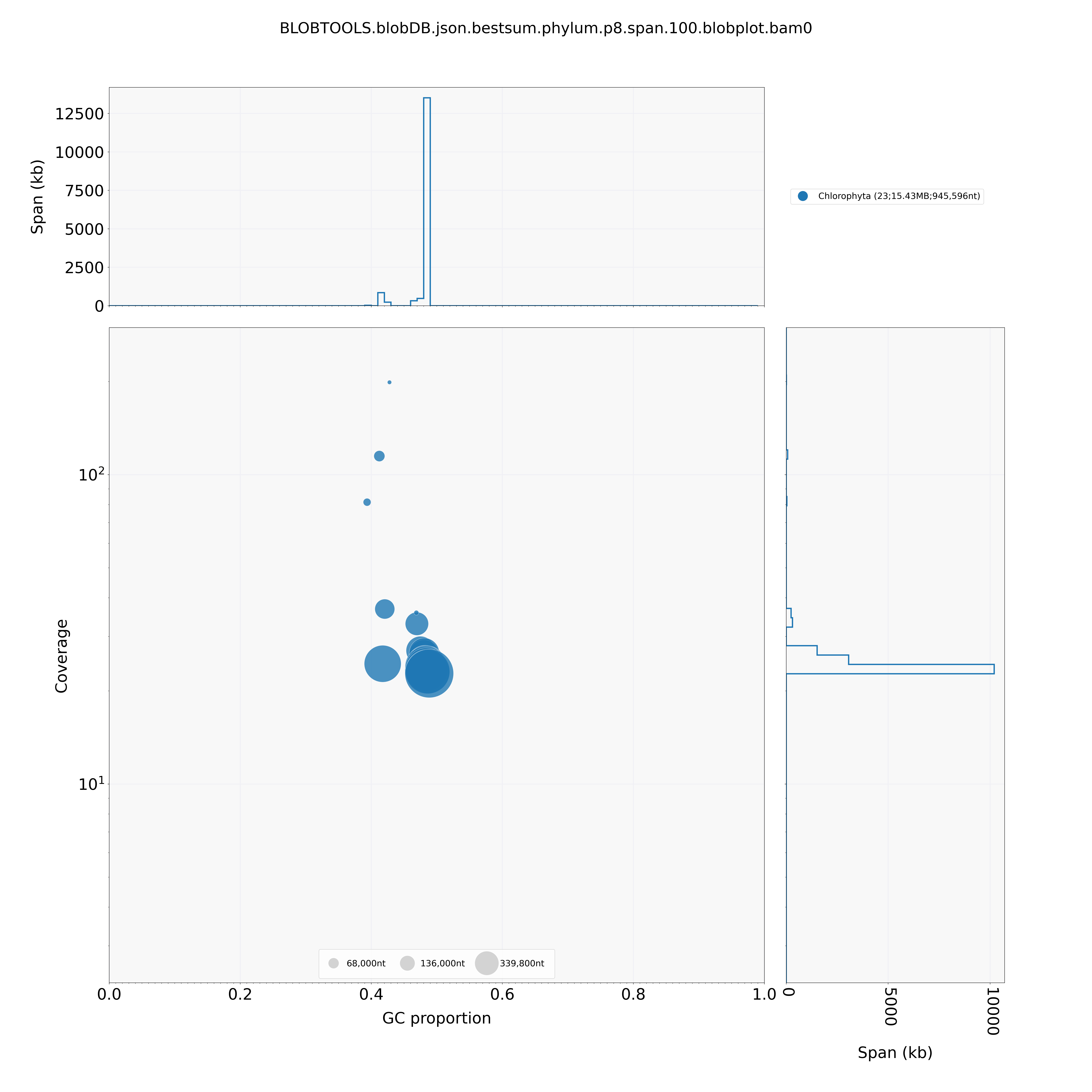

Supplement: Supplementary file 2 — Data S2. Taxonomic partitioning of assembled contigs. [file TPJ-126-0-s002.zip › blobtoolsA8/BLOBTOOLS.blobDB.json.bestsum.phylum.p8.span.100.blobplot.bam0.png]

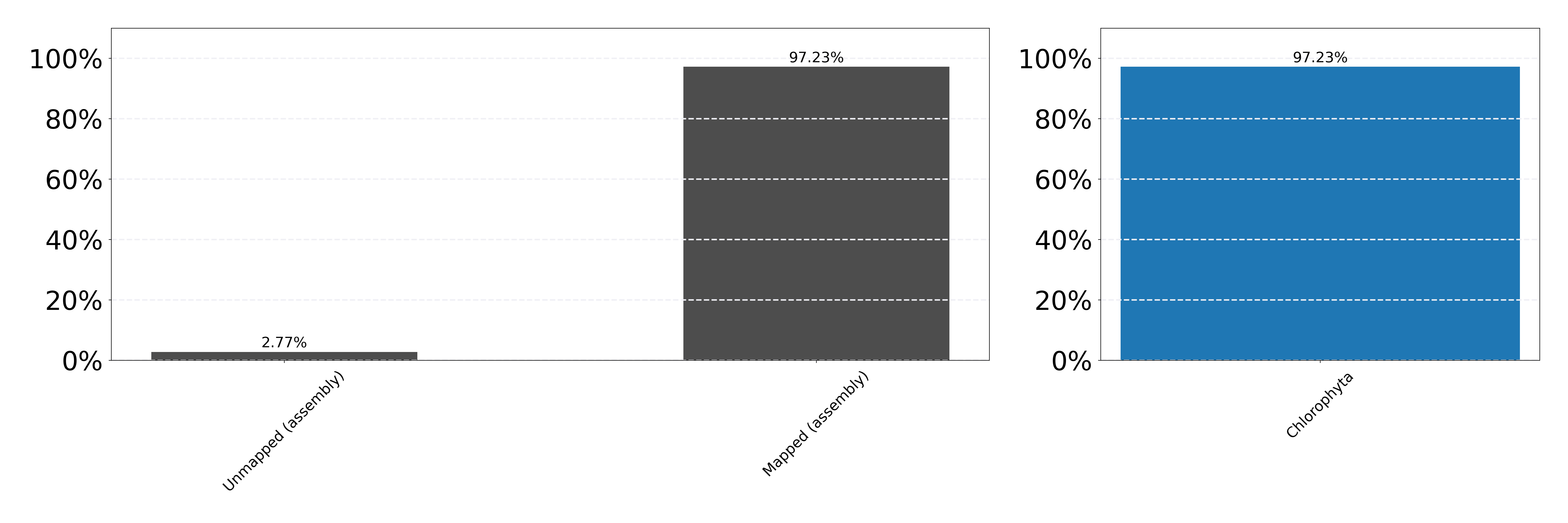

Supplement: Supplementary file 2 — Data S2. Taxonomic partitioning of assembled contigs. [file TPJ-126-0-s002.zip › blobtoolsA8/BLOBTOOLS.blobDB.json.bestsum.phylum.p8.span.100.blobplot.read_cov.bam0.png]

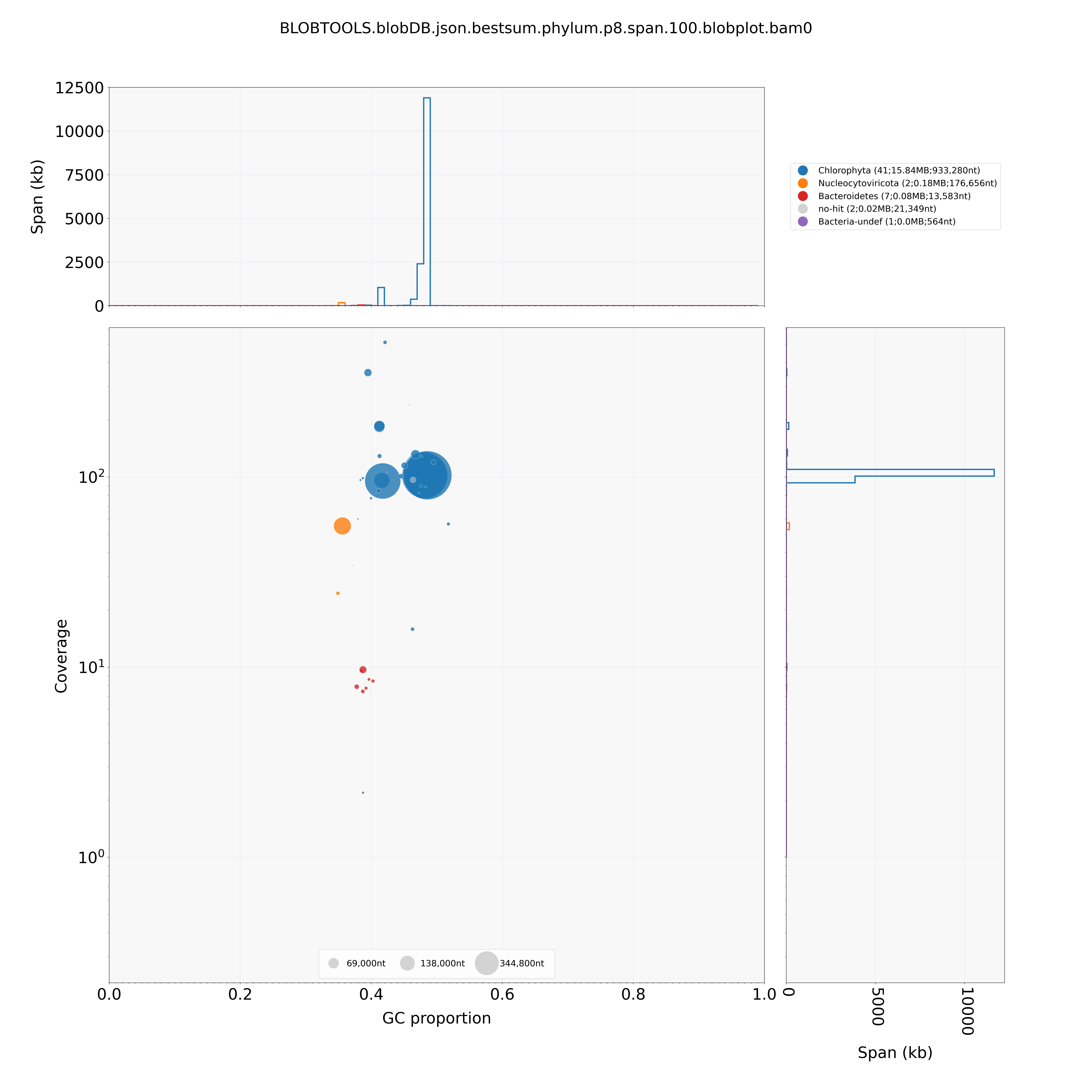

Supplement: Supplementary file 2 — Data S2. Taxonomic partitioning of assembled contigs. [file TPJ-126-0-s002.zip › blobtoolsA818/BLOBTOOLS.blobDB.json.bestsum.phylum.p8.span.100.blobplot.bam0.png]

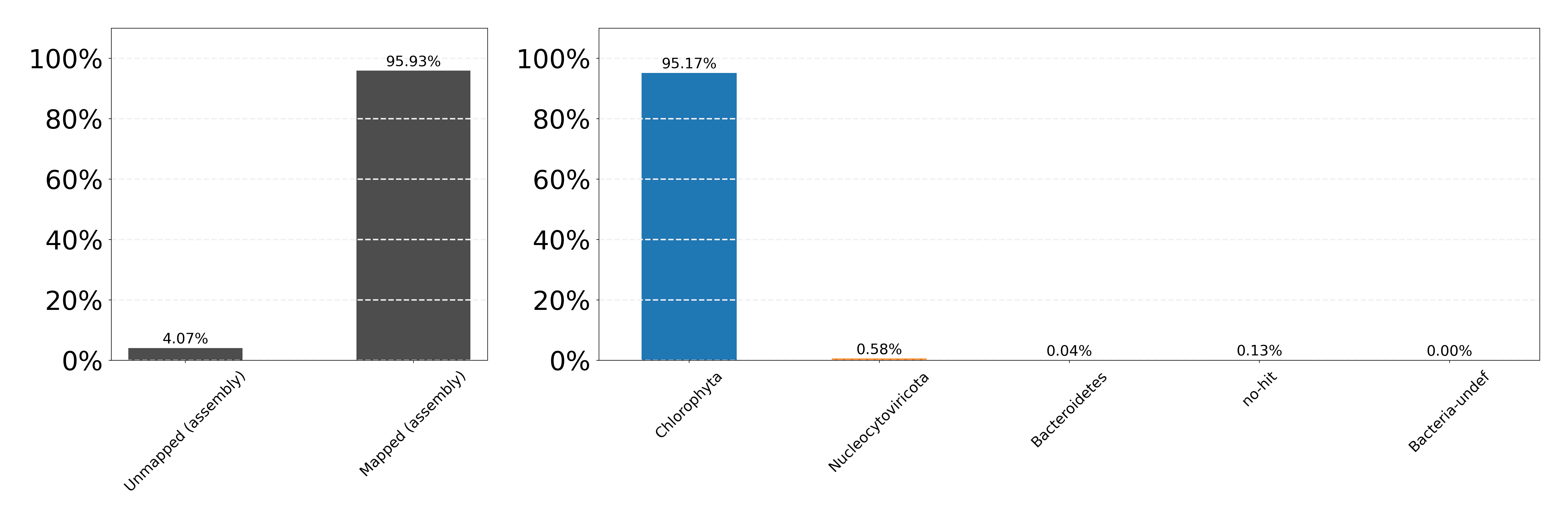

Supplement: Supplementary file 2 — Data S2. Taxonomic partitioning of assembled contigs. [file TPJ-126-0-s002.zip › blobtoolsA818/BLOBTOOLS.blobDB.json.bestsum.phylum.p8.span.100.blobplot.read_cov.bam0.png]

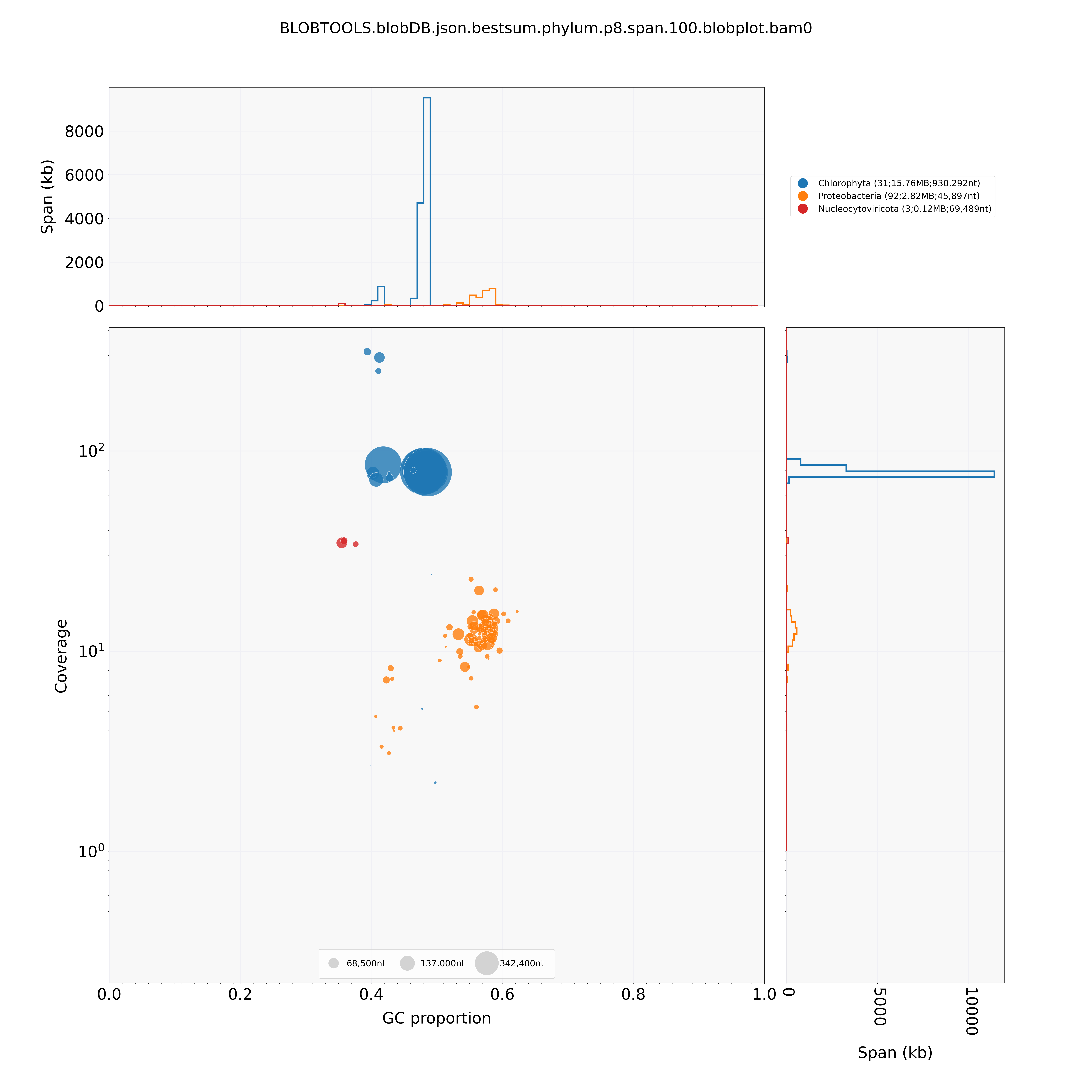

Supplement: Supplementary file 2 — Data S2. Taxonomic partitioning of assembled contigs. [file TPJ-126-0-s002.zip › blobtoolsA827/BLOBTOOLS.blobDB.json.bestsum.phylum.p8.span.100.blobplot.bam0.png]

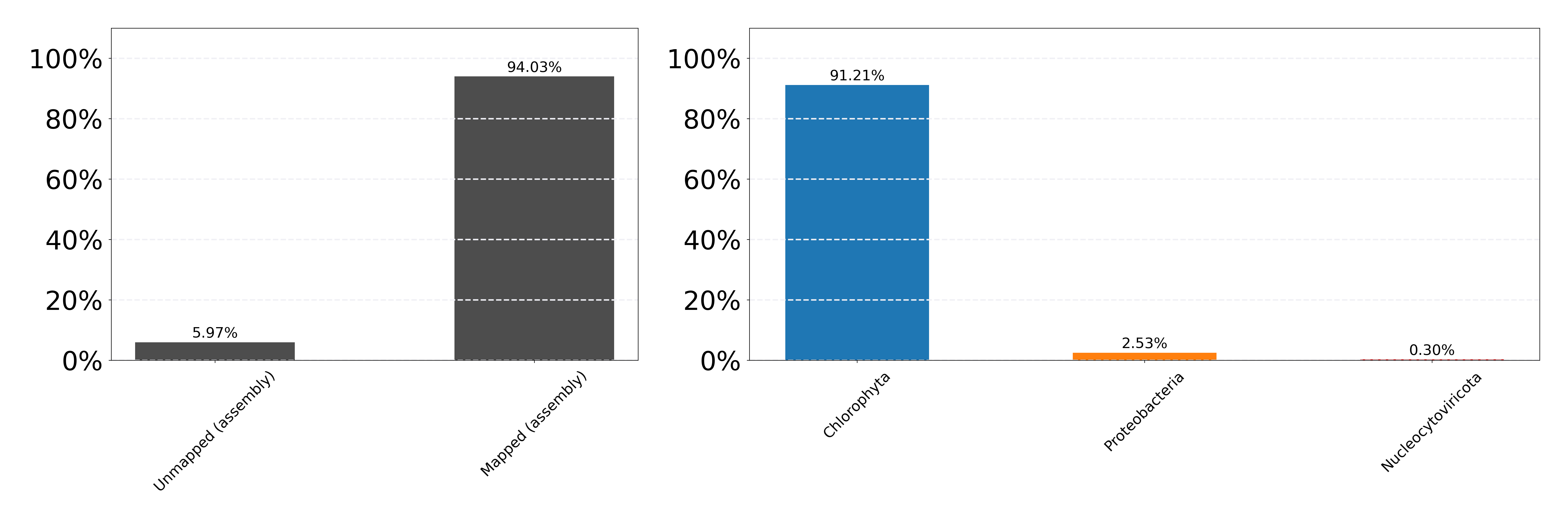

Supplement: Supplementary file 2 — Data S2. Taxonomic partitioning of assembled contigs. [file TPJ-126-0-s002.zip › blobtoolsA827/BLOBTOOLS.blobDB.json.bestsum.phylum.p8.span.100.blobplot.read_cov.bam0.png]

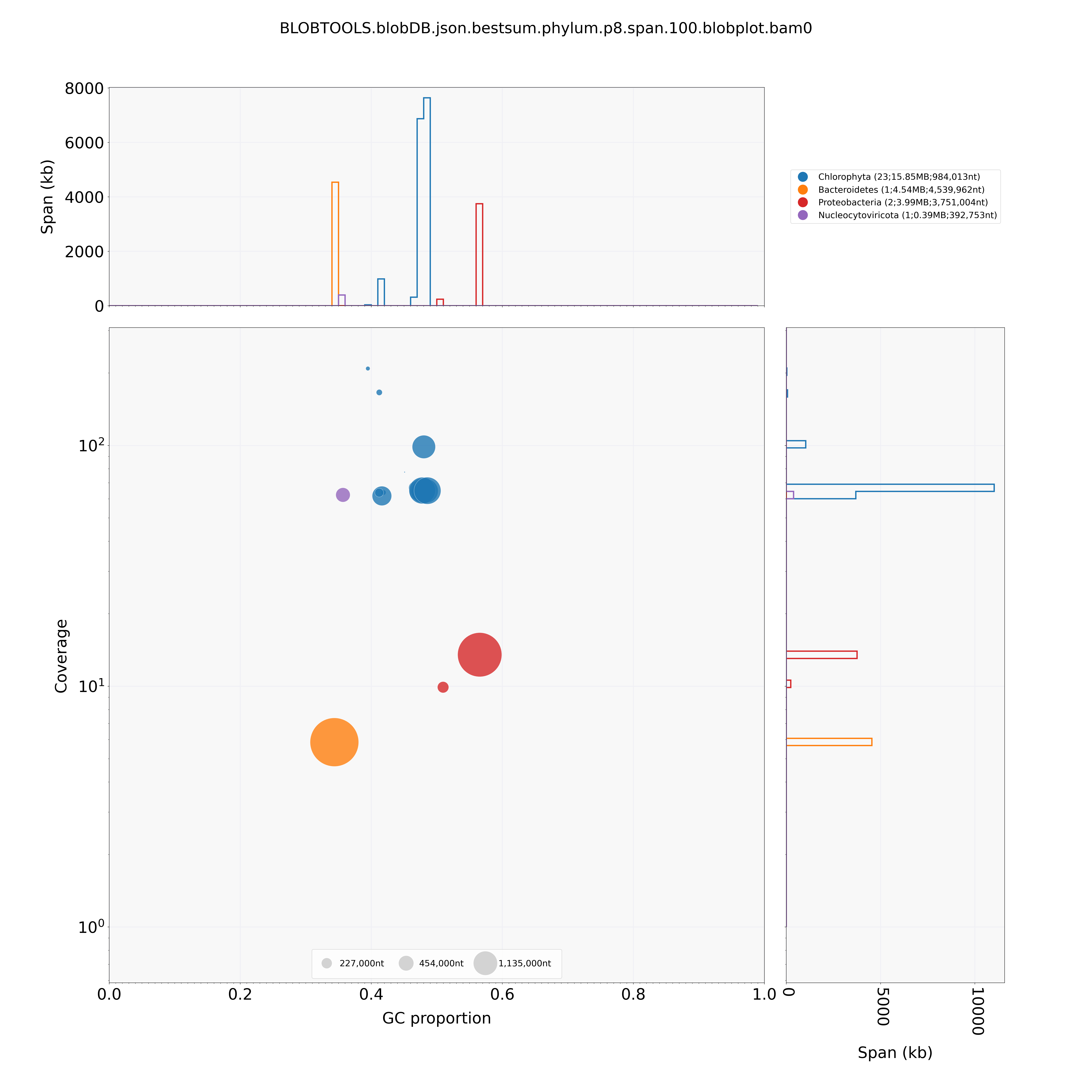

Supplement: Supplementary file 2 — Data S2. Taxonomic partitioning of assembled contigs. [file TPJ-126-0-s002.zip › blobtoolsB218/BLOBTOOLS.blobDB.json.bestsum.phylum.p8.span.100.blobplot.bam0.png]

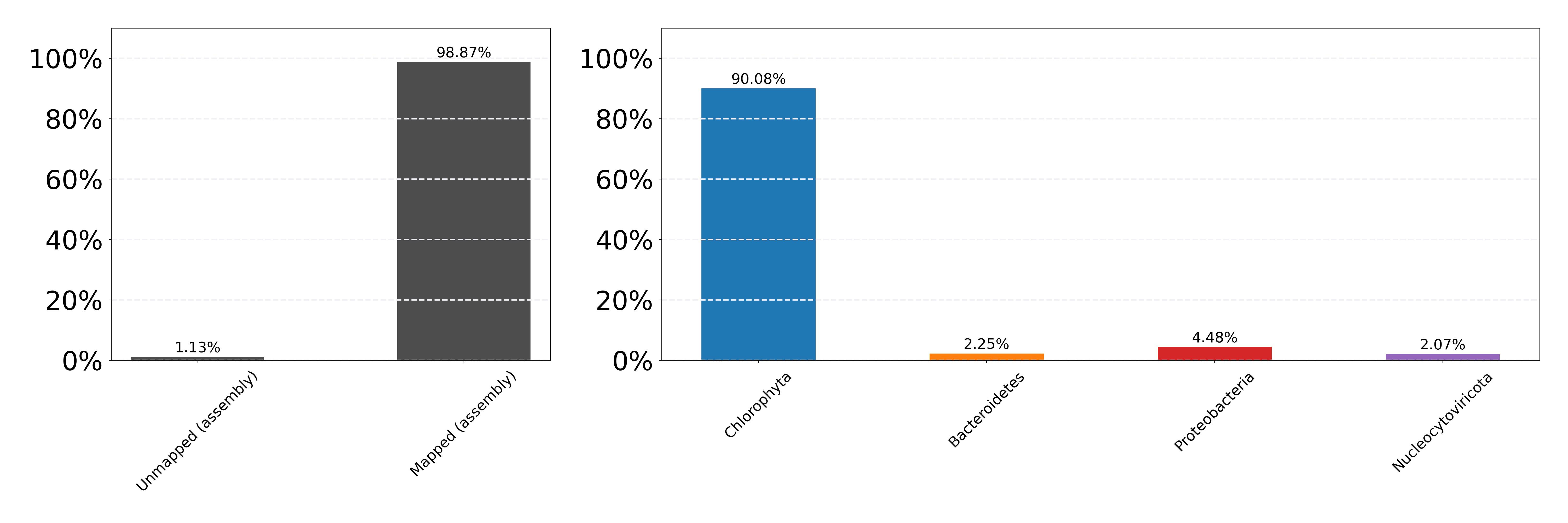

Supplement: Supplementary file 2 — Data S2. Taxonomic partitioning of assembled contigs. [file TPJ-126-0-s002.zip › blobtoolsB218/BLOBTOOLS.blobDB.json.bestsum.phylum.p8.span.100.blobplot.read_cov.bam0.png]

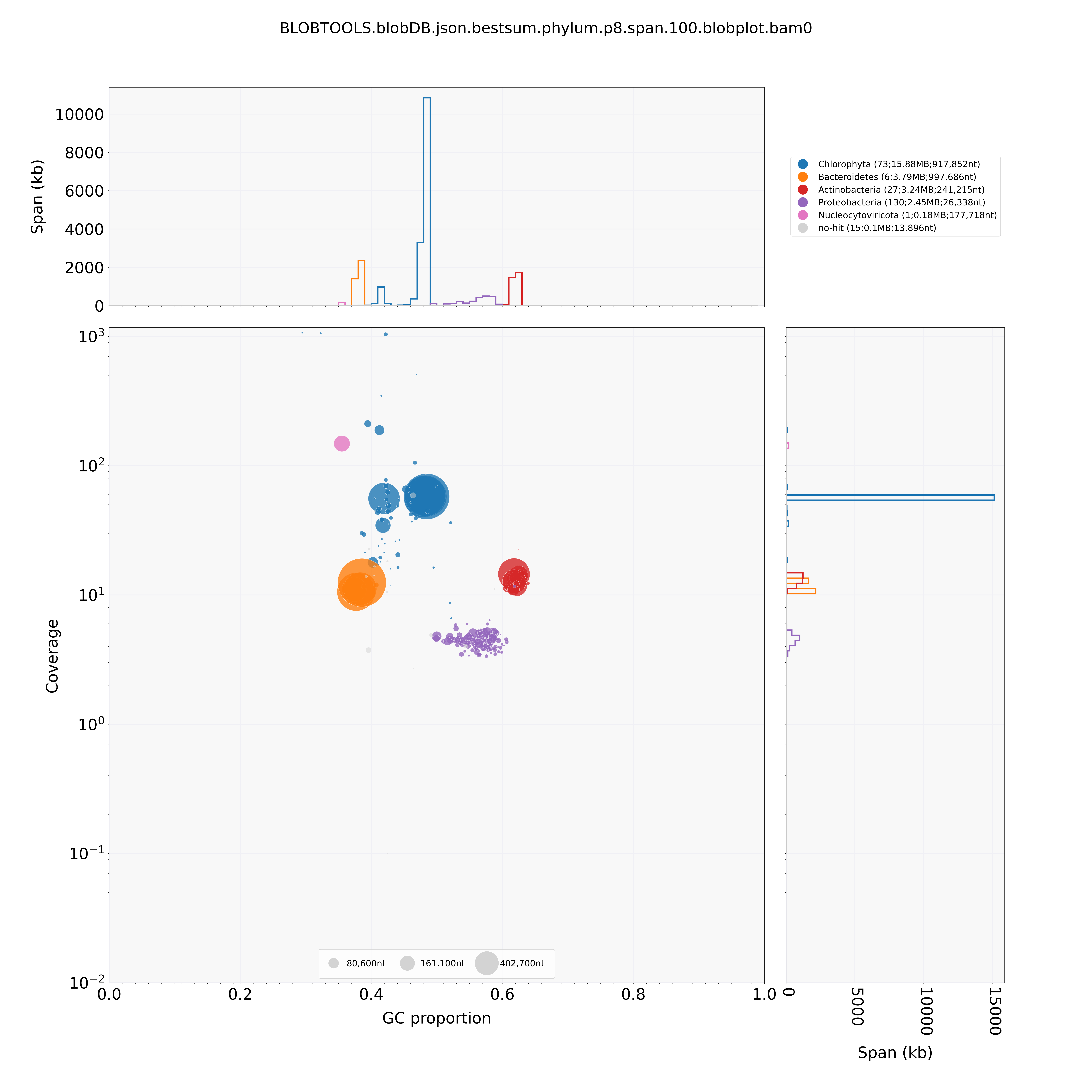

Supplement: Supplementary file 2 — Data S2. Taxonomic partitioning of assembled contigs. [file TPJ-126-0-s002.zip › blobtoolsB518/BLOBTOOLS.blobDB.json.bestsum.phylum.p8.span.100.blobplot.bam0.png]

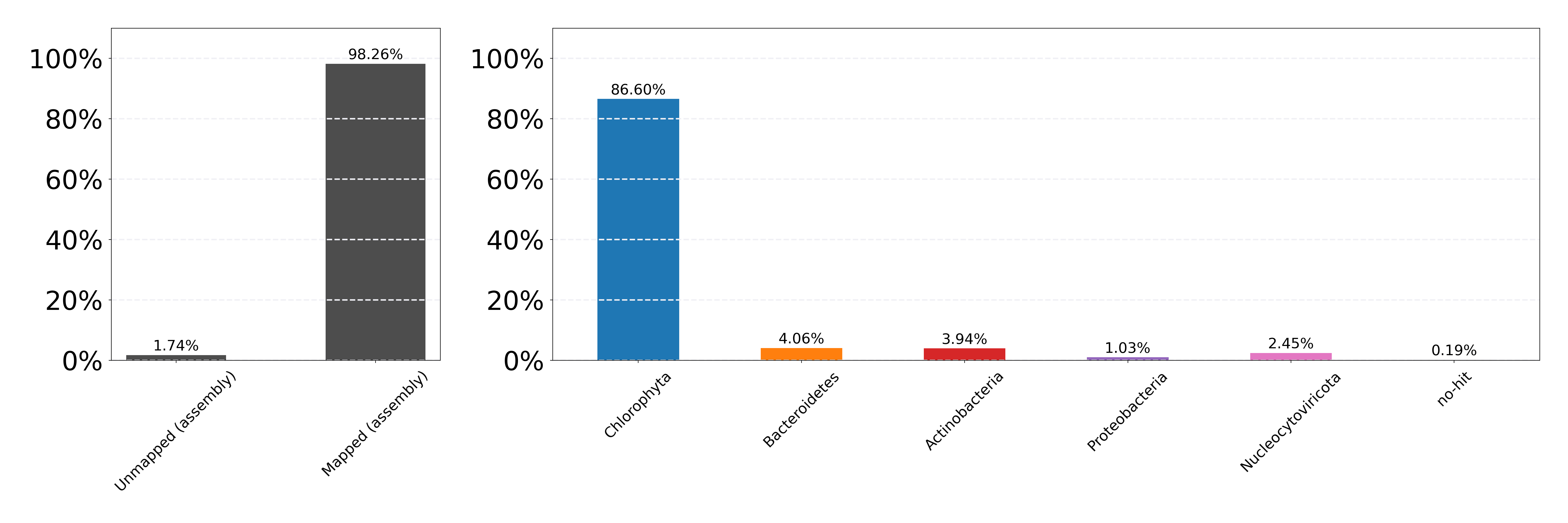

Supplement: Supplementary file 2 — Data S2. Taxonomic partitioning of assembled contigs. [file TPJ-126-0-s002.zip › blobtoolsB518/BLOBTOOLS.blobDB.json.bestsum.phylum.p8.span.100.blobplot.read_cov.bam0.png]

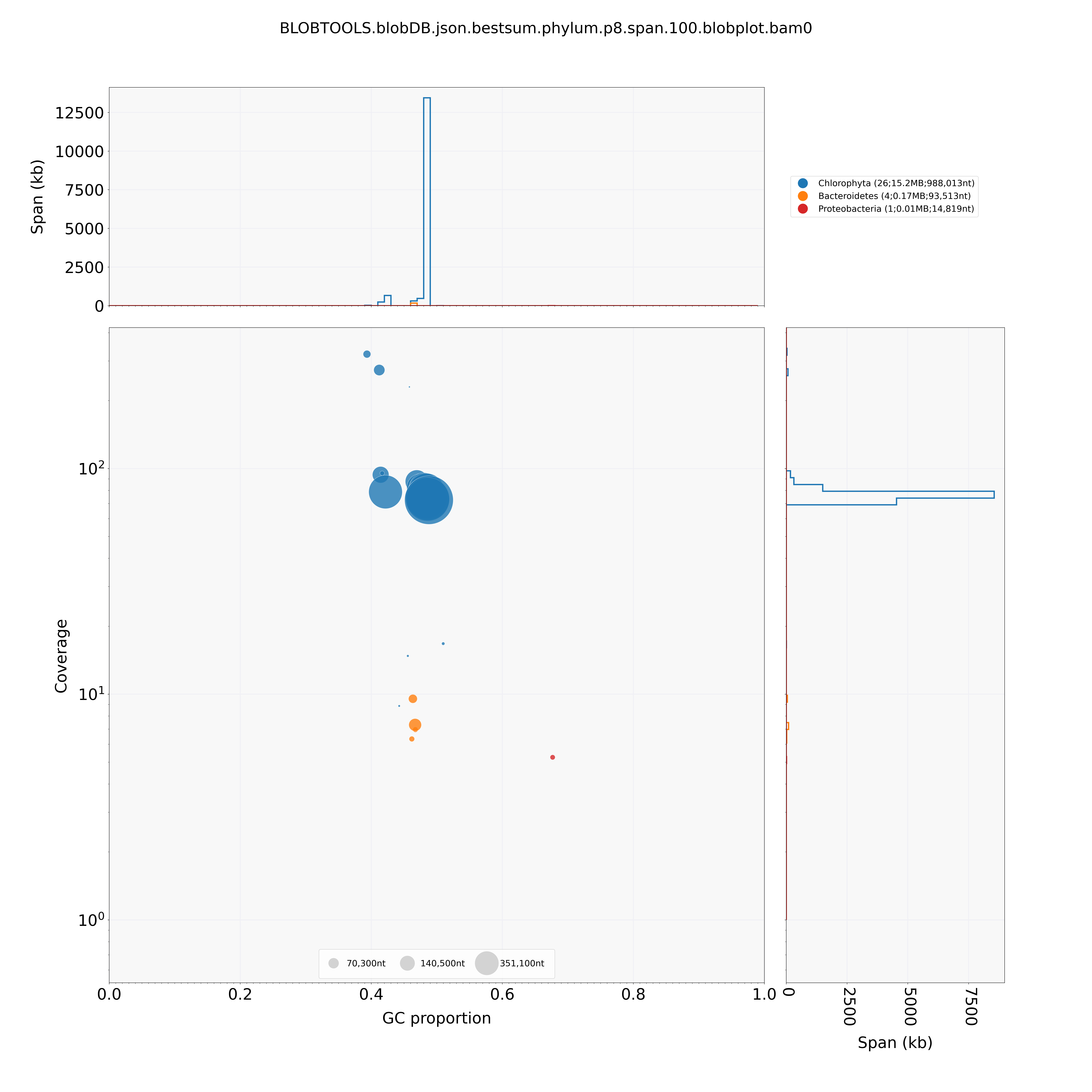

Supplement: Supplementary file 2 — Data S2. Taxonomic partitioning of assembled contigs. [file TPJ-126-0-s002.zip › blobtoolsC2/BLOBTOOLS.blobDB.json.bestsum.phylum.p8.span.100.blobplot.bam0.png]

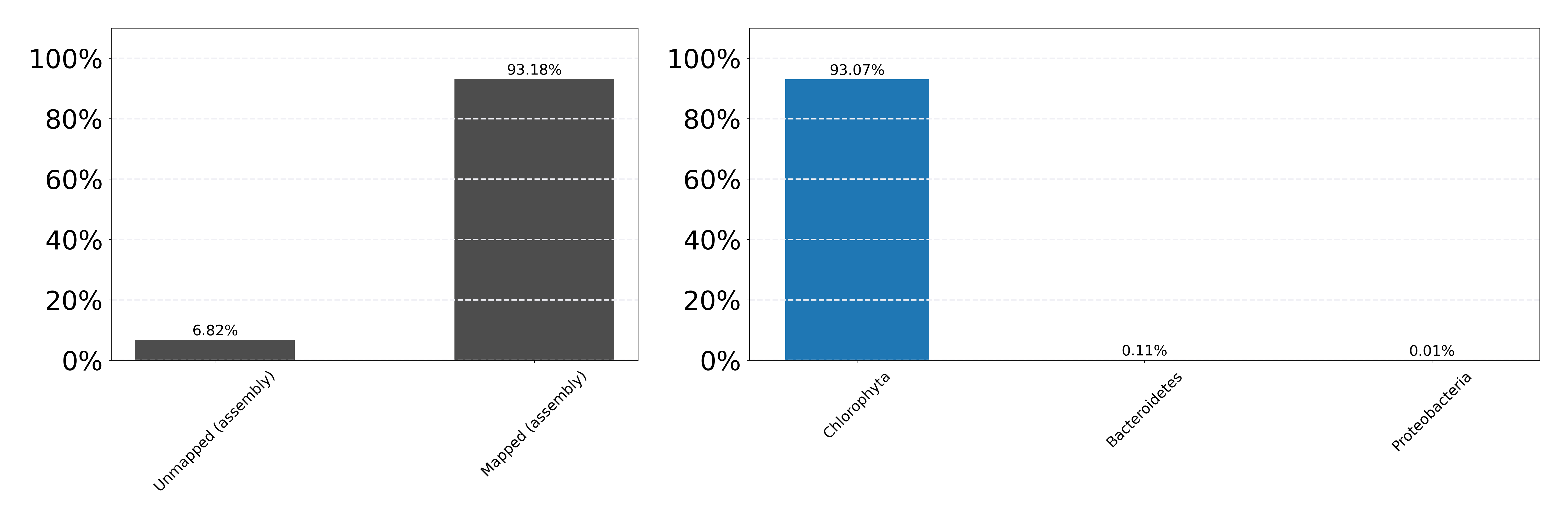

Supplement: Supplementary file 2 — Data S2. Taxonomic partitioning of assembled contigs. [file TPJ-126-0-s002.zip › blobtoolsC2/BLOBTOOLS.blobDB.json.bestsum.phylum.p8.span.100.blobplot.read_cov.bam0.png]

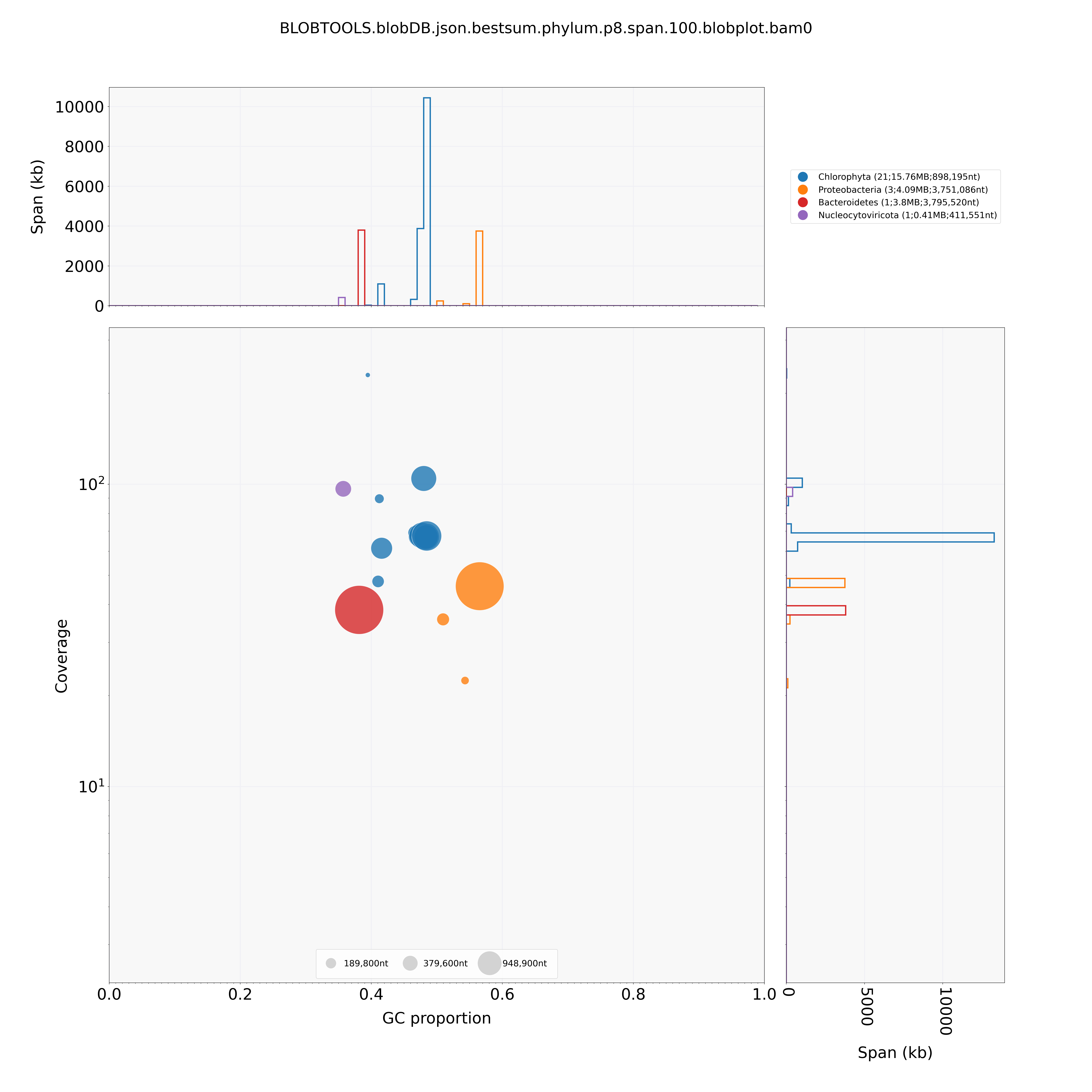

Supplement: Supplementary file 2 — Data S2. Taxonomic partitioning of assembled contigs. [file TPJ-126-0-s002.zip › blobtoolsC218/BLOBTOOLS.blobDB.json.bestsum.phylum.p8.span.100.blobplot.bam0.png]

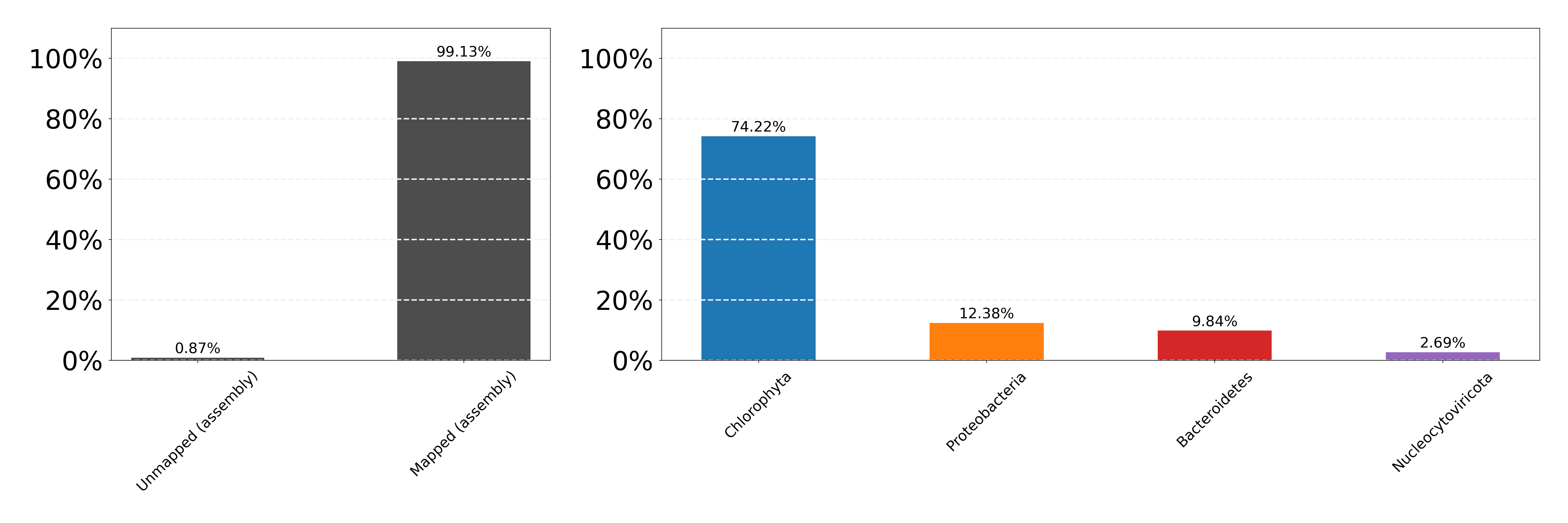

Supplement: Supplementary file 2 — Data S2. Taxonomic partitioning of assembled contigs. [file TPJ-126-0-s002.zip › blobtoolsC218/BLOBTOOLS.blobDB.json.bestsum.phylum.p8.span.100.blobplot.read_cov.bam0.png]

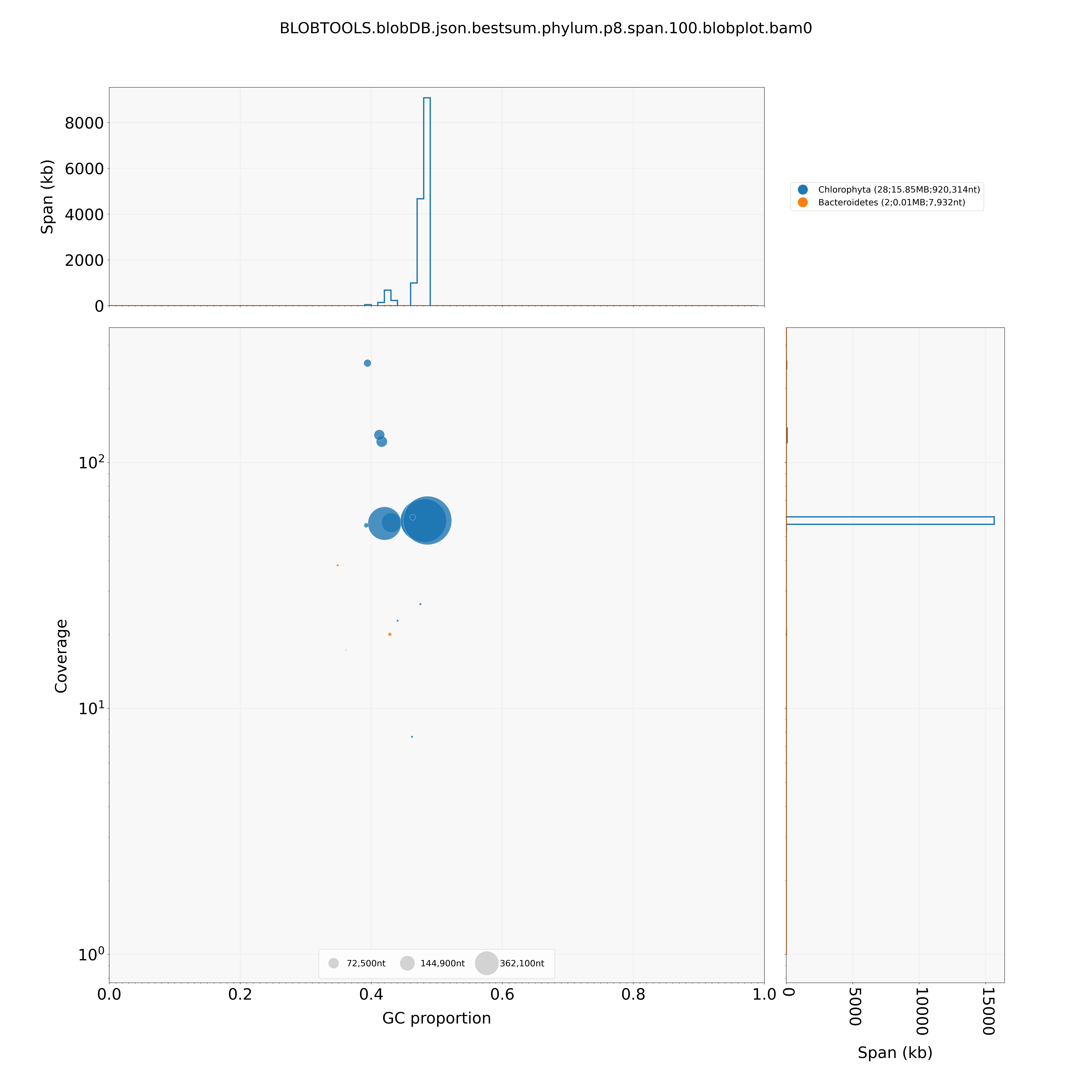

Supplement: Supplementary file 2 — Data S2. Taxonomic partitioning of assembled contigs. [file TPJ-126-0-s002.zip › blobtoolsD119/BLOBTOOLS.blobDB.json.bestsum.phylum.p8.span.100.blobplot.bam0.png]

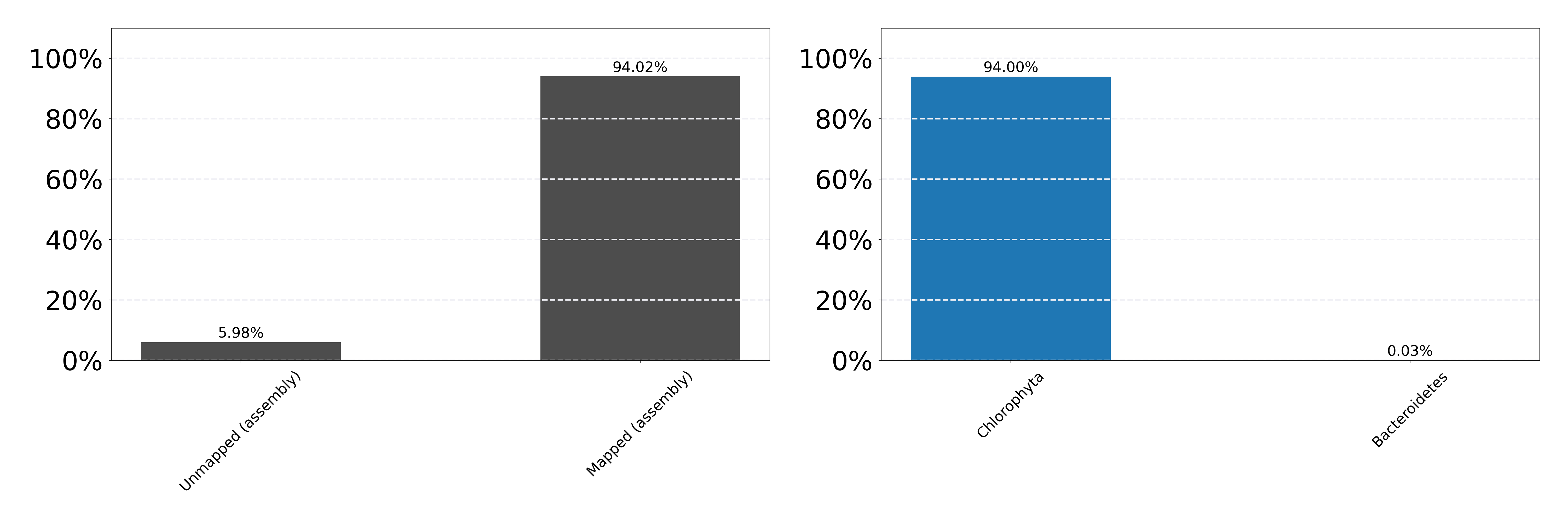

Supplement: Supplementary file 2 — Data S2. Taxonomic partitioning of assembled contigs. [file TPJ-126-0-s002.zip › blobtoolsD119/BLOBTOOLS.blobDB.json.bestsum.phylum.p8.span.100.blobplot.read_cov.bam0.png]

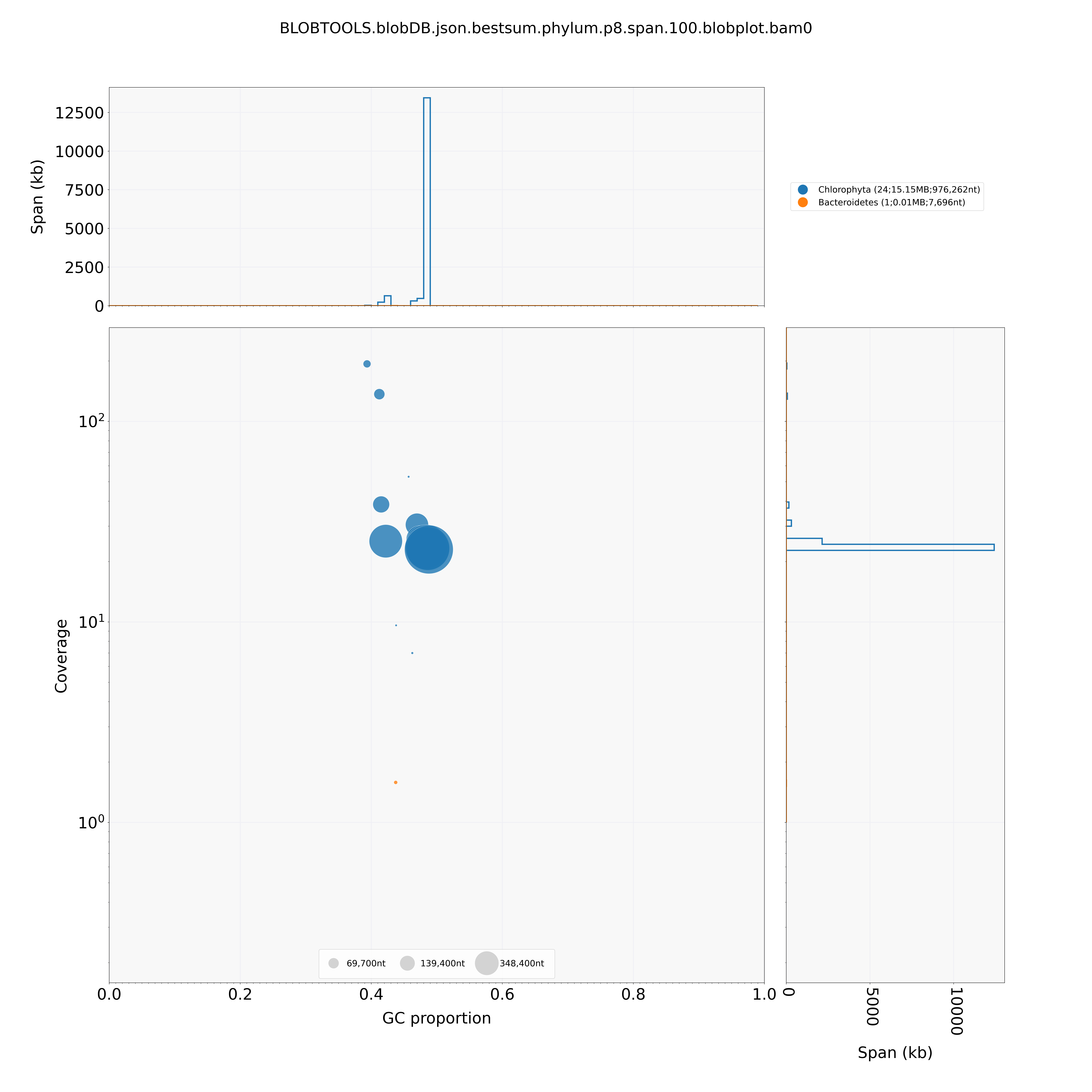

Supplement: Supplementary file 2 — Data S2. Taxonomic partitioning of assembled contigs. [file TPJ-126-0-s002.zip › blobtoolsE2/BLOBTOOLS.blobDB.json.bestsum.phylum.p8.span.100.blobplot.bam0.png]

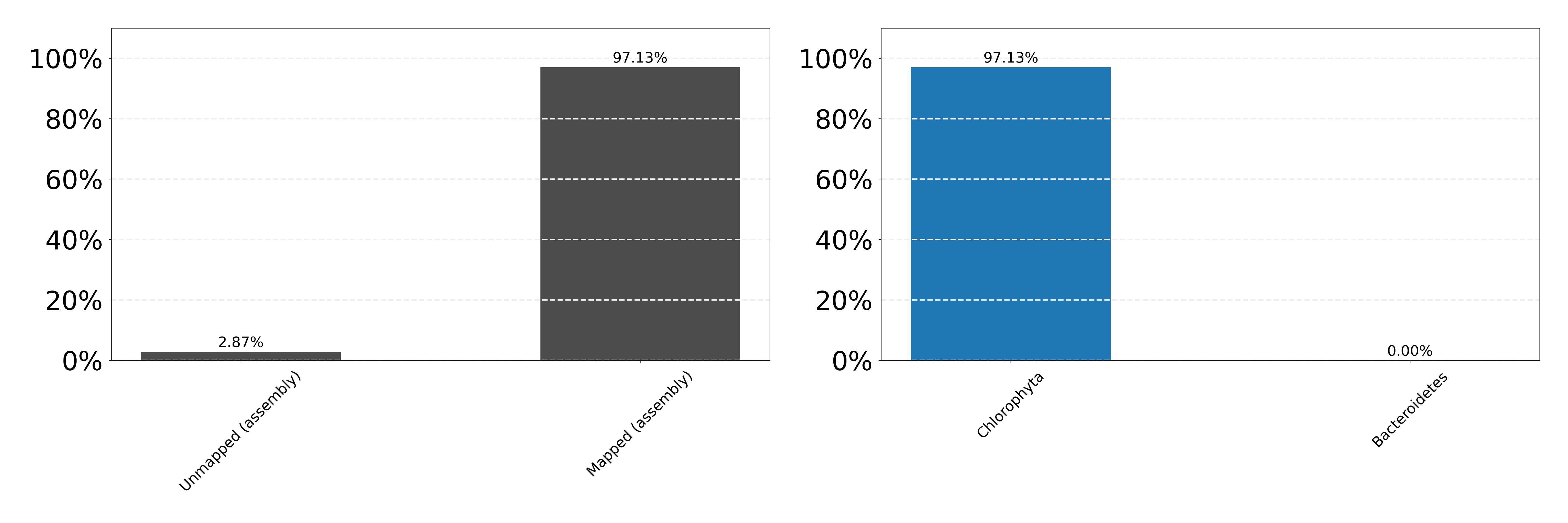

Supplement: Supplementary file 2 — Data S2. Taxonomic partitioning of assembled contigs. [file TPJ-126-0-s002.zip › blobtoolsE2/BLOBTOOLS.blobDB.json.bestsum.phylum.p8.span.100.blobplot.read_cov.bam0.png]

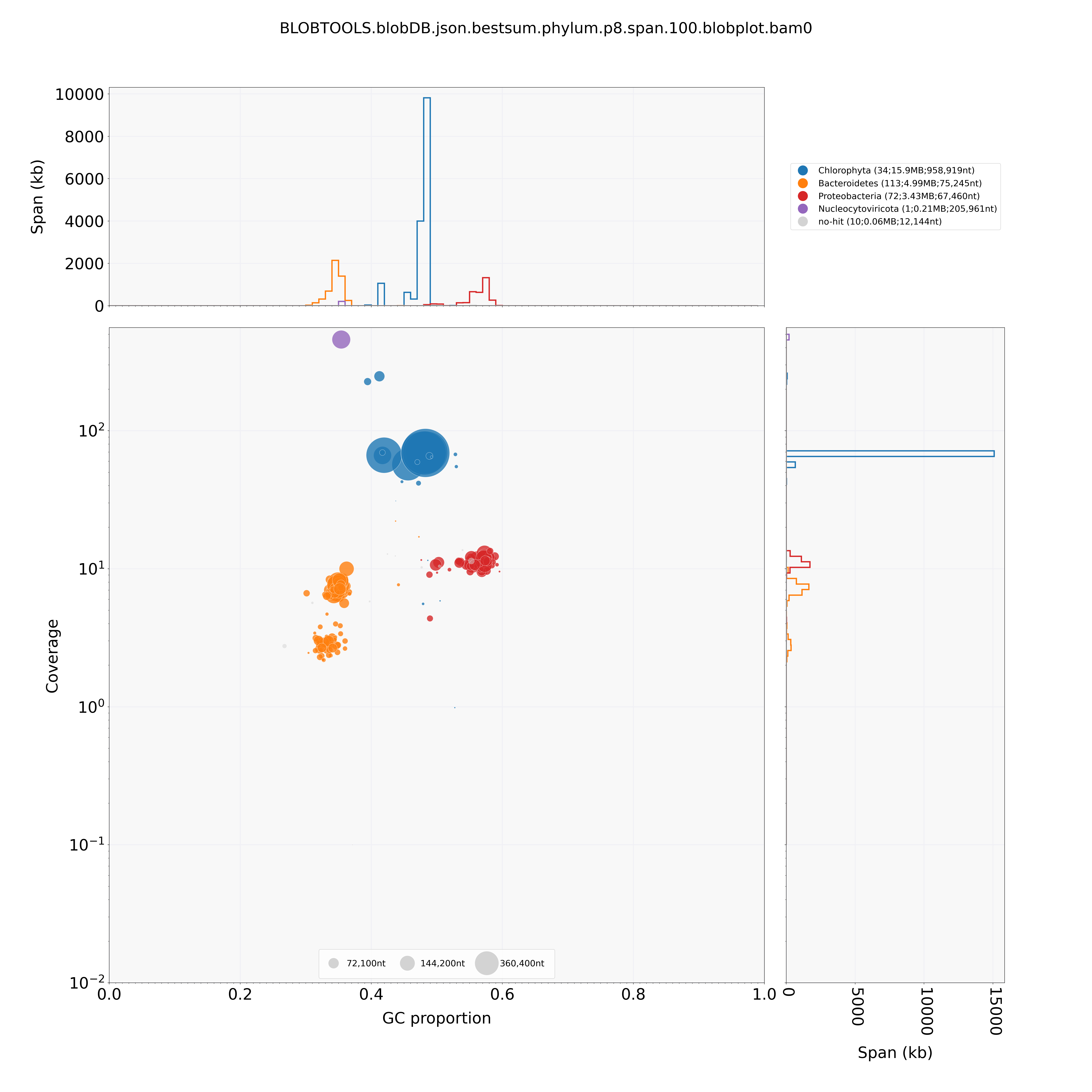

Supplement: Supplementary file 2 — Data S2. Taxonomic partitioning of assembled contigs. [file TPJ-126-0-s002.zip › blobtoolsE318/BLOBTOOLS.blobDB.json.bestsum.phylum.p8.span.100.blobplot.bam0.png]

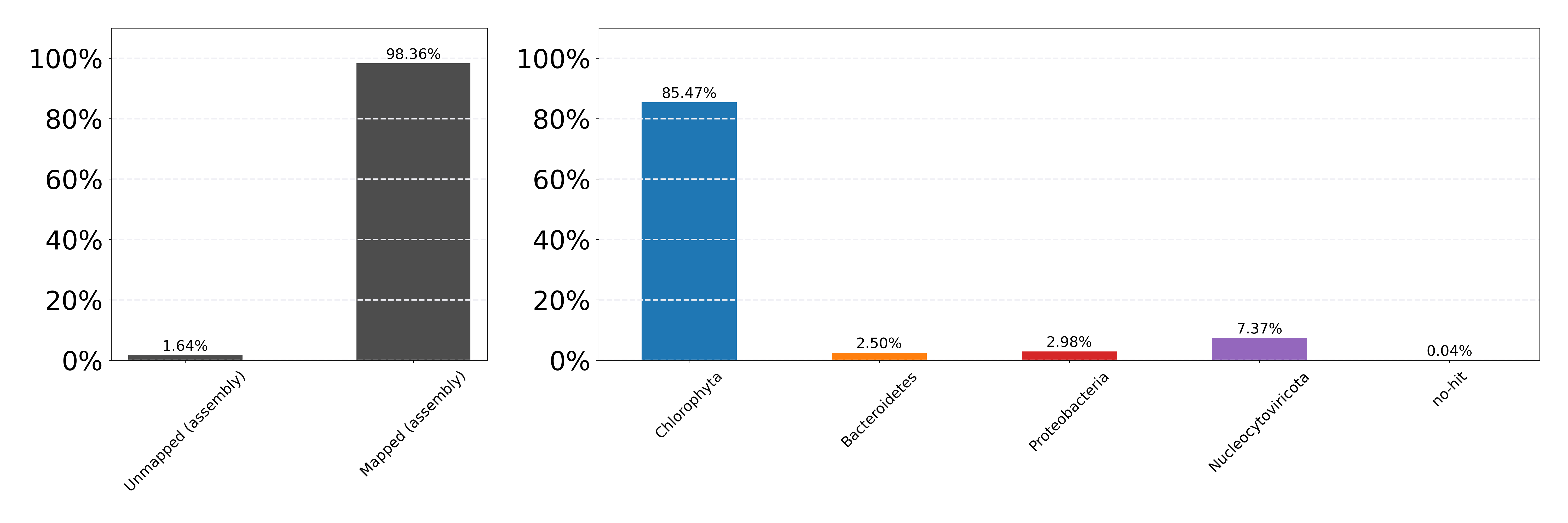

Supplement: Supplementary file 2 — Data S2. Taxonomic partitioning of assembled contigs. [file TPJ-126-0-s002.zip › blobtoolsE318/BLOBTOOLS.blobDB.json.bestsum.phylum.p8.span.100.blobplot.read_cov.bam0.png]

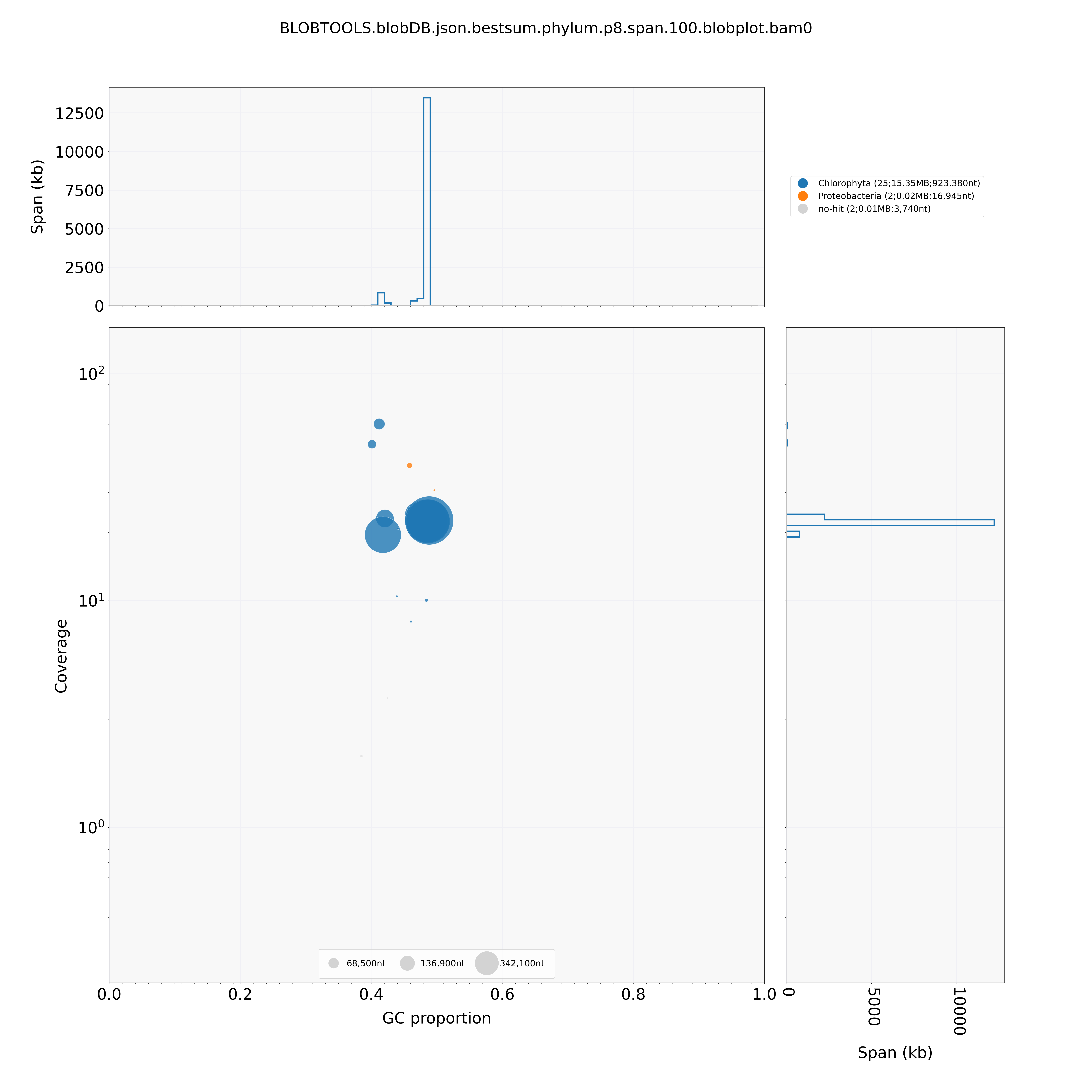

Supplement: Supplementary file 2 — Data S2. Taxonomic partitioning of assembled contigs. [file TPJ-126-0-s002.zip › blobtoolsG11/BLOBTOOLS.blobDB.json.bestsum.phylum.p8.span.100.blobplot.bam0.png]

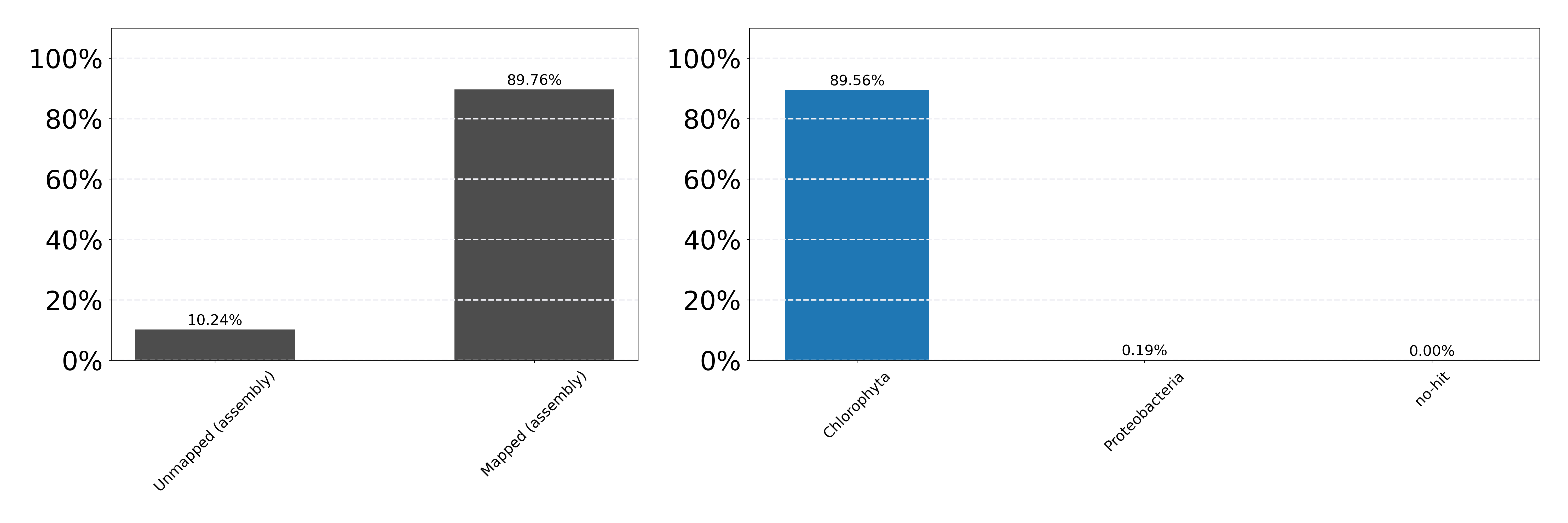

Supplement: Supplementary file 2 — Data S2. Taxonomic partitioning of assembled contigs. [file TPJ-126-0-s002.zip › blobtoolsG11/BLOBTOOLS.blobDB.json.bestsum.phylum.p8.span.100.blobplot.read_cov.bam0.png]

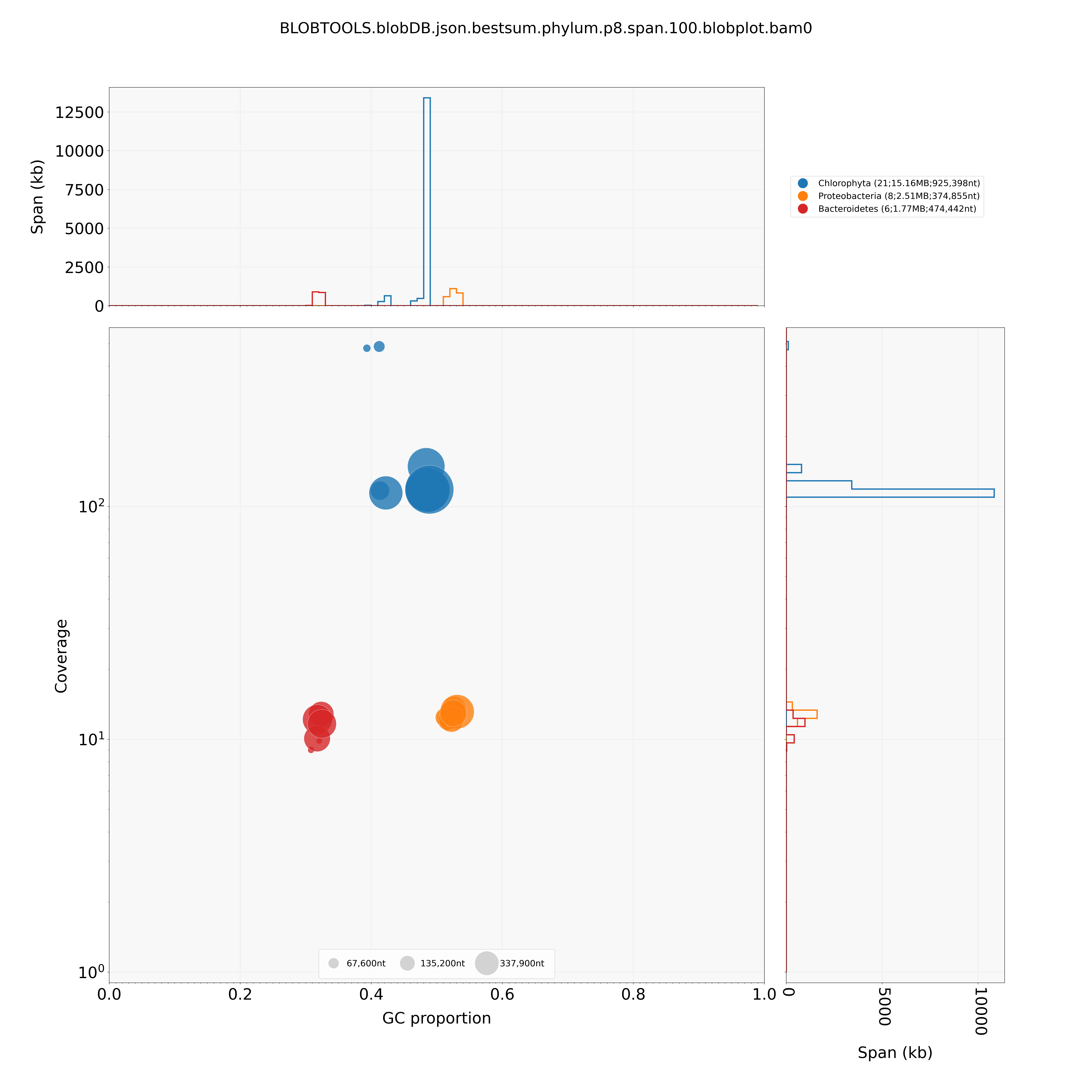

Supplement: Supplementary file 2 — Data S2. Taxonomic partitioning of assembled contigs. [file TPJ-126-0-s002.zip › blobtoolsG2/BLOBTOOLS.blobDB.json.bestsum.phylum.p8.span.100.blobplot.bam0.png]

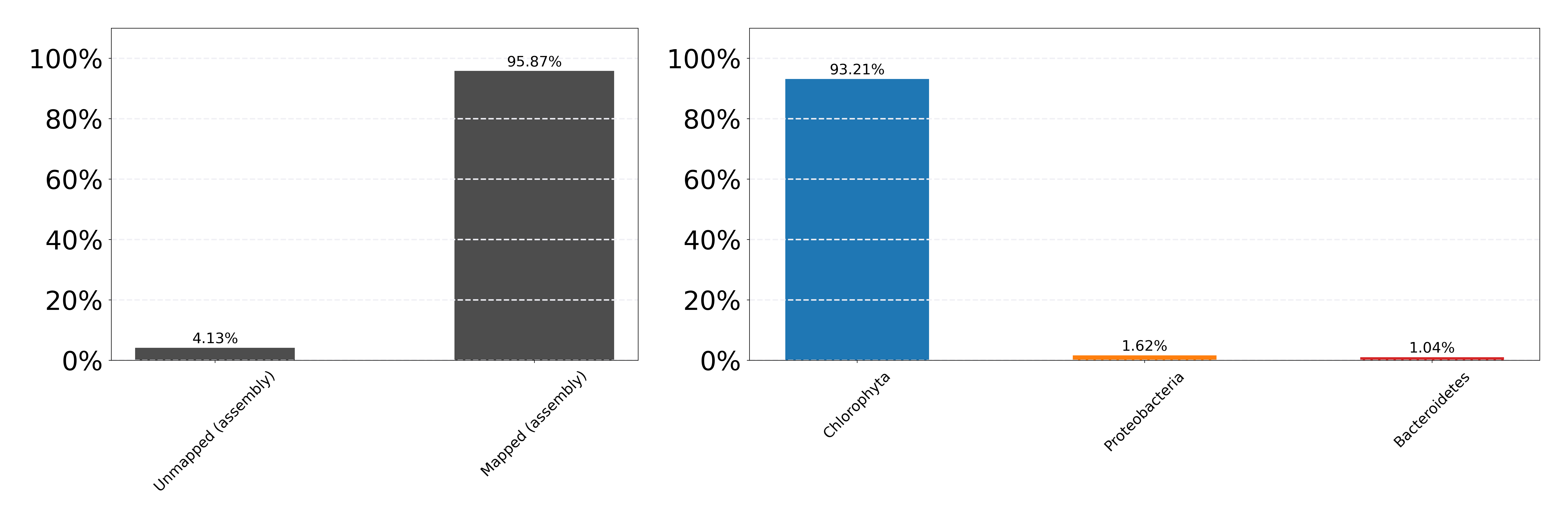

Supplement: Supplementary file 2 — Data S2. Taxonomic partitioning of assembled contigs. [file TPJ-126-0-s002.zip › blobtoolsG2/BLOBTOOLS.blobDB.json.bestsum.phylum.p8.span.100.blobplot.read_cov.bam0.png]

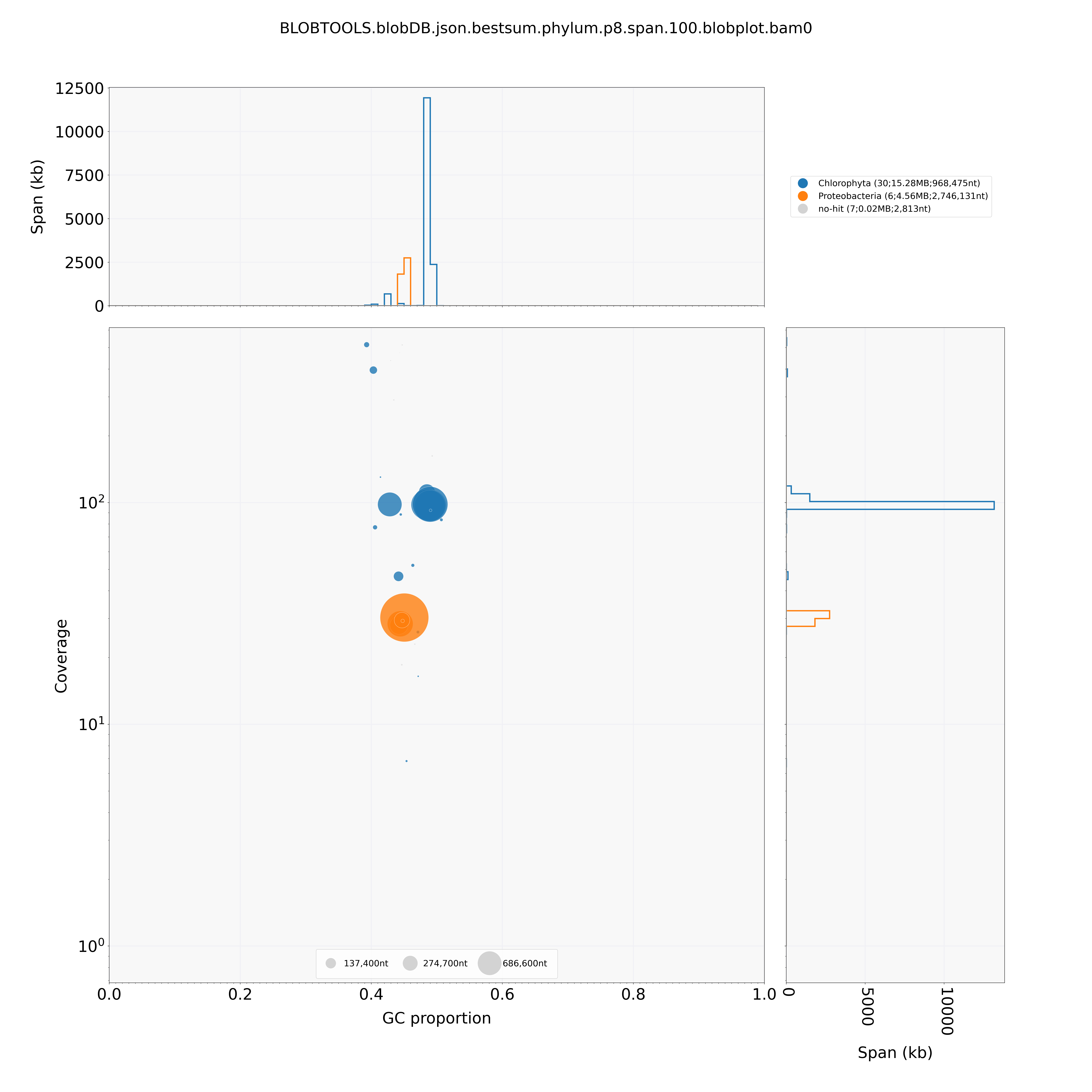

Supplement: Supplementary file 2 — Data S2. Taxonomic partitioning of assembled contigs. [file TPJ-126-0-s002.zip › blobtoolsG5/BLOBTOOLS.blobDB.json.bestsum.phylum.p8.span.100.blobplot.bam0.png]

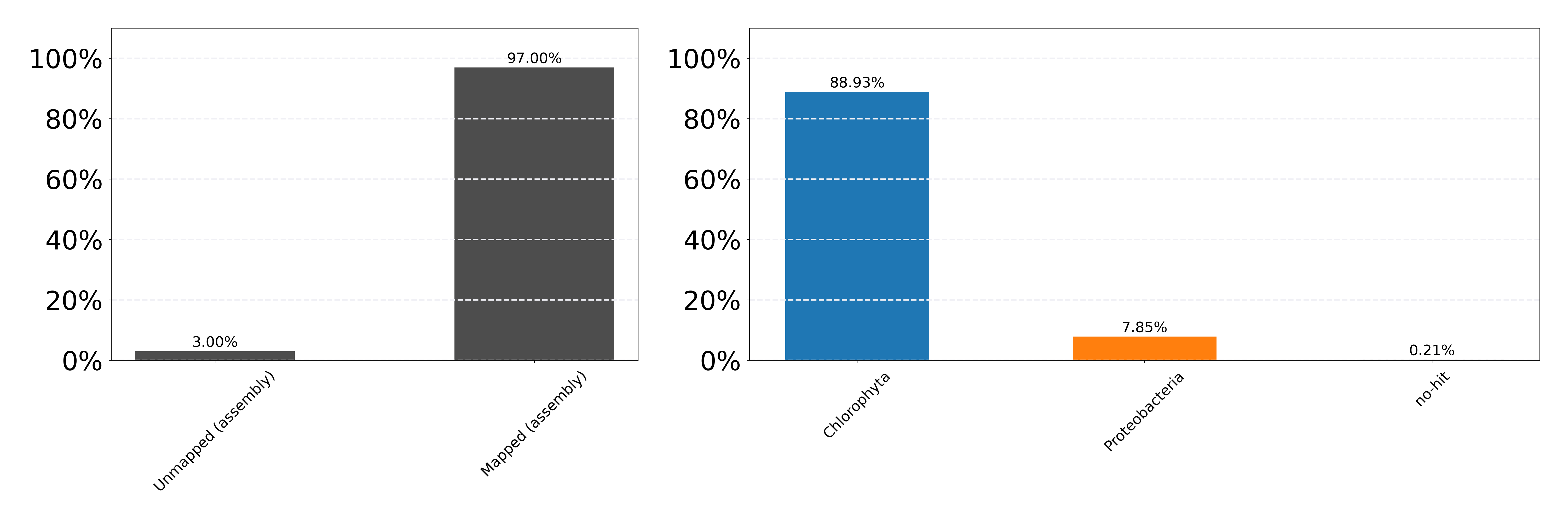

Supplement: Supplementary file 2 — Data S2. Taxonomic partitioning of assembled contigs. [file TPJ-126-0-s002.zip › blobtoolsG5/BLOBTOOLS.blobDB.json.bestsum.phylum.p8.span.100.blobplot.read_cov.bam0.png]

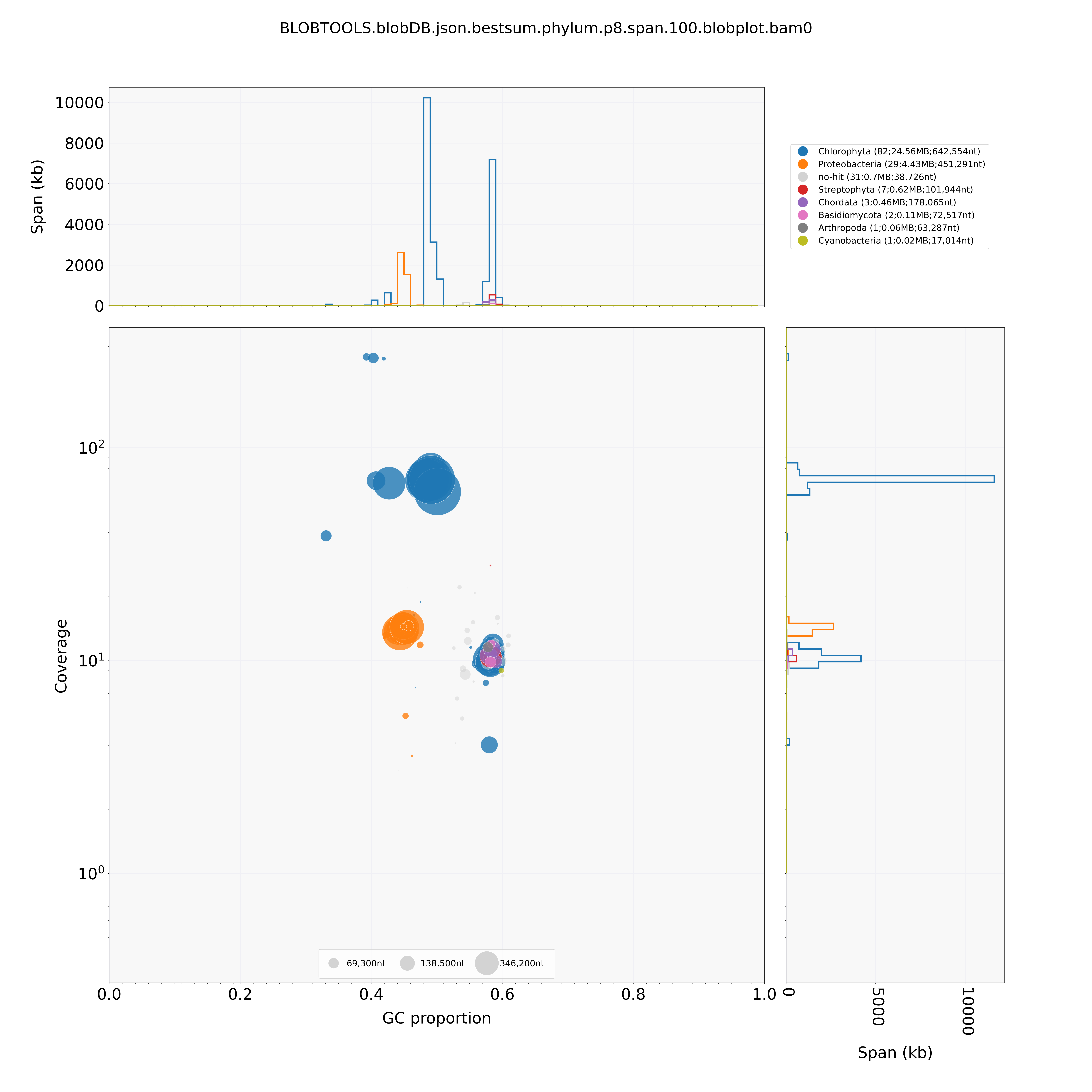

Supplement: Supplementary file 2 — Data S2. Taxonomic partitioning of assembled contigs. [file TPJ-126-0-s002.zip › blobtoolsG8/BLOBTOOLS.blobDB.json.bestsum.phylum.p8.span.100.blobplot.bam0.png]

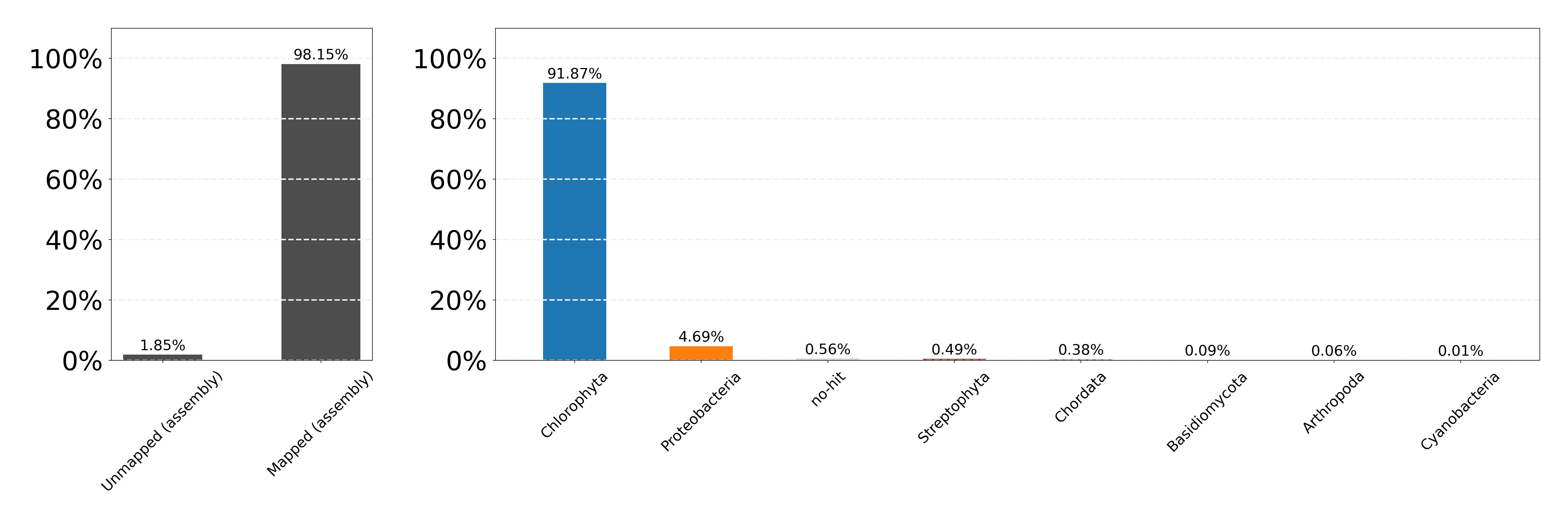

Supplement: Supplementary file 2 — Data S2. Taxonomic partitioning of assembled contigs. [file TPJ-126-0-s002.zip › blobtoolsG8/BLOBTOOLS.blobDB.json.bestsum.phylum.p8.span.100.blobplot.read_cov.bam0.png]

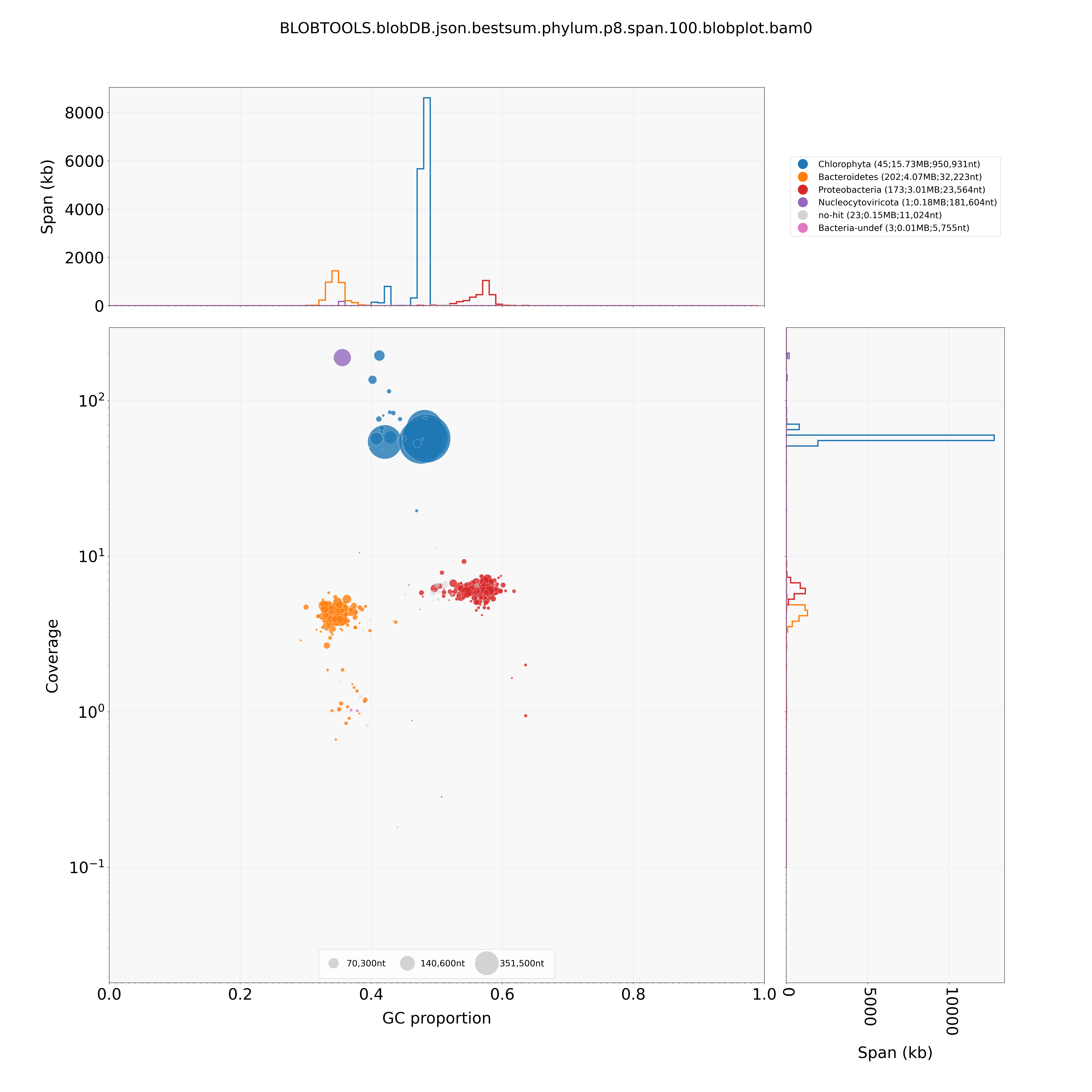

Supplement: Supplementary file 2 — Data S2. Taxonomic partitioning of assembled contigs. [file TPJ-126-0-s002.zip › blobtoolsH718/BLOBTOOLS.blobDB.json.bestsum.phylum.p8.span.100.blobplot.bam0.png]

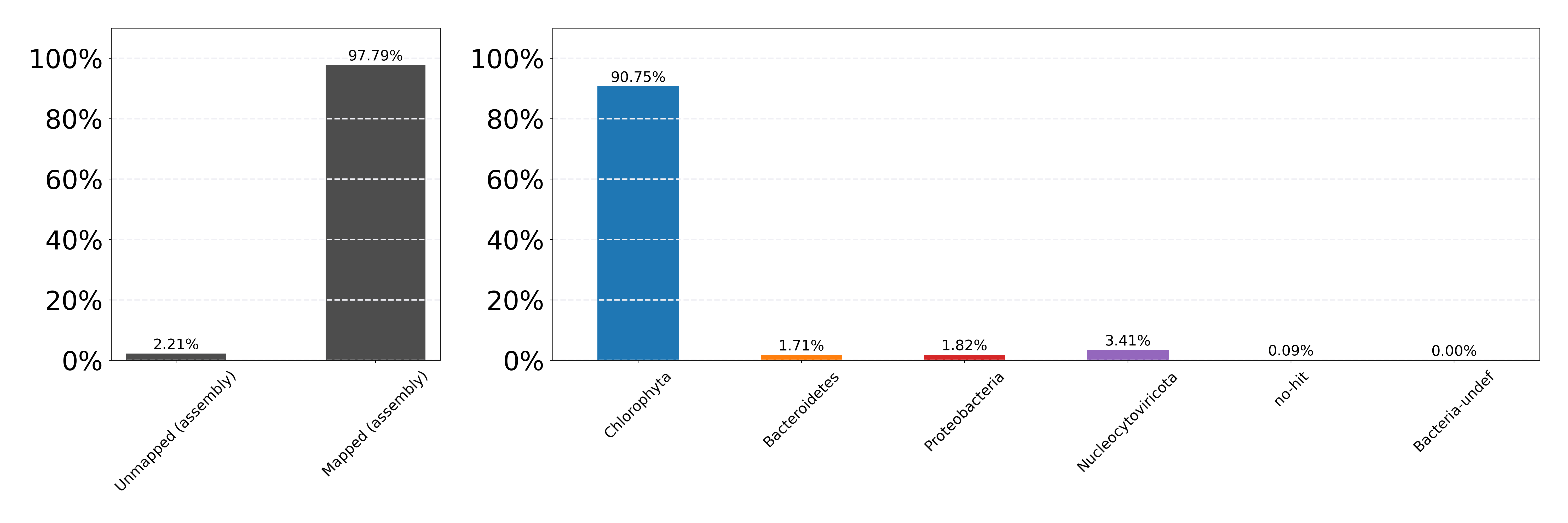

Supplement: Supplementary file 2 — Data S2. Taxonomic partitioning of assembled contigs. [file TPJ-126-0-s002.zip › blobtoolsH718/BLOBTOOLS.blobDB.json.bestsum.phylum.p8.span.100.blobplot.read_cov.bam0.png]

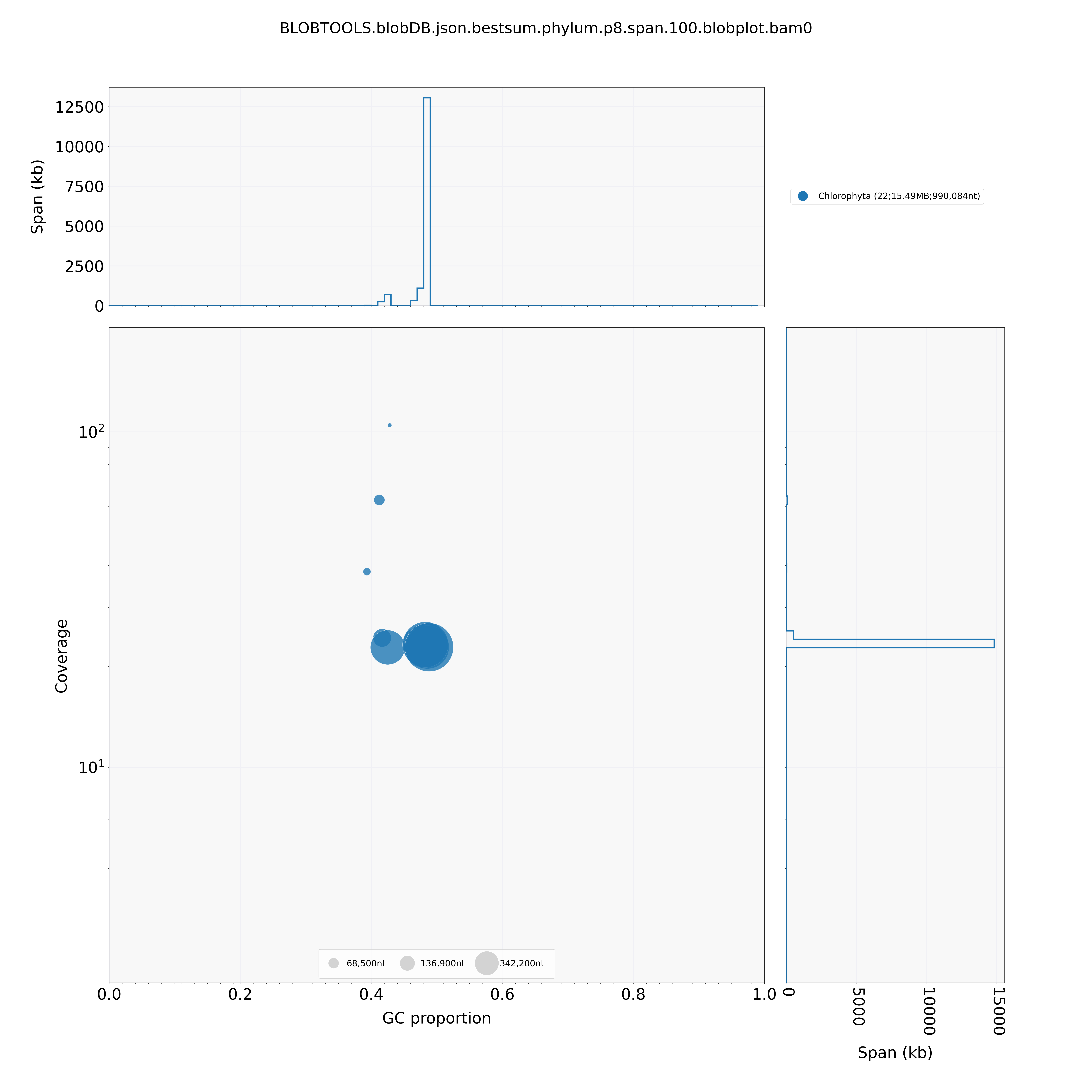

Supplement: Supplementary file 2 — Data S2. Taxonomic partitioning of assembled contigs. [file TPJ-126-0-s002.zip › blobtoolsRCC1613/BLOBTOOLS.blobDB.json.bestsum.phylum.p8.span.100.blobplot.bam0.png]

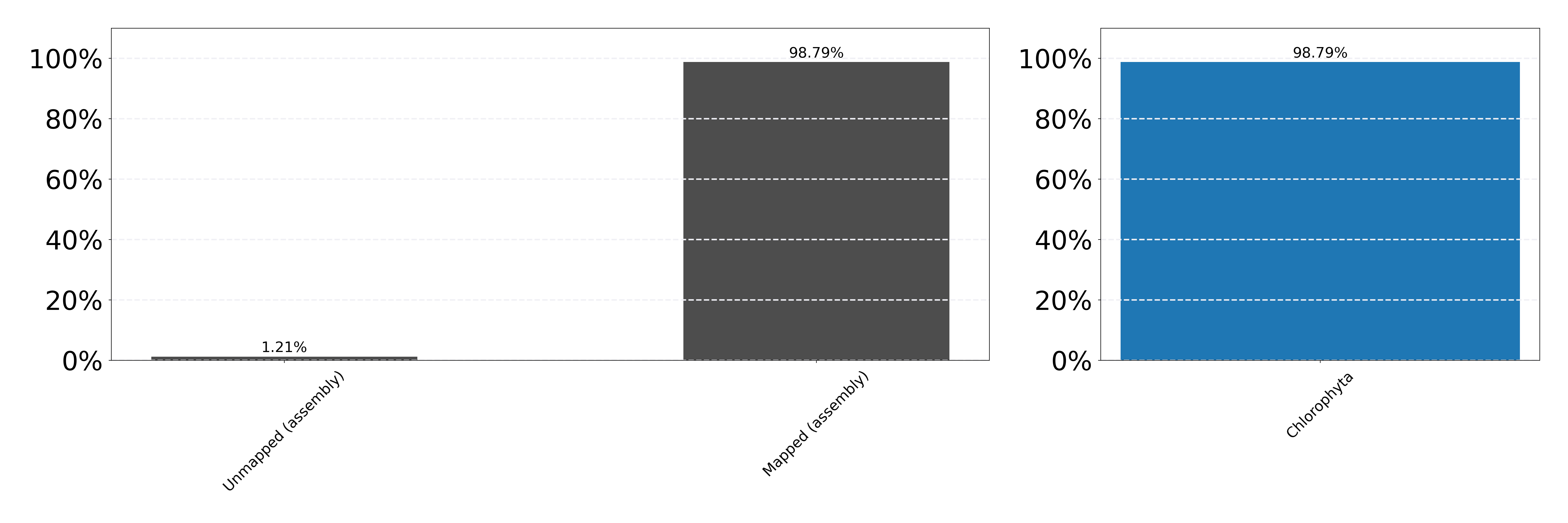

Supplement: Supplementary file 2 — Data S2. Taxonomic partitioning of assembled contigs. [file TPJ-126-0-s002.zip › blobtoolsRCC1613/BLOBTOOLS.blobDB.json.bestsum.phylum.p8.span.100.blobplot.read_cov.bam0.png]

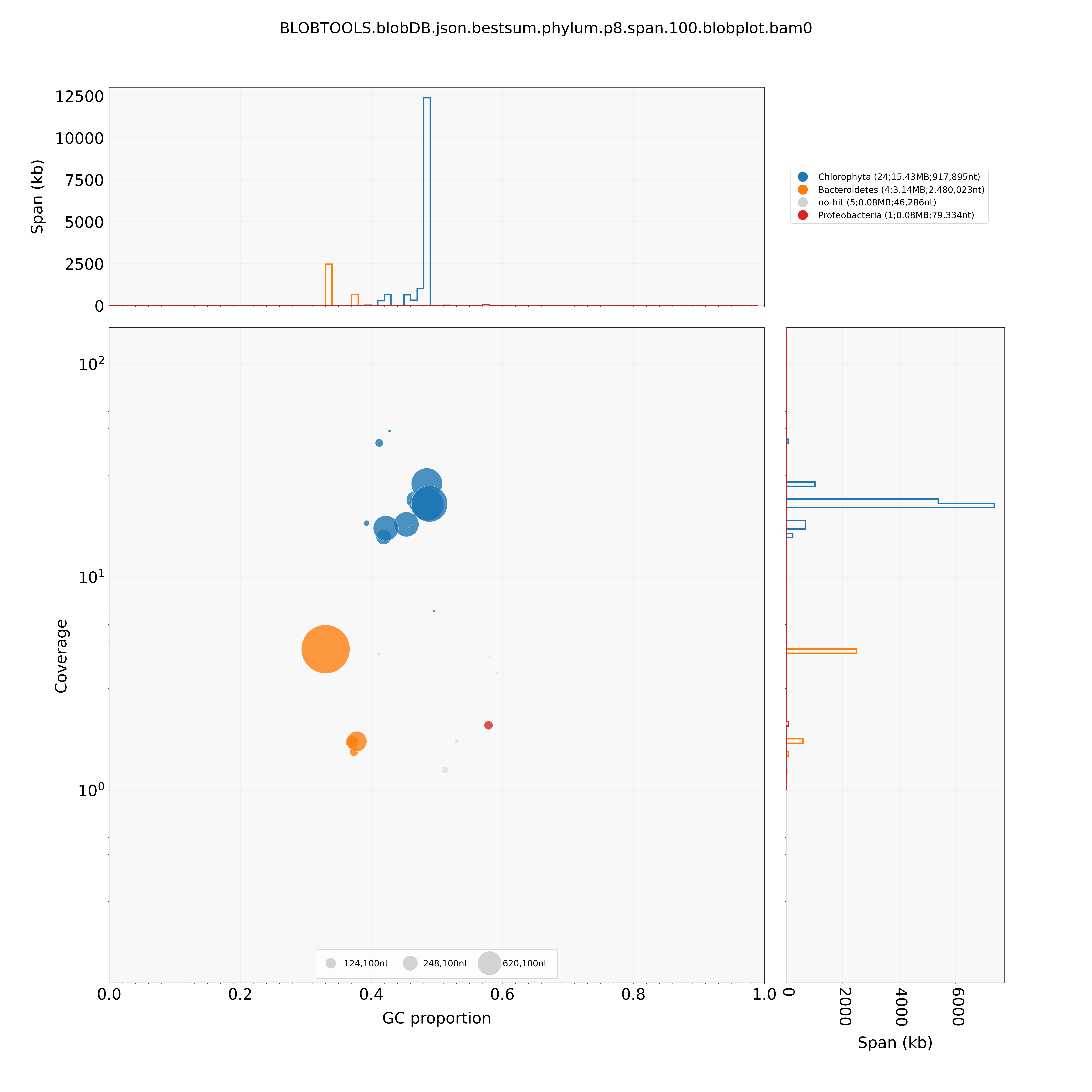

Supplement: Supplementary file 2 — Data S2. Taxonomic partitioning of assembled contigs. [file TPJ-126-0-s002.zip › blobtoolsRCC1615/BLOBTOOLS.blobDB.json.bestsum.phylum.p8.span.100.blobplot.bam0.png]

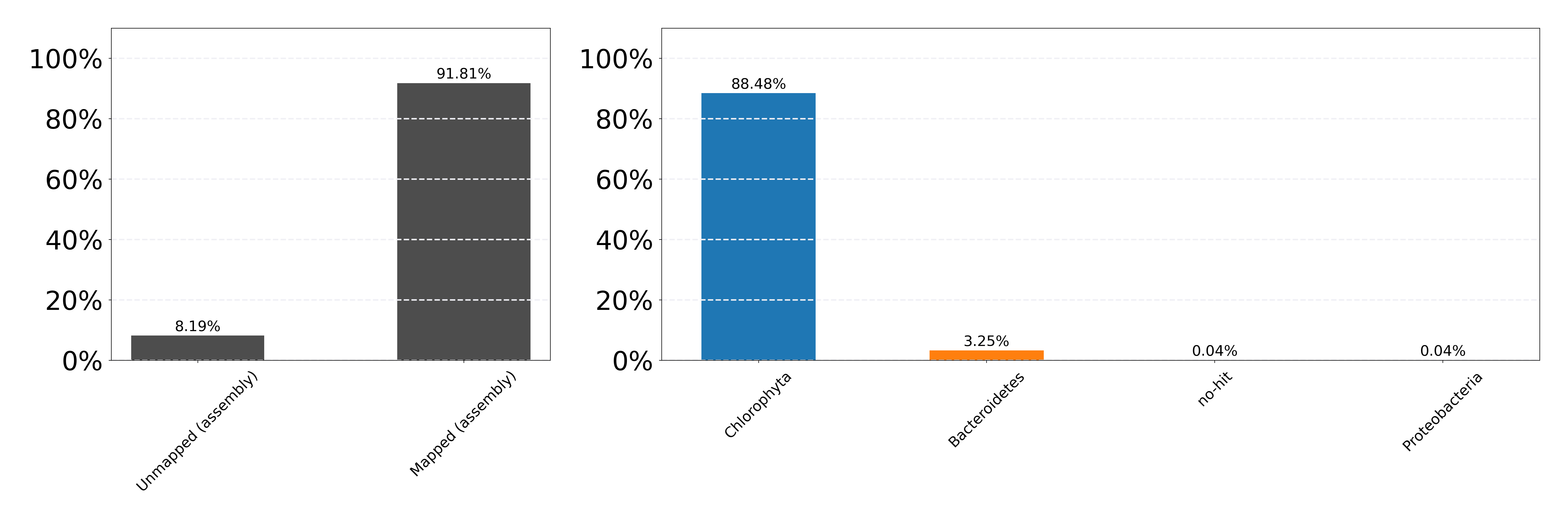

Supplement: Supplementary file 2 — Data S2. Taxonomic partitioning of assembled contigs. [file TPJ-126-0-s002.zip › blobtoolsRCC1615/BLOBTOOLS.blobDB.json.bestsum.phylum.p8.span.100.blobplot.read_cov.bam0.png]

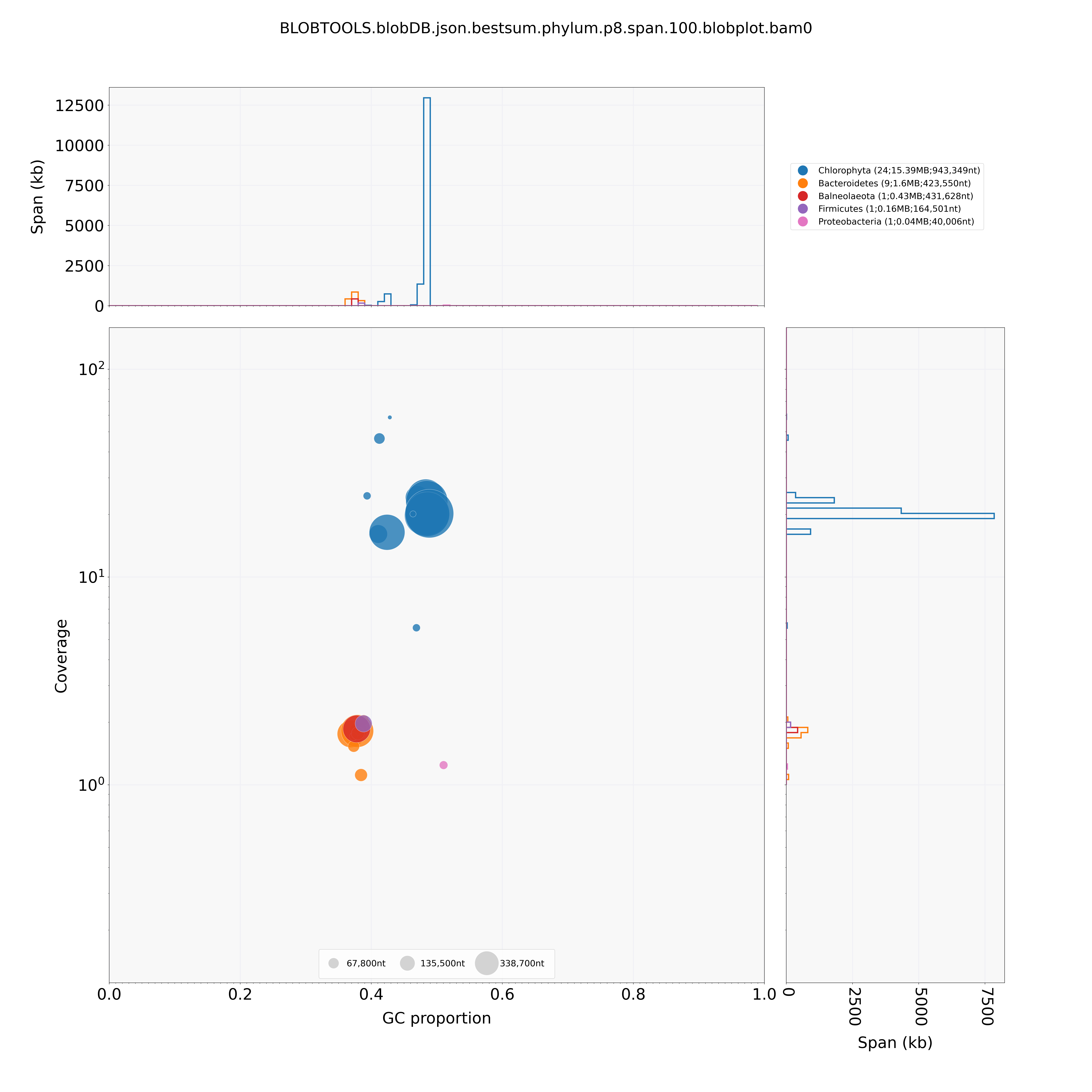

Supplement: Supplementary file 2 — Data S2. Taxonomic partitioning of assembled contigs. [file TPJ-126-0-s002.zip › blobtoolsRCC1868/BLOBTOOLS.blobDB.json.bestsum.phylum.p8.span.100.blobplot.bam0.png]

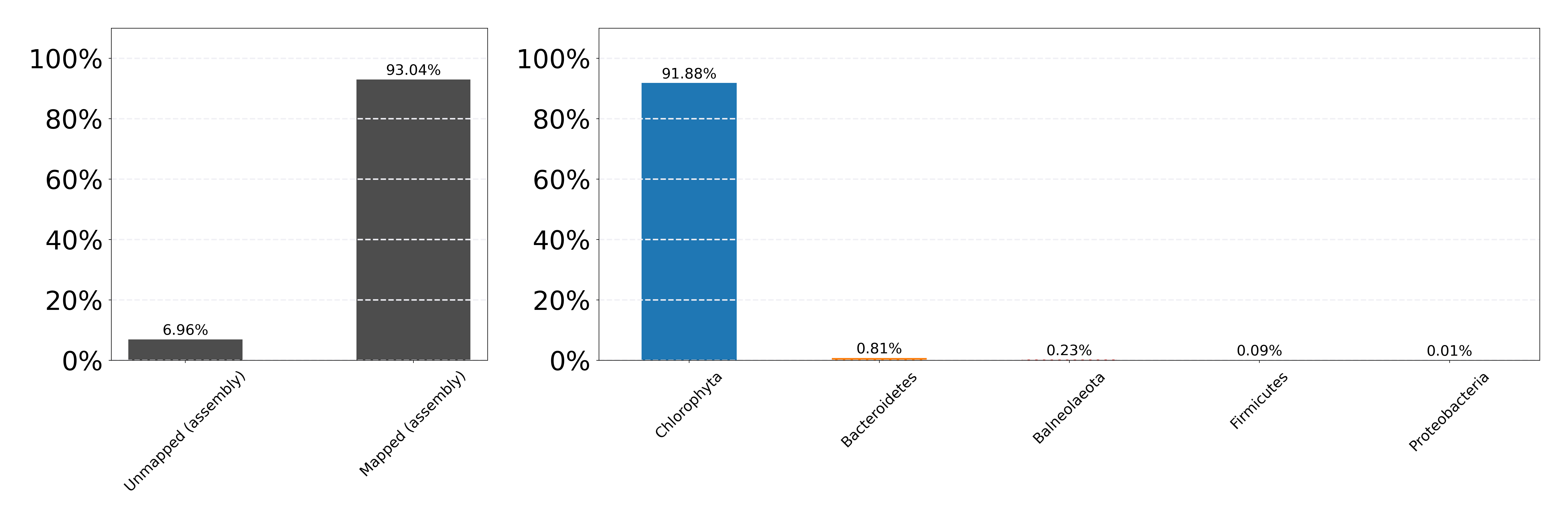

Supplement: Supplementary file 2 — Data S2. Taxonomic partitioning of assembled contigs. [file TPJ-126-0-s002.zip › blobtoolsRCC1868/BLOBTOOLS.blobDB.json.bestsum.phylum.p8.span.100.blobplot.read_cov.bam0.png]

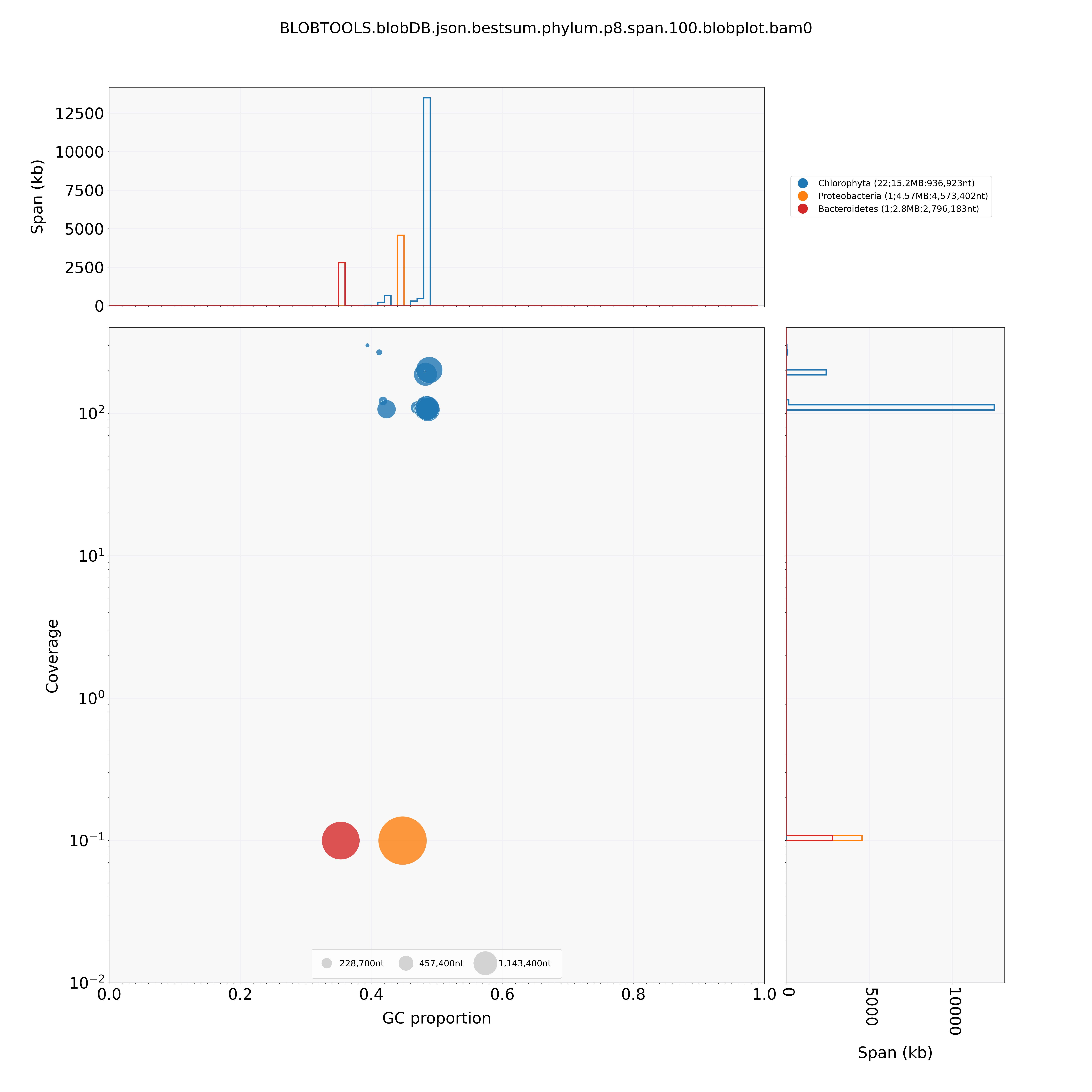

Supplement: Supplementary file 2 — Data S2. Taxonomic partitioning of assembled contigs. [file TPJ-126-0-s002.zip › blobtoolsRCC4222/BLOBTOOLS.blobDB.json.bestsum.phylum.p8.span.100.blobplot.bam0.png]

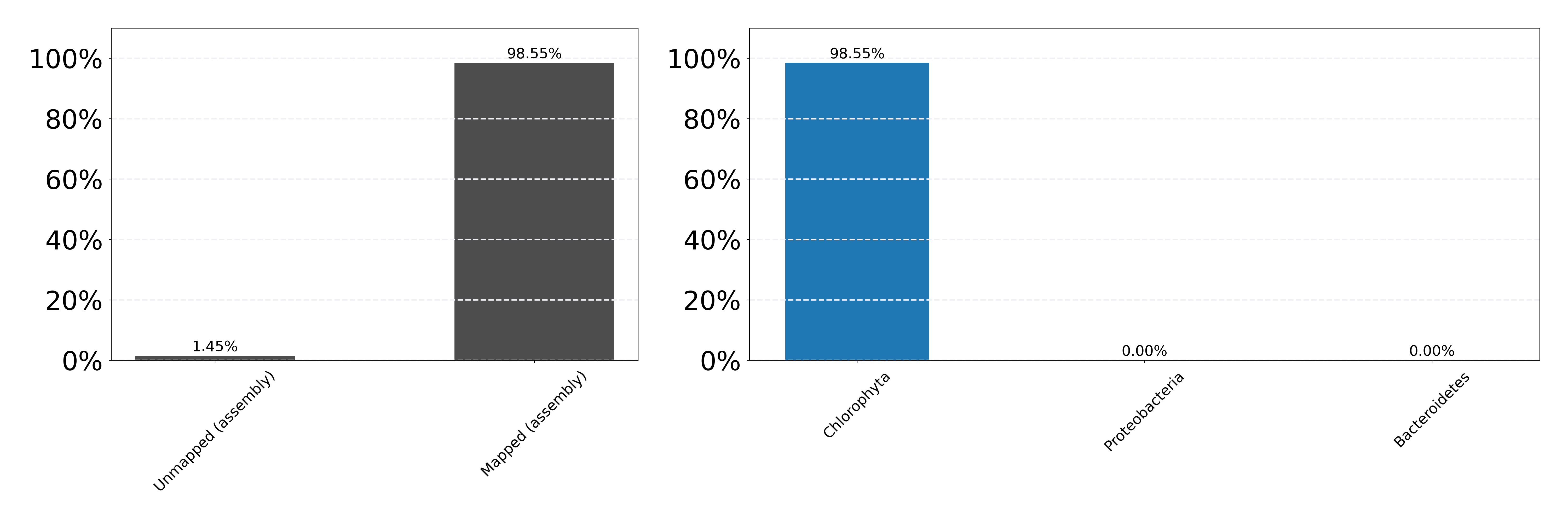

Supplement: Supplementary file 2 — Data S2. Taxonomic partitioning of assembled contigs. [file TPJ-126-0-s002.zip › blobtoolsRCC4222/BLOBTOOLS.blobDB.json.bestsum.phylum.p8.span.100.blobplot.read_cov.bam0.png]

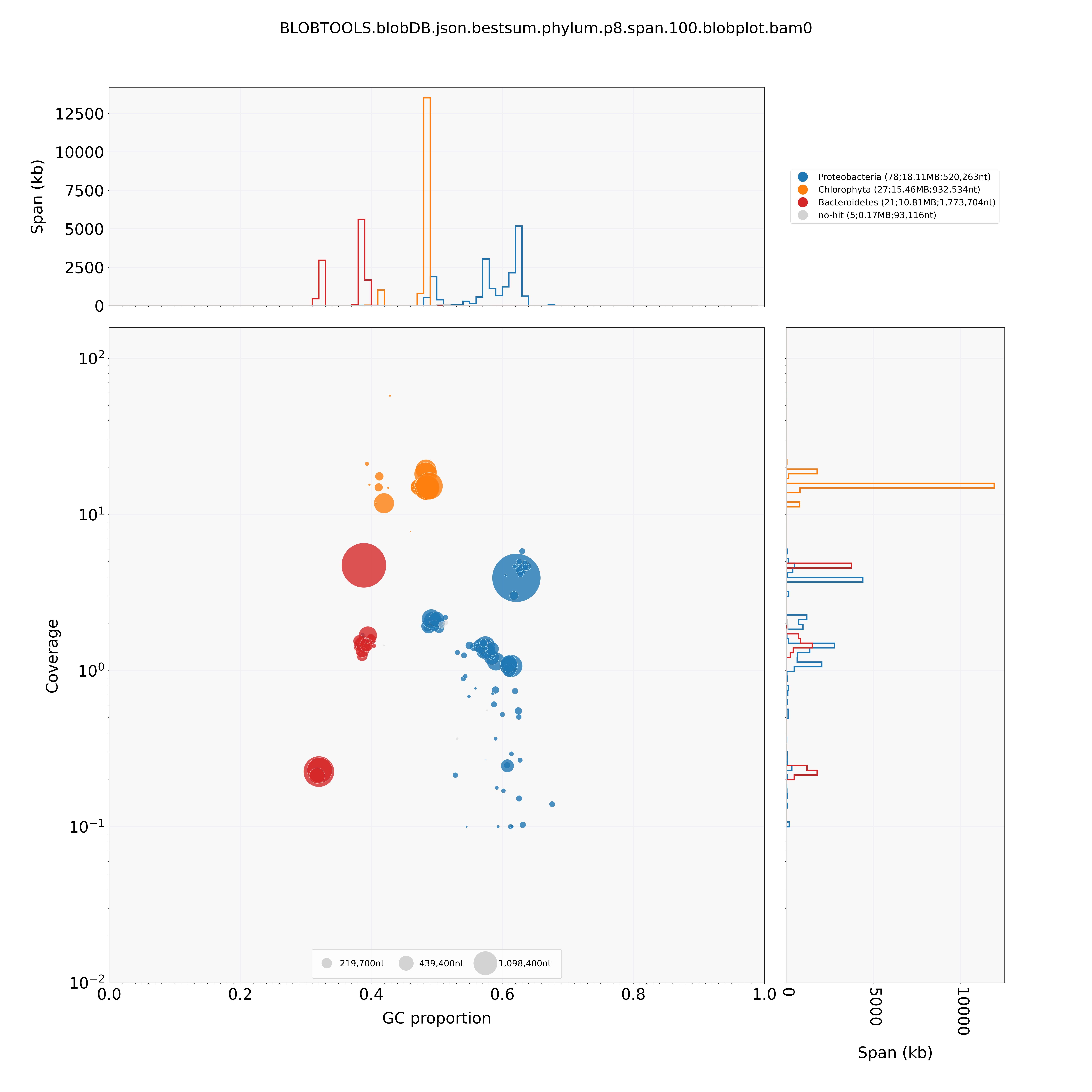

Supplement: Supplementary file 2 — Data S2. Taxonomic partitioning of assembled contigs. [file TPJ-126-0-s002.zip › blobtoolsRCC4752/BLOBTOOLS.blobDB.json.bestsum.phylum.p8.span.100.blobplot.bam0.png]

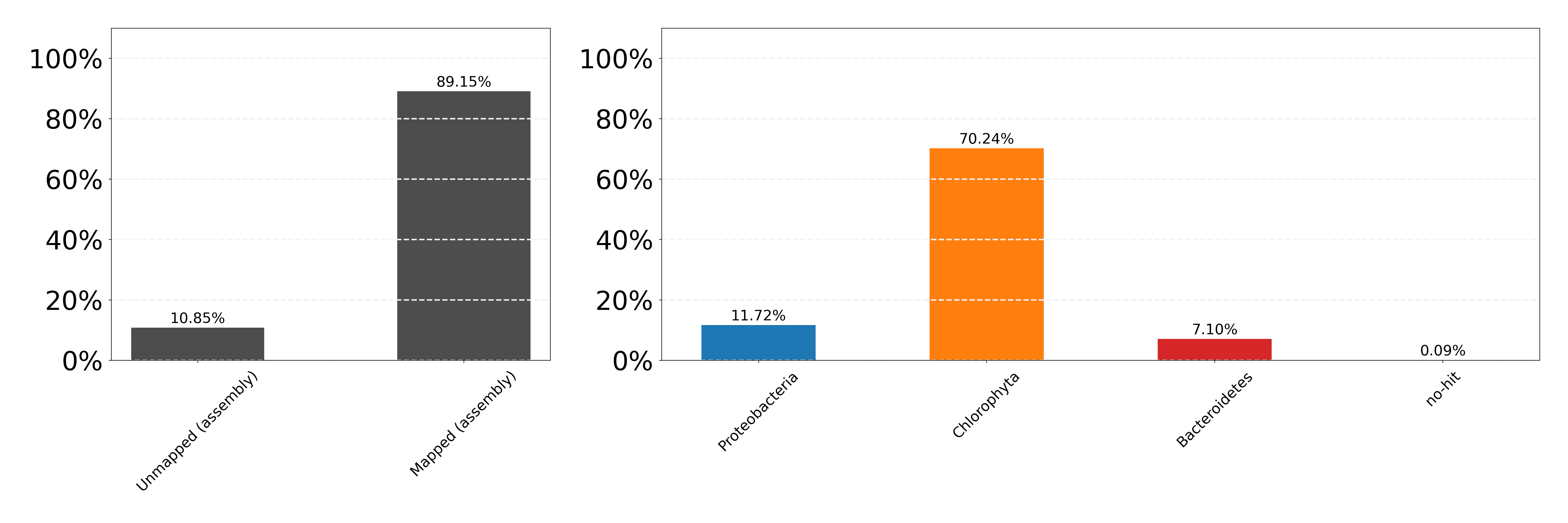

Supplement: Supplementary file 2 — Data S2. Taxonomic partitioning of assembled contigs. [file TPJ-126-0-s002.zip › blobtoolsRCC4752/BLOBTOOLS.blobDB.json.bestsum.phylum.p8.span.100.blobplot.read_cov.bam0.png]

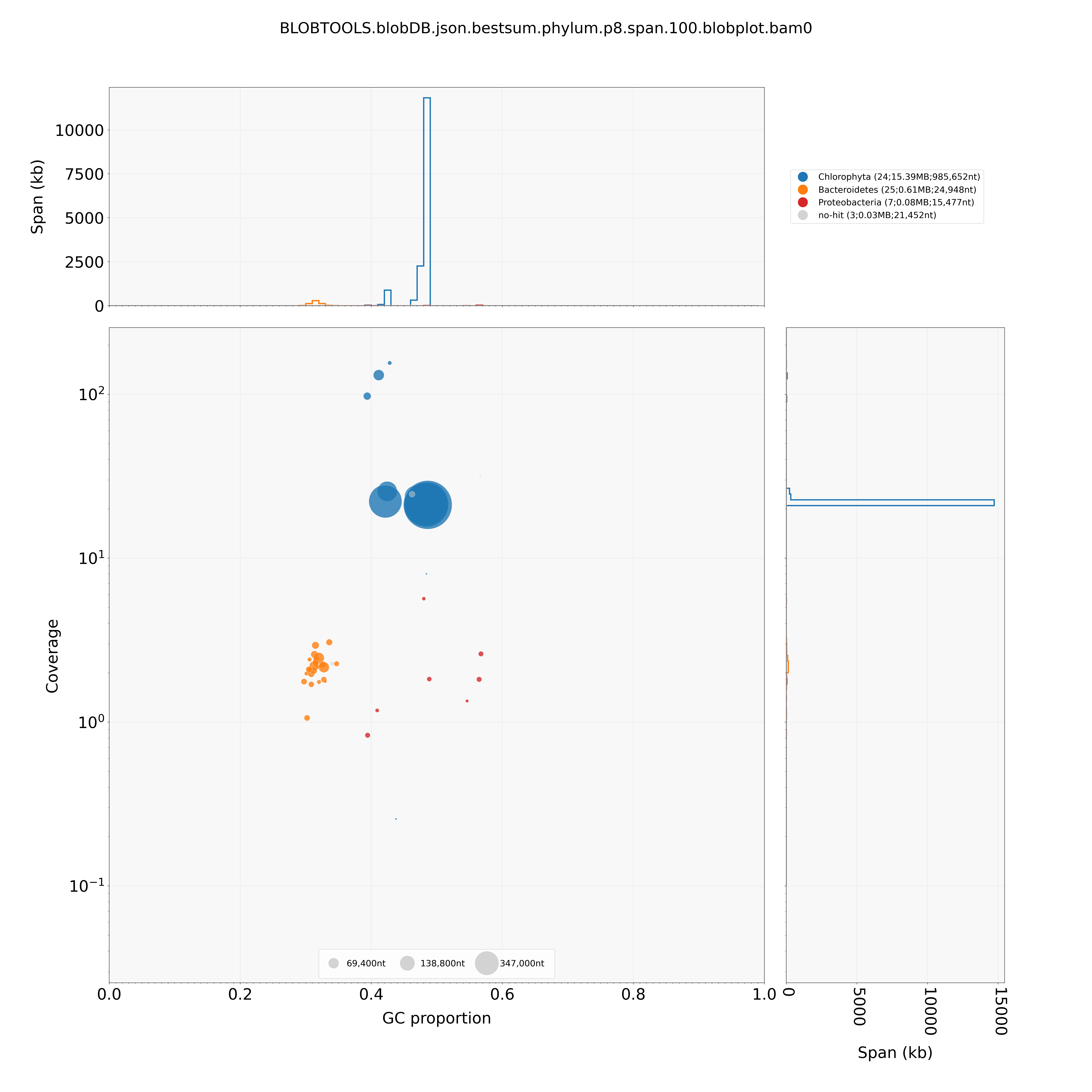

Supplement: Supplementary file 2 — Data S2. Taxonomic partitioning of assembled contigs. [file TPJ-126-0-s002.zip › blobtoolsRCC5417/BLOBTOOLS.blobDB.json.bestsum.phylum.p8.span.100.blobplot.bam0.png]

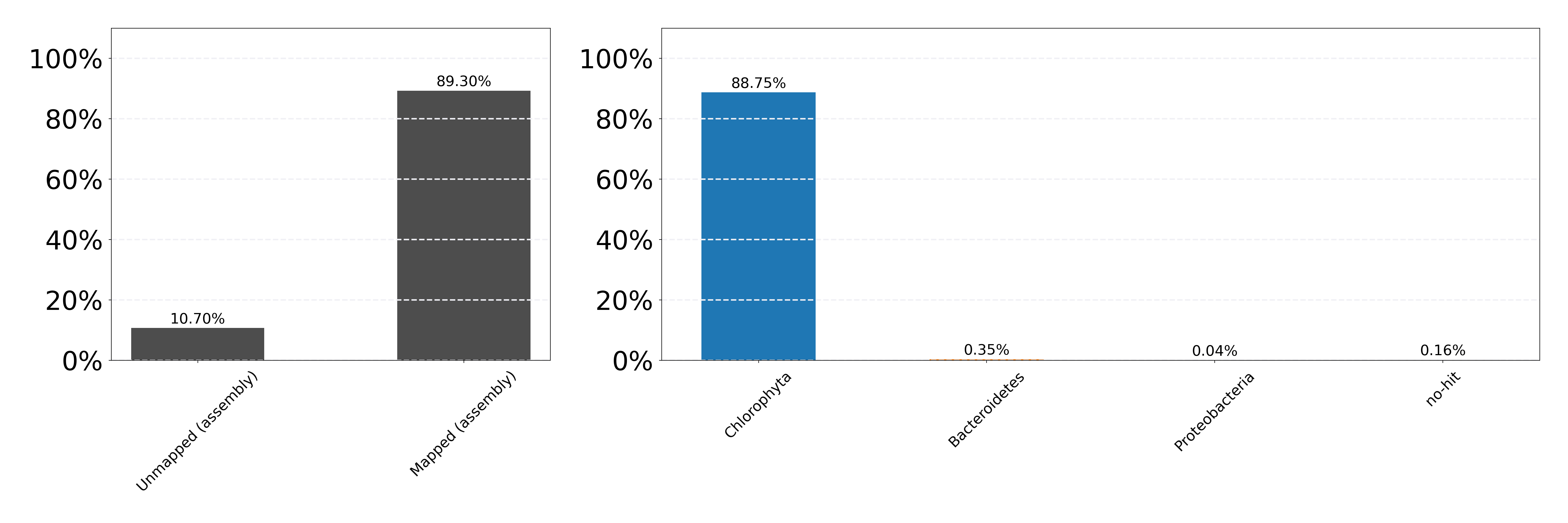

Supplement: Supplementary file 2 — Data S2. Taxonomic partitioning of assembled contigs. [file TPJ-126-0-s002.zip › blobtoolsRCC5417/BLOBTOOLS.blobDB.json.bestsum.phylum.p8.span.100.blobplot.read_cov.bam0.png]

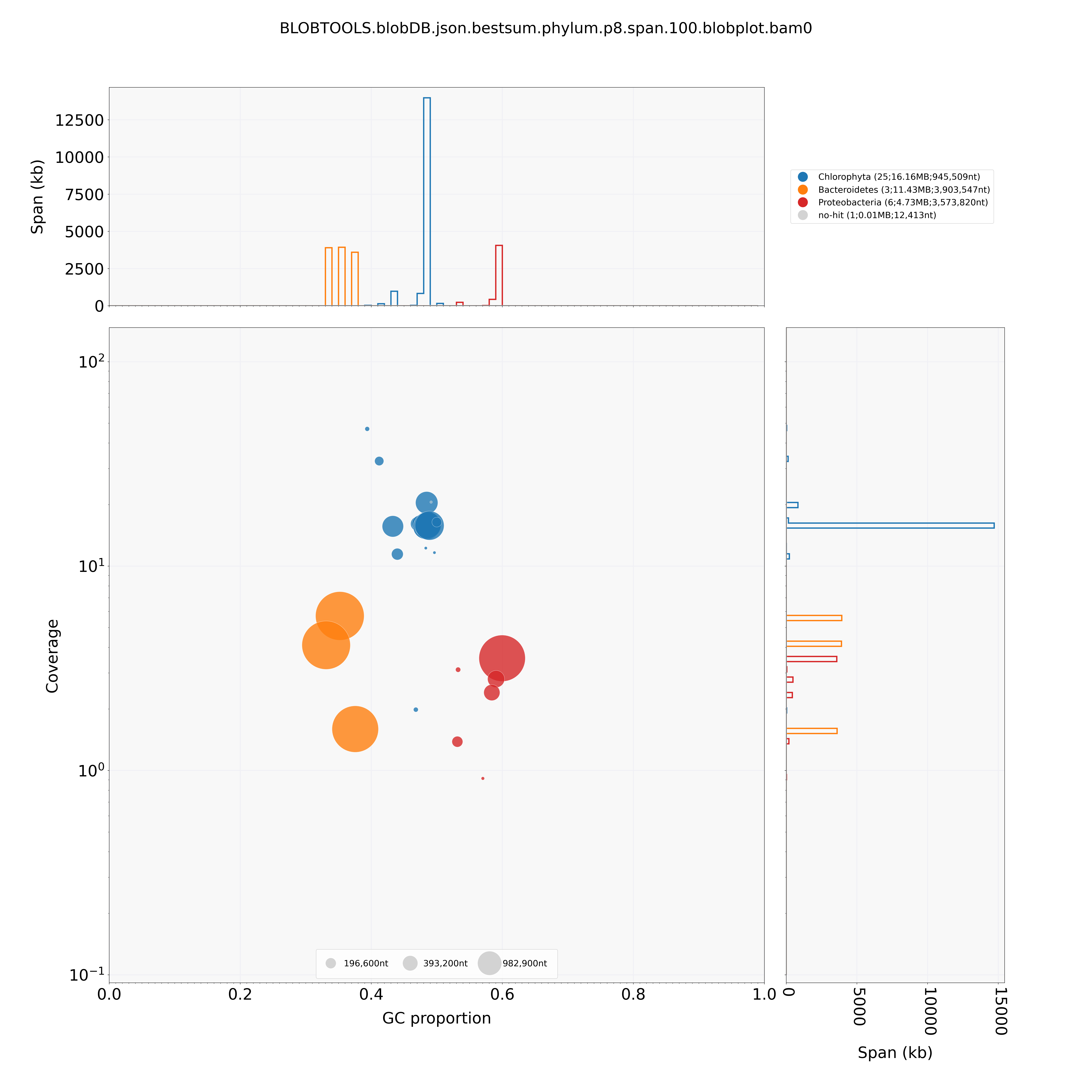

Supplement: Supplementary file 2 — Data S2. Taxonomic partitioning of assembled contigs. [file TPJ-126-0-s002.zip › blobtoolsRCC685/BLOBTOOLS.blobDB.json.bestsum.phylum.p8.span.100.blobplot.bam0.png]

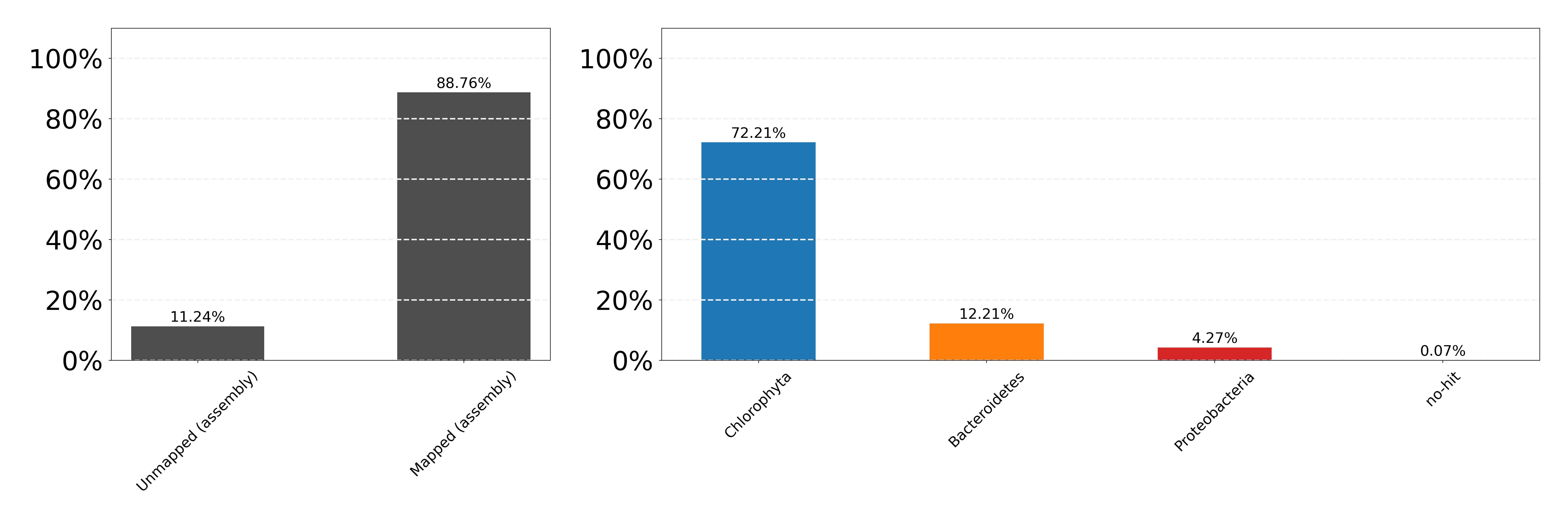

Supplement: Supplementary file 2 — Data S2. Taxonomic partitioning of assembled contigs. [file TPJ-126-0-s002.zip › blobtoolsRCC685/BLOBTOOLS.blobDB.json.bestsum.phylum.p8.span.100.blobplot.read_cov.bam0.png]

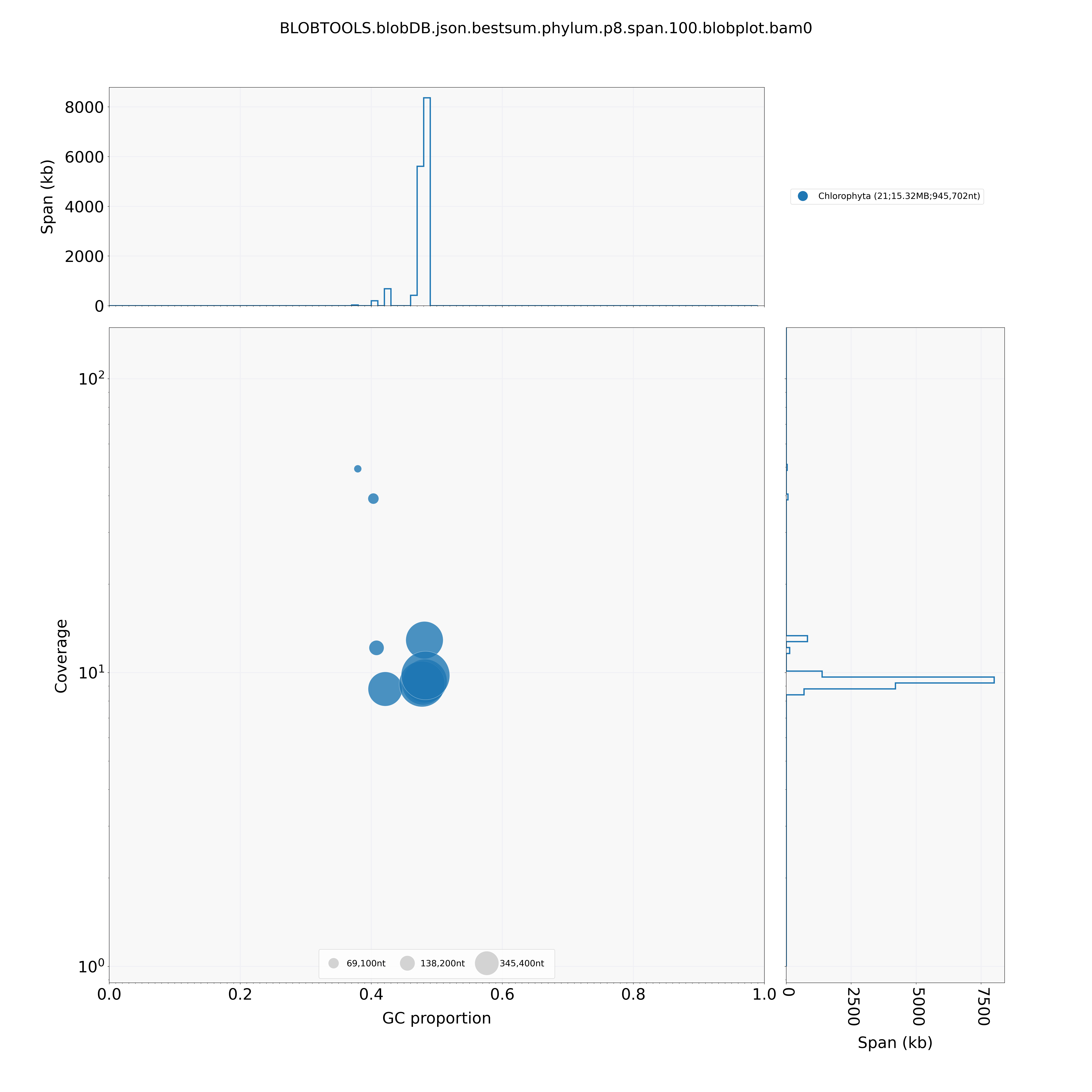

Supplement: Supplementary file 2 — Data S2. Taxonomic partitioning of assembled contigs. [file TPJ-126-0-s002.zip › blobtoolsRCC716/BLOBTOOLS.blobDB.json.bestsum.phylum.p8.span.100.blobplot.bam0.png]

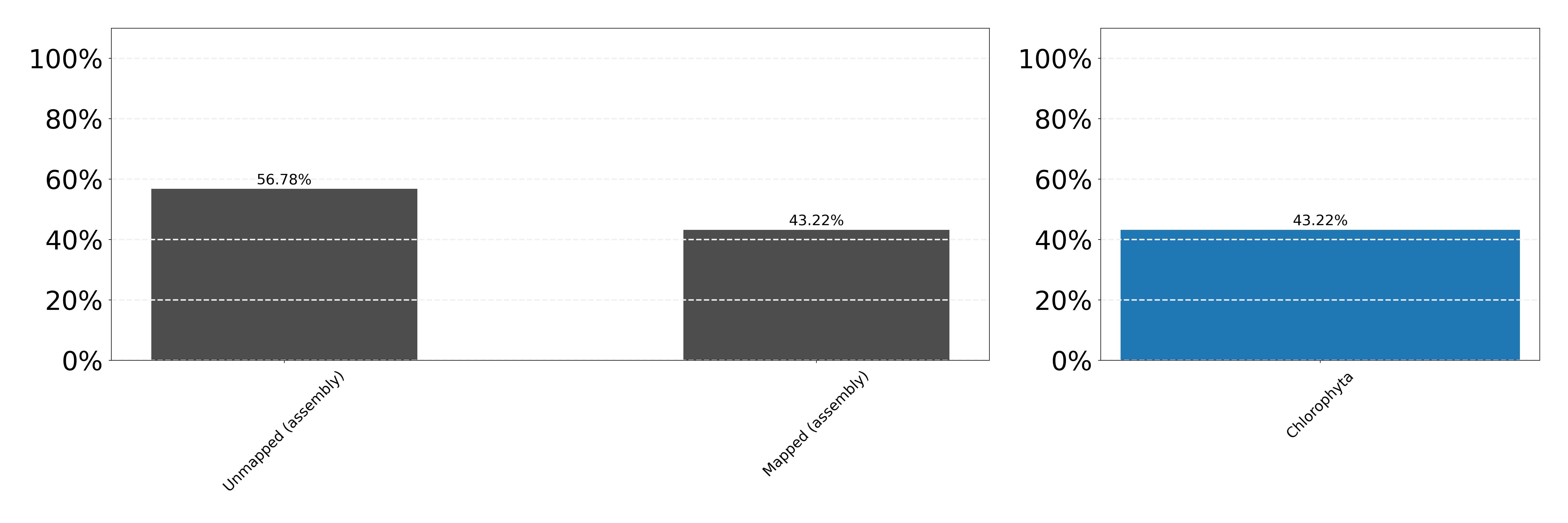

Supplement: Supplementary file 2 — Data S2. Taxonomic partitioning of assembled contigs. [file TPJ-126-0-s002.zip › blobtoolsRCC716/BLOBTOOLS.blobDB.json.bestsum.phylum.p8.span.100.blobplot.read_cov.bam0.png]
